# Copper-catalyzed diamination of unactivated alkenes with hydroxylamines†
†Electronic supplementary information (ESI) available: Characterization data and experimental procedures. See DOI: 10.1039/c5sc00897b


**DOI:** 10.1039/c5sc00897b

**Published:** 2015-05-18

**Authors:** Kun Shen, Qiu Wang

**Affiliations:** a Department of Chemistry , Duke University , Durham , NC 27708–0346 , US . Email: qiu.wang@duke.edu

## Abstract

A copper-catalyzed regio- and stereoselective diamination of unactivated alkenes has been developed with *O*-acylhydroxylamines as electrophilic nitrogen sources and oxidants. This method provides the first example of metal-catalyzed alkene diamination for directly installing an electron-rich amino group and extends the diamination scope for the synthesis of diverse 1,2-diamines. It offers a rapid and efficient approach to construct a wide range of 1,2-diamines that are an important structural motif in organic synthesis, medicines, catalysts and ligands.

## Abstract 2

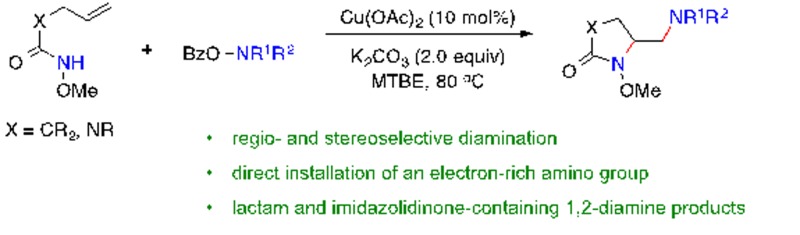
A copper-catalyzed regio- and stereoselective diamination of unactivated alkenes has been developed with *O*-acylhydroxylamines as electrophilic nitrogen sources and oxidants.

## Introduction

**Scheme 1:**
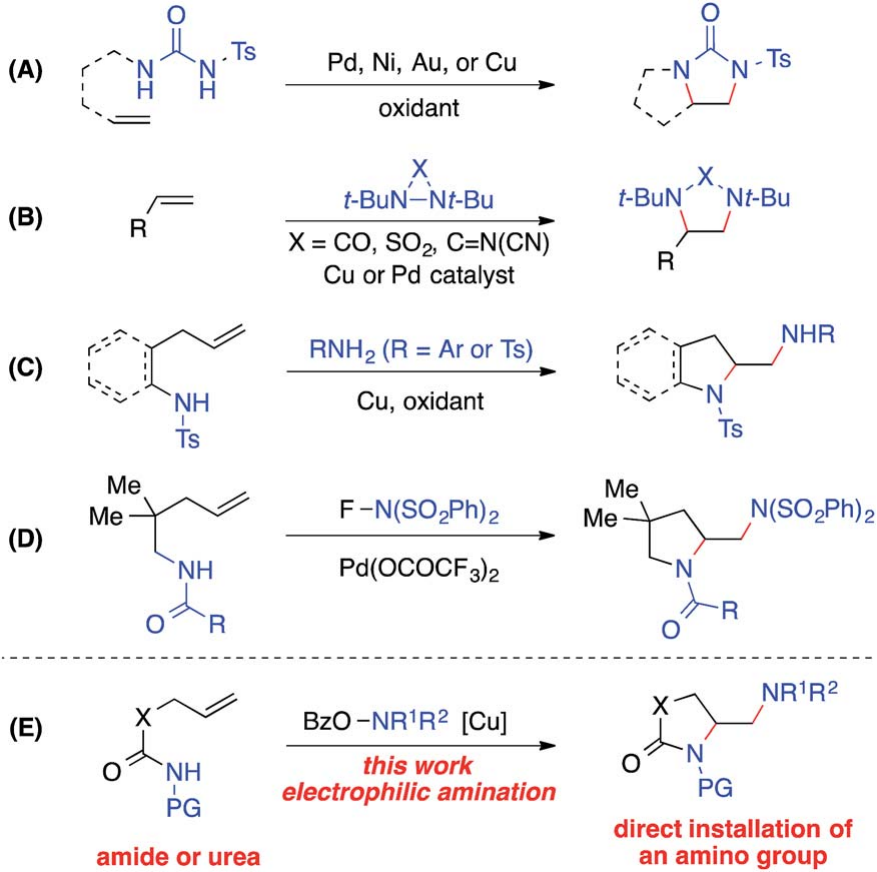
Metal-catalyzed alkene diaminations.

The 1,2-diamine moiety is widely represented in bioactive compounds, synthetic building blocks, catalysts, ligands, and medicines.^1^ Alkene diamination reactions that can directly add two amino groups across a double bond provide a straightforward route to the synthesis of 1,2-diamines and are therefore of great interest.^2^ A number of elegant transition-metal-catalyzed intra- and intermolecular diamination reactions have been reported (Scheme 1).^3^–^5^ Oxidative diaminations of alkenes have been developed with Pd, Ni, Au or Cu as a catalyst using *N*,*N*′-disubstituted ureas as nitrogen sources (Scheme 1, **A**).^6^ The Shi group employed diaziridines as both nitrogen sources and oxidants and established the Cu- and Pd-catalyzed intermolecular alkene diamination reactions (Scheme 1, **B**).^7^ Recently, Chemler reported a copper-catalyzed diamination with free sulfonamides or anilines in the second intermolecular C–N bond-forming step in the presence of an oxidant (Scheme 1, **C**),^8^ and the Michael group reported a Pd(ii)-catalyzed alkene diamination using electrophilic *N*-fluorobenzenesulfonimide in the intermolecular amination step as its nitrogen source and oxidant (Scheme 1, **D**).^9^

Despite these important advances in alkene diamination, the direct installation of an amino group in metal-catalyzed diamination has not been reported,^5^ likely because electron-rich free amines would lead to strong coordination to the metal and subsequent catalyst poisoning.^2^,^3^ So far, the introduction of an amino group has to be derived from nitrogen sources that are compatible with metal-catalyzed diaminations, such as ureas, sulfonamides, and anilines. Thus, the resulting amino group is restricted to primary and secondary amines, in many cases, an NH_2_ group.^2^,^3^ Furthermore, most intramolecular diamination reactions provided pyrrolidine-containing diamines. Few examples starting from unsaturated amide precursors were achieved for the synthesis of lactam-containing diamination products.8*b* Therefore, it is of great value to develop new alkene diaminations that can directly incorporate an amino group for the synthesis of more diverse 1,2-diamine skeletons.

Herein we report a copper-catalyzed alkene diamination that achieves, for the first time, the direct installation of an amino group *via* an intermolecular electrophilic amination step. Recently, our group reported the direct amination of sp^2^ and sp^3^ C–H bonds^10^*via* copper-catalyzed electrophilic amination using hydroxylamine as an amine source.^11^,^12^ We postulated that such an intermolecular electrophilic amination, in conjunction with copper-catalyzed aminocyclization, would offer an attractive alkene diamination strategy to achieve the direct addition of an amino group (Scheme 1, **E**). Particularly advantageous is the use of *O*-acylhydroxylamines, readily available from diverse amino precursors, with the dual role of an amine source and oxidant in the proposed diamination reaction. It bypasses the poisoning interference of a free amine group with the catalyst, offers a source for diverse amino groups, and eliminates the need of excess external oxidants required in an oxidative diamination reaction. Thus, the development of this new method will provide a rapid and efficient approach to 1,2-diamino skeletons, especially those containing cyclic and acyclic tertiary amines or lactam-based 1,2-diamines that would be inaccessible by other metal-catalyzed diaminations. Furthermore, the wide applicability of this strategy renders it highly valuable for the synthesis of important 1,2-diamines in catalysis, biological studies, and medicinal chemistry.

## Results and discussion

Our studies began with the diamination of unsaturated amide **1a** with *O*-benzoyl hydroxylmorpholine **2a** (Table 1). Preliminary studies found that the desired diamination product was observed in 68% yield in the presence of Cu(OAc)_2_ catalyst in toluene at 80 °C (Table 1, entry 1). Among different solvents, MTBE was best for the formation of the desired product **3aa** (entries 2–5). When different copper catalysts were examined, Cu(OAc)_2_ remained most effective, providing **3aa** in 80% yield (entries 5–10). The control experiment proved that no product was formed in the absence of a copper catalyst (entry 11). During the studies, we recognized that the protecting group (PG) played an important role in this reaction. Similar to *N*-methoxy amide **1a**, *N*-benzyloxy amide **1b** smoothly reacted with hydroxylamine **2a**, giving diamination product **3ba** in 74% yield. However, similar substrates bearing other *N*-protecting groups such as the tosyl, benzyl, or the free amide did not afford the desired products (entries 13–15). These results suggested the critical role of alkoxyl group^13^ on the nitrogen, which might coordinate and stabilize the alkyl-copper intermediate resulting from the alkene aminocupration step.

**Table 1:** Condition optimization for copper-catalyzed intra-/inter-molecular alkene diamination of unsaturated amide with hydroxylamine **2a**^*a*^

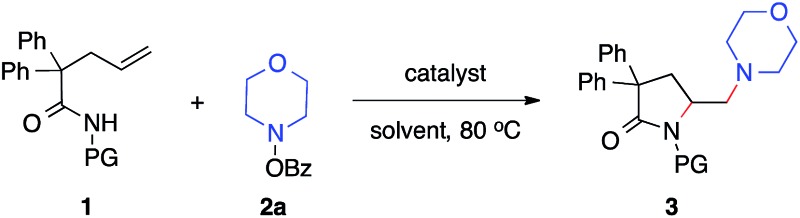				
Entry	**1**	Catalyst	Solvent	**3**, yield^*b*^ [%]
1	**1a**, PG = OMe	Cu(OAc)_2_	Toluene	**3aa**, 68
2	**1a**	Cu(OAc)_2_	DCE	**3aa**, 74
3	**1a**	Cu(OAc)_2_	THF	**3aa**, 48
4	**1a**	Cu(OAc)_2_	CH_3_CN	**3aa**, 70
**5**	**1a**	**Cu(OAc)**_**2**_	**MTBE**	**3aa, 84 (80)** ^*c*^
6	**1a**	Cu(CF_3_COO)_2_	MTBE	**3aa**, 60
7	**1a**	Cu(OTf)_2_	MTBE	**3aa**, 46
8	**1a**	Cu(acac)_2_	MTBE	**3aa**, 24
9	**1a**	CuCl_2_	MTBE	**3aa**, 39
10	**1a**	CuOAc	MTBE	**3aa**, 60
11	**1a**	—	MTBE	**3aa**, 0^*d*^
12	**1b**, PG = OBn	Cu(OAc)_2_	MTBE	**3ba**, 81 (74)^*c*^
13	**1c**, PG = Ts	Cu(OAc)_2_	MTBE	**3ca**, 0^*d*^
14	**1d**, PG = Bn	Cu(OAc)_2_	MTBE	**3da**, 0^*d*^
15	**1e**, PG = H	Cu(OAc)_2_	MTBE	**3ea**, 0^*d*^

^*a*^Reaction conditions: **1a** (0.20 mmol, 1.0 equiv.), **2a** (1.2 equiv.), catalyst (10 mol%), K_2_CO_3_ (2.0 equiv.), solvent (1 mL), 2 h.

^*b*^Yields were determined by ^1^H NMR with CH_2_Br_2_ as an internal standard.

^*c*^The isolation yield was indicated in the parenthesis.

^*d*^Not detected by GC/MS or ^1^H NMR. PG = protecting group. DCE = 1,2-dichloroethane. MTBE = methyl *tert*-butyl ether.

With established diamination conditions, we examined the scope of this alkene diamination using different hydroxylamines (Table 2). *O*-Benzoylhydroxylamines **2a–2e**, derived from 6-membered cyclic amines such as morpholine, *N*-Boc piperazine and piperidines, all readily participated in the diamination reaction and gave corresponding 1,2-diamines **3aa–3ae** in high yields. Azepane-derived 7-membered *O*-benzoylhydroxylamine **2f** was also a viable substrate and afforded **3af** in 44% yield. The reactions with *O*-benzoylhydroxylamines derived from acyclic amines, such as *N*,*N*-diethylamine, *N*,*N*-diallylamine and *N*-methyl-*N*-benzylamine, were found to be more effective under the conditions with a higher catalyst loading and elevated temperature.^14^ Nonetheless, all underwent the diamination reaction and successfully formed the corresponding 1,2-diamine products **3ag–3ai**. Notably, the compatibility with benzyl and allyl group in these reactions offered opportunities for further transformations. For example, the cleavage of the allyl group or benzyl group can afford either a primary amine or a secondary amine.^15^

**Table 2:** Diamination reactions with different hydroxylamines^*a*^

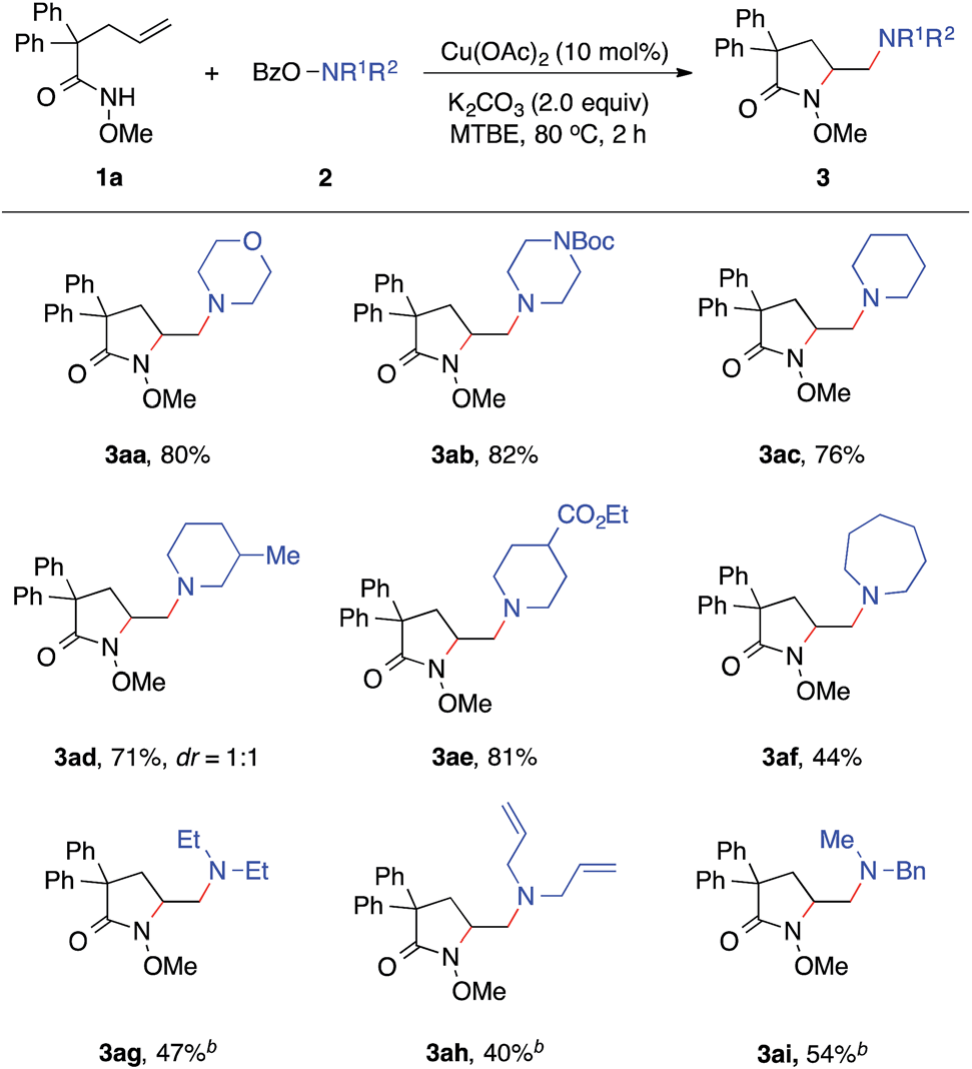

^*a*^Reaction conditions: **1a** (0.30 mmol, 1.0 equiv.), **2** (1.2 equiv.), Cu(OAc)_2_ (10 mol%), K_2_CO_3_ (2.0 equiv.), MTBE (1.5 mL), 80 °C, 2 h, unless otherwise noted.

^*b*^ **2** (2.0 equiv.), Cu(OAc)_2_ (20 mol%), 120 °C, 2 h.

We next examined the alkene scope of the diamination reaction using hydroxylamine **2a** (Table 3). Similar to **1a**, monosubstituted alkenes **1f–1j** bearing different substituents on the alkenyl chain all underwent smooth 5-*exo* cyclization and afforded the γ-substituted amino lactam products **3fa–3ja** in modest to high yields. In addition, disubstituted alkene **3k** also readily provided **3ka** in 62% yield. The formation of 6-membered lactam **3la** was also effective. Urea-based alkenes **1m–1n** proved to be viable substrates for the diamination reaction and successfully provided desired amino-imidazolidinones **3ma–3na**. It is important to note that this diamination reaction occurs with high diastereoselectivity, as observed in the formation of major products **3ja** and **3na**.

**Table 3:** Alkene scope of diamination^*a*^

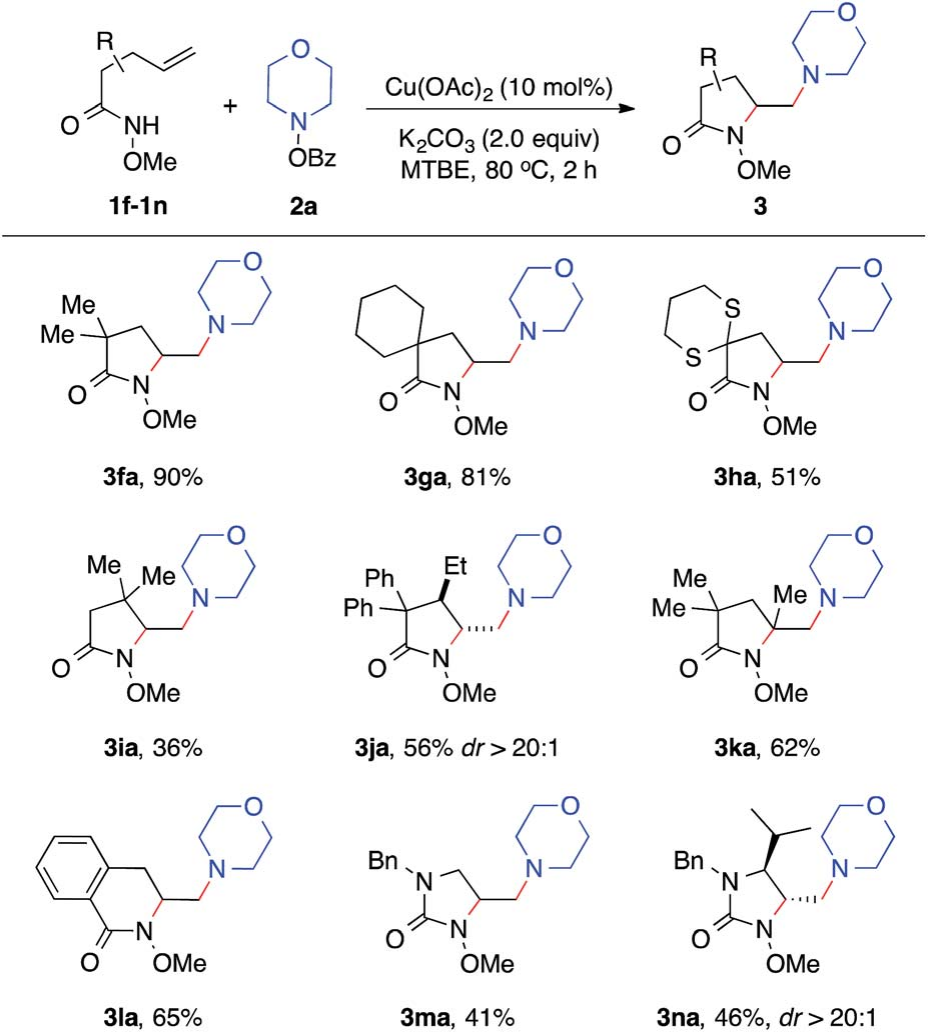

^*a*^Reaction conditions: **1a** (0.30 mmol, 1.0 equiv.), **2** (1.2 equiv.), Cu(OAc)_2_ (10 mol%), K_2_CO_3_ (2.0 equiv.), MTBE (1.5 mL), 80 °C, 2 h.

**Scheme 2:**
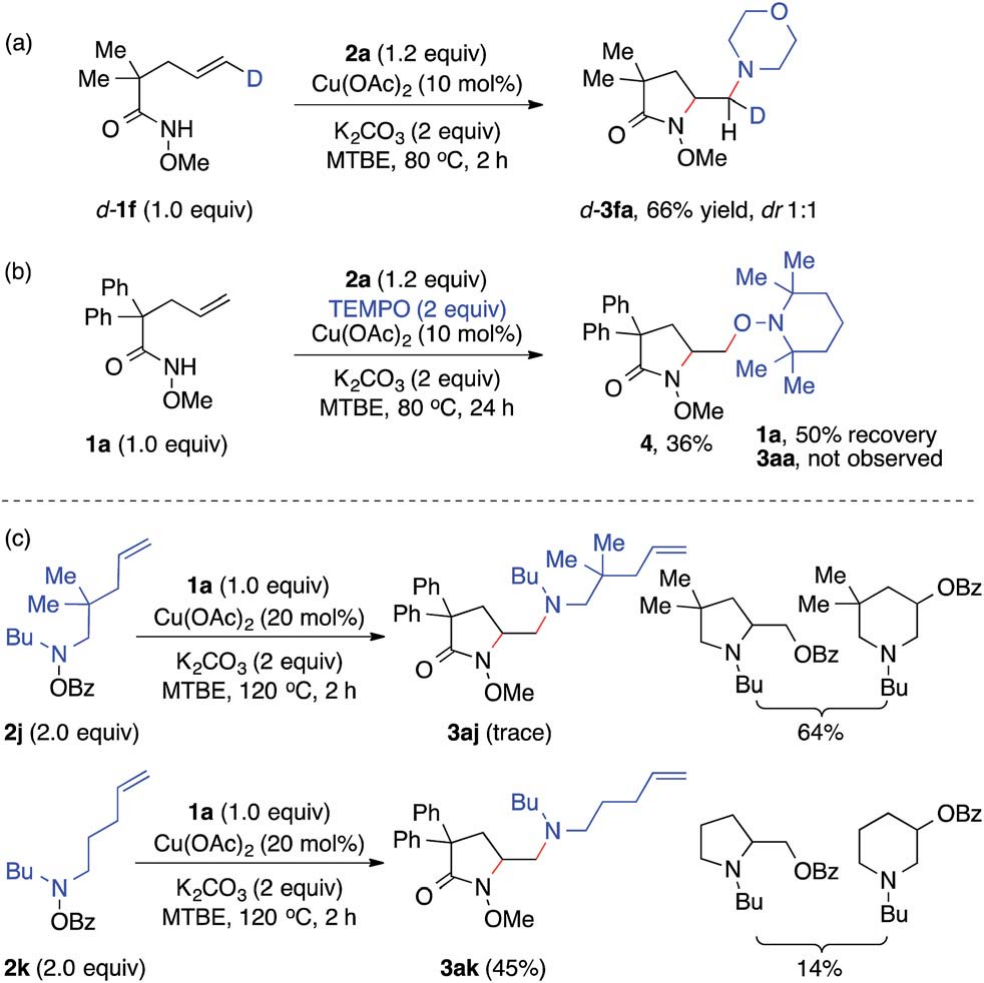
Mechanism investigations.

To study the nature of the intermolecular C–N bond formation step with hydroxylamines, the *trans-d*-substituted alkenyl amide *d-***1f** was subjected to the standard diamination reaction, giving a 1 : 1 mixture of *d*-substituted diaminated diastereomers *d-***3fa** in 66% yield (Scheme 2, **a**). The loss of stereochemistry implies the radical nature of the alkyl-Cu complex that results from the intramolecular aminocupration step.^16^ Next when the reaction of model substrates **1a** and **2a** was performed in the presence of a radical scavenger TEMPO (Scheme 2, **b**), aminooxygenation product **4** was isolated in 36% yield and no diamination product **3aa** was observed, indicating the radical intermediate was trapped by TEMPO.

To investigate the involvement of the hydroxylamine-derived amino radical in the diamination reaction, we examined the reactions of alkene **1a** using hydroxylamines **2j** and **2k**, both of which contain a tethered olefin for possible amino-radical-initiated cyclization (Scheme 2, **c**). The reaction with **2j** only provided a trace amount of desired diamination product **3aj** with a significant amount of aminooxygenation products.11*b* However, the reaction with **2k** was much more effective and offered diamination product **3ak** in 45% yield. These results suggested that amino radicals might be generated under the standard diamination conditions and the diamination reaction would be less competitive for those hydroxylamines that might undergo side reactions, such as facile aminocyclization (**2j**).

**Scheme 3:**
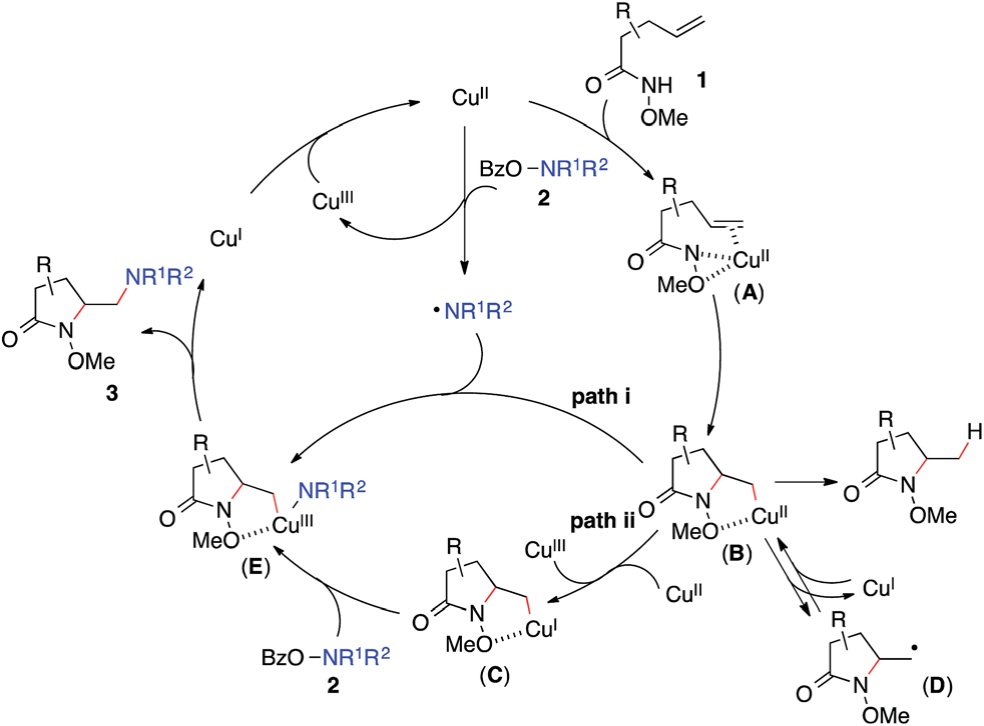
Proposed mechanism.

Based on these results, Scheme 3 outlines the possible pathways involved in this copper-catalyzed diamination reaction. First, the intramolecular aminocupration of alkene **1** will occur upon activation by a copper catalyst to form alkyl-Cu(ii) complex **B**.^17^ We propose that the intermediate **B** could associate with the amino radical, which is generated from *O*-benzoylhydroxylamine **2** in the presence of a copper catalyst, to form Cu(iii)–complex **E** (path i). Alternatively, the intermediate **B** may be reduced to Cu(i)–complex **C**, which can undergo a direct electrophilic amination with hydroxylamine **2** to form the same intermediate **E** (path ii). In either path, **E** would readily undergo reductive elimination, providing the diaminated product **3** and regenerating the Cu(ii)-catalyst.^18^ The alkoxyl group of the amide, which was found to be critical for the diamination reaction (Table 1, entries 5 and 12–15), would contribute to stabilizing the alkyl-copper intermediates **B** and **C**, therefore facilitating the intermolecular amination. Furthermore, radical intermediate **D** may form from the intermediate **B** upon a reversible C–Cu(ii) homolysis, thus abolishing the stereochemical control in the intermolecular amination step (Scheme 2, **a**).^17^ Besides the desired intermolecular amination, direct H-abstraction of **B** may occur to produce the hydroamination byproduct.

**Scheme 4:**
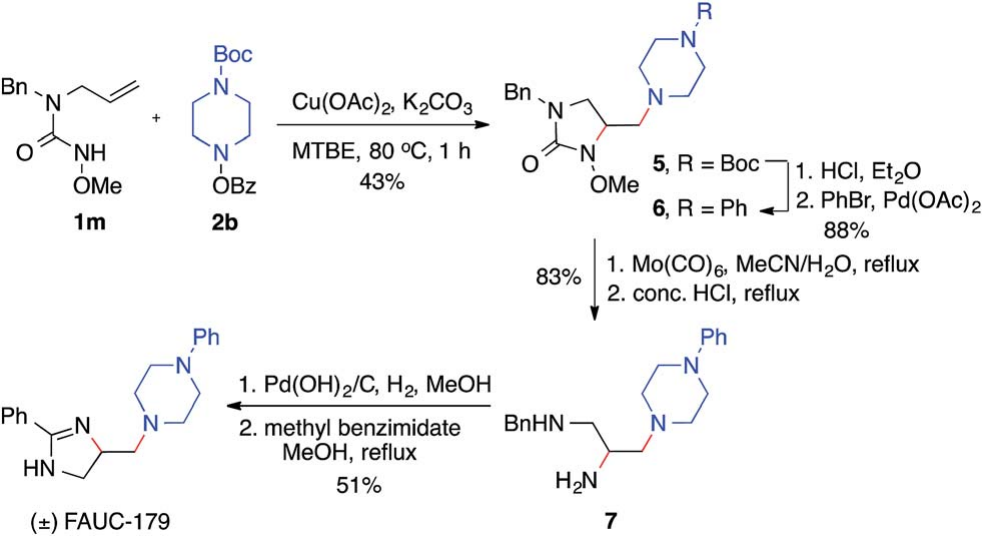
Synthesis of FAUC-179.

The utility of this diamination reaction for the synthesis of valuable diamine-containing agents was demonstrated by the rapid synthesis of FAUC-179, a selective dopamine D4 receptor partial agonist.^19^ As shown in Scheme 4, the preparation of FAUC-179 was readily achieved by using the diamination reaction of the simple alkene **1m**, followed by removal of protecting groups and subsequent functionalizations. It is noteworthy that the methoxy protecting group on the nitrogen necessary for the diamination reaction can be easily cleaved by Mo(CO)_6_. This example also highlights the applicability of this diamination method in the synthesis of 1,2,3-triamines with distinct substitutions (*e.g.***7**), which would be challenging for other diamination methods.

## Conclusions

In summary, a copper-catalyzed intramolecular diamination reaction of unactivated alkenes with *O*-benzoylhydroxylamines has been developed. It is the first metal-catalyzed alkene diamination that enables the direct incorporation of an electron-rich amino group. The method offers a rapid and efficient approach to construct diverse 1,2-diamine skeletons, including biologically and medicinally important γ-lactams and imidazolidinones. Further studies of the reaction mechanism and the development of an enantioselective diamination procedure are currently underway in our laboratory.

## Supplementary Material

Supplementary information
