# Supplementary material for: Copper-catalyzed diamination of unactivated alkenes with hydroxylamines
Source: Chem Sci. 2015 May 18;6(7):4279–83. doi: 10.1039/c5sc00897b (PMC5707484; doi:10.1039/c5sc00897b)
Supplement: Supplementary file 1 [file SC-006-C5SC00897B-s001.pdf]

Electronic Supplementary Material (ESI) for Chemical Science.
This journal is © The Royal Society of Chemistry 2015

# Copper-Catalyzed Diamination of Unactivated Alkenes with Hydroxylamines

Kun Shen and Qiu Wang*

Department of Chemistry, Duke University, Durham, North Carolina 27708

Email: qiu.wang@duke.edu

## Supporting Information

**Content**

| | |
|---|---|
| **I. General Procedures** | S1 |
| **II. Materials and Instrumentation** | S1 |
| **III. Supplementary Tables for Condition Optimization** | S2-3 |
| **IV. Substrate Synthesis** | S3-8 |
| **V. General Procedure for Diamination Reaction** | S8-14 |
| **VI. Mechanism Investigation** | S14-15 |
| **VII. Deprotection Conditions for 3aa** | S15-16 |
| **VIII. Synthesis of (±)-FAUC-179** | S16-17 |
| **IX. References** | S17 |
| **X. NMR Spectra** | S18-106 |

**I. General Procedures.** Glassware and stir bars were dried in an oven at 140 °C for at least 12 h and then cooled in a desiccator cabinet over Drierite prior to use. Optimization and substrate screens were performed in 8-mL microwave vials. Vials were fitted with crimp top septa under a positive pressure of nitrogen that had been passed through a column (5 x 20 cm) of Drierite, unless otherwise noted. All other reactions were performed in round-bottom flasks sealed with rubber septa. Plastic syringes or glass pipets were used to transfer liquid reagents. Reactions were stirred magnetically using Teflon-coated, magnetic stir bars. Analytical thin-layer chromatography (TLC) was performed using aluminum plates pre-coated with 0.25 mm of 230–400 mesh silica gel impregnated with a fluorescent indicator (254 nm). TLC plates were visualized by exposure to ultraviolet light and/or exposure to $KMnO_4$ stain. Organic solutions were concentrated under reduced pressure using a rotary evaporator. Flash-column chromatography was performed on silica gel (60 Å, standard grade) or with pre-packed FLASH silica gel columns.

**II. Materials and Instrumentation.** Nuclear magnetic resonance spectra were recorded at ambient temperature (unless otherwise stated) on 400 MHz or 500 MHz spectrometers. All values for proton chemical shifts are reported in parts per million (δ) and are referenced to the residual protium in $CDCl_3$ (δ 7.26). All values for carbon chemical shifts are reported in parts per million (δ) and are referenced to the carbon resonances in $CDCl_3$ (δ 77.0). NMR data are represented as follows: chemical shift, multiplicity (s = singlet, d = doublet, t = triplet, q = quartet, quin = quintet, m = multiplet, br = broad), coupling constant (Hz), and integration. Infrared spectroscopic data are reported in wavenumbers ($cm^{-1}$). High-resolution mass spectra were obtained using a liquid chromatography-electrospray ionization and Time-of-flight mass spectrometer.

## III. Supplementary Tables for Condition Optimization

**a. Condition optimizations for the diamination of alkene 1a and hydroxylmorpholine 2a**[a]

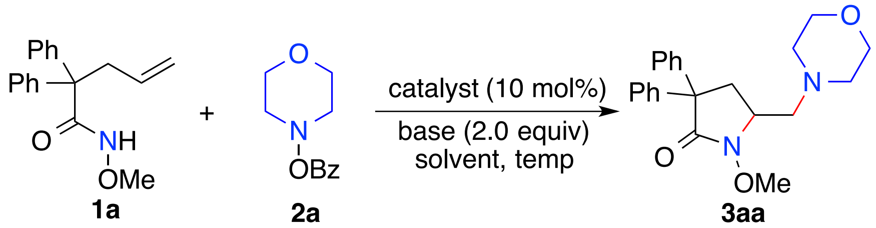

| entry | catalyst | base | solvent | temp (°C) | yield (%)[b] |
|---|---|---|---|---|---|
| 1 | $Cu(OAc)_2$ | $K_2CO_3$ | toluene | 80 | 68 |
| 2 | $Cu(OAc)_2$ | $K_2CO_3$ | THF | 80 | 74 |
| 3 | $Cu(OAc)_2$ | $K_2CO_3$ | DCE | 80 | 48 |
| 4 | $Cu(OAc)_2$ | $K_2CO_3$ | $CH_3CN$ | 80 | 70 |
| **5** | **$Cu(OAc)_2$** | $K_2CO_3$ | MTBE | 80 | **84 (80)**[c] |
| 6 | $Cu(OAc)_2$ | $K_2CO_3$ | DMF | 80 | trace |
| 7 | $Cu(CF_3COO)_2$ | $K_2CO_3$ | MTBE | 80 | 60 |
| 8 | $Cu(OTf)_2$ | $K_2CO_3$ | MTBE | 80 | 46 |
| 9 | $Cu(acac)_2$ | $K_2CO_3$ | MTBE | 80 | 24 |
| 10 | $CuCl_2$ | $K_2CO_3$ | MTBE | 80 | 39 |
| 11 | $CuF_2$ | $K_2CO_3$ | MTBE | 80 | trace |
| 12 | CuOAc | $K_2CO_3$ | MTBE | 80 | 60 |
| 13 | - | $K_2CO_3$ | MTBE | 80 | 0 |
| 14 | $Cu(OAc)_2$ | - | MTBE | 80 | 60 |
| 15 | $Cu(OAc)_2$ | $Na_2CO_3$ | MTBE | 80 | 58 |
| 16 | $Cu(OAc)_2$ | $Cs_2CO_3$ | MTBE | 80 | 0 |
| 17[d] | $Cu(OAc)_2$ | $K_2CO_3$ | MTBE | 40 | 0 |
| 18[d] | $Cu(OAc)_2$ | $K_2CO_3$ | MTBE | 60 | 44 |
| 19 | $Cu(OAc)_2$ | $K_2CO_3$ | MTBE | 100 | 74 |

[a]Reaction Conditions: The reactions were performed in a sealed tube with **1a** (0.20 mmol, 1.0 equiv), **2a** (0.24 mmol, 1.2 equiv), catalyst (0.02 mmol, 10 mol%), base (0.40 mmol, 2.0 equiv) and solvent (1 mL) for 2 h, unless otherwise noted. [b]The yield was determined by $^1H$ NMR with diboromethane as an internal standard. [c]The number in the parathesis was the isolation yield. [d]The reaction was run for 4 h.

**b. Condition optimization for the diamination reaction of 1a and *N,N*-diethyl-*O*-benzoyl hydroxylamine 2g**[a]

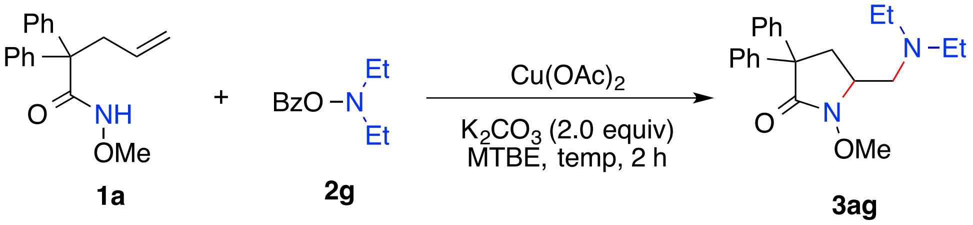


| entry | $Cu(OAc)_2$ (mol%) | **2g** (equiv) | temp (°C) | yield (%)[b] |
|---|---|---|---|---|
| 1 | 10 | 1.2 | 80 | 22 |
| 2 | 20 | 1.2 | 80 | 24 |
| 3 | 40 | 1.2 | 80 | 23 |
| 4 | 10 | 1.2 | 120 | 36 |
| 5 | 20 | 1.2 | 120 | 41 |
| 6 | 40 | 1.2 | 120 | 37 |
| 7 | 20 | 2.0 | 80 | 31 |
| 8 | **20** | **2.0** | **120** | **54** |

[a]Reaction were performed in a 0.1 mmol scale. [b]The yield was determined by $^1$H NMR with diboromethane as an internal standard.

## IV. Synthesis of Substrates.

### *N*-Methoxy-2,2-diphenylpent-4-enamide (1a).

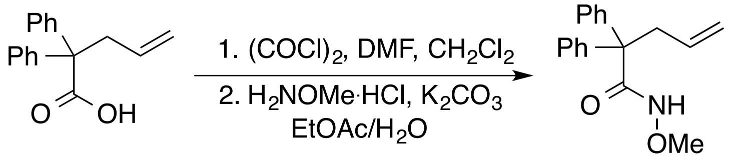


To a solution of 2,2-diphenylpent-4-enoic acid (5.04 g, 20 mmol) in $CH_2Cl_2$ (20 mL) was added dropwise oxalyl chloride (2.2 mL, 26 mmol) followed by a catalytic amount of DMF. The mixture was stirred at room temperature for 1 h, and then was concentrated under reduced pressure to remove the solvent. The resulting residue was added dropwise to a biphasic mixture of $MeONH_2$·HCl (2.51 g, 30 mmol) and $K_2CO_3$ (5.52 g, 40 mmol) in EtOAc (36 mL) and $H_2O$ (18 mL). The reaction was stirred at room temperature for 2 h. The organic layer was separated and the aqueous layer was extracted with EtOAc. The combined extracts were washed with brine, dried over $Na_2SO_4$, and filtrated. The filtrate was concentrated. Purification by column chromatography (33% EtOAc in hexanes) gave **1a** as a white solid (5.0 g, 89% yield); $R_f$= 0.55 (50% EtOAc in EtOAc in hexanes); $^1$H NMR (400 MHz, $CDCl_3$): δ 8.16 (s, 1H), 7.45–7.15 (m, 10H), 5.85–5.65 (m, 1H), 4.99 (d, $J$ = 17.6 Hz, 1H), 4.95 (d, $J$ = 11.6 Hz, 1H), 3.66 (s, 3H), 3.20 (d, $J$ = 6.8 Hz, 2H); $^{13}$C NMR (100 MHz, $CDCl_3$): δ 171.9, 141.9, 134.5, 128.7, 128.3, 127.1, 118.2, 64.0, 58.9, 43.2; IR (neat): 3272, 1650, 1488, 1442, 906, 733, 699 $cm^{-1}$; HRMS (m/z) Calcd for ($C_{18}H_{20}NO_2$) ($[M+H]^+$): 282.1489; found: 282.1486.

### *N*-(Benzyloxy)-2,2-diphenylpent-4-enamide (1b).

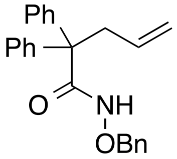

Follow the same procedure with **1a**. Purification by column chromatography (33% EtOAc in hexanes) gave **1b** as a yellow solid (91% yield); $R_f$= 0.42 (20% EtOAc in hexanes); $^1$H NMR (400 MHz, $CDCl_3$): δ 8.12 (s,

1H), 7.38–7.18 (m, 15H), 5.85–5.65 (m, 1H), 4.99 (d, $J$ = 11.6 Hz, 1H), 4.97–4.92 (m, 1H), 4.85 (s, 2H), 3.22 (d, $J$ = 6.8 Hz, 2H); $^{13}$C NMR (100 MHz, $CDCl_3$): δ 171.3, 141.8, 134.8, 134.4, 129.1, 128.6, 128.4, 128.3, 128.1, 126.9, 118.0, 77.7, 58.9, 43.0; IR (neat): 3261, 3063, 3033, 1646, 1491, 1464, 1441, 917, 745, 691 cm$^{-1}$; HRMS (m/z) Calcd for ($C_{24}H_{24}NO_2$) ($[M+H]^+$): 358.1802; found: 358.1802.

***N*-Benzyl-2,2-diphenylpent-4-enamide (1d).**

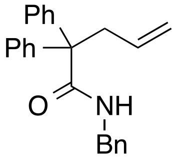


Follow the same procedure with **1a**. Purification by column chromatography (15% EtOAc in hexanes) gave **1d** as a yellow solid (88% yield); $R_f$= 0.54 (20% EtOAc in hexanes); $^1$H NMR (400 MHz, $CDCl_3$): δ 7.34–7.28 (m, 8H), 7.28–7.20 (m, 5H), 7.10–7.03 (m, 2H), 5.86 (s, br, 1H), 5.81–5.69 (m, 1H), 5.02–4.89 (m, 2H), 4.44 (d, $J$ = 5.6 Hz, 2H), 3.25 (d, $J$ = 6.8 Hz, 2H); $^{13}$C NMR (100 MHz, $CDCl_3$): δ 173.9, 142.8, 138.1, 135.1, 129.0, 128.5, 128.2, 127.3, 127.2, 126.9, 117.8, 60.6, 43.7, 43.3; IR (neat): 3332, 3060, 3026, 1643, 1524, 1247, 733, 694 cm$^{-1}$; HRMS (m/z) Calcd for ($C_{24}H_{24}NO$) ($[M+H]^+$): 342.1852; found: 342.1853.

***N*-Methoxy-2,2-dimethylpent-4-enamide (1f).**

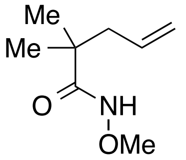


Follow the same procedure with **1a**. Purification by column chromatography (25% EtOAc in hexanes) gave **1f** as a yellow oil (55% yield); $R_f$= 0.31 (50% EtOAc in hexanes); $^1$H NMR (400 MHz, $CDCl_3$): δ 9.72 (s, 1H), 5.71–5.55 (m, 1H), 4.98–4.88 (m, 2H), 3.60 (s, 3H), 2.17 (d, $J$ = 7.6 Hz, 2H), 1.06 (s, 3H); $^{13}$C NMR (100 MHz, $CDCl_3$): δ 174.8, 133.7, 117.8, 63.4, 44.6, 40.8, 24.3; IR (neat): 3212, 2970, 2934, 1640, 1498, 1473, 1048, 908, 575 cm$^{-1}$; HRMS (m/z) Calcd for ($C_8H_{16}NO_2$) ($[M+H]^+$): 158.1176; found: 158.1176.

**1-Allyl-*N*-methoxycyclohexane-1-carboxamide (1g).**

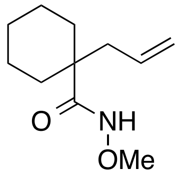


Follow the same procedure with **1a**. Purification by column chromatography (25% EtOAc in hexanes) gave **1g** as a yellow oil (54% yield); $R_f$= 0.48 (50% EtOAc in hexanes); $^1$H NMR (400 MHz, $CDCl_3$): δ 9.61 (s, 1H), 5.70–5.56 (m, 1H), 4.92 (d, $J$ = 16.0 Hz, 1H), 4.90 (d, $J$ = 11.2 Hz, 1H), 3.61 (s, 3H), 2.14 (d, $J$ = 7.2 Hz, 2H), 1.93–1.79 (m, 2H), 1.51–1.09 (m, 8H); $^{13}$C NMR (100 MHz, $CDCl_3$): δ 173.3, 133.3, 117.6, 63.5, 45.1, 43.9, 33.1, 25.5, 22.4; IR (neat): 3142, 2925, 1636, 1520, 1435, 1047, 901, 619 cm$^{-1}$; HRMS (m/z) Calcd for ($C_{11}H_{20}NO_2$) ($[M+H]^+$): 198.1489; found: 198.1488.

**2-Allyl-*N*-methoxy-1,3-dithiane-2-carboxamide (1h).**

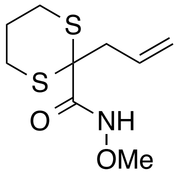


Follow the same procedure with **1a**. Purification by column chromatography (50% EtOAc in hexanes) gave **1h** as a white solid (47% yield); $R_f$= 0.17 (50% EtOAc in hexanes); $^1$H NMR (400 MHz, $CDCl_3$): δ 9.73 (s, 1H), 5.86–5.72 (m, 1H), 5.22–5.12 (m, 2H), 3.78 (s, 3H), 2.96 (dd, $J$ = 12.4, 2.8 Hz, 1H), 2.92 (dd, $J$ = 12.4, 2.8 Hz, 1H), 2.72 (dd, $J$ = 4.4, 3.2 Hz, 1H), 2.69 (dd, $J$ = 4.4, 3.2 Hz, 1H), 2.66 (dt, $J$ = 7.6, 0.8 Hz, 2H), 2.14–2.03 (m, 1H), 1.92–1.77 (m, 1H); $^{13}$C NMR (100 MHz, $CDCl_3$): δ 166.5, 129.9, 119.6, 63.7, 56.0, 44.2,

27.9, 23.7; IR (neat): 3273, 2932, 2905, 1661, 1481, 1415, 1045, 992, 926, 730, 661, 534 cm$^{-1}$; HRMS (m/z) Calcd for ($C_9H_{16}NO_2S_2$) ($[M+H]^+$): 234.0617; found: 234.0617.

***N*-Methoxy-2,2,4-trimethylpent-4-enamide (1k).**

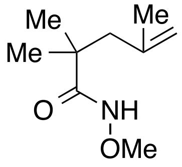

Follow the same procedure with **1a**. Purification by column chromatography (33% EtOAc in hexanes) gave **1k** as a yellow oil (67% yield); $R_f$= 0.31 (50% EtOAc in hexanes); $^1$H NMR (400 MHz, $CDCl_3$): δ 8.59 (s, 1H), 4.82–4.79 (m, 1H), 4.68–4.64 (m, 1H), 3.71 (s, 3H), 2.27 (s, 2H), 1.67 (s, 3H), 1.15 (s, 6H); $^{13}$C NMR (100 MHz, $CDCl_3$): δ 175.4, 142.3, 114.6, 64.0, 48.3, 41.1, 25.4, 23.8; IR (neat): 3212, 2967, 2934, 1642, 1497, 1472, 1052, 890, 585, 539 cm$^{-1}$; HRMS (m/z) Calcd for ($C_9H_{18}NO_2$) ($[M+H]^+$): 172.1332; found: 172.1332.

**2-Allyl-*N*-methoxybenzamide (1l).**

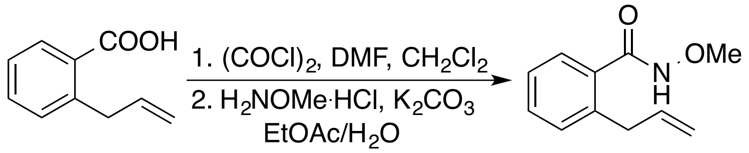


Follow the same procedure with **1a**. 2-Allylbenzoic acid was synthesized according to literature procedure[1]. Purification by column chromatography (33% EtOAc in hexanes) gave **1l** as a white solid (45% yield); $R_f$= 0.38 (50% EtOAc in hexanes); $^1$H NMR (400 MHz, $CDCl_3$): δ 9.53 (s, 1H), 7.37 (t, *J* = 7.6 Hz, 1H), 7.30 (d, *J* = 7.6 Hz, 1H), 7.23 (d, *J* = 7.6 Hz, 1H), 7.18 (t, *J* = 7.6 Hz, 1H), 6.00–5.87 (m, 1H), 5.10–4.95 (m, 2H), 3.77 (s, 3H), 3.49 (d, *J* = 6.4 Hz, 2H); $^{13}$C NMR (100 MHz, $CDCl_3$): δ 167.4, 138.4, 137.0, 132.5, 130.4, 130.2, 127.5, 126.0, 116.0, 64.0, 37.0; IR (neat): 3123, 2935, 2820, 1633, 1529, 1316, 1039, 902, 746 cm$^{-1}$; HRMS (m/z) Calcd for ($C_{11}H_{14}NO_2$) ($[M+H]^+$): 192.1019; found: 192.1019.

***N*-Methoxy-3,3-dimethylpent-4-enamide (1i).**

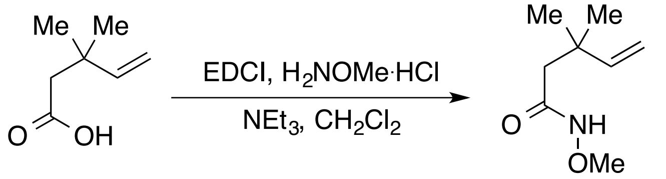


To a solution of 3,3-dimethylpent-4-enoic acid (1.28 g, 10 mmol) in $CH_2Cl_2$ (10 mL), was added sequentially triethylamine (2.2 mL, 16 mmol), EDCI (3.44 g, 18 mmol) and $MeONH_2$·HCl (1.33 g, 16 mmol). The resulting mixture was stirred at room temperature overnight and then was filtered through a pale of Celite. The filtrate was concentrated. Purification by column chromatography (33% EtOAc in hexanes) gave **1i** as a colorless oil (68% yield); $R_f$= 0.28 (50% EtOAc in hexanes); $^1$H NMR (400 MHz, $CDCl_3$): δ 9.21 (s, br, 1H), 5.85 (dd, *J* = 17.6, 10.8 Hz, 1H), 4.95 (d, *J* = 17.6 Hz, 1H), 4.93 (d, *J* = 10.0 Hz, 1H), 3.68 (s, 3H), 2.07 (s, 2H), 1.10 (s, 6H); $^{13}$C NMR (100 MHz, $CDCl_3$): δ 168.9, 146.9, 111.2, 64.0, 45.3, 36.2, 26.8; IR (neat): 3170, 2962, 1649, 1515, 1463, 1438, 1364, 1068, 910, 724, 675, 590 cm$^{-1}$; HRMS (m/z) Calcd for ($C_8H_{16}NO_2$) ($[M+H]^+$): 158.1176; found: 158.1176.

**3-Ethyl-*N*-methoxy-2,2-diphenylpent-4-enamide (1j).**

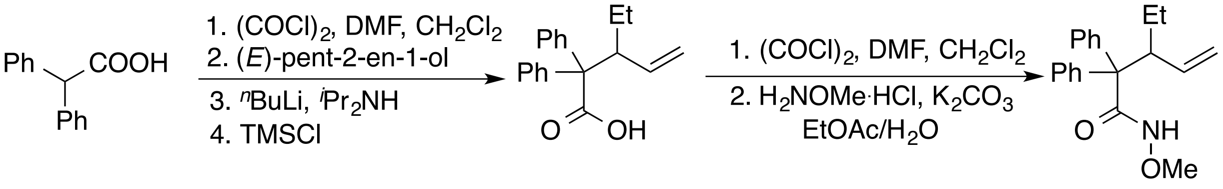


To a solution of 2,2-diphenylacetic acid (2.12 g, 10 mmol) in $CH_2Cl_2$ (10 mL) was added dropwise oxalyl chloride (0.93 mL, 11 mmol) followed by a catalytic amount of DMF. The mixture was stirred at room

temperature for 1 h, and then was added dropwise pyridine (1.76 mL, 22 mmol) and (*E*)-pent-2-en-1-ol (1.22 mL, 12 mmol). The resulting mixture was stirred ar room temperature overnight and then was quenched by the addition of a saturated aqueous solution of $Na_2CO_3$. The organic layer was separated and the aqueous layer was extracted with $CH_2Cl_2$. The combined organic layers were washed with brine, dried over $Na_2SO_4$, and filtrated. The filtrate was concentrated, providing the crude ester product which was directly used for the next step. To the solution of the crude ester product in THF (10 mL), was added dropwise at –78 °C the freshly prepared LDA solution (20 mmol, 2 equiv) in THF (15 mL). The reaction mixture was allowed to stir at –78 °C for 1 h and then was added TMSCl (2.7 mL, 21 mmol). The reaction mixture was slowly warmed to room temperature and then stirred at 60 °C overnight. After cooling down to room retemperature, the reaction was quenched with an aqueous solution of HCl (2 M). The organic layer was separated and the aqueous layer was extracted with EtOAc. The combined extracts were washed with brine, dried over $Na_2SO_4$, and filtrated. The filtrate was concentrated. Purification by column chromatography (10% EtOAc in hexanes) gave 3-ethyl-2,2-diphenylpent-4-enoic acid as a white solid (1.67 g, 60% yield); $R_f$= 0.44 (20% EtOAc in hexanes); $^1H$ NMR (400 MHz, $CDCl_3$): δ 7.40–7.16 (m, 10H); 5.34–5.06 (m, 3H), 3.41 (t, *J* = 8.8 Hz, 1H), 1.71–1.54 (m, 1H), 0.89 (t, *J* = 7.2 Hz, 3H), 0.64–0.46 (m, 1H); $^{13}C$ NMR (100 MHz, $CDCl_3$): δ 179.6, 137.6, 130.9 (2C), 130.1 (2C), 127.6 (2C), 127.03 (2C), 127.00 (2C), 126.8 (2C), 119.0, 64.9, 48.8, 23.5, 12.0; IR (neat): 2935, 2872, 1695, 1264, 1249, 722, 709 $cm^{-1}$; HRMS (m/z) Calcd for ($C_{19}H_{20}O_2$) ($[M+H]^+$): 281.1536; found: 281.1536.

To a solution of 3-ethyl-2,2-diphenylpent-4-enoic acid (1.04 g, 3.7 mmol) in $CH_2Cl_2$ (5 mL) was added dropwise oxalyl chloride (0.42 mL, 4.9 mmol) followed by a catalytic amount of DMF. The mixture was stirred at room temperature for 1 h, and then was concentrated under reduced pressure. The residue was added dropwise to a biphasic mixture of $MeONH_2·HCl$ (464 mg, 5.6 mmol) and $K_2CO_3$ (1.02 g, 7.4 mmol) in EtOAc (20 mL) and $H_2O$ (10 mL). The reaction mixture was stirred at room temperature for 2 h. Then the organic layer was separated and the aqueous layer was extracted with EtOAc. The combined extracts were washed with brine, dried over $Na_2SO_4$, and filtrated. The filtrate was concentrated. Purification by column chromatography (33% EtOAc in hexanes) gave **1j** as a yellow oil (520 mg, 46% yield); $R_f$= 0.59 (50% EtOAc in hexanes); $^1H$ NMR (400 MHz, $CDCl_3$): δ 8.37 (s, 1H), 7.33 (t, *J* = 7.2 Hz, 4H), 7.30–7.18 (m, 6H), 5.48–5.36 (m, 1H), 5.13–5.03 (m, 2H), 3.49 (s, 3H), 3.38 (t, *J* = 9.6 Hz, 1H), 1.75–1.62 (m, 1H), 0.89 (t, *J* = 7.2 Hz, 3H), 0.79–0.65 (m, 1H); $^{13}C$ NMR (100 MHz, $CDCl_3$): δ 171.2, 140.6, 140.2, 137.6, 130.0, 129.5, 127.7, 127.6, 126.9, 126.8, 118.3, 63.5, 63.4, 50.0, 23.6, 12.0; IR (neat): 3246, 2962, 1654, 1494, 1441, 909, 717, 696 $cm^{-1}$; HRMS (m/z) Calcd for ($C_{20}H_{24}NO_2$) ($[M+H]^+$): 310.1802; found: 310.1802.

**2,2-Diphenyl-*N*-tosylpent-4-enamide (1c).**

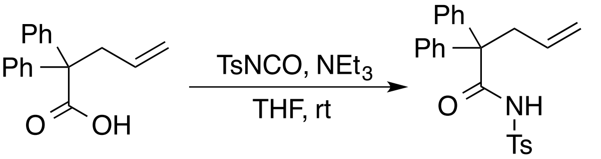


To a solution of 2,2-diphenylpent-4-enoic acid (1.26 g, 5 mmol) in THF (20 mL) was added *p*-tosyl isocyanate (985 mg, 5 mmol), followed by the dropwise addition of triethyl amine (0.7 mL, 5 mmol) with release of gas. The mixture was stirred at room temperature for 3 h, and then was diluted with EtOAc. The organic layer was sequentially washed with an aqueous solution of HCl (2 M), a saturated aqueous solution of $NaHCO_3$ and brine, dried over $Na_2SO_4$, and filtered. The filtrate was concentrated. Purification by column chromatography (20% EtOAc in hexanes) gave **1c** as a white solid (1.19 g, 59% yield); $R_f$= 0.46 (20% EtOAc in hexanes); $^1H$ NMR (400 MHz, $CDCl_3$): δ 7.89 (s, 1H), 7.72 (d, *J* = 8.4 Hz, 2H), 7.34–7.22 (m, 8H), 7.17–7.08 (m, 4H), 5.58–5.43 (m, 1H), 4.90–4.79 (m, 2H), 3.04 (d, *J* = 6.8 Hz, 2H), 2.43 (s, 3H); $^{13}C$ NMR (100 MHz, $CDCl_3$): δ 171.3, 144.9, 140.1, 134.9, 133.3, 129.3, 128.7, 128.6, 128.4, 127.6, 119.0, 61.4, 42.6, 21.6; IR (neat): 3273, 3067, 3026, 1718, 1698, 1398, 1341, 1305, 698, 659, 550 $cm^{-1}$; HRMS (m/z) Calcd for ($C_{24}H_{24}NO_3S$) ($[M+H]^+$): 406.1471; found: 406.1471.

**1-Allyl-1-benzyl-3-methoxyurea (1m).**

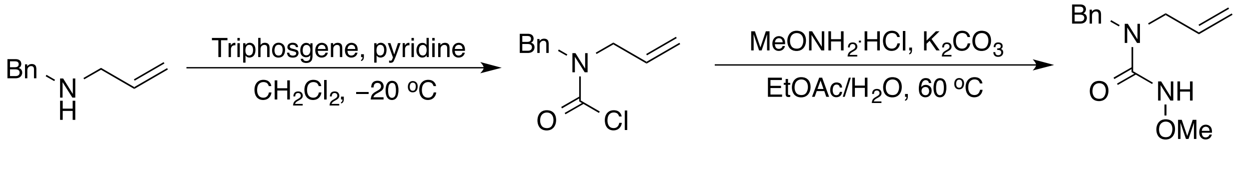

To a solution of triphosgene (7.8 g, 26 mmol) in anhydrous $CH_2Cl_2$ (50 mL) at –20 °C, was added dropwise pyridine (5.2 mL, 52 mmol), followed by a solution of *N*-benzylprop-2-en-1-amine (2.94 g, 20 mmol) in $CH_2Cl_2$ (10 mL). The reaction mixture was stirred at room temperature for 12 h and then was quenched with a saturated aqueous solution of $NH_4Cl$. The organic layer was separated and the aqueous layer was extracted with $CH_2Cl_2$. The combined extracts were washed with brine, dried over $Na_2SO_4$, and filtrated. The filtrate was concentrated, providing the crude carbamoyl chloride product that was directly used for the next step. The crude carbamoyl chloride product was added dropwise to a biphasic mixture of $MeONH_2$·HCl (2.5 g, 30 mmol) and $K_2CO_3$ (5.52 g, 40 mmol) in EtOAc (30 mL) and $H_2O$ (15 mL). The reaction mixture was stirred at 60 °C overnight and then the organic layer was separated. The aqueous layer was extracted with EtOAc. The combined organic layers were washed with brine, dried over $Na_2SO_4$, and filtrated. The filtrate was concentrated. Purification by column chromatography (50% EtOAc in hexanes) gave **1m** as a yellow solid (2.97 g, 68% yield); $R_f$= 0.33 (50% EtOAc in hexanes); $^1$H NMR (400 MHz, $CDCl_3$): δ 7.51 (s, 1H), 7.31–7.15 (m, 5H), 5.74–5.62 (m, 1H), 5.17–5.07 (m, 2H), 4.11 (s, 2H), 3.74 (d, *J* = 5.6 Hz, 2H), 3.64 (s, 3H); $^{13}$C NMR (100 MHz, $CDCl_3$): δ 159.4, 136.9, 132.8, 128.5, 127.4 (2C), 117.2, 64.0, 49.6, 48.6; IR (neat): 3247, 2992, 2962, 2929, 2890, 1650, 1479, 1250, 943, 693 cm$^{-1}$; HRMS (m/z) Calcd for ($C_{12}H_{17}N_2O_2$) ($[M+H]^+$): 221.1285; found: 221.1285.

**(*S*)-1-Benzyl-3-methoxy-1-(4-methylpent-1-en-3-yl) (1n).**

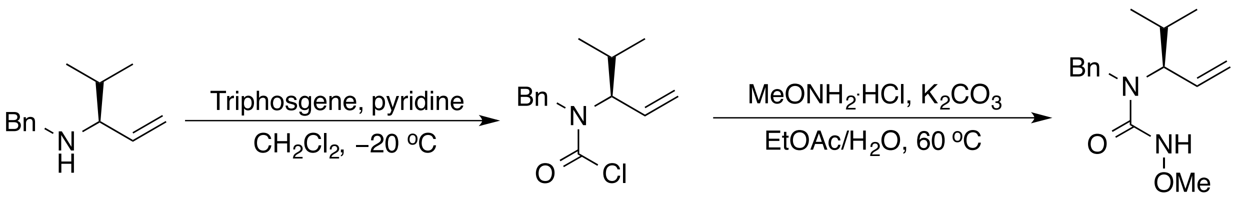


To a solution of triphosgene (1.55 g, 5.2 mmol) in anhydrous $CH_2Cl_2$ (10 mL) at –20 °C, was added dropwise pyridine (1 mL, 10 mmol) followed by a solution of (*S*)-*N*-benzyl-4-methylpent-1-en-3-amine[2] (756 mg, 4 mmol) in $CH_2Cl_2$ (2 mL). The reaction mixture was stirred at room temperature for 9 h, and then was quenched with a saturated aqueous solution of $NH_4Cl$. The organic layer was separated and the aqueous layer was extracted with $CH_2Cl_2$. The combined extracts were washed with brine, dried over $Na_2SO_4$, and filtrated. The filtrate was concentrated, providing the crude carbamoyl chloride product that was directly used for the next step. The crude carbamoyl chloride product was added dropwise to a biphasic mixture of $MeONH_2$·HCl (501 mg, 6 mmol) and $K_2CO_3$ (1.1 g, 8 mmol) in EtOAc (20 mL) and $H_2O$ (10 mL). The reaction mixture was stirred at 60 °C for 14 h and then the organic layer was separated. The aqueous layer was extracted with EtOAc. The combined organic layers were washed with brine, dried over $Na_2SO_4$, and filtrated. The filtrate was concentrated. Purification by column chromatography (20% EtOAc in hexanes) gave **1n** as a white solid (438 mg, 42% yield); $[\alpha]^{20}_D$ = +9.0 (c = 1.0, $CHCl_3$); $R_f$= 0.52 (50% EtOAc in hexanes); $^1$H NMR (400 MHz, $CDCl_3$): δ 7.35–7.20 (m, 5H), 6.96 (s, 1H), 5.75 (ddd, *J* = 17.2, 10.0, 8.4 Hz, 1H), 5.22 (dd, *J* = 17.2, 0.8 Hz, 1H), 5.17 (dt, *J* = 10.0, 0.8 Hz, 1H), 4.36 (d, *J* = 16.8 Hz, 1H), 4.27 (d, *J* = 16.8 Hz, 1H), 4.22 (t, *J* = 9.6 Hz, 1H), 3.56 (s, 3H), 2.04–1.90 (m, 1H), 0.92 (d, *J* = 6.8 Hz, 3H), 0.89 (d, *J* = 6.8 Hz, 3H); $^{13}$C NMR (100 MHz, $CDCl_3$): δ 159.5, 137.1, 135.7, 128.9, 127.7, 126.7, 118.9, 65.8, 64.0, 47.4, 29.8, 20.1, 19.5; IR (neat): 3205, 2958, 2873, 1728, 1637, 1475, 927, 717, 578 cm$^{-1}$; HRMS (m/z) Calcd for ($C_{15}H_{23}N_2O_2$) ($[M+H]^+$): 263.1754; found: 263.1754.

**(*E*)-2,2-dimethylpent-4-enoic-5-*d* acid.**

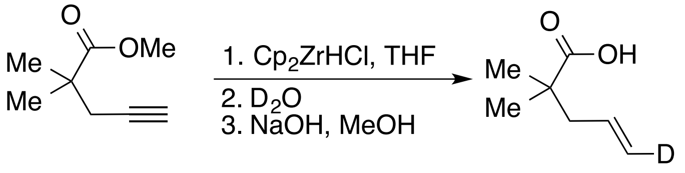


To a suspension of $Cp_2ZrHCl$ (2.55 g, 9.9 mmol) in THF (16 mL) at room temperature under nitrogen, was added dropwise a solution of methyl 2,2-dimethylpent-4-ynoate[3] in THF (2 mL). The reaction was stirred for 2 h and was added $D_2O$ (1 mL). After 24 h, the mixture was concentrated under reduced pressure. To the resulting crude, were added NaOH (1.5 g, 37.5 mmol), MeOH (10 mL) and $H_2O$ (5 mL) and the resulting reaction mixture was stirred at 60 °C for 5 h. After cooling down to room temperature, the reaction mixture was concentrated and then diluted with $H_2O$ (15 mL). The aqueous mixture was washed with EtOAc. The aqueous layer was acidified with a concentrated aqueous solution of HCl and extracted with EtOAc. The

organic extracts were washed with brine, dried over $Na_2SO_4$, and filtrated. The filtrate was concentrated. The crude residue was purified by column chromatography (2% ethyl acetate in hexanes) to give the (*E*)-2,2-dimethylpent-4-enoic-5-*d* acid as a yellow oil (550 mg, 48%). $R_f$= 0.58 (20% EtOAc in hexanes); $^1$H NMR (400 MHz, $CDCl_3$): δ 11.2 (br, 1H), 5.74 (dt, *J* = 17.2, 7.2 Hz, 1H), 5.03 (dt, *J* = 17.2, 1.2 Hz, 1H), 2.27 (dd, *J* = 17.2, 1.2 Hz, 2H), 1.17 (s, 6H); $^{13}$C NMR (100 MHz, $CDCl_3$): δ 184.5, 133.7, 117.9 (t, *J* = 25 Hz), 44.3, 42.1, 24.5; IR (neat): 2972, 2932, 2910, 2875, 1696, 1473, 1283, 1221, 1160, 982, 940, 873, 830, 552 cm$^{-1}$; HRMS (m/z) Calcd for ($C_5H_{10}DO_2$) ([M-H]$^-$): 128.0829; found: 128.0829.

**(*E*)-*N*-Methoxy-2,2-dimethylpent-4-en-5-*d*-amide (*d*-1f).**

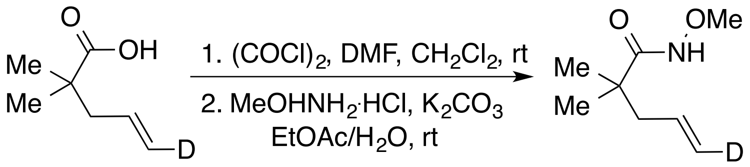


To a solution of (*E*)-2,2-dimethylpent-4-enoic-5-*d* acid (387 mg, 3 mmol) in $CH_2Cl_2$ (5 mL), was added dropwise oxalyl chloride (0.33 mL, 3.9 mmol) followed by a catalytic amount of DMF. The mixture was stirred at room temperature for 1 h and then was added dropwise to a biphasic mixture of $MeONH_2$·HCl (376 mg, 4.5 mmol) and $K_2CO_3$ (828 mg, 6 mmol) in EtOAc (10 mL) and $H_2O$ (5 mL). The reaction mixture was stirred at room temperature for 2 h. Then the organic layer was separated and the aqueous layer was extracted with EtOAc. The combined extracts were washed with brine, dried over $Na_2SO_4$, and filtrated. The filtrate was concentrated. Purification by column chromatography (25% EtOAc in hexanes) gave ***d*-1f** as a yellow oil (215 mg, 46% yield); $R_f$= 0.31 (50% EtOAc in hexanes); $^1$H NMR (400 MHz, $CDCl_3$): δ 9.30 (s, 1H), 5.66 (dt, *J* = 17.2, 7.6 Hz, 1H), 4.98 (dd, *J* = 17.2, 1.2 Hz, 1H), 3.64 (s, 3H), 2.20 (dd, *J* = 7.6, 1.2 Hz, 2H), 1.09 (s, 6H); $^{13}$C NMR (100 MHz, $CDCl_3$): δ 174.9, 133.7, 117.9 (t, *J* = 25 Hz), 63.8, 44.7, 41.0, 24.4; IR (neat): 3212, 2967, 2934, 1645, 1498, 1473, 1048, 982, 903, 592 cm$^{-1}$; HRMS (m/z) Calcd for ($C_8H_{15}DNO_2$) ([M+H]$^+$): 159.1238; found: 159.1238.

**V. General Procedure for Diamination Reaction.**

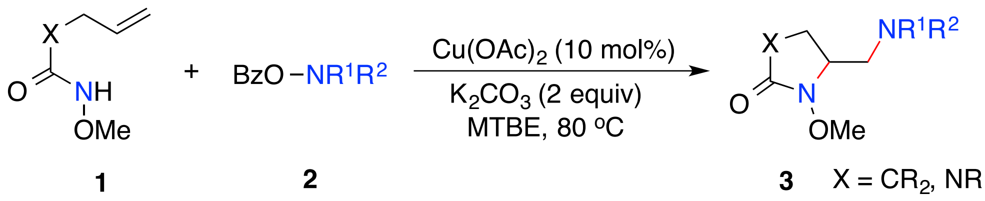


To a reaction tube charged with *N*-methoxylamide **1** (0.3 mmol, 1.0 equiv) and hydroxylamine **2** (0.36 mmol, 1.2 equiv), was added $Cu(OAc)_2$ (0.03 mmol, 10 mol%), $K_2CO_3$ (0.6 mmol, 2.0 equiv) and MTBE (1.5 mL). The reaction tube was capped and the resulting mixure was stirred at 80 °C for 2 h. After cooling down to room temperature, the reaction mixture was diluted with EtOAc (5 mL) and washed with a saturated aquous solution of $Na_2CO_3$ (5 mL). The aqueous layers were extracted with EtOAc (10 mL × 2). The combined organic layers were dried over $Na_2SO_4$, and filtered. The filtrate was concentrated *in vacuo*. The resulting crude mixture was subject to flash column chromatography to provide the diaminated product **3**.

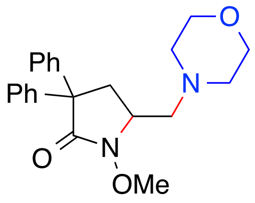


**1-Methoxy-5-(morpholinomethyl)-3,3-diphenylpyrrolidin-2-one (3aa).** Purification by column chromatography (50% EtOAc in hexanes then 50% EtOAc in hexanes containing 2% TEA) gave **3aa** as a yellow oil (87.9 mg, 80% yield); $R_f$= 0.25 (33% EtOAc in hexanes containing 2% TEA); $^1$H NMR (400 MHz, $CDCl_3$): δ 7.40–7.16 (m, 10H), 3.92–3.82 (m, 1H), 3.81 (s, 3H), 3.72–3.61 (m, 4H), 2.95 (dd, *J* = 12.8, 6.4 Hz, 1H), 2.75 (dd, *J* = 12.8, 4.0 Hz, 1H), 2.52 (dd, *J* = 12.8, 8.4 Hz, 1H), 2.50–2.40 (m, 5H); $^{13}$C NMR (100 MHz, $CDCl_3$): δ 171.9, 143.7, 141.7, 128.4, 128.2, 127.63, 127.62, 127.1, 126.8, 66.8, 62.7, 60.7, 54.3,

54.2, 53.0, 38.2; IR (neat): 2930, 2860, 2820, 1708, 1445, 1278, 1252, 1115, 697 $cm^{-1}$; HRMS (m/z) Calcd for ($C_{22}H_{27}N_2O_3$) ($[M+H]^+$): 367.2016; found: 367.2016.

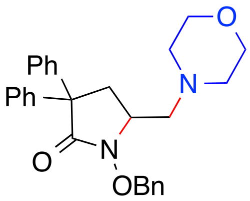


**1-(Benzyloxy)-5-(morpholinomethyl)-3,3-diphenylpyrrolidin-2-one (3ba).** The reaction was runned in a 0.2 mmol scale. Purification by column chromatography (50% EtOAc in hexanes then 50% EtOAc in hexanes containing 2% TEA) gave **3ba** as a yellow oil (65.4 mg, 74% yield); $R_f$= 0.32 (33% EtOAc in hexanes containing 2% TEA); $^1$H NMR (400 MHz, $CDCl_3$): δ 7.40–7.18 (m, 15H), 5.04 (d, $J$ = 10.4 Hz, 1H), 4.95 (d, $J$ = 10.4 Hz, 1H), 3.70–3.57 (m, 4H), 3.57–3.48 (m, 1H), 2.90 (dd, $J$ = 13.2, 6.4 Hz, 1H), 2.62 (dd, $J$ = 12.8, 4.0 Hz, 1H), 2.43 (dd, $J$ = 13.2, 8.0 Hz, 1H), 2.41–2.33 (m, 4H), 2.31 (dd, $J$ = 12.8, 7.2 Hz, 1H); $^{13}$C NMR (100 MHz, $CDCl_3$): δ 171.6, 144.1, 141.6, 135.0, 129.7, 128.8, 128.44, 128.42, 128.2, 127.71, 127.70, 127.1, 126.7, 76.8, 66.8, 60.4, 54.3, 54.1, 53.8, 38.1; IR (neat): 2918, 2850, 1701, 1493, 1445, 1114, 751, 695 $cm^{-1}$; HRMS (m/z) Calcd for ($C_{28}H_{31}N_2O_3$) ($[M+H]^+$): 443.2329; found: 443.2329.

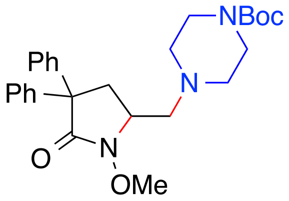


**1-Methoxy-5-(4'-*tert*-butyloxycarbonylpiperazinomethyl)-3,3-diphenylpyrrolidin-2-one (3ab).** Purification by column chromatography (50% EtOAc in hexanes then 50% EtOAc in hexanes containing 2% TEA) gave **3ab** as a yellow oil (114.4 mg, 82% yield); $R_f$= 0.31 (33% EtOAc in hexanes containing 2% TEA); $^1$H NMR (400 MHz, $CDCl_3$): δ 7.40–7.15 (m, 10H), 3.90–3.83 (m, 1H), 3.80 (s, 3H), 3.50–3.26 (m, 4H), 2.95 (dd, $J$ = 13.2, 6.4 Hz, 1H), 2.75 (dd, $J$ = 12.8, 4.0 Hz, 1H), 2.50 (dd, $J$ = 13.2, 8.0 Hz, 1H), 2.45 (dd, $J$ = 12.8, 7.2 Hz, 1H), 2.48–2.34 (m, 4H), 1.43 (s, 9H); $^{13}$C NMR (100 MHz, $CDCl_3$): δ 171.9, 154.6, 143.7, 141.6, 128.5, 128.3, 127.64, 127.62, 127.1, 126.8, 79.7, 62.6, 60.3, 54.2, 53.7 (2C), 53.1, 38.2, 28.3; IR (neat): 2975, 2934, 1695, 1421, 1246, 1171, 1006, 699 $cm^{-1}$; HRMS (m/z) Calcd for ($C_{27}H_{36}N_3O_4$) ($[M+H]^+$): 466.2700; found: 466.2698.

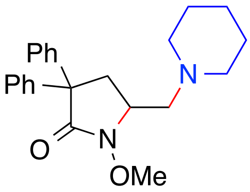


**1-Methoxy-3,3-diphenyl-5-(piperidinomethyl)pyrrolidin-2-one (3ac).** Purification by column chromatography (50% EtOAc in hexanes then 50% EtOAc in hexanes containing 2% TEA) gave **3ac** as a yellow oil (83.0 mg, 76% yield); $R_f$= 0.31 (33% EtOAc in hexanes containing 2% TEA); $^1$H NMR (400 MHz, $CDCl_3$): δ 7.42–7.16 (m, 10H), 3.90–3.81 (m, 1H), 3.81 (s, 3H), 2.97 (dd, $J$ = 13.2, 6.4 Hz, 1H), 2.75 (dd, $J$ = 12.8, 4.4 Hz, 1H), 2.49 (dd, $J$ = 13.2, 8.0 Hz, 1H), 2.49–2.29 (m, 5H), 1.63–1.47 (m, 4H), 1.40 (q, $J$ = 5.6 Hz, 2H); $^{13}$C NMR (100 MHz, $CDCl_3$): δ 172.0, 143.9, 141.8, 128.4, 128.2, 127.73, 127.69, 127.0, 126.7, 62.7, 61.4, 55.5, 54.3, 53.5, 38.6, 26.0, 24.1; IR (neat): 2935, 2778, 1707, 1444, 1025, 695 $cm^{-1}$; HRMS (m/z) Calcd for ($C_{23}H_{29}N_2O_2$) ($[M+H]^+$): 365.2224; found: 365.2222.

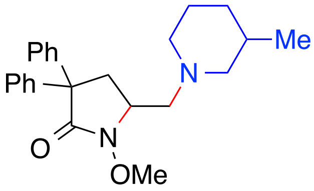

**1-Methoxy-5-(3'-methylpiperidinomethyl)-3,3-diphenylpyrrolidin-2-one (3ad).** Purification by column chromatography (50% EtOAc in hexanes then 50% EtOAc in hexanes containing 2% TEA) gave **3ad** as a yellow oil (79.9 mg, 71% yield, inseparable diastereoisomer, *dr* = 1:1); $R_f$= 0.34 (33% EtOAc in hexanes containing 2% TEA); $^1$H NMR (400 MHz, $CDCl_3$): δ 7.39–7.22 (m, 10H), 3.93–3.83 (m, 1H), 3.82 (s, 3H), 2.97 (dd, *J* = 12.8, 6.0 Hz, 1H), 2.82–2.67 (m, 3H), 2.56–2.44 (m, 1H), 2.37 (dd, *J* = 12.8, 7.6 Hz, 1H), 2.03–1.87 (m, 1H), 1.73–1.45 (m, 5H), 0.92–0.78 (m, 1H), 0.85 (d, *J* = 6.4 Hz, 3H); $^{13}$C NMR (100 MHz, $CDCl_3$): δ 172.0, 171.9, 143.91, 143.89, 141.9, 141.8, 128.4, 128.2, 127.73, 127.69, 127.03, 127.02, 126.7, 63.2, 62.77, 62.75, 62.7, 61.2, 61.1, 55.2, 54.6, 54.26, 54.24, 53.5, 38.62, 38.59, 32.69, 32.67, 31.08, 31.06, 25.48, 25.44, 19.60, 19.58; IR (neat): 2927, 1711, 1494, 1446, 1042, 1027, 761, 698 cm$^{-1}$; HRMS (m/z) Calcd for ($C_{24}H_{31}N_2O_2$) ($[M+H]^+$): 379.2380; found: 379.2381.

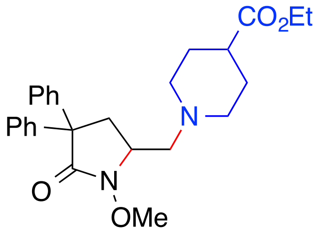


**1-Methoxy-5-(4'-ethoxylcarbonylpiperidinomethyl)-3,3-diphenylpyrrolidin-2-one (3ae).** Purification by column chromatography (50% EtOAc in hexanes then 50% EtOAc in hexanes containing 2% TEA) gave **3ae** as a yellow oil (102.7 mg, 81% yield); $R_f$= 0.26 (33% EtOAc in hexanes containing 2% TEA); $^1$H NMR (400 MHz, $CDCl_3$): δ 7.40–7.15 (m, 10H), 4.10 (q, *J* = 7.2 Hz, 2H), 3.88–3.80 (m, 1H), 3.79 (s, 3H), 2.95 (dd, *J* = 13.2, 6.4 Hz, 1H), 2.85–2.73 (m, 2H), 2.72 (dd, *J* = 12.4, 4.0 Hz, 1H), 2.48 (dd, *J* = 13.2, 8.0 Hz, 1H), 2.39 (dd, *J* = 12.4, 7.6 Hz, 1H), 2.24 (tt, *J* = 11.2, 4.0 Hz, 1H), 2.16–2.04 (m, 2H), 1.90–1.80 (m, 2H), 1.78–1.63 (m, 2H), 1.23 (t, *J* = 7.2 Hz, 3H); $^{13}$C NMR (100 MHz, $CDCl_3$): δ 175.0, 172.0, 143.8, 141.8, 128.5, 128.2, 127.68, 127.65, 127.0, 126.7, 62.7, 60.6, 60.3, 54.2, 54.1, 53.5, 53.4, 40.8, 38.3, 28.28, 28.22, 14.2; IR (neat): 2929, 1708, 1445, 1260, 1179, 1043, 1026, 760, 698 cm$^{-1}$; HRMS (m/z) Calcd for ($C_{26}H_{33}N_2O_4$) ($[M+H]^+$): 437.2435; found: 437.2435.

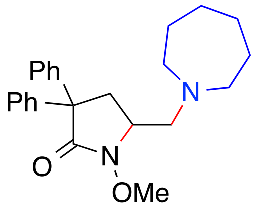


**5-(Azepanomethyl)-1-methoxy-3,3-diphenylpyrrolidin-2-one (3af).** Purification by column chromatography (50% EtOAc in hexanes then 50% EtOAc in hexanes containing 2% TEA) gave **3af** as a yellow oil (49.9 mg, 44% yield); $R_f$= 0.30 (33% EtOAc in hexanes containing 2% TEA); $^1$H NMR (400 MHz, $CDCl_3$): δ 7.38–7.32 (m, 4H), 7.32–7.25 (m, 4H), 7.25–7.18 (m, 2H), 3.88–3.78 (m, 1H), 3.80 (s, 3H), 3.05–2.95 (m, 2H), 2.73–2.64 (m, 4H), 2.58–2.47 (m, 2H), 1.72–1.48 (m, 8H); $^{13}$C NMR (100 MHz, $CDCl_3$): δ 171.9, 143.9, 141.7, 128.4, 128.2, 127.7, 127.6, 127.0, 126.7, 62.6, 59.8, 56.2, 54.2, 54.1, 38.3, 28.0, 27.0; IR (neat): 2925, 2852, 1708, 1445, 1042, 1026, 761, 698 cm$^{-1}$; HRMS (m/z) Calcd for ($C_{24}H_{31}N_2O_2$) ($[M+H]^+$): 379.2380; found: 379.2381.

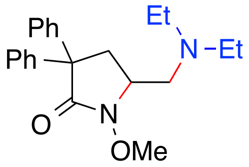


**5-((Diethylamino)methyl)-1-methoxy-3,3-diphenylpyrrolidin-2-one (3ag).** Purification by column chromatography (50% EtOAc in hexanes then 50% EtOAc in hexanes containing 2% TEA) gave **3ag** as a yellow oil (48.9 mg, 47% yield); $R_f$= 0.33 (33% EtOAc in hexanes containing 2% TEA); $^1$H NMR (400 MHz, $CDCl_3$): δ 7.39–7.17 (m, 10H), 3.84–3.74 (m, 1H), 3.81 (s, 3H), 3.03 (dd, *J* = 13.2, 6.4 Hz, 1H), 2.87 (dd, *J* = 13.2, 3.6 Hz, 1H), 2.61–2.39 (m, 6H), 0.97 (t, *J* = 7.2 Hz, 6H); $^{13}$C NMR (100 MHz, $CDCl_3$): δ 171.7, 144.0, 141.5, 128.3, 128.1, 127.6, 127.5, 126.9, 126.5, 62.4, 55.5, 54.12, 54.07, 47.8, 38.5, 11.7; IR

(neat): 2971, 2932, 2811, 1707, 1444, 1025, 696 cm$^{-1}$; HRMS (m/z) Calcd for ($C_{22}H_{29}N_2O_2$) ($[M+H]^+$): 353.2224; found: 353.2223.

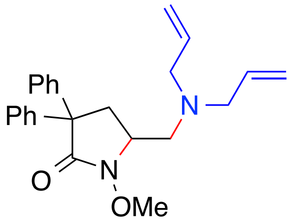


**5-((Diallylamino)methyl)-1-methoxy-3,3-diphenylpyrrolidin-2-one (3ah).** Purification by column chromatography (50% EtOAc in hexanes then 50% EtOAc in hexanes containing 2% TEA) gave **3ah** as a yellow oil (45.4 mg, 40% yield); $R_f$= 0.40 (33% EtOAc in hexanes containing 2% TEA); $^1$H NMR (400 MHz, $CDCl_3$): δ 7.38–7.18 (m, 10H), 5.85–5.70 (m, 2H), 5.18–5.07 (m, 4H), 3.88–3.80 (m, 1H), 3.78 (s, 3H), 3.16–3.03 (m, 4H), 2.99 (dd, *J* = 13.2, 6.4 Hz, 1H), 2.86 (dd, *J* = 12.8, 4.0 Hz, 1H), 2.50 (dd, *J* = 13.2, 8.4 Hz, 1H), 2.44 (dd, *J* = 13.2, 8.0 Hz, 1H); $^{13}$C NMR (100 MHz, $CDCl_3$): δ 171.9, 144.0, 141.6, 135.3, 128.4, 128.3, 127.74, 127.67, 127.1, 126.7, 117.8, 62.5, 58.0, 55.6, 54.2, 53.9, 38.4; IR (neat): 2931, 2813, 1710, 1446, 1261, 1042, 1027, 920, 761, 698 cm$^{-1}$; HRMS (m/z) Calcd for ($C_{24}H_{29}N_2O_2$) ($[M+H]^+$): 377.2222; found: 377.2222.

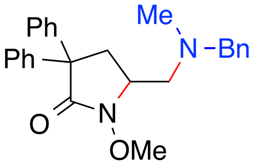


**5-((Benzyl(methyl)amino)methyl)-1-methoxy-3,3-diphenylpyrrolidin-2-one (3ai).** Purification by column chromatography (50% EtOAc in hexanes then 50% EtOAc in hexanes containing 2% TEA) gave **3ai** as a colorless oil (64.2 mg, 54% yield); $R_f$= 0.33 (33% EtOAc in hexanes containing 2% TEA); $^1$H NMR (400 MHz, $CDCl_3$): δ 7.36–7.20 (m, 15H), 3.90–3.82 (m, 1H), 3.81 (s, 3H), 3.57 (d, *J* = 12.8 Hz, 1H), 3.51 (d, *J* = 12.8 Hz, 1H), 2.99 (dd, *J* = 13.2, 6.4 Hz, 1H), 2.87 (dd, *J* = 12.4, 4.0 Hz, 1H), 2.52 (dd, *J* = 12.4, 8.4 Hz, 1H), 2.48 (dd, *J* = 13.2, 8.0 Hz, 1H), 2.27 (s, 3H); $^{13}$C NMR (100 MHz, $CDCl_3$): δ 172.0, 143.8, 141.8, 138.7, 128.9, 128.5, 128.3, 128.2, 127.7, 127.6, 127.2, 127.0, 126.7, 63.2, 62.7, 59.7, 54.2, 53.7, 43.5, 38.3; IR (neat): 2921, 2848, 1705, 1448, 1024, 698 cm$^{-1}$; HRMS (m/z) Calcd for ($C_{26}H_{29}N_2O_2$) ($[M+H]^+$): 401.2224; found: 401.2222.

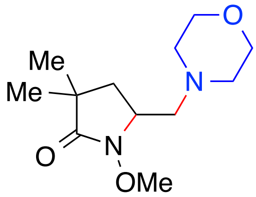


**1-Methoxy-3,3-dimethyl-5-(morpholinomethyl)pyrrolidin-2-one (3fa).** Purification by column chromatography (50% EtOAc in hexanes then 50% EtOAc in hexanes containing 2% TEA) gave **3fa** as a yellow oil (65.1 mg, 90% yield); $R_f$= 0.29 (50% EtOAc in hexanes containing 2% TEA); $^1$H NMR (400 MHz, $CDCl_3$): δ 3.80–3.70 (m, 1H), 3.74 (s, 3H), 3.66–3.61 (m, 4H), 2.66 (dd, *J* = 12.8, 4.4 Hz, 1H), 2.50–2.42 (m, 4H), 2.37 (dd, *J* = 12.8, 7.2 Hz, 1H), 1.98 (dd, *J* = 12.8, 7.2 Hz, 1H), 1.62 (dd, *J* = 12.8, 7.6 Hz, 1H), 1.16 (s, 3H), 1.07 (s, 3H); $^{13}$C NMR (100 MHz, $CDCl_3$): δ 176.2, 66.8, 62.4, 61.4, 54.4, 52.7, 37.8, 37.3, 25.7, 25.1; IR (neat): 2962, 2848, 2808, 1699, 1454, 1252, 1110, 1009, 859 cm$^{-1}$; HRMS (m/z) Calcd for ($C_{12}H_{23}N_2O_3$) ($[M+H]^+$): 243.1704; found: 243.1704.

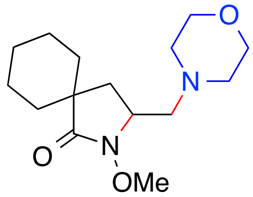

**2-Methoxy-3-(morpholinomethyl)-2-azaspiro[4.5]decan-1-one (3ga).** Purification by column chromatography (50% EtOAc in hexanes then 50% EtOAc in hexanes containing 2% TEA) gave **3ga** as a yellow oil (69 mg, 81% yield); $R_f$= 0.28 (50% EtOAc in hexanes containing 2% TEA); $^1$H NMR (400 MHz, $CDCl_3$): δ 3.75–3.68 (m, 1H), 3.72 (s, 3H), 3.63 (t, *J* = 4.8 Hz, 4H), 2.65 (dd, *J* = 12.8, 4.0 Hz, 1H), 2.45 (t, *J* = 4.8 Hz, 4H), 2.35 (dd, *J* = 12.8, 7.2 Hz, 1H), 2.11 (dd, *J* = 13.2, 7.6 Hz, 1H), 1.75–1.45 (m, 6H), 1.40–1.16 (m, 5H); $^{13}$C NMR (100 MHz, $CDCl_3$): δ 176.0, 66.8, 62.5, 61.6, 54.4, 53.0, 41.4, 34.2, 33.5, 32.0, 25.1, 21.54, 21.50; IR (neat): 2924, 2851, 2798, 2755, 1701, 1442, 1364, 1275, 1114, 866 cm$^{-1}$; HRMS (m/z) Calcd for ($C_{15}H_{27}N_2O_3$) ([M+H]$^+$): 283.2016; found: 283.2014.

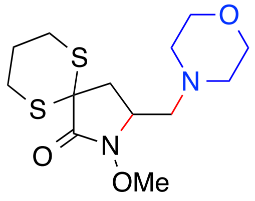


**2-Methoxy-3-(morpholinomethyl)-6,10-dithia-2-azaspiro[4.5]decan-1-one (3ha).** Purification by column chromatography (50% EtOAc in hexanes then 50% EtOAc in hexanes containing 2% TEA) gave **3ha** as a yellow oil (48.6 mg, 51% yield); $R_f$= 0.29 (50% EtOAc in hexanes containing 2% TEA); $^1$H NMR (400 MHz, $CDCl_3$): δ 3.72 (t, *J* = 13.2 Hz, 1H), 3.65–3.55 (m, 1H), 3.53 (s, 3H), 3.44 (t, *J* = 13.2 Hz, 1H), 3.39 (t, *J* = 4.8 Hz, 4H), 2.46 (dd, *J* = 12.8, 4.4 Hz, 1H), 2.32–2.24 (m, 1H), 2.28 (dd, *J* = 14.0, 3.6 Hz, 1H), 2.19 (t, *J* = 4.8 Hz, 4H), 2.22–2.14 (m, 1H), 1.95 (dd, *J* = 14.0, 6.8 Hz, 1H), 1.97–1.88 (m, 1H), 1.71 (dd, *J* = 14.0, 7.2 Hz, 1H), 1.67–1.52 (m, 1H); $^{13}$C NMR (100 MHz, $CDCl_3$): δ 169.8, 66.8, 62.4, 60.5, 54.3, 52.5, 43.1, 37.7, 27.4, 27.0, 24.3; IR (neat): 2922, 2851, 2813, 1706, 1423, 1278, 1116, 865 cm$^{-1}$; HRMS (m/z) Calcd for ($C_{13}H_{23}N_2O_3S_2$) ([M+H]$^+$): 319.1145; found: 319.1145.

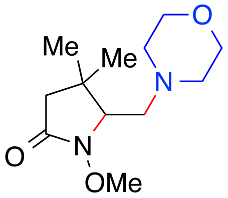


**1-Methoxy-4,4-dimethyl-5-(morpholinomethyl)pyrrolidin-2-one (3ia).** Purification by column chromatography (50% EtOAc in hexanes then 50% EtOAc in hexanes containing 2% TEA) gave **3ia** as a yellow oil (26.2 mg, 36% yield); $R_f$= 0.26 (50% EtOAc in hexanes containing 2% TEA); $^1$H NMR (400 MHz, $CDCl_3$): δ 3.77 (s, 3H), 3.65 (t, *J* = 4.4 Hz, 4H), 3.43 (t, *J* = 5.6 Hz, 1H), 2.55 (d, *J* = 5.6 Hz, 2H), 2.52–2.41 (m, 4H), 2.27 (d, *J* = 16.4 Hz, 1H), 2.05 (d, *J* = 16.4 Hz, 1H), 1.20 (s, 3H), 1.10 (s, 3H); $^{13}$C NMR (125 MHz, $CDCl_3$): δ 163.4, 60.0, 56.7, 55.4, 49.2, 47.3, 36.5, 26.8, 22.0, 16.0; IR (neat): 2957, 2851, 1702, 1453, 1292, 1114, 1061, 865 cm$^{-1}$; HRMS (m/z) Calcd for ($C_{12}H_{23}N_2O_3$) ([M+H]$^+$): 243.1703; found: 243.1703.

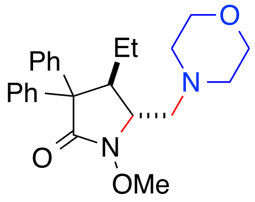


**(*trans*)-4-Ethyl-1-methoxy-5-(morpholinomethyl)-3,3-diphenylpyrrolidin-2-one (3ja).** Purification by column chromatography (50% EtOAc in hexanes then 50% EtOAc in hexanes containing 2% TEA) gave **3ja** as a yellow oil (66.3 mg, 56% yield, *dr* > 20:1); $R_f$= 0.28 (33% EtOAc in hexanes containing 2% TEA); $^1$H NMR (400 MHz, $CDCl_3$): δ 7.64 (d, *J* = 7.6 Hz, 2H), 7.34 (t, *J* = 7.6 Hz, 2H), 7.28 (d, *J* = 7.6 Hz, 1H), 7.25–7.15 (m, 3H), 6.94 (d, *J* = 7.6 Hz, 2H), 3.93 (s, 3H), 3.69–3.53 (m, 5H), 3.10 (dt, *J* = 8.8, 5.6 Hz, 1H), 2.57 (dd, *J* = 4.8, 2.0 Hz, 2H), 2.53–2.38 (m, 4H), 1.20–1.00 (m, 2H), 0.88 (t, *J* = 7.6 Hz, 3H); $^{13}$C NMR (100 MHz, $CDCl_3$): δ 172.1, 141.7, 141.5, 128.9, 128.4, 128.0, 127.9, 127.1, 126.7, 66.9, 62.6, 59.4, 58.7, 58.1, 54.3, 43.7, 24.2, 12.7; IR (neat): 2960, 2933, 2850, 2809, 1707, 1494, 1444, 1116, 700 cm$^{-1}$; HRMS (m/z) Calcd for ($C_{24}H_{31}N_2O_3$) ([M+H]$^+$): 395.2329; found: 395.2328.

The relative stereochemistry of **3ja** was determined by NOESY.

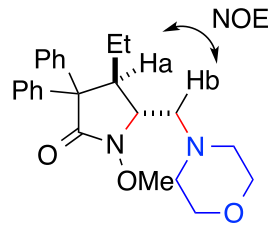


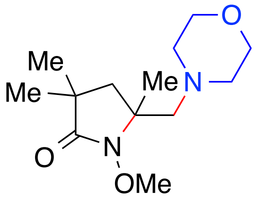


**1-Methoxy-3,3,5-trimethyl-5-(morpholinomethyl)pyrrolidin-2-one (3ka).** Purification by column chromatography (50% EtOAc in hexanes then 50% EtOAc in hexanes containing 2% TEA) gave **3ka** as a yellow oil (48.4 mg, 62% yield); $R_f$= 0.35 (50% EtOAc in hexanes containing 2% TEA); $^1$H NMR (400 MHz, $CDCl_3$): δ 3.80 (s, 3H), 3.65–3.50 (m, 4H), 2.65–2.54 (m, 2H), 2.52 (d, *J* = 13.6 Hz, 1H), 2.41–2.29 (m, 2H), 2.03 (d, *J* = 6.0 Hz, 1H), 2.00 (d, *J* = 6.0 Hz, 1H), 1.56 (d, *J* = 13.6 Hz, 1H), 1.27 (s, 3H), 1.23 (s, 3H), 1.18 (s, 3H); $^{13}$C NMR (100 MHz, $CDCl_3$): δ 174.9, 67.0, 63.8 (2C), 61.1, 55.2, 43.0, 36.4, 29.4, 26.0, 25.0; IR (neat): 2964, 2853, 2805, 1697, 1454, 1381, 1356, 1315, 1115, 1009, 864 cm$^{-1}$; HRMS (m/z) Calcd for ($C_{13}H_{25}N_2O_3$) ([M+H]$^+$): 257.1860; found: 257.1860.

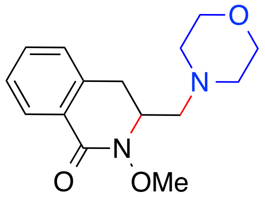


**2-Methoxy-3-(morpholinomethyl)-3,4-dihydroisoquinolin-1(2*H*)-one (3la).** Purification by column chromatography (50% EtOAc in hexanes then 50% EtOAc in hexanes containing 2% TEA) gave **3la** as a yellow oil (53 mg, 65% yield); $R_f$= 0.23 (50% EtOAc in hexanes containing 2% TEA); $^1$H NMR (400 MHz, $CDCl_3$): δ 8.04 (d, *J* = 7.6 Hz, 1H), 7.38 (t, *J* = 7.6 Hz, 1H), 7.28 (t, *J* = 7.6 Hz, 1H), 7.13 (d, *J* = 7.6 Hz, 1H), 4.00–3.92 (m, 1H), 3.85 (s, 3H), 3.61–3.51 (m, 2H), 3.51–3.42 (m, 2H), 3.36 (dd, *J* = 16.0, 6.0 Hz, 1H), 3.16 (dd, *J* = 16.0, 2.4 Hz, 1H), 2.55 (dd, *J* = 13.2, 3.6 Hz, 1H), 2.47–2.35 (m, 3H), 2.35–2.25 (m, 2H); $^{13}$C NMR (100 MHz, $CDCl_3$): δ 162.9, 135.5, 132.1, 128.6, 127.68, 127.66, 126.9, 66.8, 62.6, 57.9, 56.6, 54.1, 32.2; IR (neat): 2933, 2852, 2808, 1663, 1458, 1266, 1114, 1004, 866, 730, 689 cm$^{-1}$; HRMS (m/z) Calcd for ($C_{15}H_{21}N_2O_3$) ([M+H]$^+$): 277.1547; found: 277.1547.

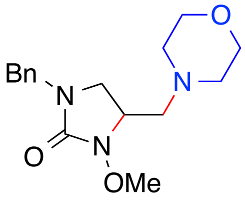


**1-Benzyl-3-methoxy-4-(morpholinomethyl)imidazolidin-2-one (3ma).** Purification by column chromatography (50% EtOAc in hexanes then 50% EtOAc in hexanes containing 2% TEA) gave **3ma** as a yellow oil (36.8 mg, 41% yield); $R_f$= 0.24 (50% EtOAc in hexanes containing 2% TEA); $^1$H NMR (400 MHz, $CDCl_3$): δ 7.35–7.15 (m, 5H), 4.40 (d, *J* = 14.8 Hz, 1H), 4.25 (d, *J* = 14.8 Hz, 1H), 3.82 (s, 3H), 3.70–3.52 (m, 1H), 3.60 (t, *J* = 4.0 Hz, 4H), 3.22 (t, *J* = 8.4 Hz, 1H), 2.86 (t, *J* = 9.2 Hz, 1H), 2.70 (dd, *J* = 12.4, 4.8 Hz, 1H), 2.44 (dd, *J* = 12.4, 8.0 Hz, 1H), 2.46–2.32 (m, 4H); $^{13}$C NMR (100 MHz, $CDCl_3$): δ 162.5, 135.8, 128.6, 128.1, 127.6, 66.7, 64.4, 60.5, 56.6, 54.1, 47.7, 45.5; IR (neat): 2934, 2852, 2811, 1723, 1436, 1239, 1114, 1034, 699 cm$^{-1}$; HRMS (m/z) Calcd for ($C_{16}H_{24}N_3O_3$) ([M+H]$^+$): 306.1812; found: 306.1812.

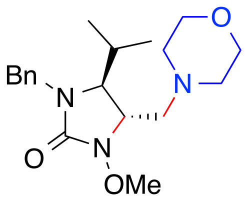


**(4*S*,5*S*)-1-Benzyl-5-isopropyl-3-methoxy-4-(morpholinomethyl)imidazolidin-2-one (3na).** Purification by column chromatography (50% EtOAc in hexanes then 50% EtOAc in hexanes containing 2% TEA) gave **3na** as a yellow oil (47 mg, 46% yield); $[\alpha]^{20}_{D}$ = −18.3 (c = 0.6, $CHCl_3$); $R_f$ = 0.31 (33% EtOAc in hexanes containing 2% TEA); $^1$H NMR (400 MHz, $CDCl_3$): δ 7.35–7.18 (m, 5H), 4.90 (d, *J* = 14.8 Hz, 1H), 3.86 (d, *J* = 14.8 Hz, 1H), 3.85 (s, 3H), 3.56 (t, *J* = 4.4 Hz, 4H), 3.41 (q, *J* = 5.6 Hz, 1H), 3.13 (dd, *J* = 5.6, 3.6 Hz, 1H), 2.47 (dd, *J* = 13.2, 5.6 Hz, 1H), 2.44–2.29 (m, 4H), 2.25 (dd, *J* = 13.2, 5.6 Hz, 1H), 2.06–1.94 (m, 1H), 0.83 (d, *J* = 3.6 Hz, 3H), 0.81 (d, *J* = 3.6 Hz, 3H); $^{13}$C NMR (125 MHz, $CDCl_3$): δ 161.7, 136.3, 128.6, 128.1, 127.6, 66.8, 64.0, 60.2, 59.1, 55.5, 54.2, 45.1, 27.4, 18.1, 15.8; IR (neat): 2958, 2852, 2808, 1717, 1424, 1115, 700 cm$^{-1}$; HRMS (m/z) Calcd for ($C_{19}H_{30}N_3O_3$) ($[M+H]^+$): 348.2282; found: 348.2283.

The relative stereochemistry of **3pa** was determined by NOESY.

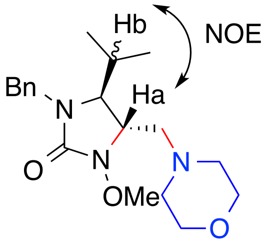


**VI. Mechanism Investigation.**

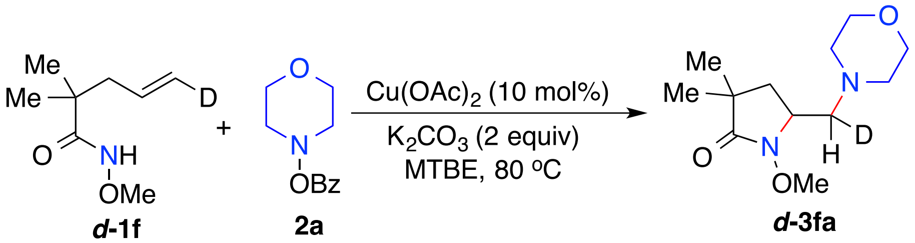


To a reaction tube charged with (*E*)-*N*-methoxy-2,2-dimethylpent-4-en-5-*d*-amide ***d*-1f** (31.6 mg, 0.2 mmol) and hydroxylamine **2a** (49.7 mg, 0.24 mmol), was added $Cu(OAc)_2$ (3.6 mg, 0.02 mmol), $K_2CO_3$ (55.2 mg, 0.4 mmol), and MTBE (1 mL). The reaction tube was capped and the resulting mixure was stirred at 80 °C for 2 h. After cooling down to room temperature, the reaction mixture was diluted with EtOAc (5 mL) and washed with a saturated aqueous solution of $Na_2CO_3$ (5 mL). The aqueous layer was separated and extracted with EtOAc (10 mL × 2). The combined organic layers were dried over $Na_2SO_4$, and filtered. The filtrate was concentrated *in vacuo*. Purification by column chromatography (50% EtOAc in hexanes then 50% EtOAc in hexanes containing 2% TEA) gave 1-methoxy-3,3-dimethyl-5-(morpholinomethyl-*d*)pyrrolidin-2-one ***d*-3fa** as a yellow oil (32.1 mg, 66% yield, inseparable diastereoisomer, *dr* = 1:1); $R_f$ = 0.29 (50% EtOAc in hexanes containing 2% TEA); $^1$H NMR (400 MHz, $CDCl_3$): δ 3.79–3.72 (m, 1H), 3.75 (s, 3H), 3.68–3.62 (m, 4H), 2.67 (d, *J* = 4.4 Hz, 0.5H), 2.53–2.40 (m, 4H), 2.36 (d, *J* = 6.8 Hz, 0.5H), 1.99 (dd, *J* = 12.8, 7.6 Hz, 1H), 1.62 (dd, *J* = 12.8, 7.6 Hz, 1H), 1.17 (s, 3H), 1.08 (s, 3H); $^{13}$C NMR (100 MHz, $CDCl_3$): δ 176.2, 66.9, 62.5, 61.0 (t, *J* = 20 Hz), 54.4, 52.7, 37.8, 37.3, 25.7, 25.1; IR (neat): 2962, 2848, 2809, 1699, 1453, 1270, 1112, 1013, 859 cm$^{-1}$; HRMS (m/z) Calcd for ($C_{12}H_{22}DN_2O_3$) ($[M+H]^+$): 244.1766; found: 244.1765.

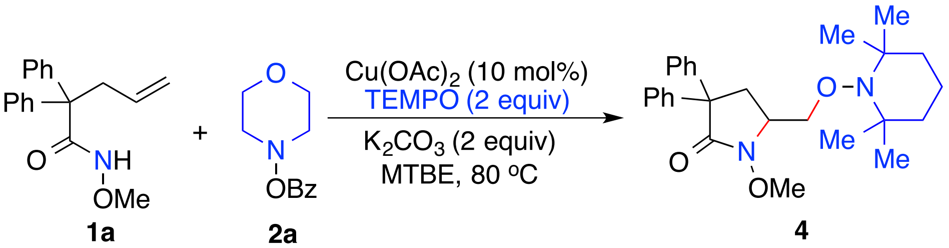

To a reaction tube charged with *N*-methoxy-2,2-diphenylpent-4-enamide (56.2 mg, 0.2 mmol) and hydroxylamine (49.7 mg, 0.24 mmol), was added $Cu(OAc)_2$ (3.6 mg, 0.02 mmol), $K_2CO_3$ (55.2 mg, 0.4 mmol), TEMPO (55.2 mg, 0.4 mmol) and MTBE (1 mL). The reaction tube was capped and the resulting mixure was stirred at 80 °C for 24 h. After cooling down to room temperature, the reaction mixture was diluted with EtOAc (5 mL), washed with a saturated aqueous solution of $Na_2S_2O_3$ (5 mL) and a saturated aqueous solution of $Na_2CO_3$ (5 mL). The aqueous layers were extracted with EtOAc (10 mL × 2). The combined organic layers were dried over $Na_2SO_4$, and filtered. The filtrate was concentrated *in vacuo*. Purification by column chromatography (5% EtOAc in hexanes) gave 1-methoxy-3,3-diphenyl-5-(((2,2,6,6-tetramethylpiperidin-1-yl)oxy)-methyl)pyrrolidin-2-one **4** as a colorless oil (31.4 mg, 36% yield); $R_f$= 0.25 (12.5% EtOAc in hexanes); $^1$H NMR (400 MHz, $CDCl_3$): δ 7.42–7.20 (m, 10H), 4.04–3.93 (m, 2H), 3.93–3.82 (m, 1H), 3.85 (s, 3H), 2.93 (dd, *J* = 12.8, 6.4 Hz, 1H), 2.64 (dd, *J* = 13.2, 8.8 Hz, 1H), 1.52–1.40 (m, 4H), 1.37–1.27 (m, 2H), 1.17 (s, 3H), 1.16 (s, 3H), 1.11 (s, 3H), 1.08 (s, 3H); $^{13}$C NMR (100 MHz, $CDCl_3$): δ 171.7, 143.8, 141.4, 128,5, 128.3, 127.8, 127.7, 127.2, 126.8, 76.0, 62.4, 60.0, 54.3, 53.6, 39.7, 36.3, 20.1, 16.9; IR (neat): 2971, 2928, 1712, 1446, 1373, 1359, 1045, 697 cm$^{-1}$; HRMS (m/z) Calcd for ($C_{27}H_{37}N_2O_3$) ($[M+H]^+$): 437.2799; found: 437.2797.

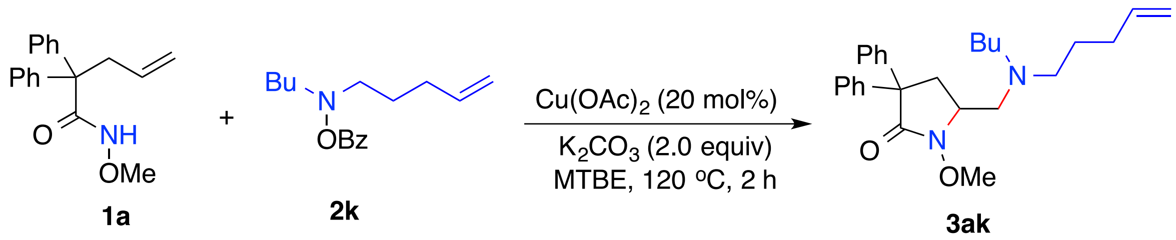


Follow the diamination reaction procedure. The reaction was runned in a 0.2 mmol scale. Purification by column chromatography (20% EtOAc in hexanes) gave 5-((butyl(pent-4’-en-1’-yl)amino)methyl)-1-methoxy-3,3-diphenylpyrrolidin-2-one **3ak** as a colorless oil (37.8 mg, 45% yield); $R_f$= 0.50 (33% EtOAc in hexanes containing 2% TEA); $^1$H NMR (400 MHz, $CDCl_3$): δ 7.42–7.19 (m, 10H), 5.85–5.71 (m, 1H), 5.04–4.91 (m, 2H), 3.87–3.78 (m, 1H), 3.82 (s, 3H), 3.06 (dd, *J* = 13.2, 6.4 Hz, 1H), 2.88 (dd, *J* = 13.2, 3.6 Hz, 1H), 2.60–2.34 (m, 6H), 2.10–1.98 (m, 2H), 1.58–1.42 (m, 2H), 1.42–1.20 (m, 4H), 0.89 (t, *J* = 7.2 Hz, 3H); $^{13}$C NMR (125 MHz, $CDCl_3$): δ 172.0, 144.1, 141.5, 138.4, 128.5, 128.3, 127.7, 127.6, 127.1, 126.8, 114.7, 62.7, 57.0, 54.7, 54.3, 54.2 (2C), 38.7, 31.3, 29.3, 26.3, 20.4, 14.0; IR (neat): 2931, 2861, 2814, 1709, 1446, 1377, 1195, 1041, 1027, 697 cm$^{-1}$; HRMS (m/z) Calcd for ($C_{27}H_{37}N_2O_3$) ($[M+H]^+$): 421.2850; found: 421.2847.

## VII. Deprotection Conditions for 3aa.

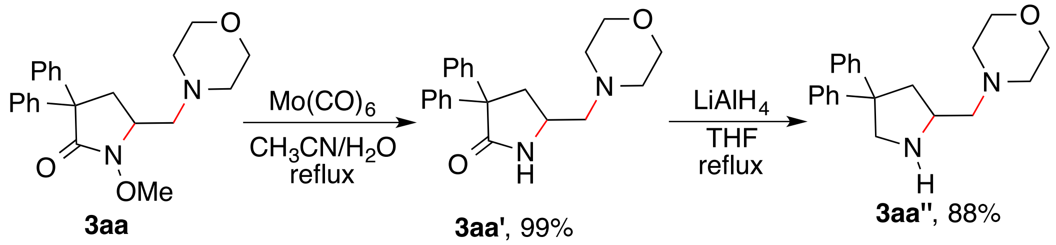


To a solution of **3aa** (73.2 mg, 0.2 mmol) in $CH_3CN$-$H_2O$ (3.2 mL, 15:1) at room temperature was added $Mo(CO)_6$[4] (63.4 mg, 0.24 mmol). The resulting mixture was heated and refluxed for 2 h. The solvent was removed uder reduced pressure. Purification by column chromatography (50% EtOAc in hexanes containing 2% TEA) gave 5-(morpholinomethyl)-3,3-diphenylpyrrolidin-2-one **3aa’** as a white solid (66.8 mg, 99% yield); $R_f$= 0.14 (50% EtOAc in hexanes containing 2% TEA); $^1$H NMR (400 MHz, $CDCl_3$): δ 7.42 (d, *J* = 7.6 Hz, 2H), 7.38–7.17 (m, 8H), 6.90 (s, br, 1H), 3.80–3.70 (m, 1H), 3.70–3.57 (m, 4H), 2.94 (dd, *J* = 13.2, 6.0 Hz, 1H), 2.58–2.48 (m, 2H), 2.46 (dd, *J* = 12.8, 4.0 Hz, 1H), 2.42–2.30 (m, 4H); $^{13}$C NMR (100 MHz, $CDCl_3$): δ 177.1, 143.9, 141.6, 128.4, 128.0, 127.9, 127.6, 127.0, 126.5, 66.8, 64.0, 57.7, 53.8, 47.6, 41.7; IR (neat): 2921, 2864, 2802, 1696, 1558, 1443, 1301, 1109, 909, 725, 696 cm$^{-1}$; HRMS (m/z) Calcd for ($C_{21}H_{25}N_2O_2$) ($[M+H]^+$): 337.1911; found: 337.1912.

To a 25 mL round-bottomed flask was added $LiAlH_4$ (38 mg, 1 mmol) followed by THF (5 mL) and the solution of **3aa’** (66.8 mg, 0.2 mmol) in THF (5 mL) at room temperature. The resulting mixture was heated

and refluxed overnight. The reaction was quenched with the addition of an anqueous solution of NaOH (0.2 M, 5 mL). The mixture was filtered through a pale of Celite. The solvent was removed under reduced pressure. Purification by column chromatography (5% MeOH in $CH_2Cl_2$) gave 5-(morpholinomethyl)-3,3-diphenylpyrrolidine **3aa''** as a colorless oil (56.8 mg, 88% yield); $R_f$= 0.13 (5% MeOH in $CH_2Cl_2$); $^1$H NMR (400 MHz, $CDCl_3$): δ 7.35–7.19 (m, 8H), 7.19–7.11 (m, 2H), 3.76–3.60 (m, 5H), 3.55–3.45 (m, 1H), 3.45 (d, $J$ = 11.2 Hz, 1H), 2.80–2.70 (m, 2H), 2.54–2.28 (m, 6H), 2.05 (dd, $J$ = 12.8, 8.8 Hz, 1H); $^{13}$C NMR (100 MHz, $CDCl_3$): δ 147.2, 146.5, 128.4 (2C), 128.3 (2C), 127.0 (2C), 126.8 (2C), 126.1 (2C), 66.9, 65.0, 57.0, 56.1, 54.2, 54.0, 43.4; IR (neat): 2958, 2855, 2807, 1734, 1445, 1372, 1240, 1115, 909, 726, 698 cm$^{-1}$; HRMS (m/z) Calcd for ($C_{21}H_{27}N_2O$) ($[M+H]^+$): 323.2123; found: 323.2125.

**VIII. Synthesis of (±)-FAUC-179**

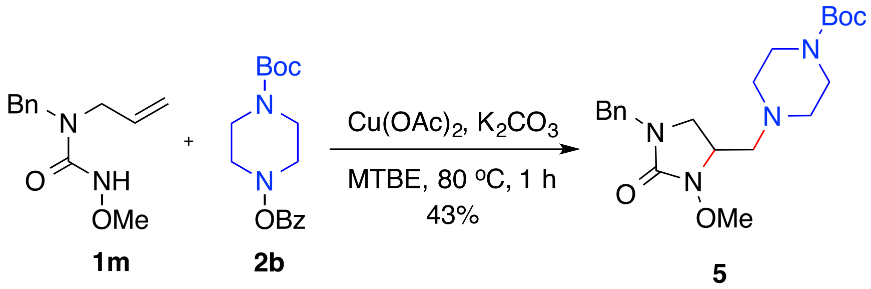


Follow the diamination procedure. Purification by column chromatography (50% EtOAc in hexanes then 50% EtOAc in hexanes containing 2% TEA) gave *tert*-butyl 4-((1'-benzyl-3'-methoxy-2'-oxoimidazolidin-4'-yl)methyl)piperazine-1-carboxylate **5** as a yellow oil (51.6 mg, 43% yield); $R_f$ = 0.45 (5% MeOH in $CH_2Cl_2$); $^1$H NMR (400 MHz, $CDCl_3$): δ 7.34–7.18 (m, 5H), 4.40 (d, $J$ = 14.8 Hz, 1H), 4.27 (d, $J$ = 14.8 Hz, 1H), 3.82 (s, 3H), 3.65–3.55 (m, 1H), 3.40–3.25 (m, 4H), 3.22 (t, $J$ = 8.4 Hz, 1H), 2.86 (t, $J$ = 9.2 Hz, 1H), 2.71 (dd, $J$ = 13.2, 4.8 Hz, 1H), 2.46 (dd, $J$ = 13.2, 8.4 Hz, 1H), 2.40–2.25 (m, 4H), 1.41 (s, 9H); $^{13}$C NMR (100 MHz, $CDCl_3$): δ 162.5, 154.6, 135.9, 128.7, 128.1, 127.7, 79.6, 64.5, 60.1, 56.8, 53.5, 47.7, 45.5, 28.3; IR (neat): 2973, 2932, 2812, 1732, 1689, 1419, 1246, 1169, 1124, 1036, 1005, 701 cm$^{-1}$; HRMS (m/z) Calcd for ($C_{21}H_{33}N_4O_4$) ($[M+H]^+$): 405.2496; found: 405.2496.

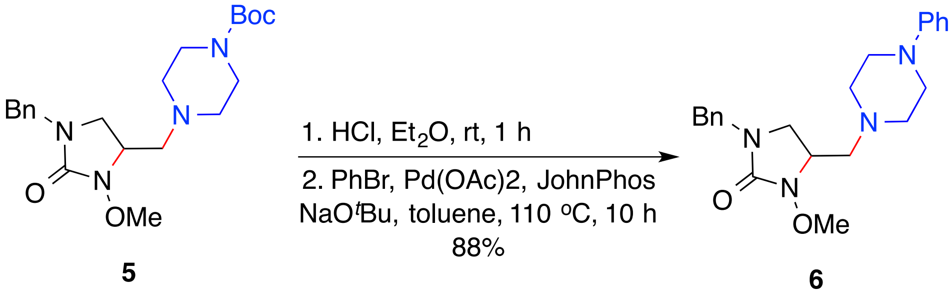


To a round-bottomed flask was charged with **5** (80.8 mg, 0.2 mmol) followed by the addition of HCl (2 M in $Et_2O$, 10 mL). The resulting mixture was stirred at room temperature for 1 h and then was added an aqueous solution of NaOH (2 M, 10 mL). The organic layer was separated and the aqueous layer was extracted with EtOAc. The combined organic solvent was dried over $Na_2SO_4$ and filtered off. The solvent was removed under reduced pressure and the residue was used for the next step. To the crude mixture, were added PhBr (47.1 mg, 0.3 mmol), NaO$^t$Bu (28.8 mg, 0.3 mmol), $Pd(OAc)_2$ (0.9 mg, 0.004 mmol), JohnPhos (2.4 mg, 0.008 mmol) and toluene (2 mL). The resulting mixture was stirred at 110 °C for 10 h under nitrogen atmosphere. Then the reaction mixture was cooled down to room temperature and filtered through a pale of Celite. The filtrate was concentrated under reduced pressure. Purification by column chromatography (50% EtOAc in hexanes then 100% EtOAc) gave 1-benzyl-3-methoxy-4-((4'-phenylpiperazin-1'-yl)methyl)imidazolidin-2-one **6** as a yellow oil (66.7 mg, 88% yield); $R_f$= 0.43 (5% MeOH in $CH_2Cl_2$); $^1$H NMR (400 MHz, $CDCl_3$): δ 7.36–7.21 (m, 7H), 6.89 (d, $J$ = 8.8 Hz, 2H), 6.84 (t, $J$ = 7.6 Hz, 1H), 4.44 (d, $J$ = 14.8 Hz, 1H), 4.30 (d, $J$ = 14.8 Hz, 1H), 3.86 (s, 3H), 3.72–3.65 (m, 1H), 3.28 (t, $J$ = 8.6 Hz, 1H), 3.19–3.06 (m, 4H), 2.91 (t, $J$ = 9.2 Hz, 1H), 2.79 (dd, $J$ = 12.4, 4.8 Hz, 1H), 2.67–2.49 (m, 5H); $^{13}$C NMR (100 MHz, $CDCl_3$): δ 162.6, 151.1, 136.0, 129.1, 128.7, 128.2, 127.7, 116.0, 64.5, 60.2, 57.0, 53.8, 49.0, 47.8, 45.7; IR (neat): 2936, 2819, 1732, 1599, 1496, 1437, 1233, 1035, 759, 699 cm$^{-1}$; HRMS (m/z) Calcd for ($C_{22}H_{29}N_4O_2$) ($[M+H]^+$): 381.2285; found: 381.2287.

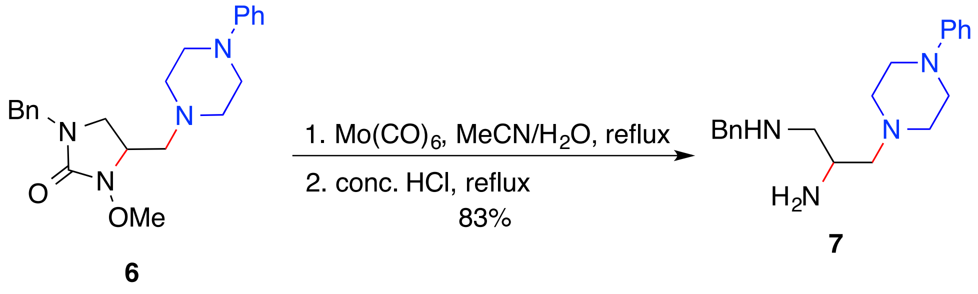


To a solution of **6** (38 mg, 0.1 mmol) in $CH_3CN$-$H_2O$ (2.2 mL, 10:1) at room temperature was added $Mo(CO)_6$ (31.7 mg, 0.12 mmol). The resulting mixture was heated and refluxed for 2 h. The reaction mixture was cooled down to room temperature and concentrated uder reduced pressure. To the resulting crude mixture, was added an aqueous solution of HCl (37% in $H_2O$, 5 mL) was added. The resulting mixture was heated and refluxed for 2 days. The mixture was basified to pH = 12 with the addition of NaOH and then was extracted with EtOAc for three times. The combined organic solvent was removed under reduced pressure. Purification by column chromatography (10% MeOH in $CH_2Cl_2$ containing 2% ammonia in $H_2O$) gave $N^1$-benzyl-3-(4'-phenylpiperazin-1'-yl)propane-1,2-diamine **7** as a colorless oil (27 mg, 83% yield); $R_f$ = 0.38 (10% MeOH in $CH_2Cl_2$ containing 1% ammonia in $H_2O$); $^1$H NMR (400 MHz, $CDCl_3$): δ 7.30 (d, $J$ = 4.4 Hz, 4H), 7.28–7.20 (m, 3H), 6.91 (d, $J$ = 7.6 Hz, 2H), 6.83 (t, $J$ = 7.6 Hz, 1H), 3.79 (s, 2H), 3.25–3.05 (m, 5H), 2.72–2.60 (m, 3H), 2.56–2.42 (m, 3H), 2.34–2.24 (m, 2H); $^{13}$C NMR (125 MHz, $CDCl_3$): δ 151.3, 140.5, 129.1, 128.4, 128.1, 126.9, 119.6, 116.0, 63.8, 54.6, 54.3, 53.6, 49.2, 47.9; IR (neat): 3286, 3058, 2924, 2818, 1599, 1495, 1453, 1233, 757, 696 $cm^{-1}$; HRMS (m/z) Calcd for ($C_{21}H_{29}N_4$) ($[M+H]^+$): 325.2387; found: 325.2386.

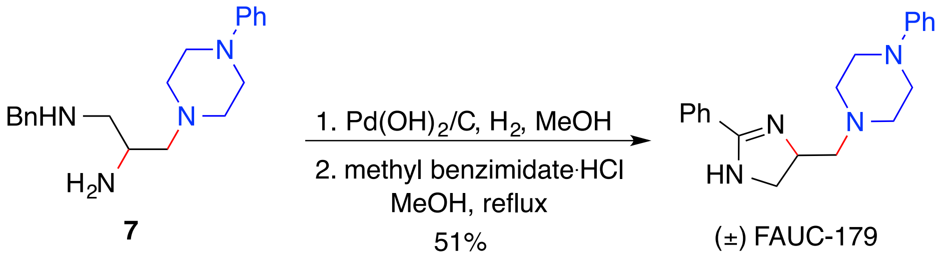


To a 25 mL round-bottomed flask was added 20% $Pd(OH)_2/C$ (50 mg), diamine **7** (26 mg, 0.08 mmol) and MeOH (5 mL) under nitrogen. An atmosphere of hydrogen was introduced by briefly evacuating the flask, then flushing with pure hydrogen (1 atm, hydrogen balloon). The mixture was stirred at room temperature overnight under $H_2$ atmosphere. The hydrogen atmosphere was then removed under vacuum, and the flask was refilled with nitrogen. The mixture was filtered through a pale of Celite and the filtrate was concentrated under reduced pressure. To the crude mixture, was added MeOH (5 mL) and methylbenzimidate hydrochloride (20 mg, 0.12 mmol) at room temperature. The resulting mixture was heated and refluxed for 1 h. Then MeOH was removed under reduced pressure. To the residue, was added EtOAc (10 mL) and a saturated aqueous solution of $Na_2CO_3$ (10 mL). The organic layer was separated and the aqueous layer was extracted with EtOAc (10 mL). The combined organic solvent was removed under reduced pressure. Purification by column chromatography (100% EtOAc containing 5% TEA) gave (±)-FAUC-179 as a white solid (13 mg, 51% yield). $^1$H NMR (400 MHz, $CD_3OD$): δ 7.89 (d, $J$ = 7.6 Hz, 2H), 7.80 (t, $J$ = 7.6 Hz, 1H), 7.66 (t, $J$ = 8.0 Hz, 2H), 7.24 (t, $J$ = 8.0 Hz, 2H), 6.98 (d, $J$ = 8.4 Hz, 2H), 6.85 (t, $J$ = 7.2 Hz, 1H), 4.77–4.65 (m, 1H), 4.21 (t, $J$ = 11.6 Hz, 1H), 3.90 (dd, $J$ = 11.6, 7.6 Hz, 1H), 3.28–3.18 (m, 4H), 2.94–2.67 (m, 6H). Spectroscopic data was identical to that reported previously.[5]

## IX. References.


1. S. Nicolai, C. Piemontesi and J. Waser, *Angew. Chem. Int. Ed.* **2011**, *50*, 4680.
2. F. C. Sequeira, B. W. Turnpenny and S. R. Chemler, *Angew. Chem. Int. Ed.* **2010**, *49*, 6365.
3. K. Komeyama, K. Takahashi and K. Takaki, *Org. Lett.* **2008**, *10*, 5119.
4. D. J. Wardrop, E. G. Bowen, R. E. Forslund, A. D. Sussman and S. L. Weerasekera, *J. Am. Chem. Soc.* **2010**, *132*, 1188.
5. J. Einsiedel, H. Hubner and P. Gmeiner, *Bioorg. Med. Chem. Lett.* **2001**, *11*, 2533.

## X. NMR Spectra.

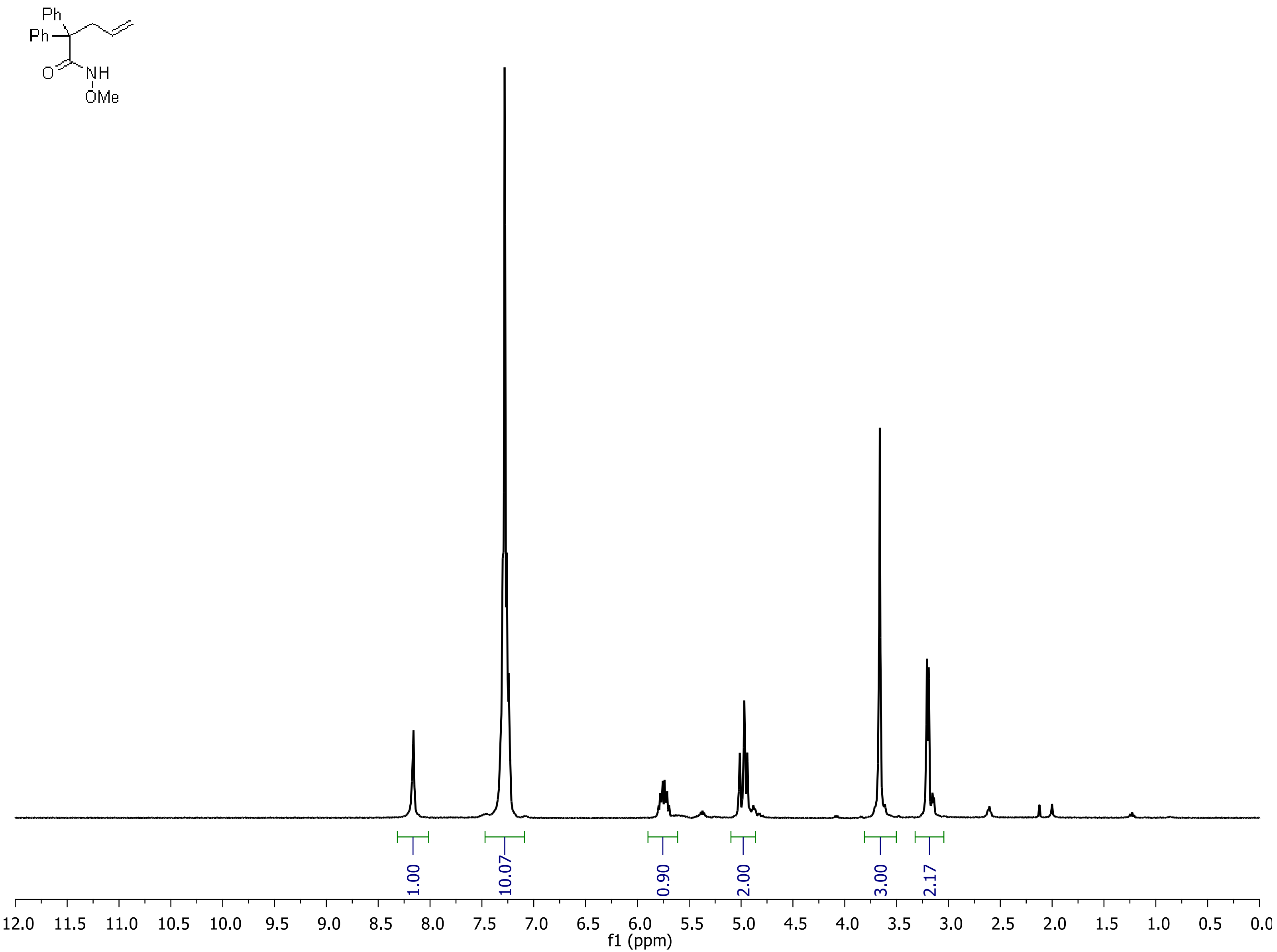

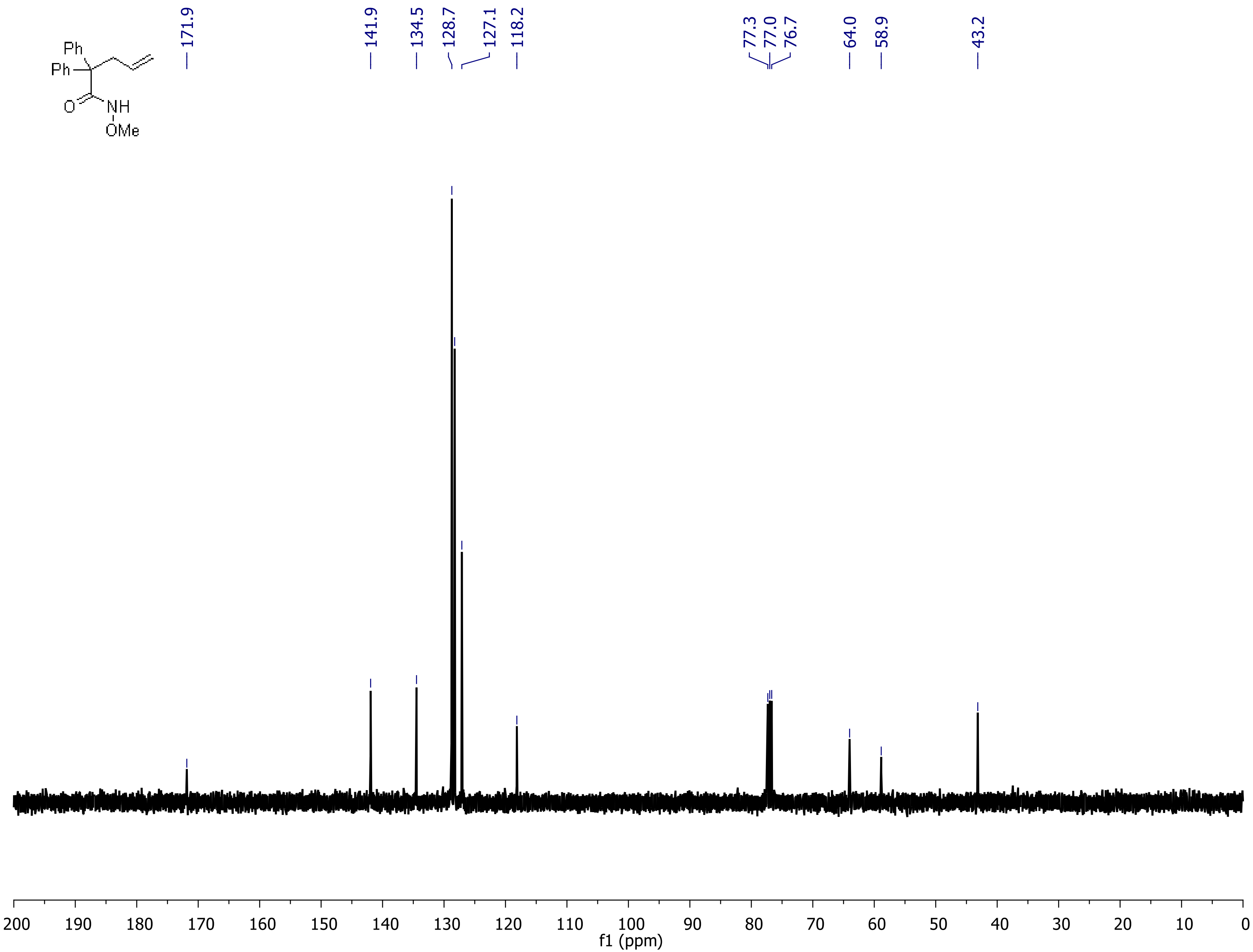
Ph
Ph
O
NH
OMe
171.9
141.9
134.5
128.7
127.1
118.2
77.3
77.0
76.7
64.0
58.9
43.2
200 190 180 170 160 150 140 130 120 110 100 90 80 70 60 50 40 30 20 10 0
f1 (ppm)

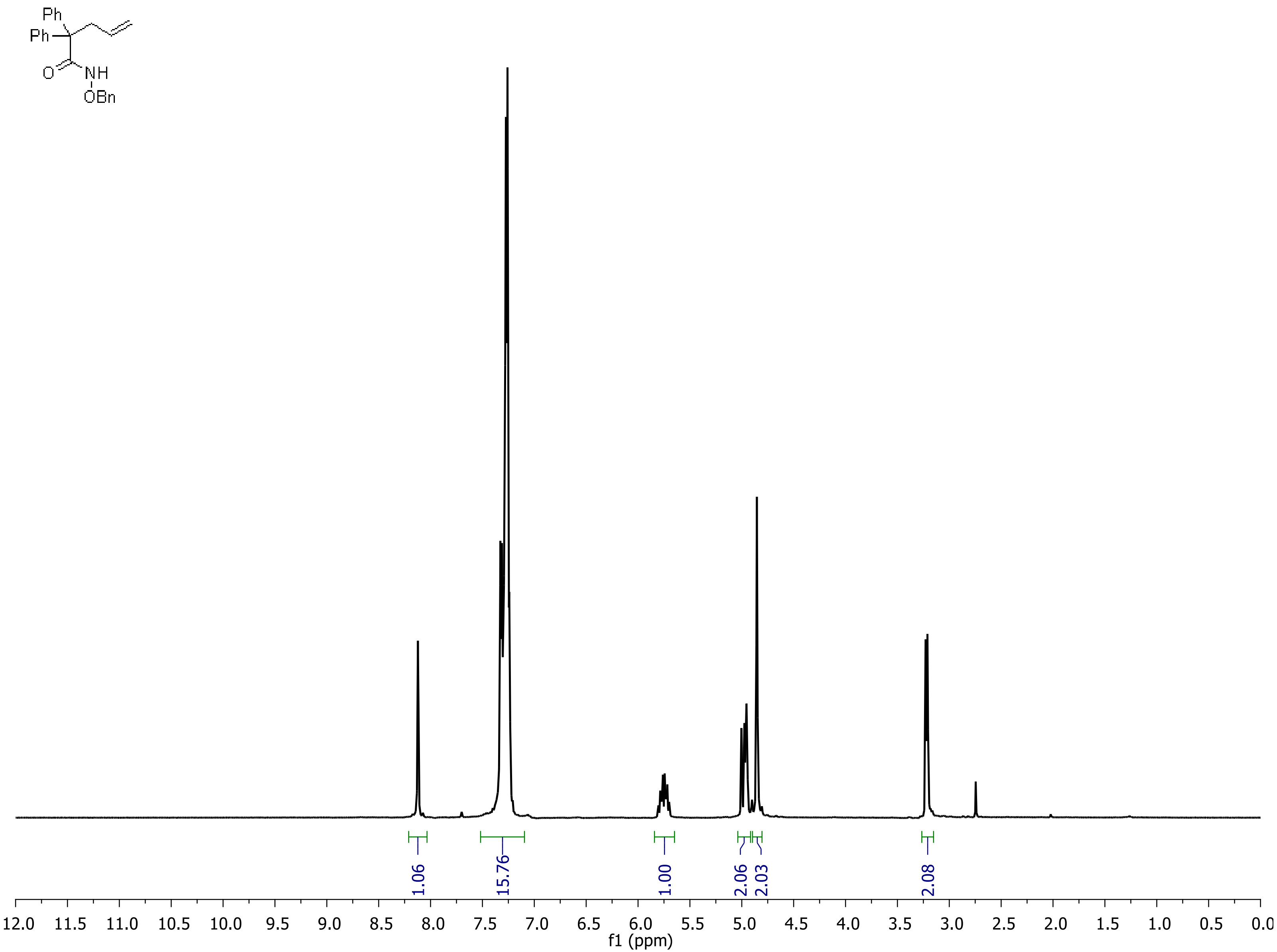

Ph
Ph
O
NH
OBn
1.06
15.76
1.00
2.06
2.03
2.08
12.0 11.5 11.0 10.5 10.0 9.5 9.0 8.5 8.0 7.5 7.0 6.5 6.0 5.5 5.0 4.5 4.0 3.5 3.0 2.5 2.0 1.5 1.0 0.5 0.0
f1 (ppm)

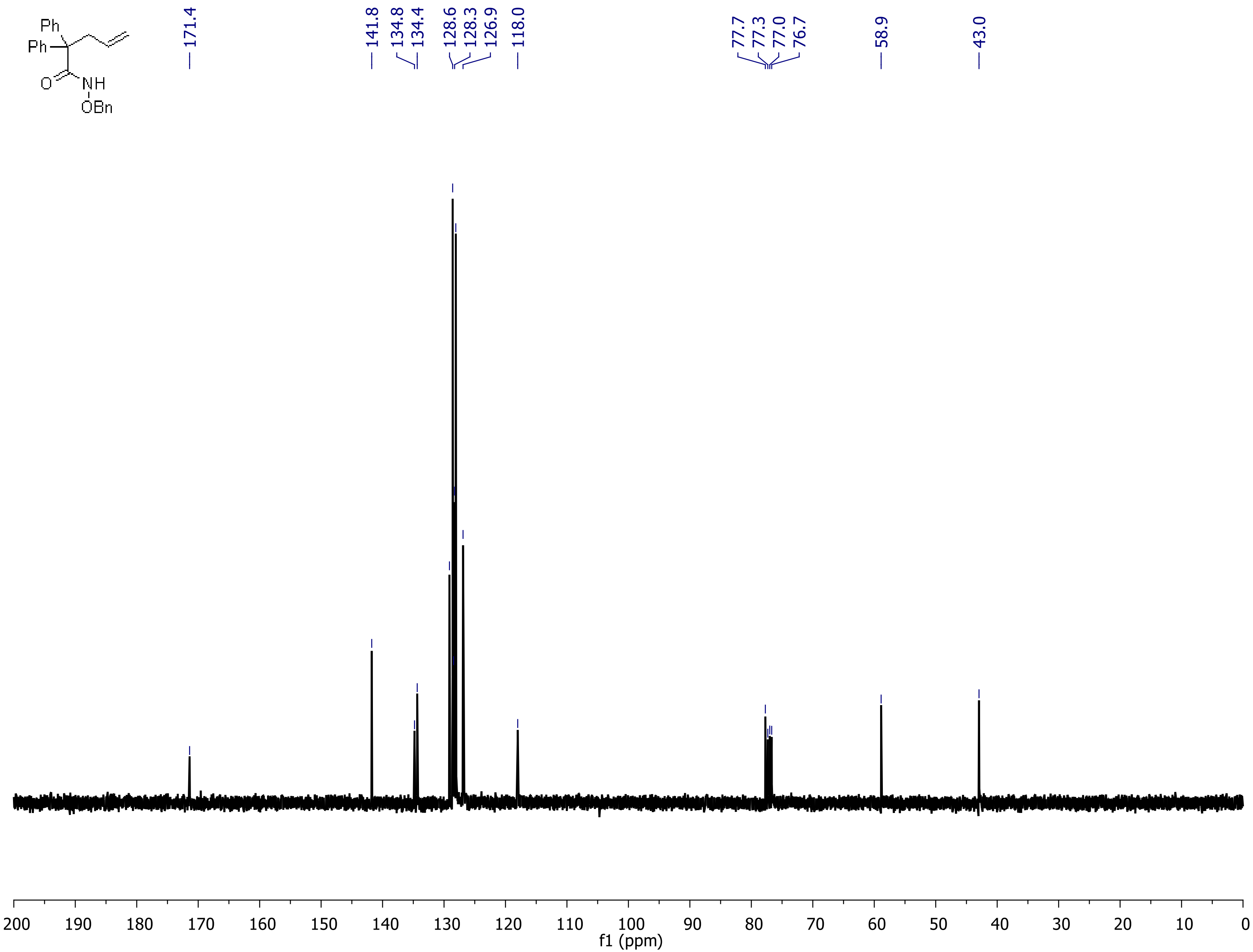
Ph
Ph
O
NH
OBn
171.4
141.8
134.8
134.4
128.6
128.3
126.9
118.0
77.7
77.3
77.0
76.7
58.9
43.0
200
190
180
170
160
150
140
130
120
110
100
90
80
70
60
50
40
30
20
10
0
f1 (ppm)

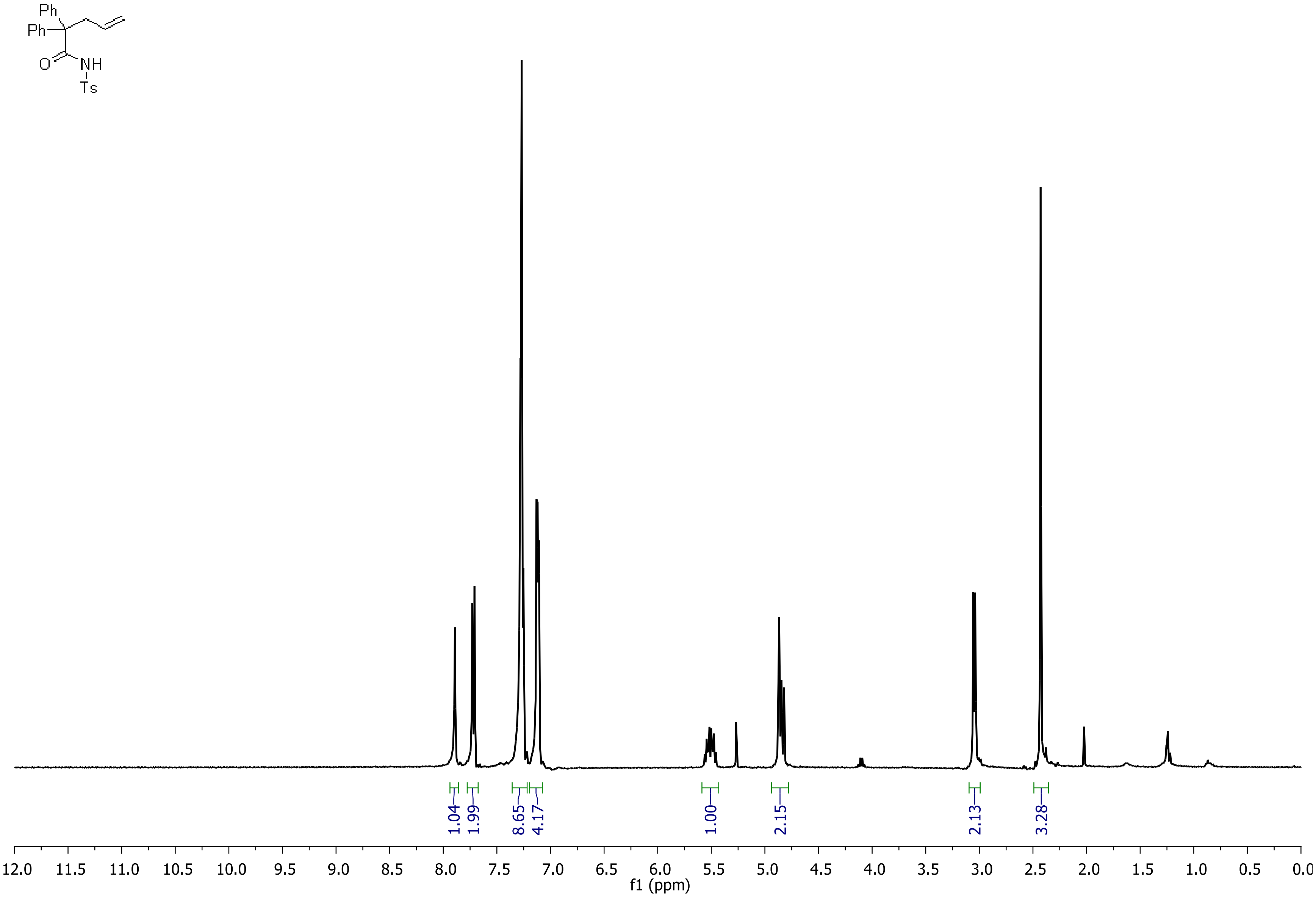
Ph
Ph
O
NH
Ts
1.04
1.99
8.65
4.17
1.00
2.15
2.13
3.28
12.0
11.5
11.0
10.5
10.0
9.5
9.0
8.5
8.0
7.5
7.0
6.5
6.0
5.5
5.0
4.5
4.0
3.5
3.0
2.5
2.0
1.5
1.0
0.5
0.0
f1 (ppm)

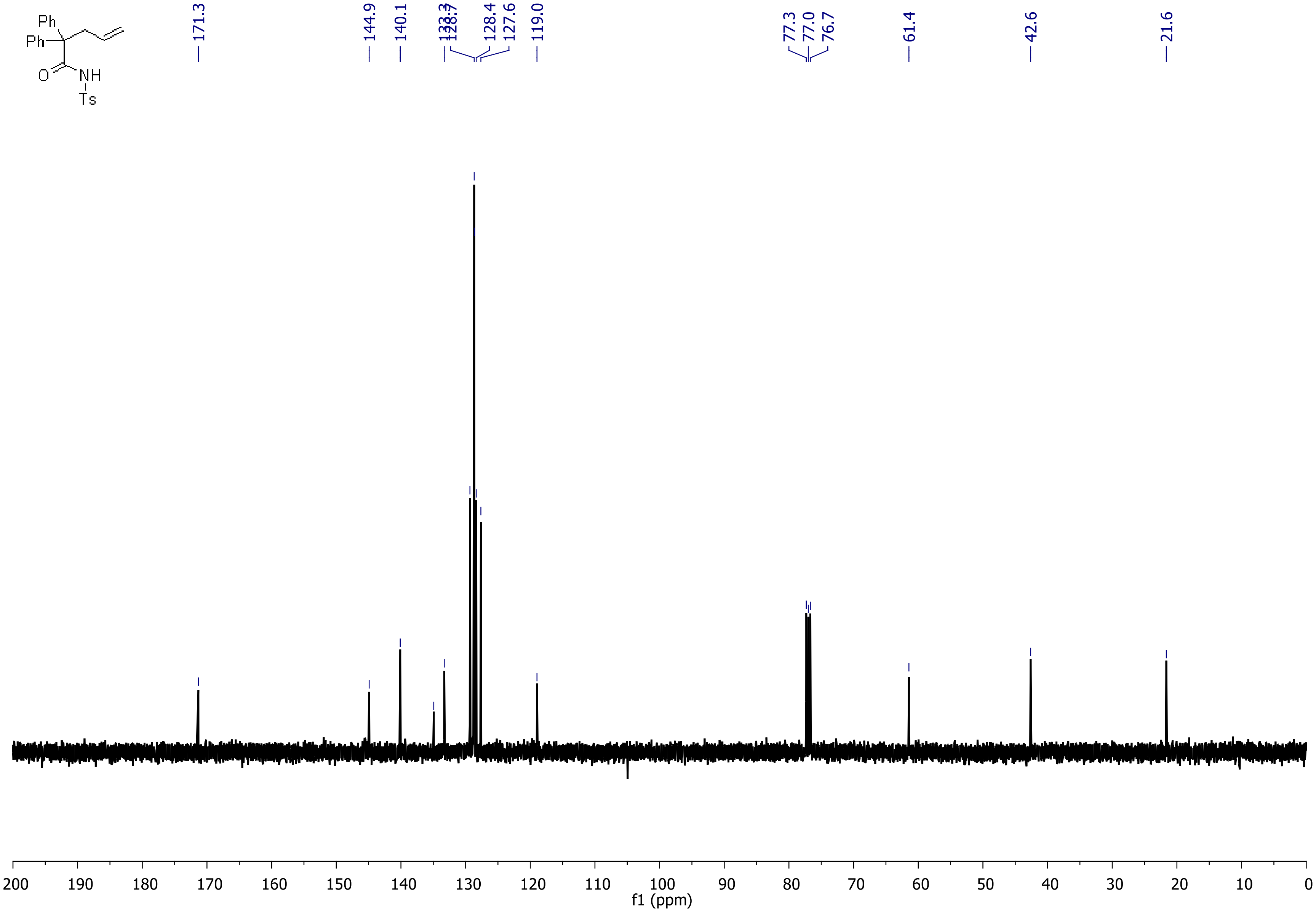

Ph
Ph
O
NH
Ts
171.3
144.9
140.1
128.7
128.4
127.6
119.0
77.3
77.0
76.7
61.4
42.6
21.6
200
190
180
170
160
150
140
130
120
110
100
90
80
70
60
50
40
30
20
10
0
f1 (ppm)

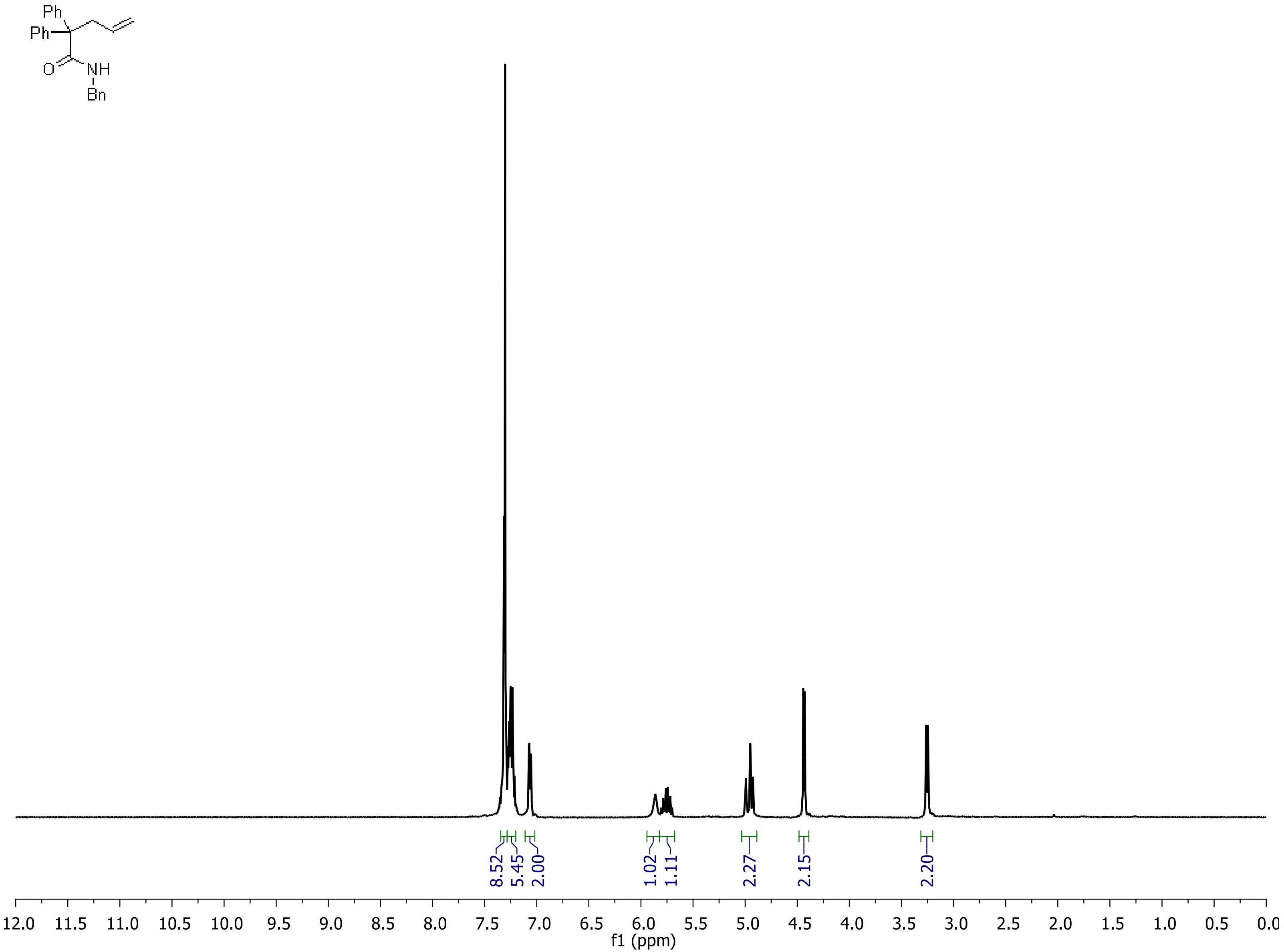
Ph
Ph
O
NH
Bn
8.52
5.45
2.00
1.02
1.11
2.27
2.15
2.20
12.0 11.5 11.0 10.5 10.0 9.5 9.0 8.5 8.0 7.5 7.0 6.5 6.0 5.5 5.0 4.5 4.0 3.5 3.0 2.5 2.0 1.5 1.0 0.5 0.0
f1 (ppm)

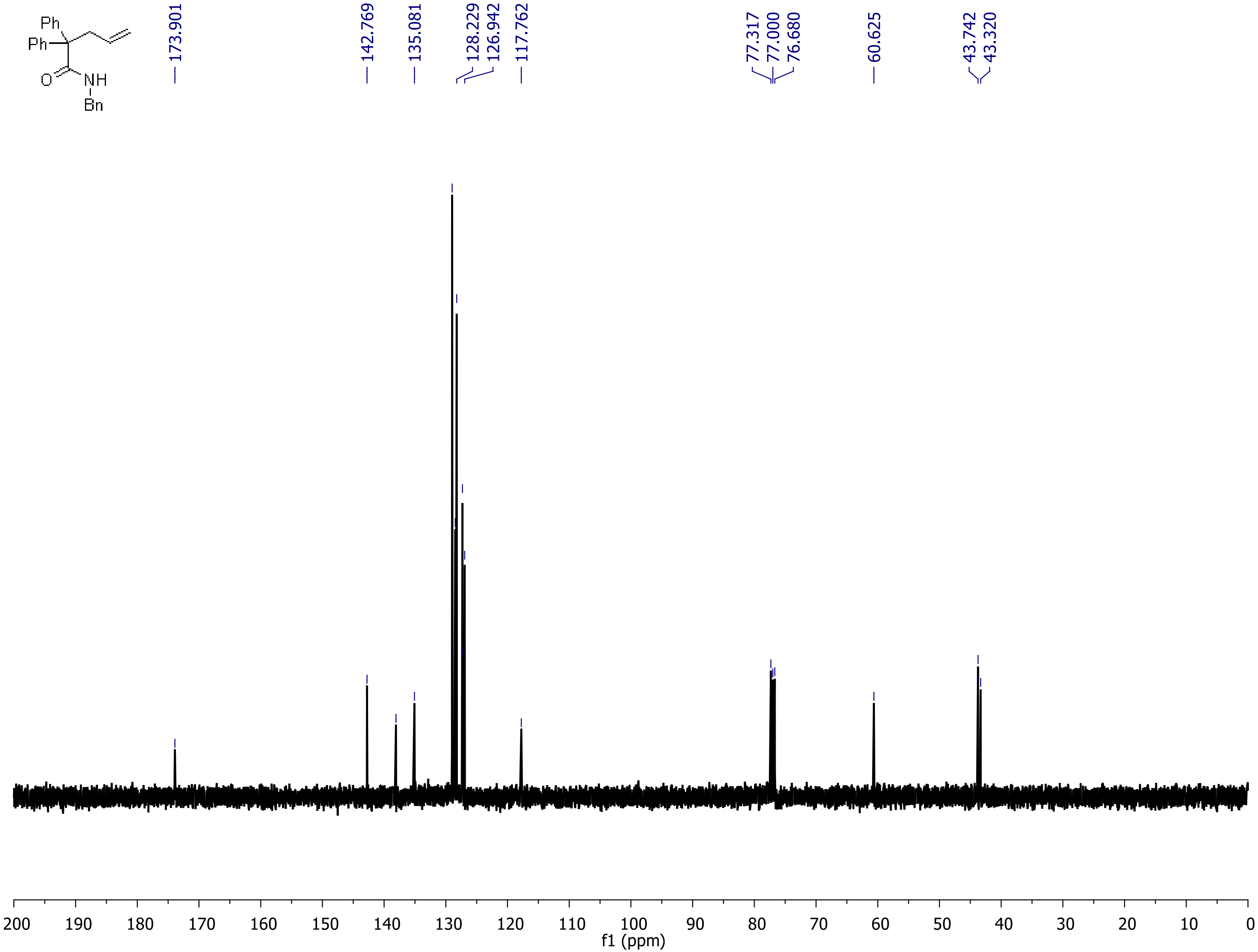
Ph
Ph
O
NH
Bn
173.901
142.769
135.081
128.229
126.942
117.762
77.317
77.000
76.680
60.625
43.742
43.320
200 190 180 170 160 150 140 130 120 110 100 90 80 70 60 50 40 30 20 10 0
f1 (ppm)

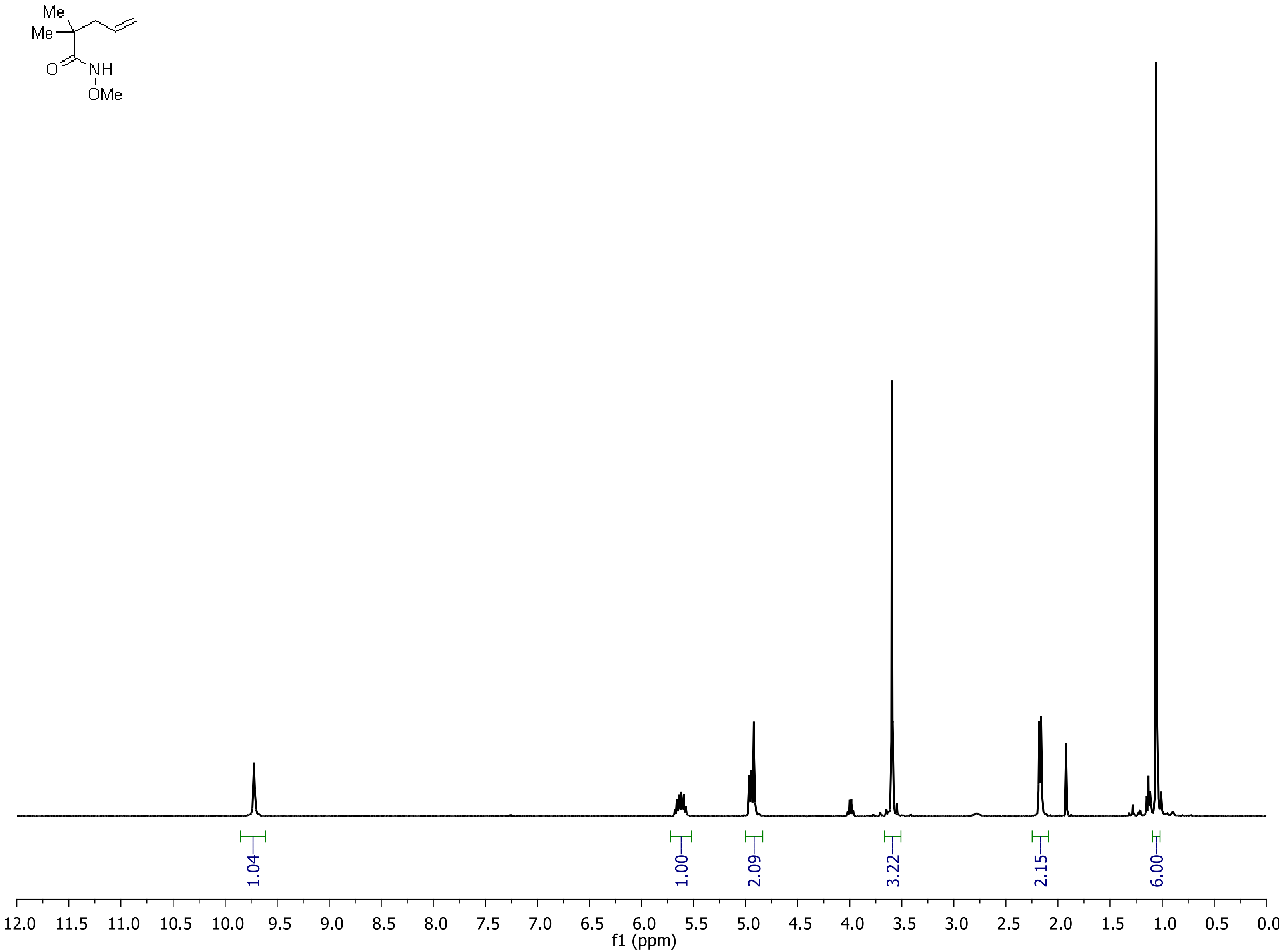
Me
Me
O
NH
OMe
1.04
1.00
2.09
3.22
2.15
6.00
12.0 11.5 11.0 10.5 10.0 9.5 9.0 8.5 8.0 7.5 7.0 6.5 6.0 5.5 5.0 4.5 4.0 3.5 3.0 2.5 2.0 1.5 1.0 0.5 0.0
f1 (ppm)

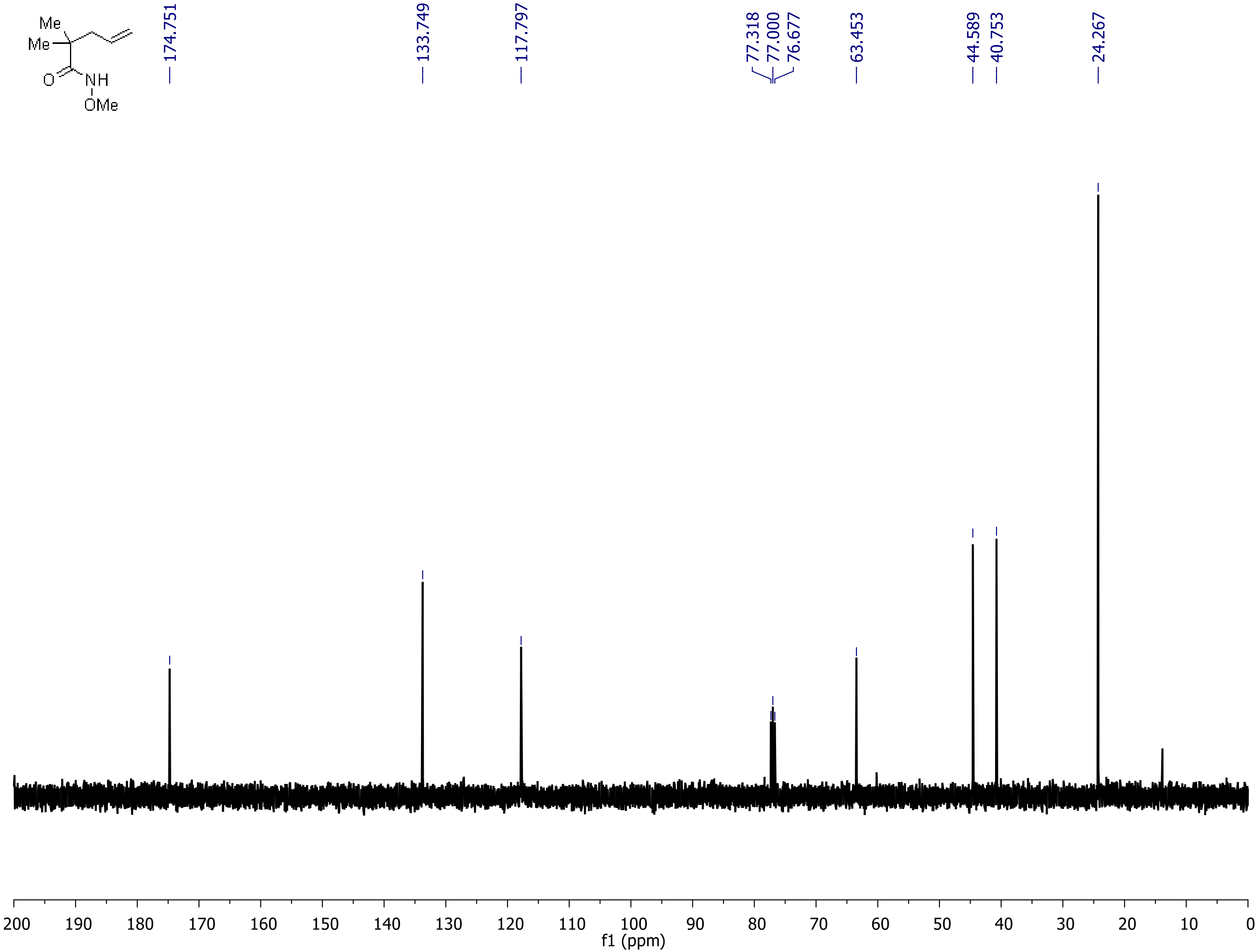
Me
Me
O
NH
OMe
174.751
133.749
117.797
77.318
77.000
76.677
63.453
44.589
40.753
24.267
200 190 180 170 160 150 140 130 120 110 100 90 80 70 60 50 40 30 20 10 0
f1 (ppm)

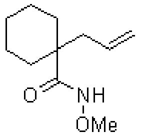

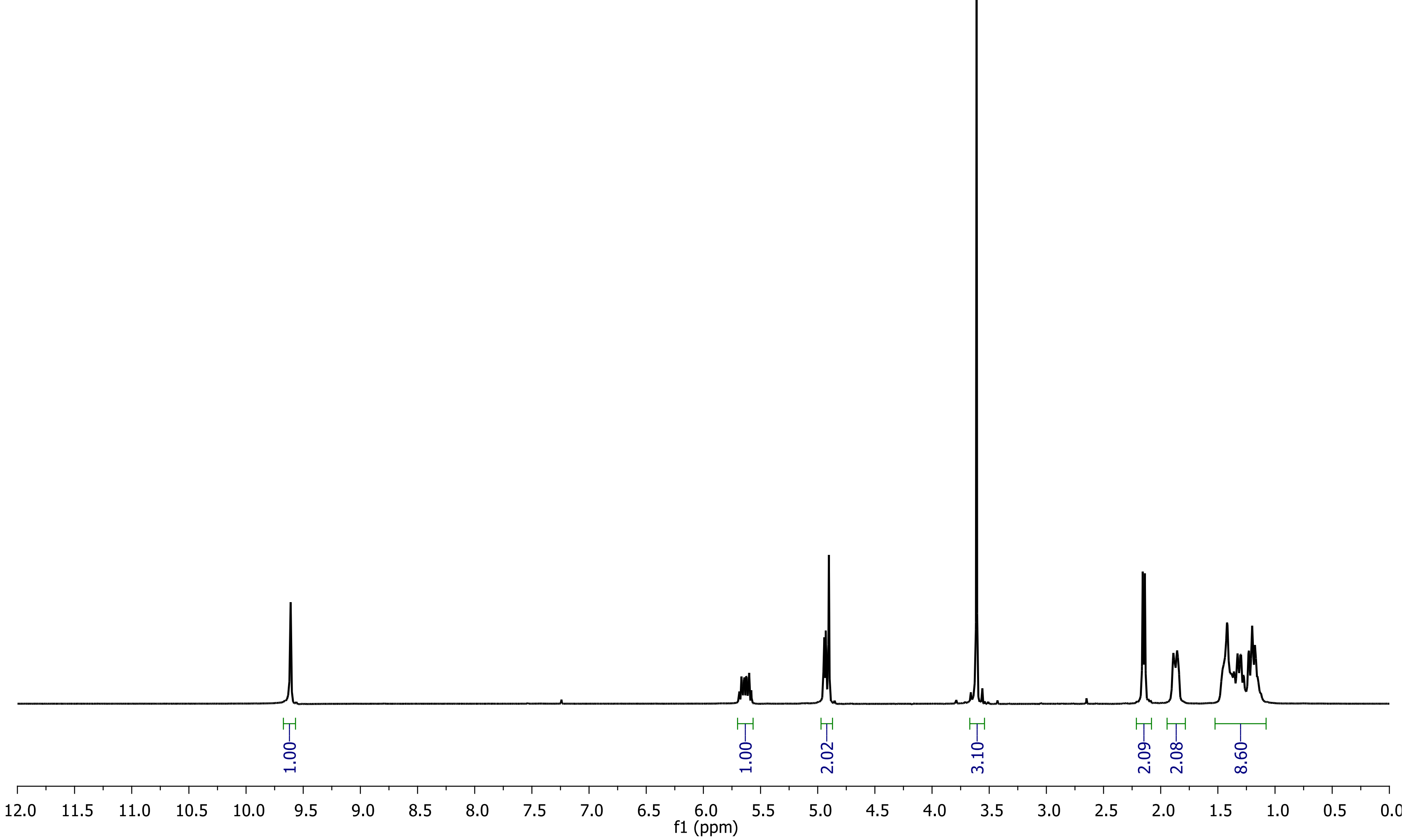

1.00
1.00
2.02
3.10
2.09
2.08
8.60
12.0 11.5 11.0 10.5 10.0 9.5 9.0 8.5 8.0 7.5 7.0 6.5 6.0 5.5 5.0 4.5 4.0 3.5 3.0 2.5 2.0 1.5 1.0 0.5 0.0
f1 (ppm)

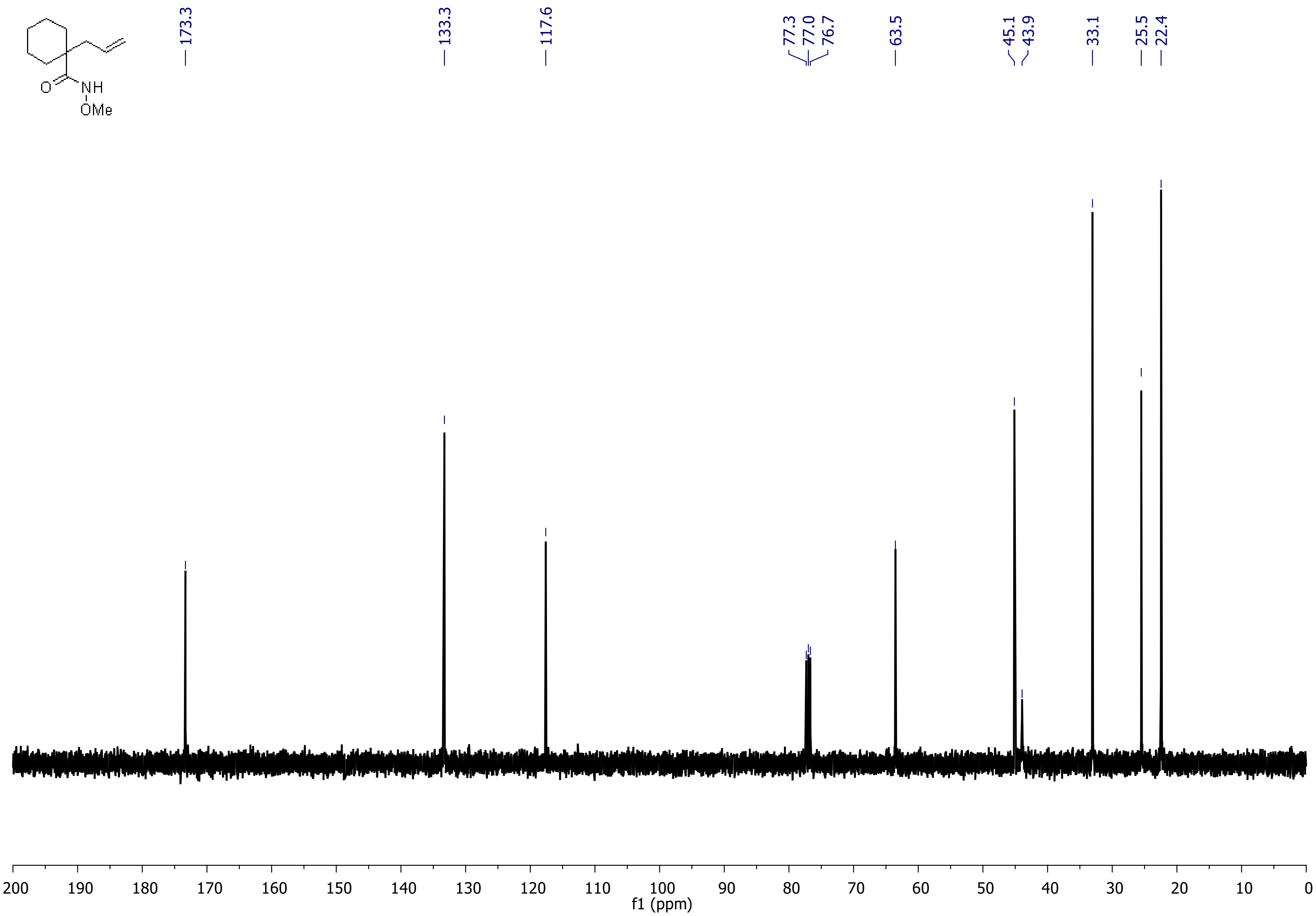
NH
OMe
O
173.3
133.3
117.6
77.3
77.0
76.7
63.5
45.1
43.9
33.1
25.5
22.4
200 190 180 170 160 150 140 130 120 110 100 90 80 70 60 50 40 30 20 10 0
f1 (ppm)

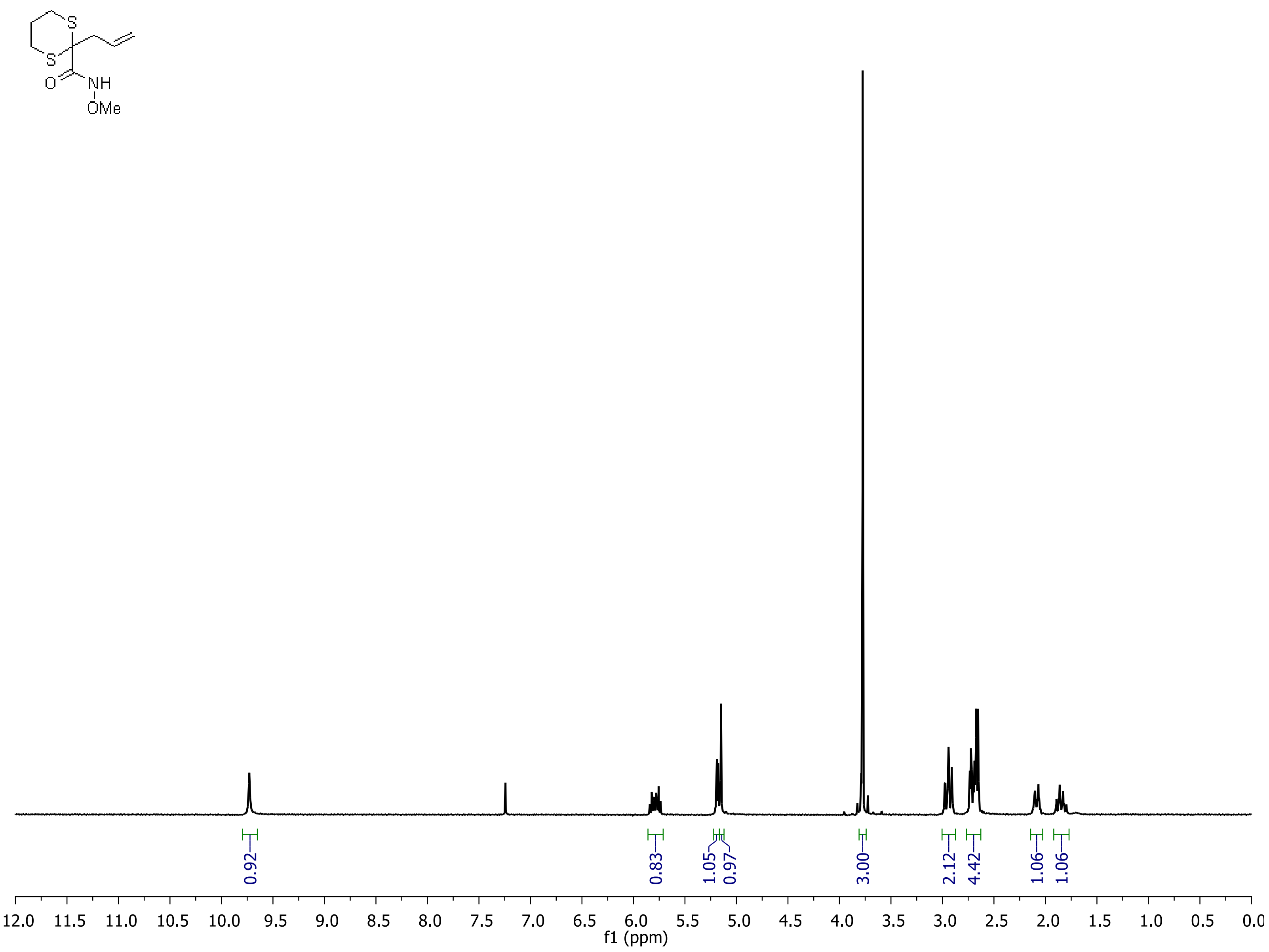

S
S
O
NH
OMe
0.92
0.83
1.05
0.97
3.00
2.12
4.42
1.06
1.06
12.0 11.5 11.0 10.5 10.0 9.5 9.0 8.5 8.0 7.5 7.0 6.5 6.0 5.5 5.0 4.5 4.0 3.5 3.0 2.5 2.0 1.5 1.0 0.5 0.0
f1 (ppm)

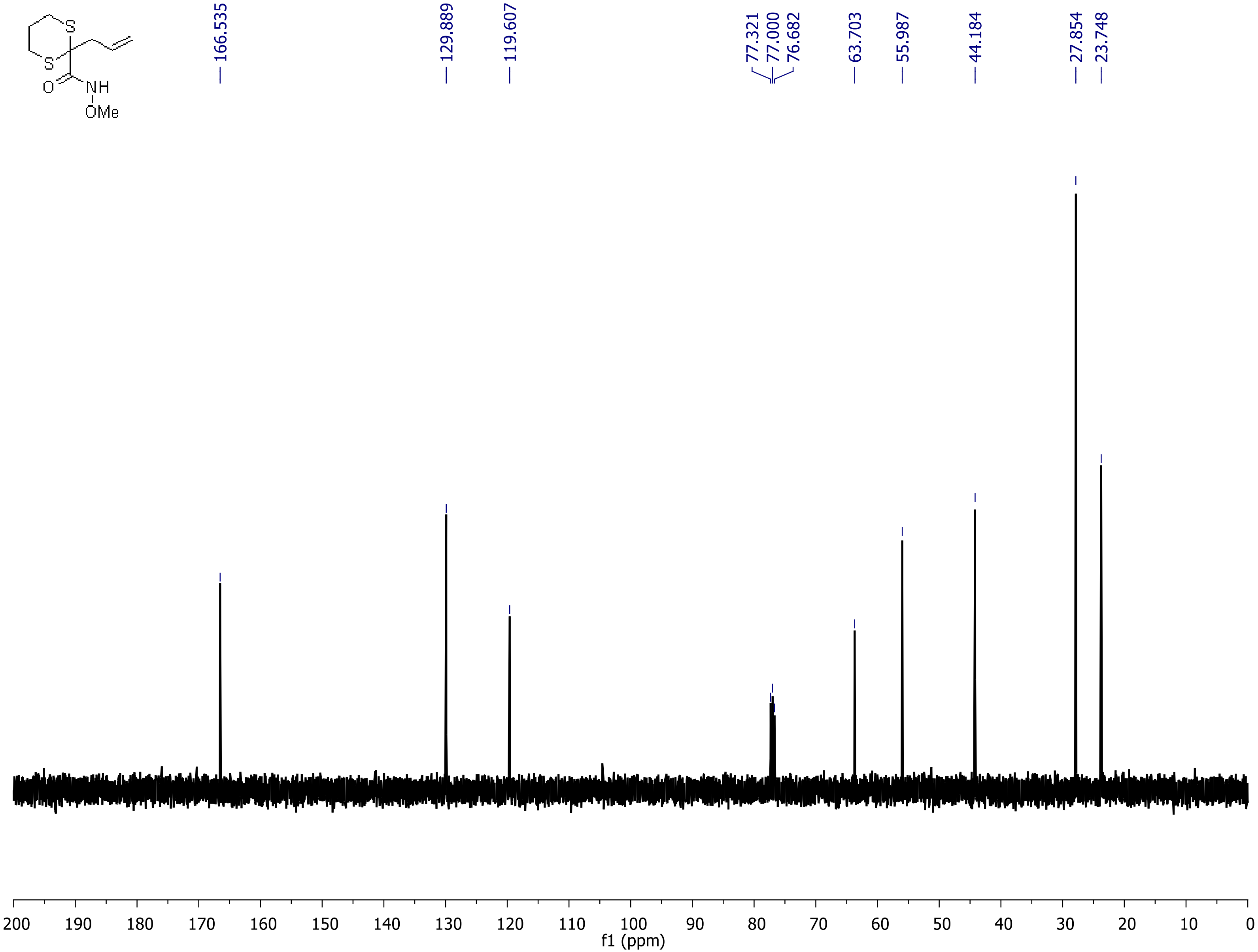
S
S
O
NH
OMe
166.535
129.889
119.607
77.321
77.000
76.682
63.703
55.987
44.184
27.854
23.748
200
190
180
170
160
150
140
130
120
110
100
90
80
70
60
50
40
30
20
10
0
f1 (ppm)

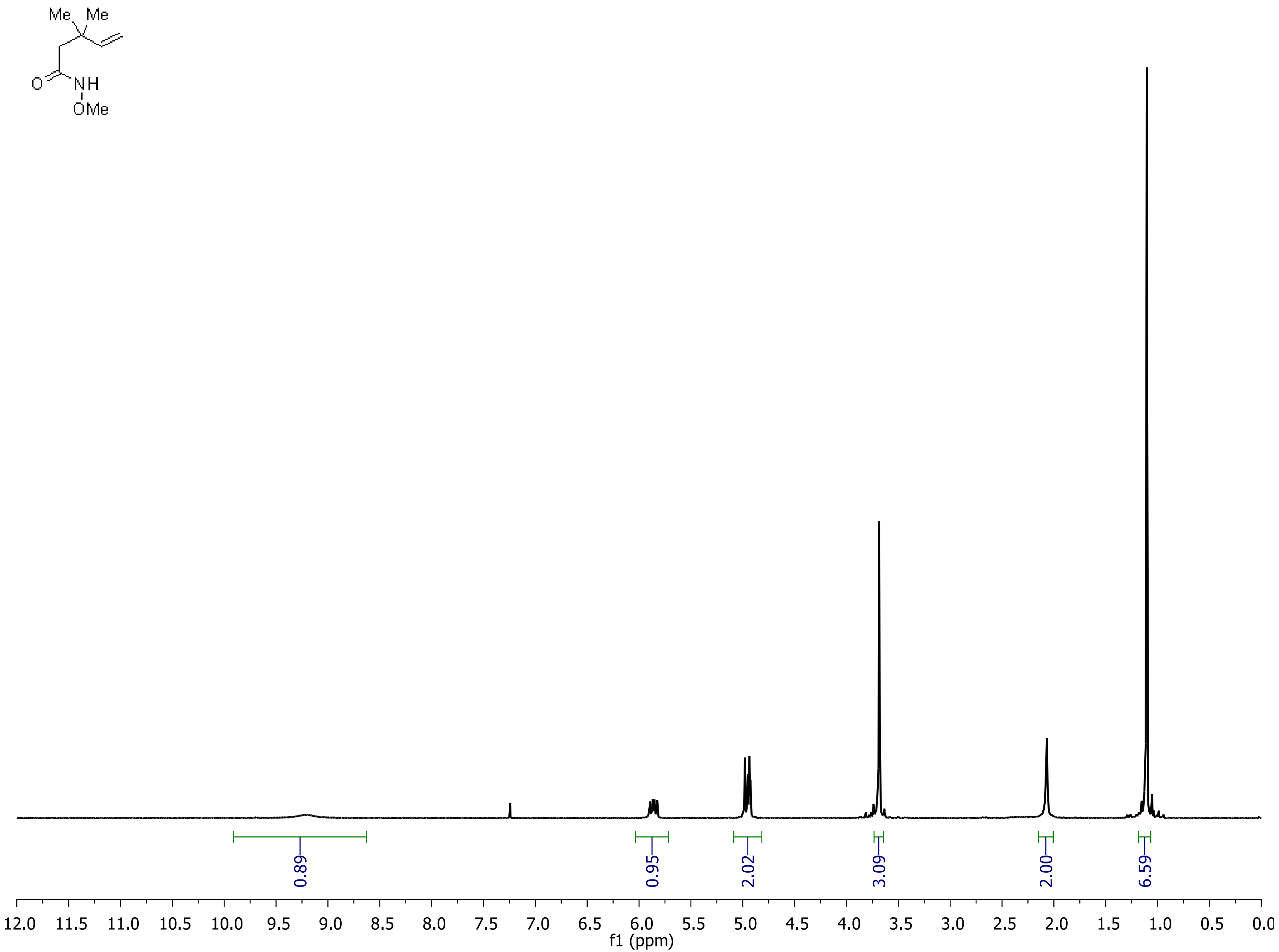
Me
Me
O
NH
OMe
0.89
0.95
2.02
3.09
2.00
6.59
12.0
11.5
11.0
10.5
10.0
9.5
9.0
8.5
8.0
7.5
7.0
6.5
6.0
5.5
5.0
4.5
4.0
3.5
3.0
2.5
2.0
1.5
1.0
0.5
0.0
f1 (ppm)

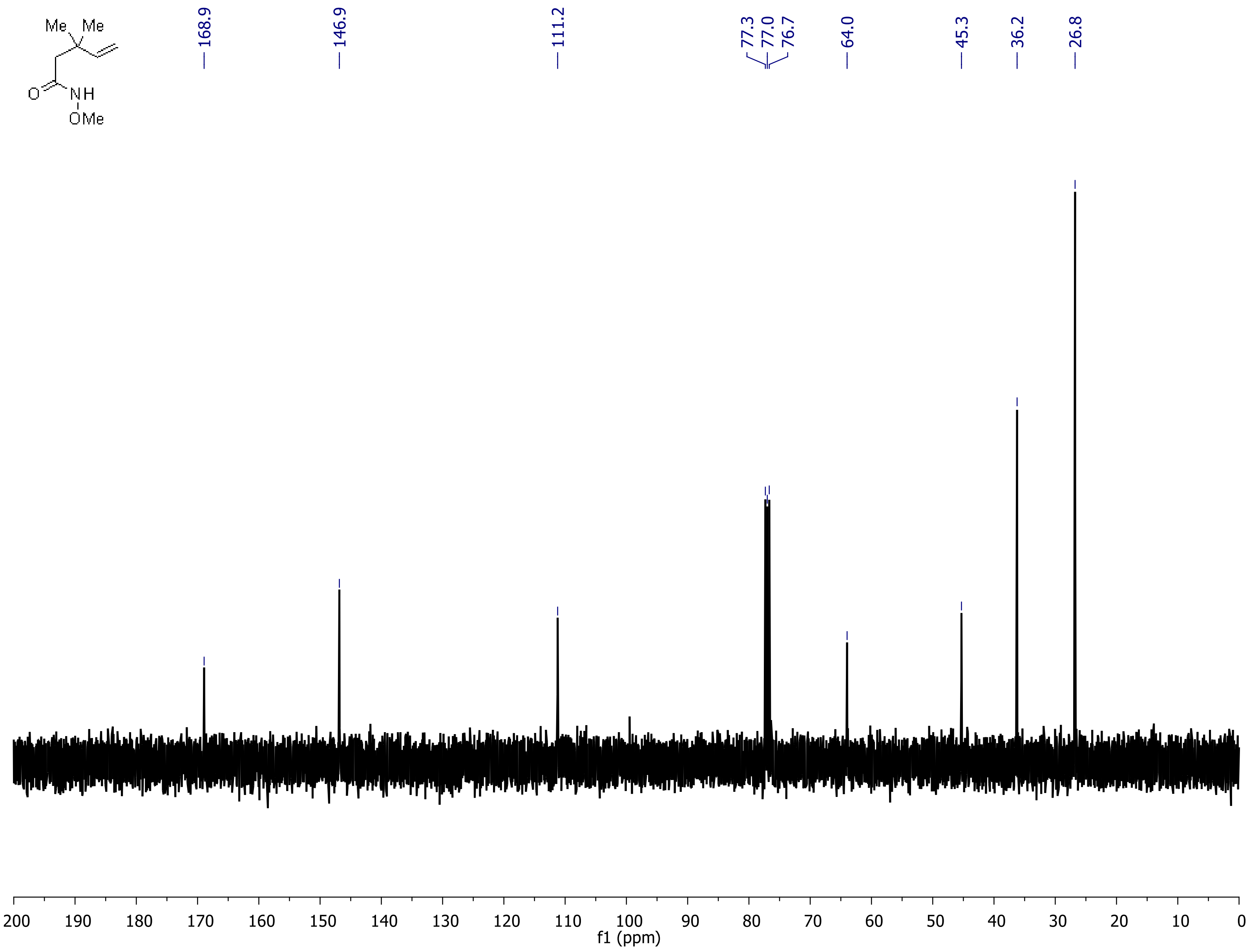

Me
Me
O
NH
OMe
168.9
146.9
111.2
77.3
77.0
76.7
64.0
45.3
36.2
26.8
200
190
180
170
160
150
140
130
120
110
100
90
80
70
60
50
40
30
20
10
0
f1 (ppm)

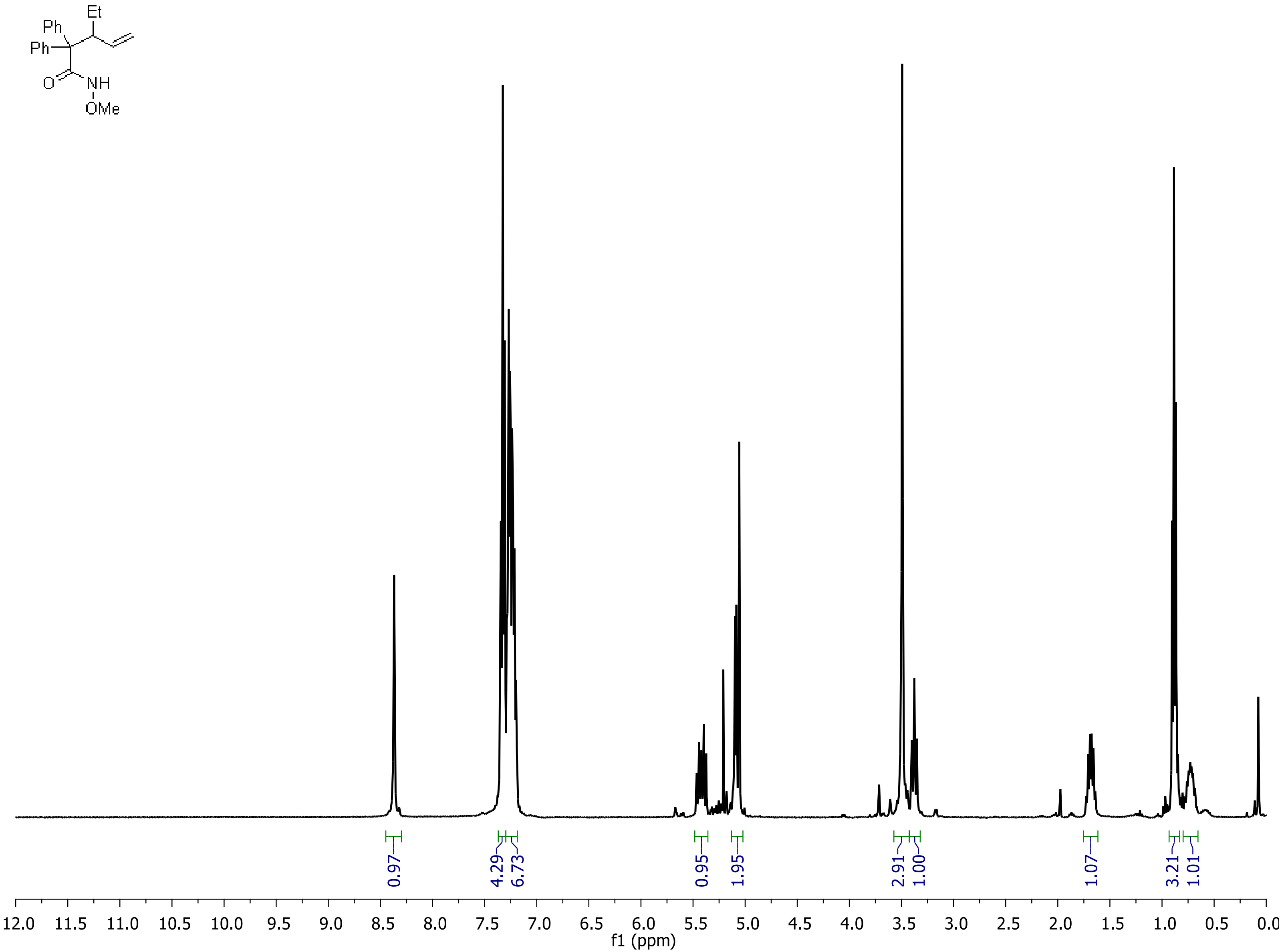

Et
Ph
Ph
O
NH
OMe
0.97
4.29
6.73
0.95
1.95
2.91
1.00
1.07
3.21
1.01
12.0 11.5 11.0 10.5 10.0 9.5 9.0 8.5 8.0 7.5 7.0 6.5 6.0 5.5 5.0 4.5 4.0 3.5 3.0 2.5 2.0 1.5 1.0 0.5 0.0
f1 (ppm)

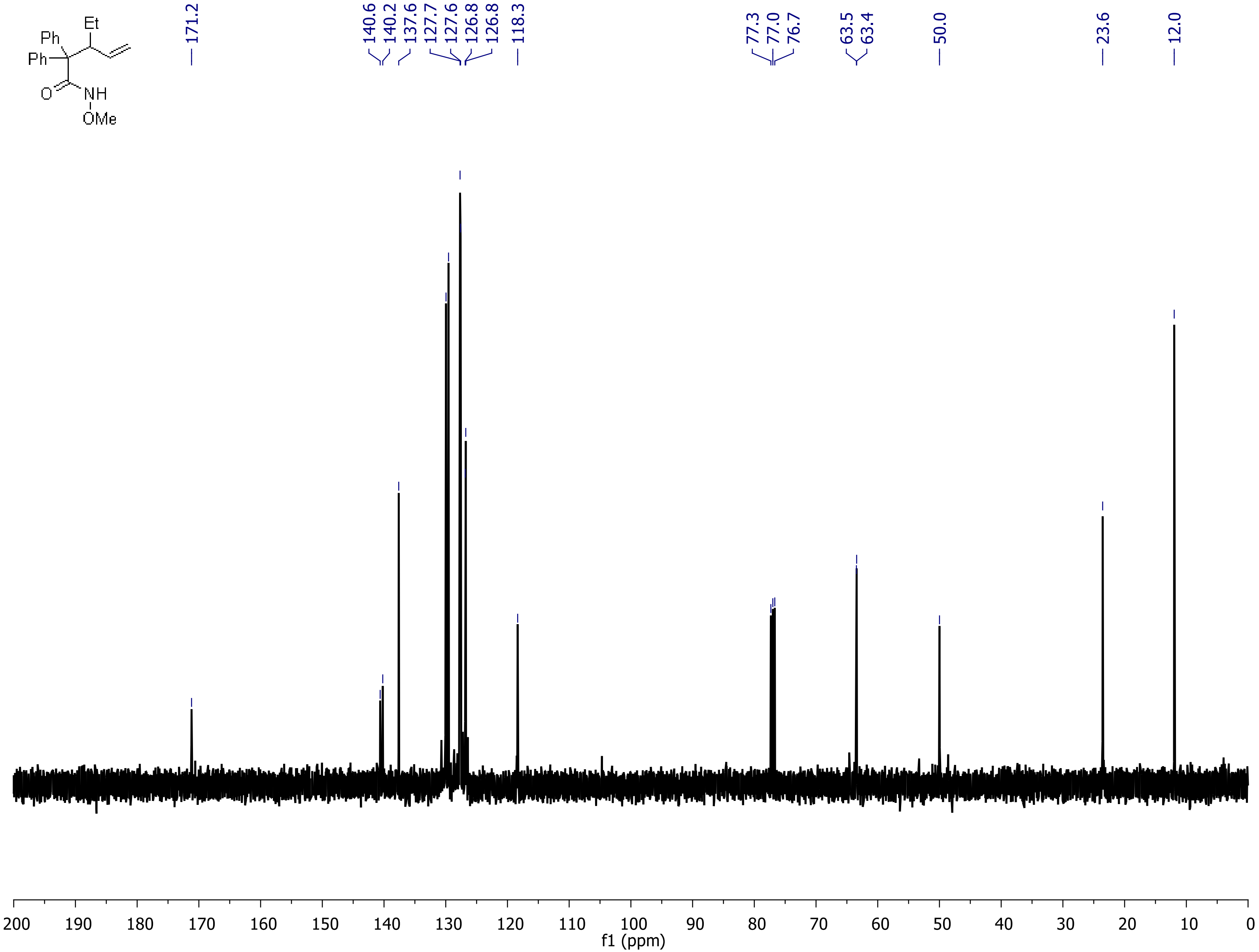
Ph
Ph
Et
O
NH
OMe
171.2
140.6
140.2
137.6
127.7
127.6
126.8
126.8
118.3
77.3
77.0
76.7
63.5
63.4
50.0
23.6
12.0
200 190 180 170 160 150 140 130 120 110 100 90 80 70 60 50 40 30 20 10 0
f1 (ppm)

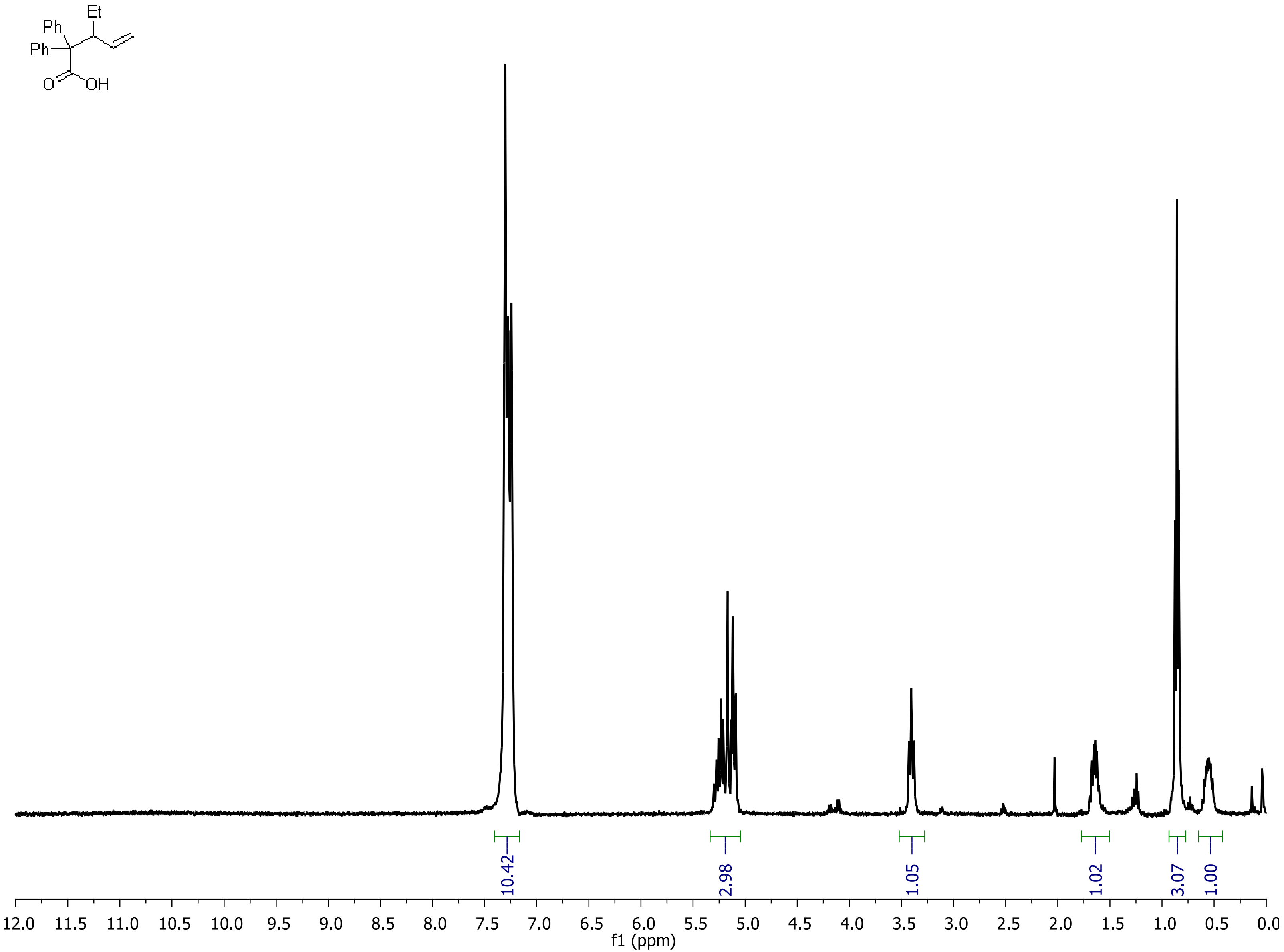
Et
Ph
Ph
O
OH
10.42
2.98
1.05
1.02
3.07
1.00
12.0 11.5 11.0 10.5 10.0 9.5 9.0 8.5 8.0 7.5 7.0 6.5 6.0 5.5 5.0 4.5 4.0 3.5 3.0 2.5 2.0 1.5 1.0 0.5 0.0
f1 (ppm)

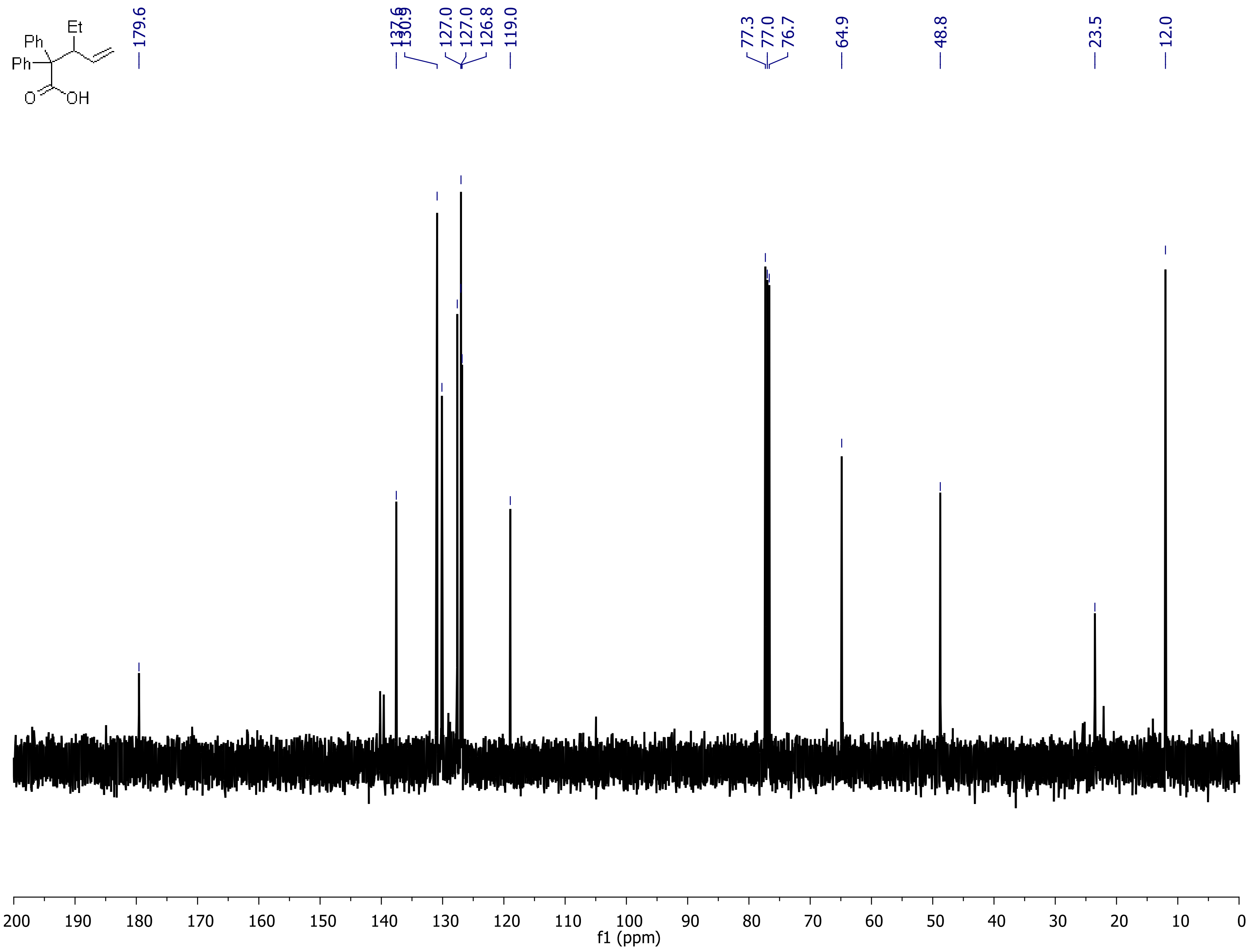

Ph
Ph
Et
O
OH
179.6
137.6
130.9
127.0
127.0
126.8
119.0
77.3
77.0
76.7
64.9
48.8
23.5
12.0
200
190
180
170
160
150
140
130
120
110
100
90
80
70
60
50
40
30
20
10
0
f1 (ppm)

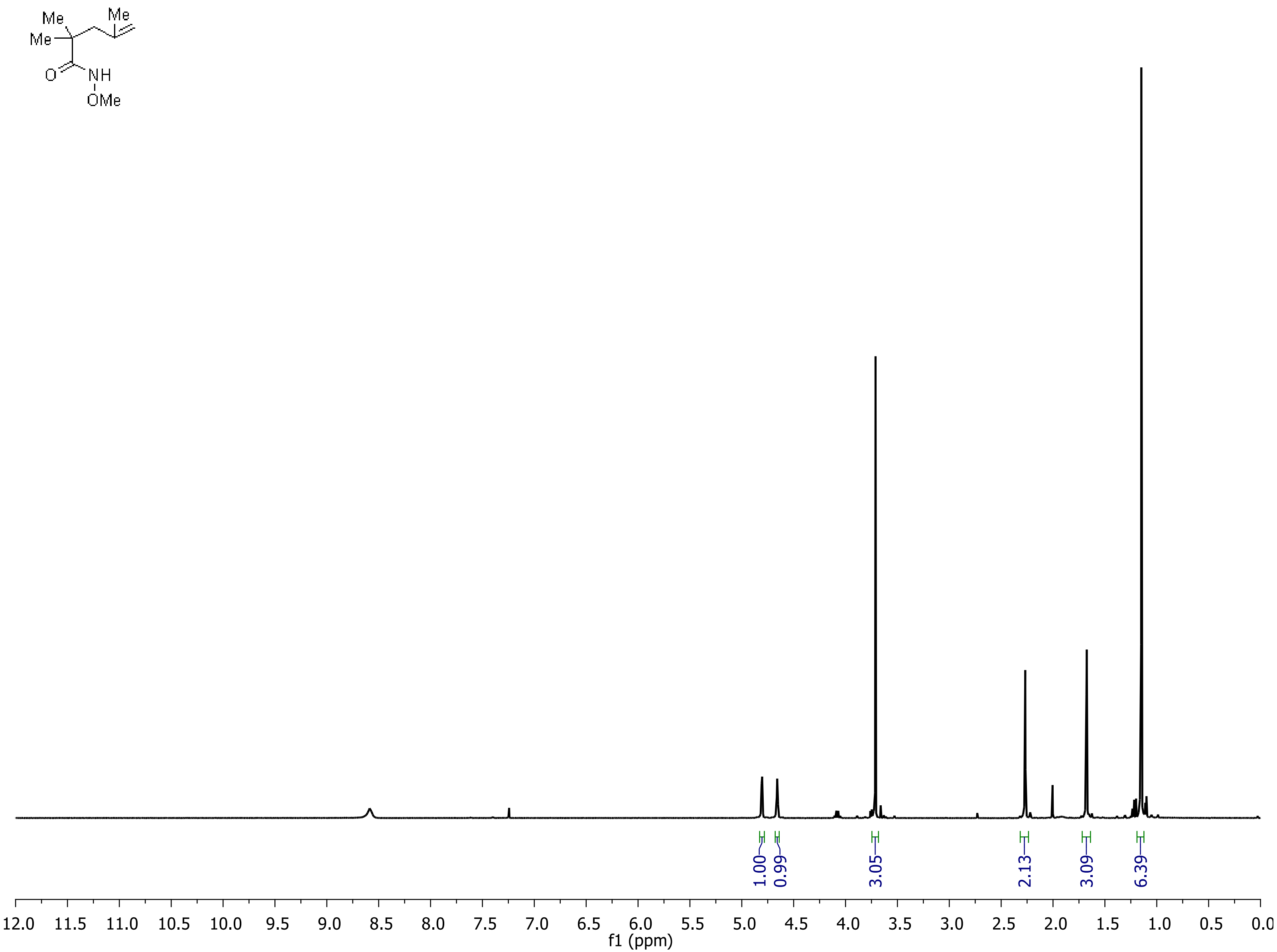
Me
Me
Me
O
NH
OMe
1.00
0.99
3.05
2.13
3.09
6.39
12.0
11.5
11.0
10.5
10.0
9.5
9.0
8.5
8.0
7.5
7.0
6.5
6.0
5.5
5.0
4.5
4.0
3.5
3.0
2.5
2.0
1.5
1.0
0.5
0.0
f1 (ppm)

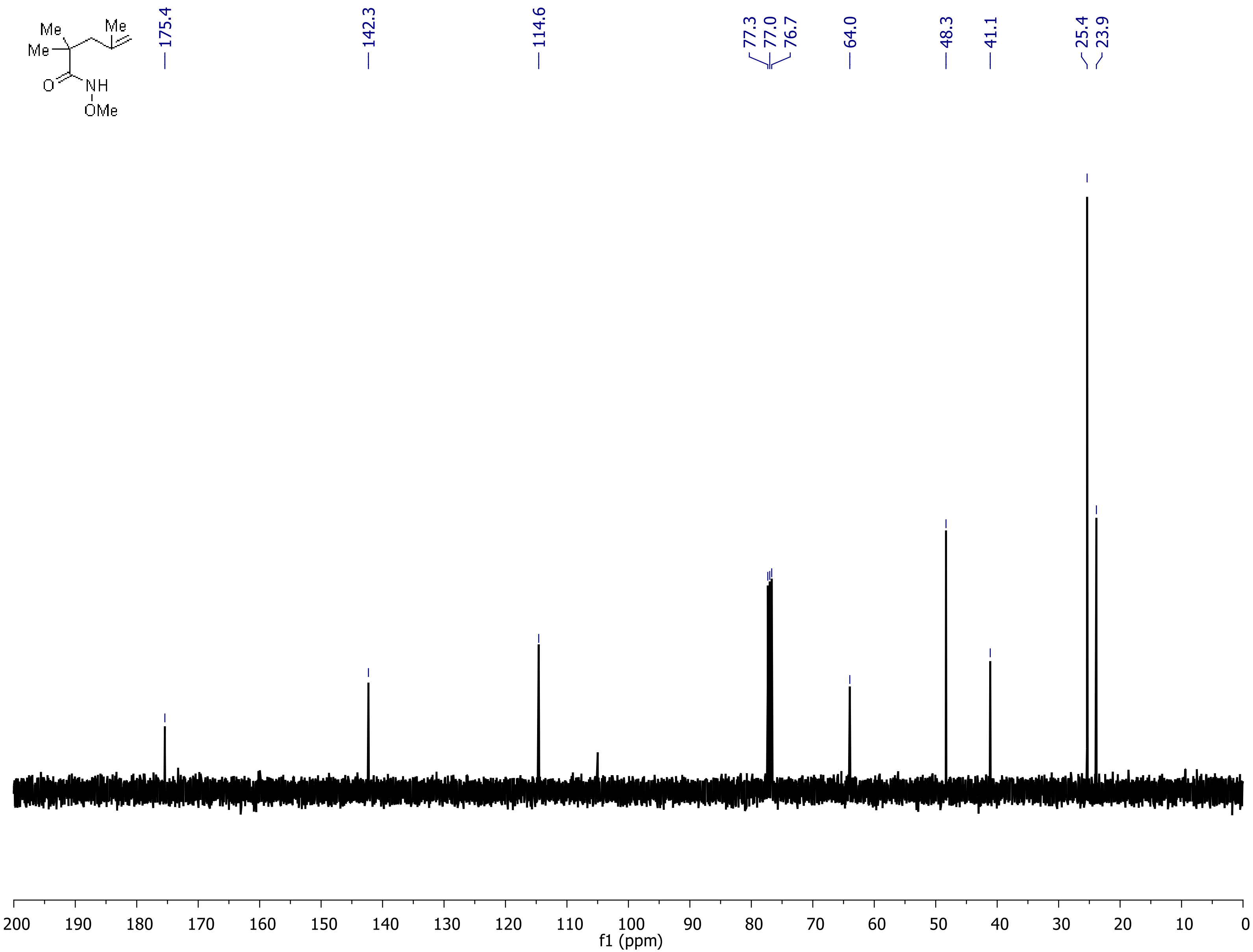
Me
Me
Me
O
NH
OMe
175.4
142.3
114.6
77.3
77.0
76.7
64.0
48.3
41.1
25.4
23.9
200
190
180
170
160
150
140
130
120
110
100
90
80
70
60
50
40
30
20
10
0
f1 (ppm)

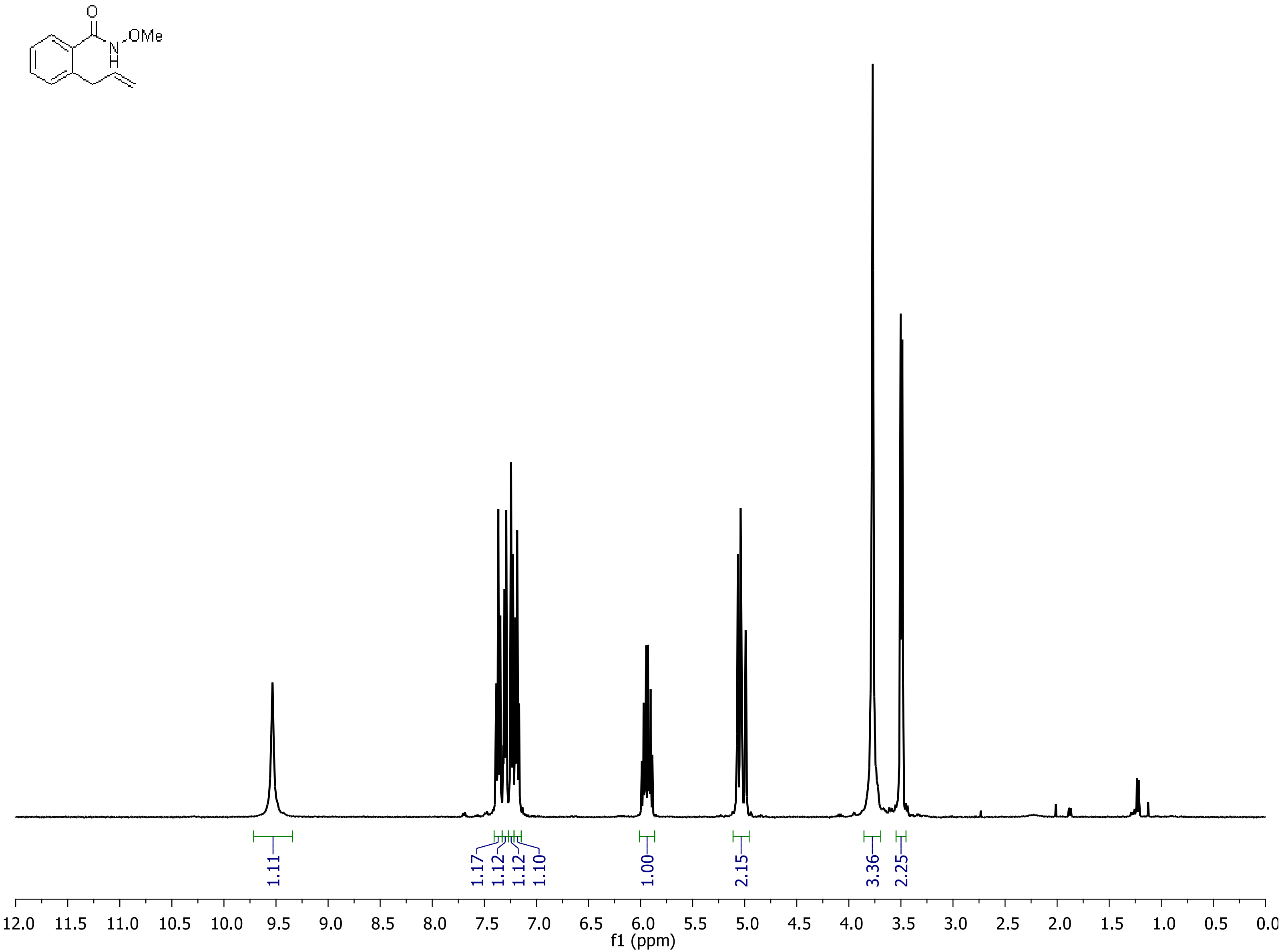

O
N
H
OMe
1.11
1.17
1.12
1.12
1.10
1.00
2.15
3.36
2.25
12.0 11.5 11.0 10.5 10.0 9.5 9.0 8.5 8.0 7.5 7.0 6.5 6.0 5.5 5.0 4.5 4.0 3.5 3.0 2.5 2.0 1.5 1.0 0.5 0.0
f1 (ppm)

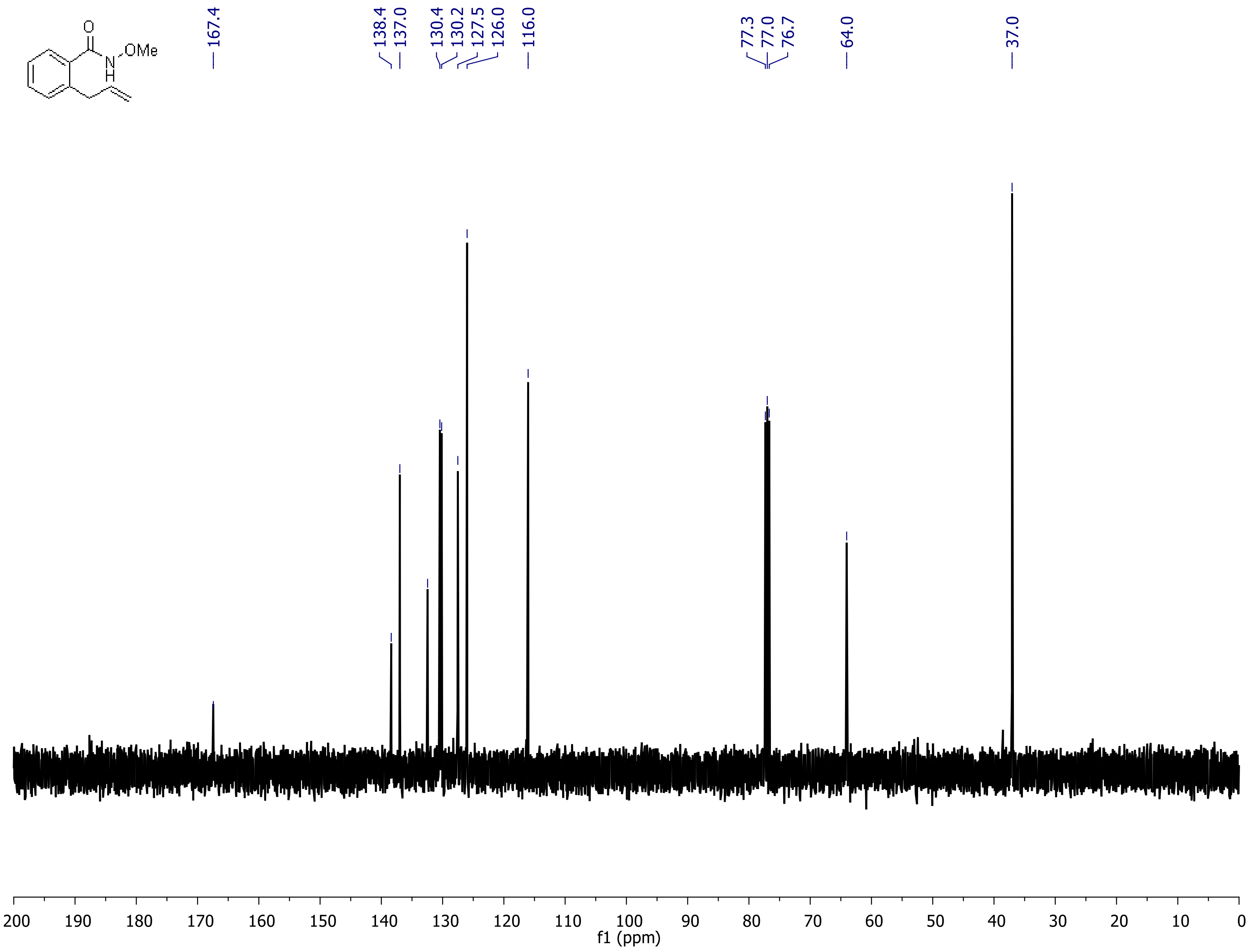
O
N
H
OMe
167.4
138.4
137.0
130.4
130.2
127.5
126.0
116.0
77.3
77.0
76.7
64.0
37.0
200
190
180
170
160
150
140
130
120
110
100
90
80
70
60
50
40
30
20
10
0
f1 (ppm)

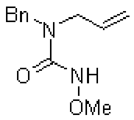
Bn
N
O
NH
OMe

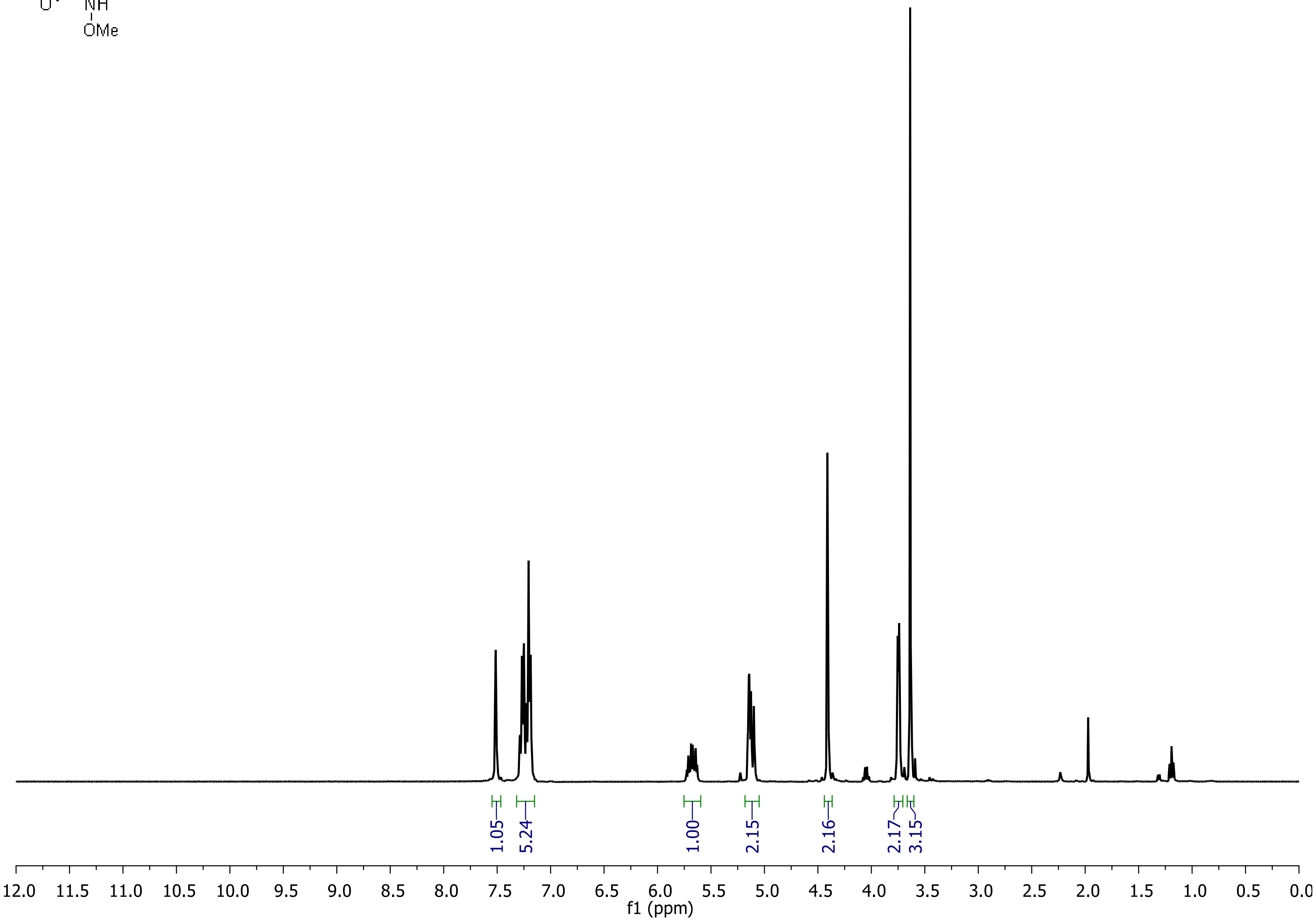
1.05
5.24
1.00
2.15
2.16
2.17
3.15
12.0 11.5 11.0 10.5 10.0 9.5 9.0 8.5 8.0 7.5 7.0 6.5 6.0 5.5 5.0 4.5 4.0 3.5 3.0 2.5 2.0 1.5 1.0 0.5 0.0
f1 (ppm)

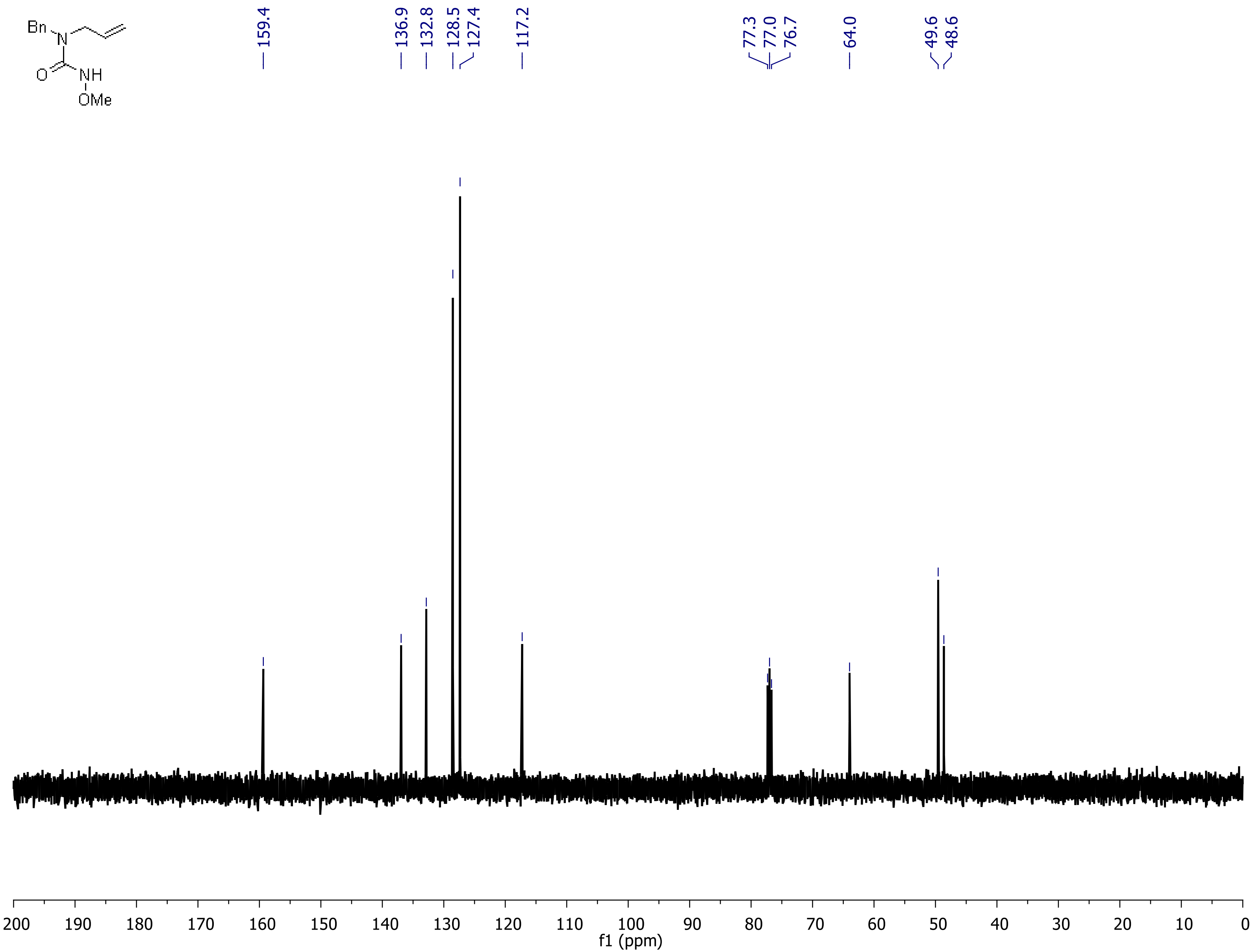
Bn
N
O
NH
OMe
159.4
136.9
132.8
128.5
127.4
117.2
77.3
77.0
76.7
64.0
49.6
48.6
200
190
180
170
160
150
140
130
120
110
100
90
80
70
60
50
40
30
20
10
0
f1 (ppm)

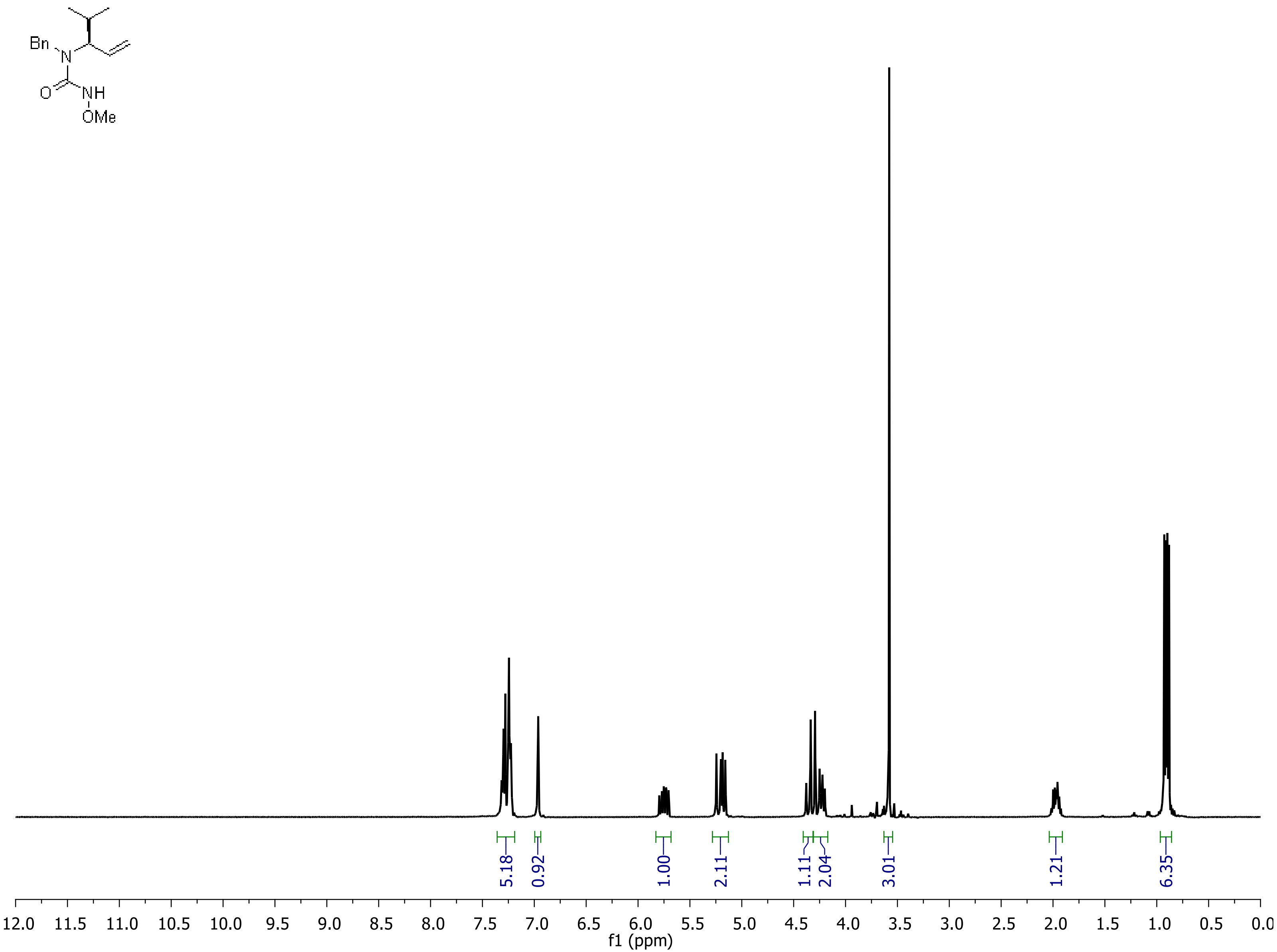
Bn
N
O
NH
OMe
5.18
0.92
1.00
2.11
1.11
2.04
3.01
1.21
6.35
12.0 11.5 11.0 10.5 10.0 9.5 9.0 8.5 8.0 7.5 7.0 6.5 6.0 5.5 5.0 4.5 4.0 3.5 3.0 2.5 2.0 1.5 1.0 0.5 0.0
f1 (ppm)

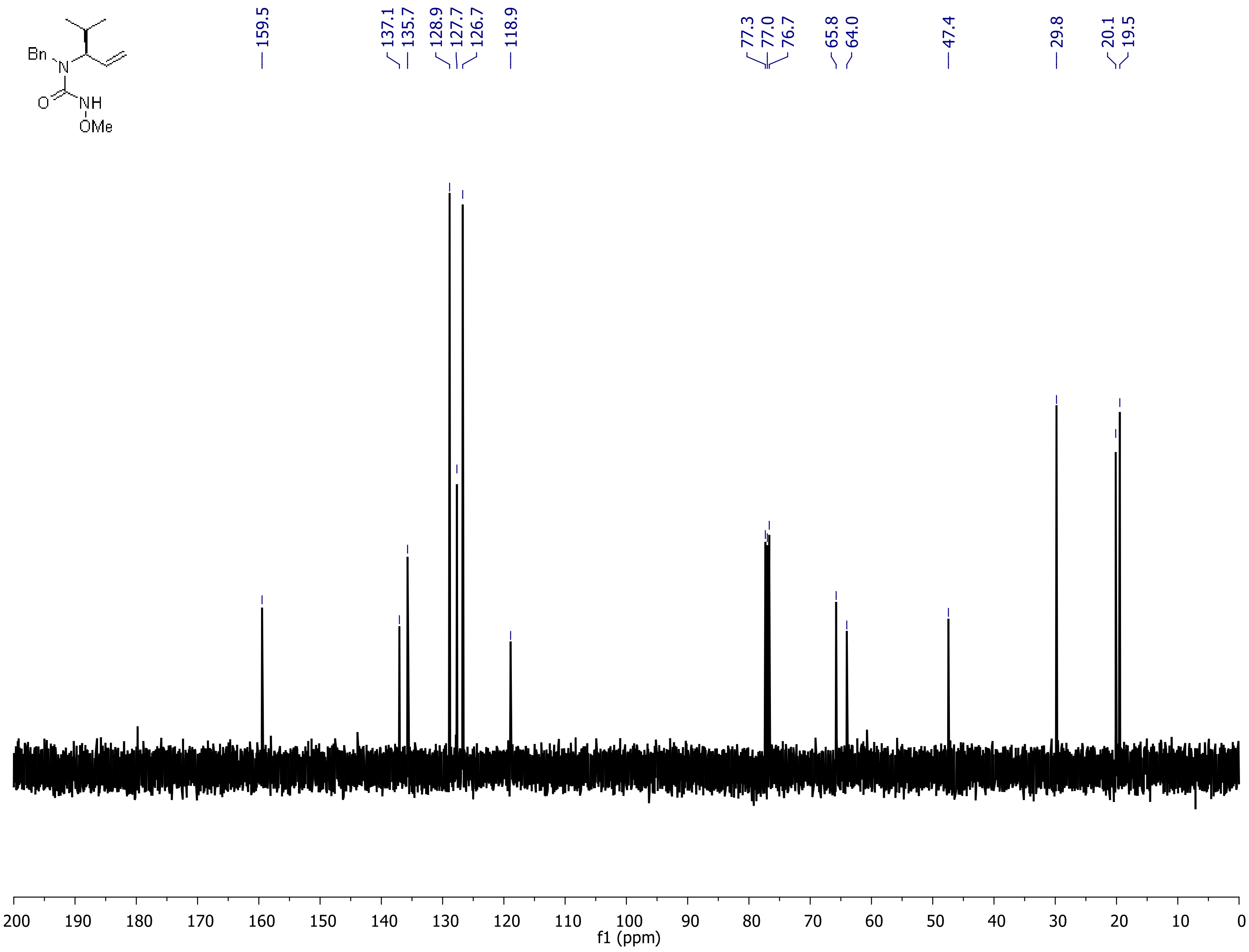
Bn
N
O
NH
OMe
159.5
137.1
135.7
128.9
127.7
126.7
118.9
77.3
77.0
76.7
65.8
64.0
47.4
29.8
20.1
19.5
200
190
180
170
160
150
140
130
120
110
100
90
80
70
60
50
40
30
20
10
0
f1 (ppm)

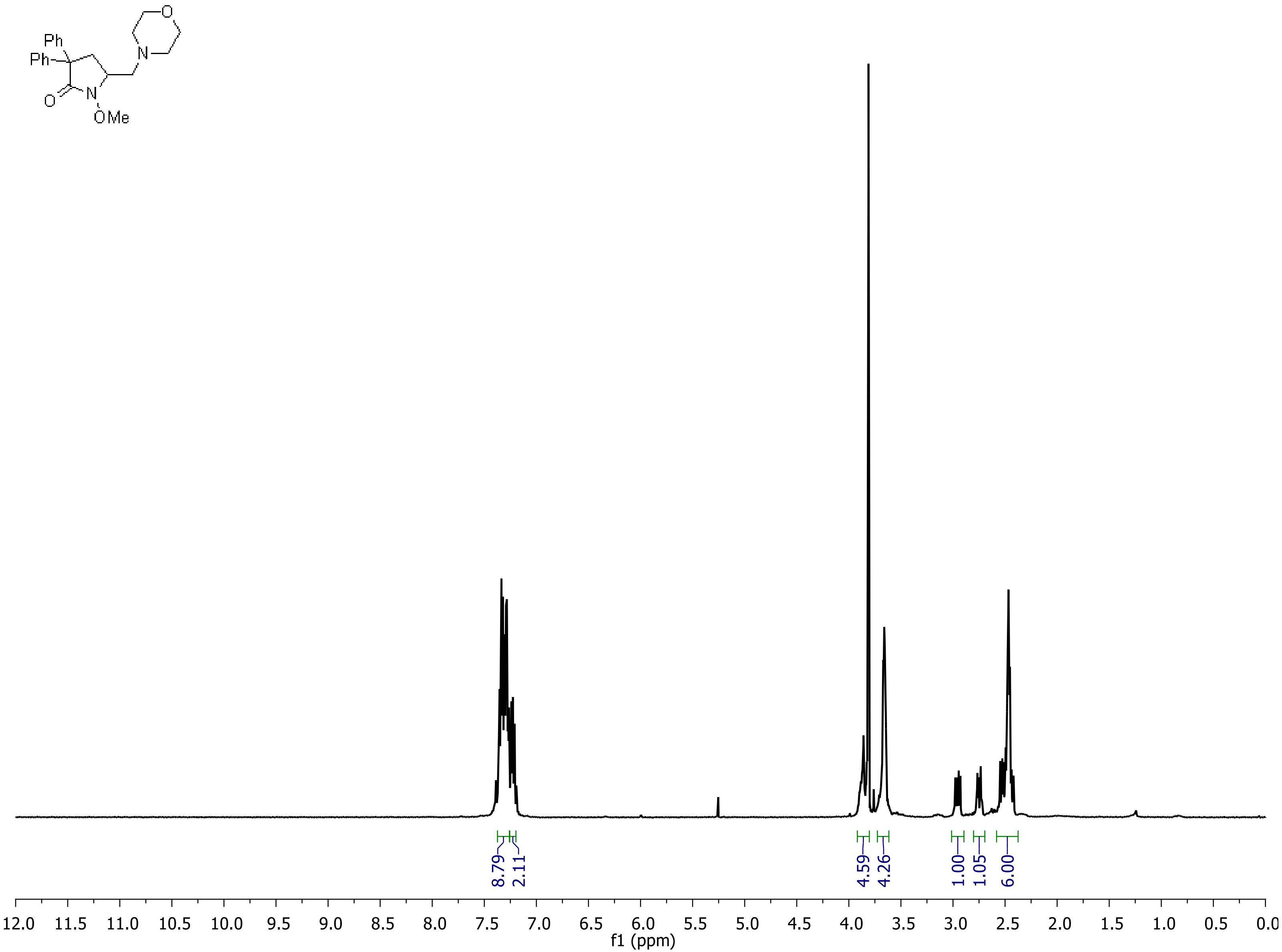
Ph
Ph
O
N
OMe
O
N
8.79
2.11
4.59
4.26
1.00
1.05
6.00
12.0 11.5 11.0 10.5 10.0 9.5 9.0 8.5 8.0 7.5 7.0 6.5 6.0 5.5 5.0 4.5 4.0 3.5 3.0 2.5 2.0 1.5 1.0 0.5 0.0
f1 (ppm)

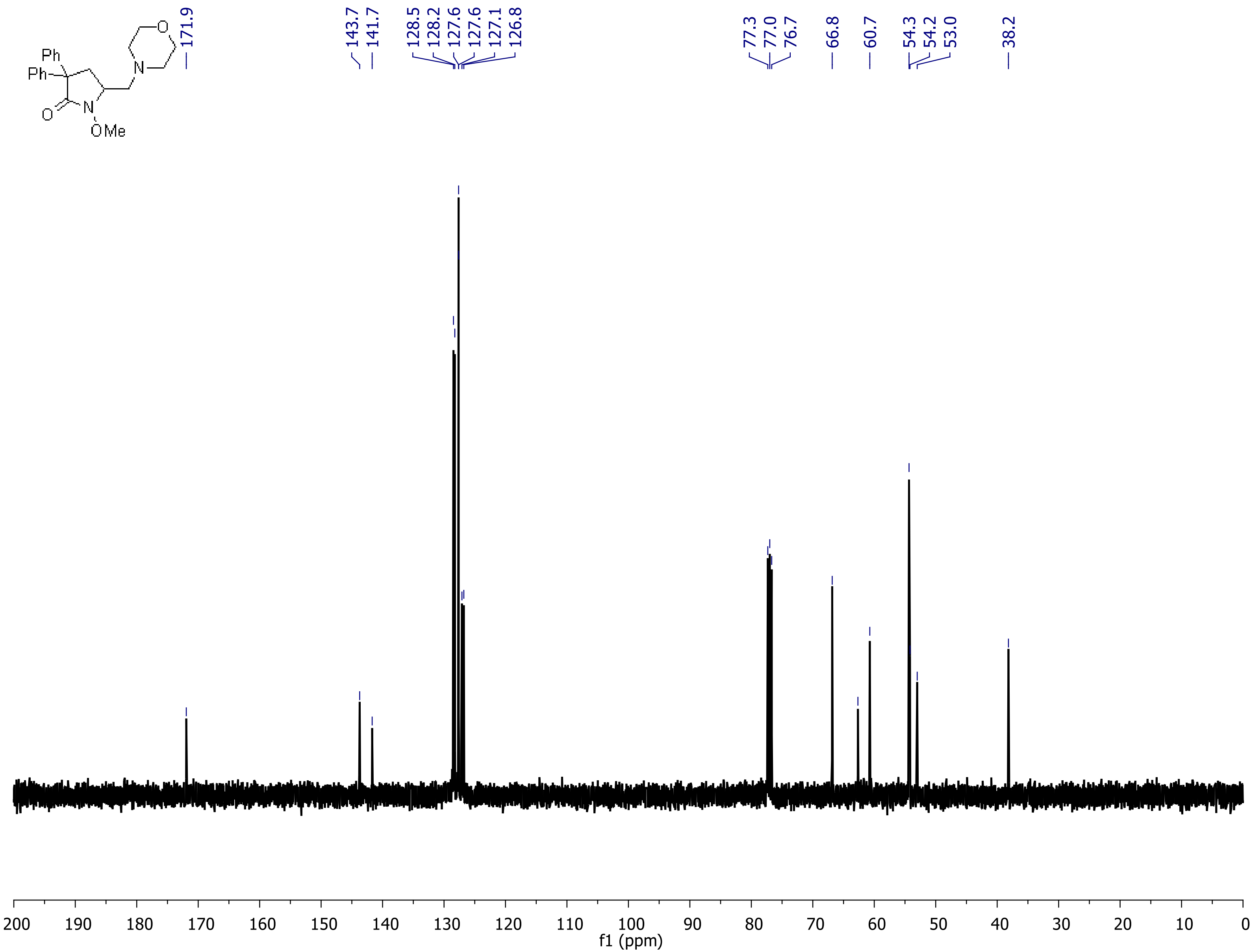

Ph
Ph
O
N
OMe
N
O
171.9
143.7
141.7
128.5
128.2
127.6
127.6
127.1
126.8
77.3
77.0
76.7
66.8
60.7
54.3
54.2
53.0
38.2
200
190
180
170
160
150
140
130
120
110
100
90
80
70
60
50
40
30
20
10
0
f1 (ppm)

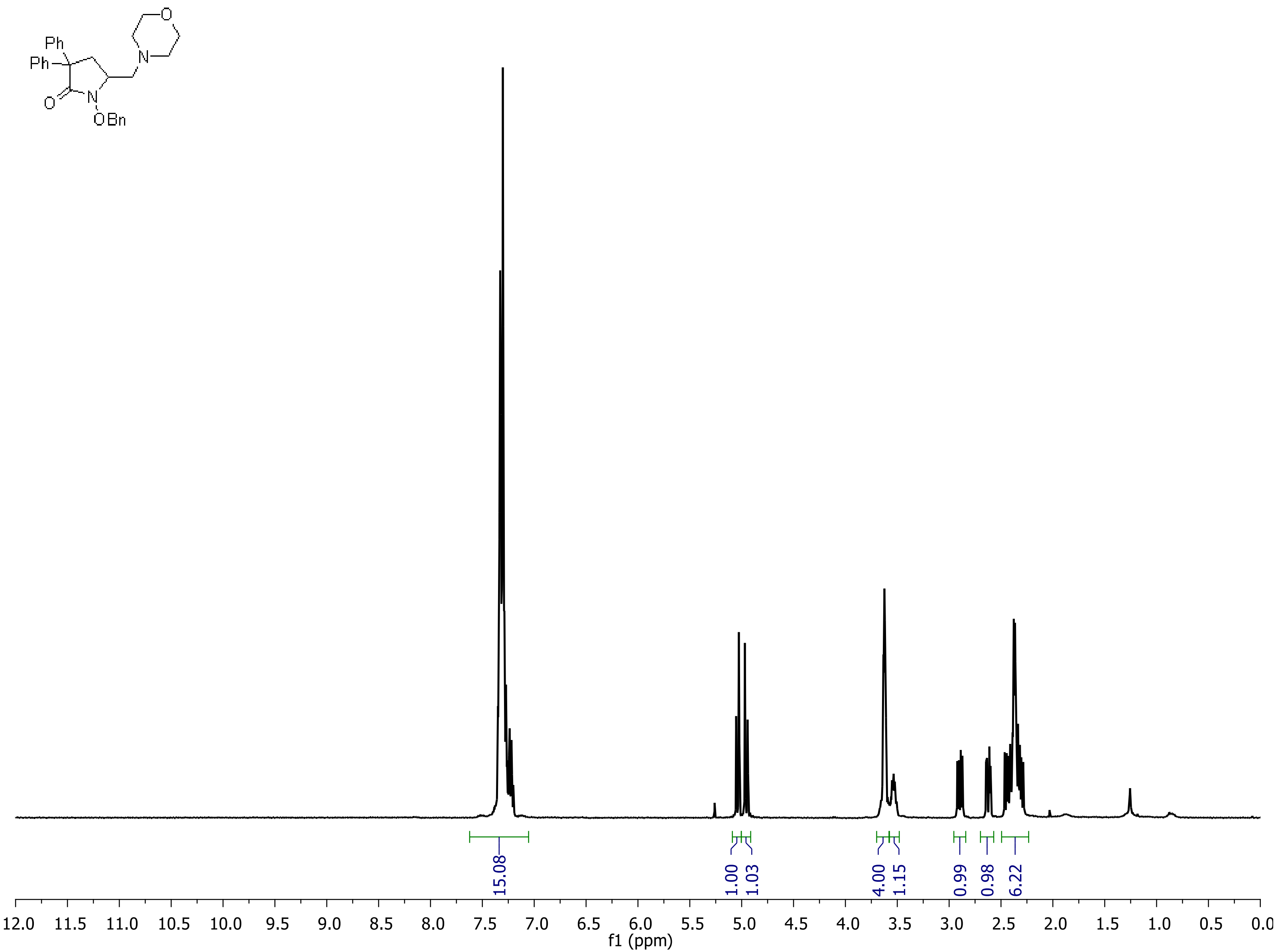

Ph
Ph
O
N
OBn
O
N
15.08
1.00
1.03
4.00
1.15
0.99
0.98
6.22
12.0 11.5 11.0 10.5 10.0 9.5 9.0 8.5 8.0 7.5 7.0 6.5 6.0 5.5 5.0 4.5 4.0 3.5 3.0 2.5 2.0 1.5 1.0 0.5 0.0
f1 (ppm)

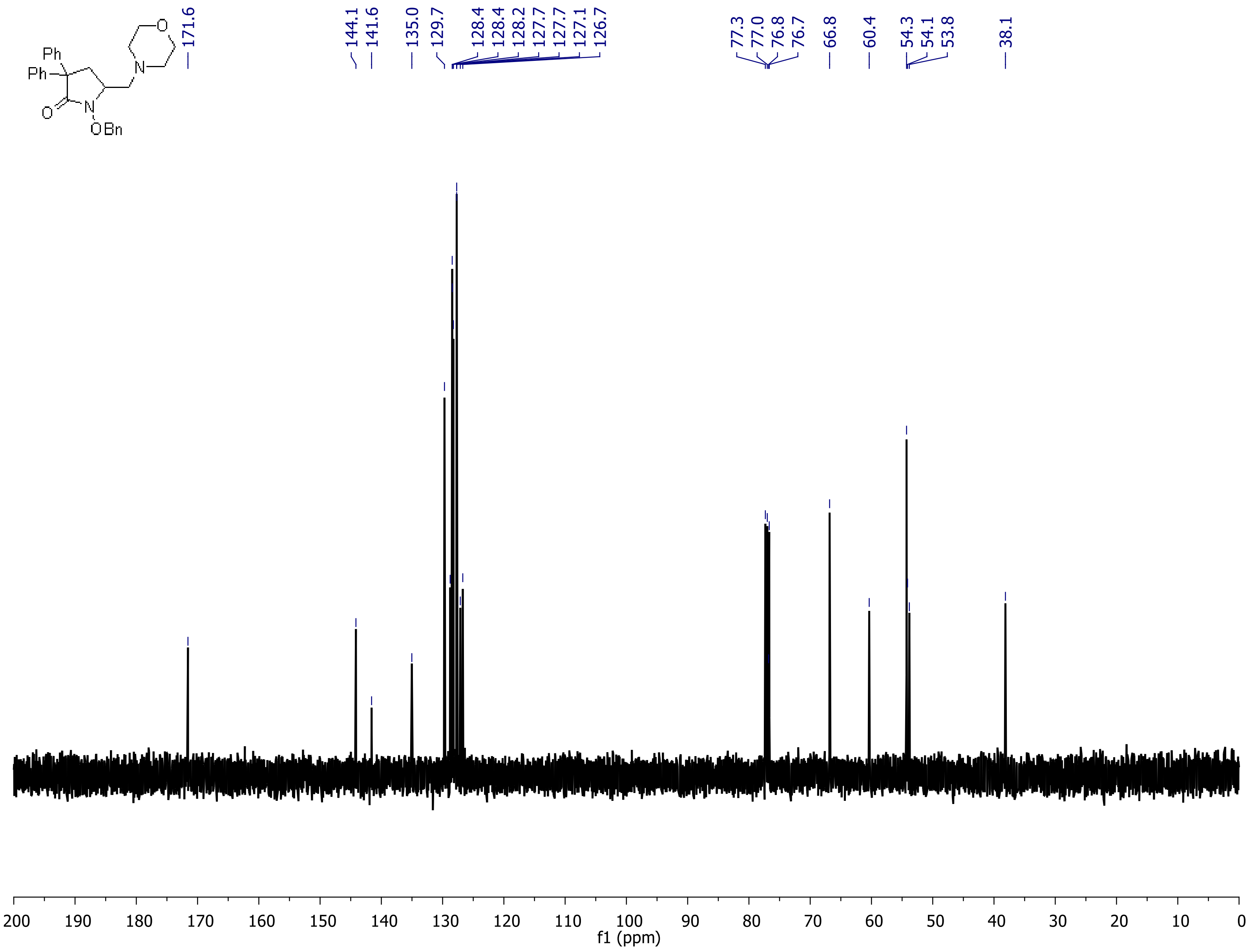
Ph
Ph
O
N
OBn
N
O
171.6
144.1
141.6
135.0
129.7
128.4
128.4
128.2
127.7
127.7
127.1
126.7
77.3
77.0
76.8
76.7
66.8
60.4
54.3
54.1
53.8
38.1
200 190 180 170 160 150 140 130 120 110 100 90 80 70 60 50 40 30 20 10 0
f1 (ppm)

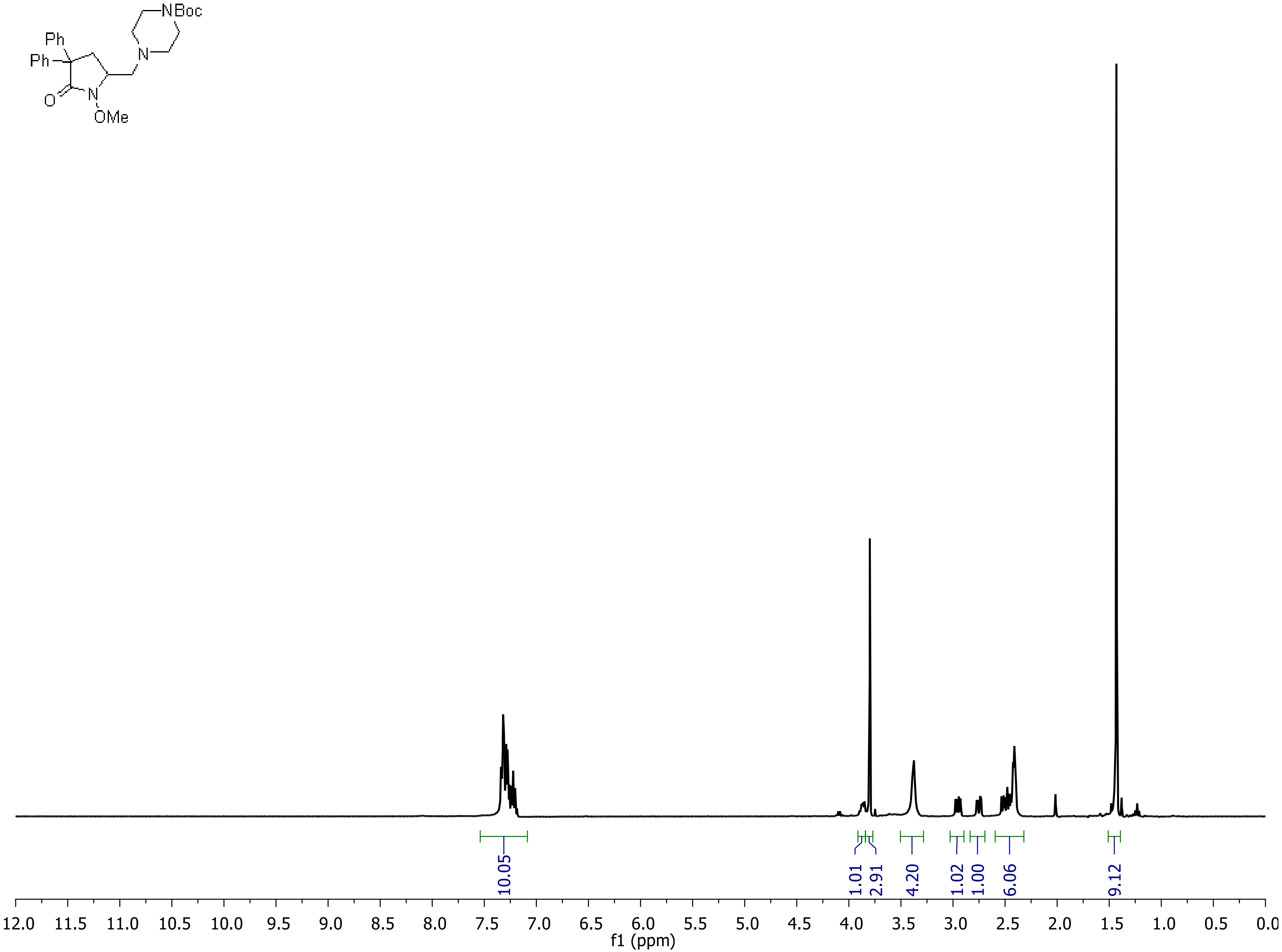

NBoc
Ph
Ph
O
N
OMe
N
10.05
1.01
2.91
4.20
1.02
1.00
6.06
9.12
12.0 11.5 11.0 10.5 10.0 9.5 9.0 8.5 8.0 7.5 7.0 6.5 6.0 5.5 5.0 4.5 4.0 3.5 3.0 2.5 2.0 1.5 1.0 0.5 0.0
f1 (ppm)

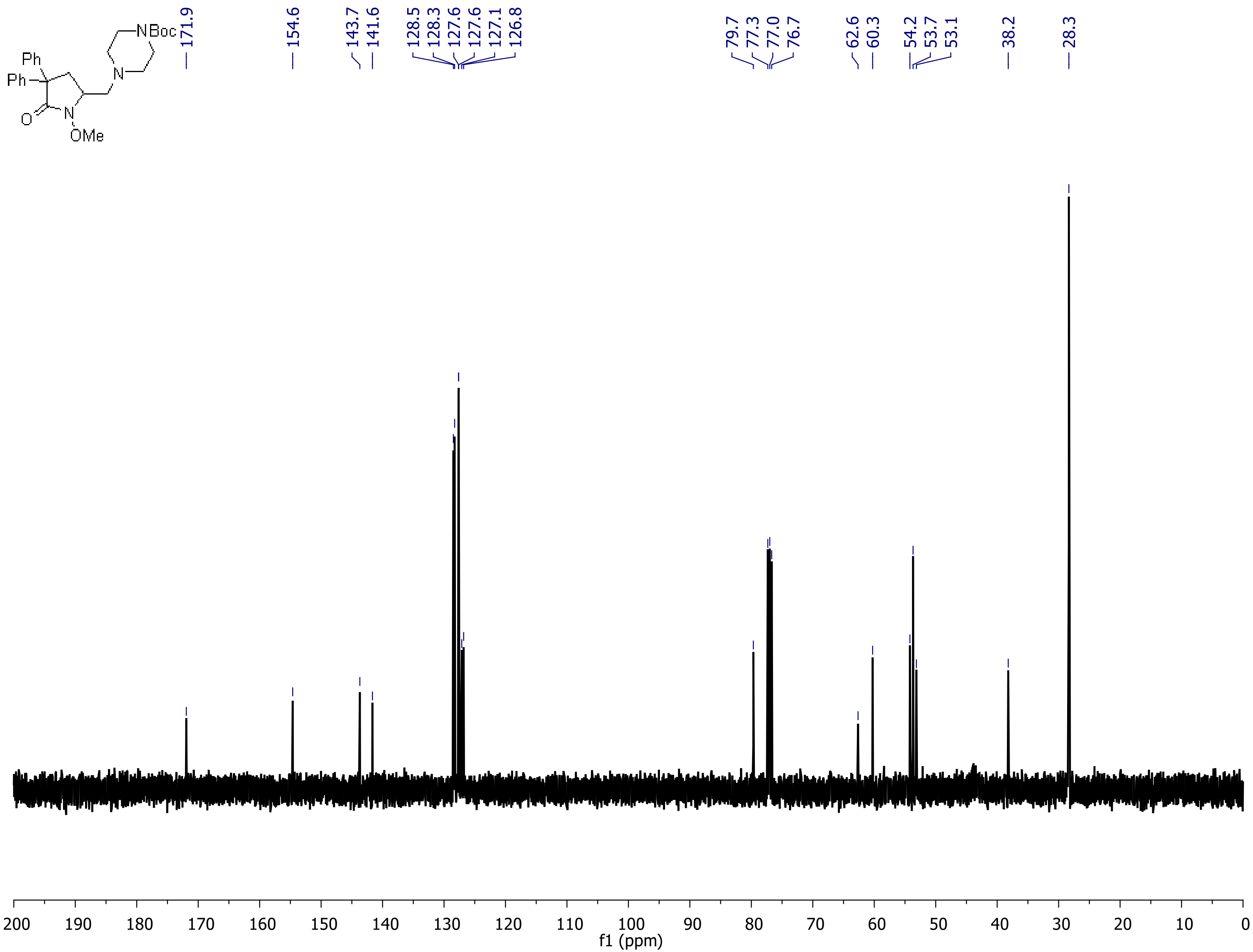
Ph
Ph
NBoc
N
O
N
OMe
171.9
154.6
143.7
141.6
128.5
128.3
127.6
127.6
127.1
126.8
79.7
77.3
77.0
76.7
62.6
60.3
54.2
53.7
53.1
38.2
28.3
200 190 180 170 160 150 140 130 120 110 100 90 80 70 60 50 40 30 20 10 0
f1 (ppm)

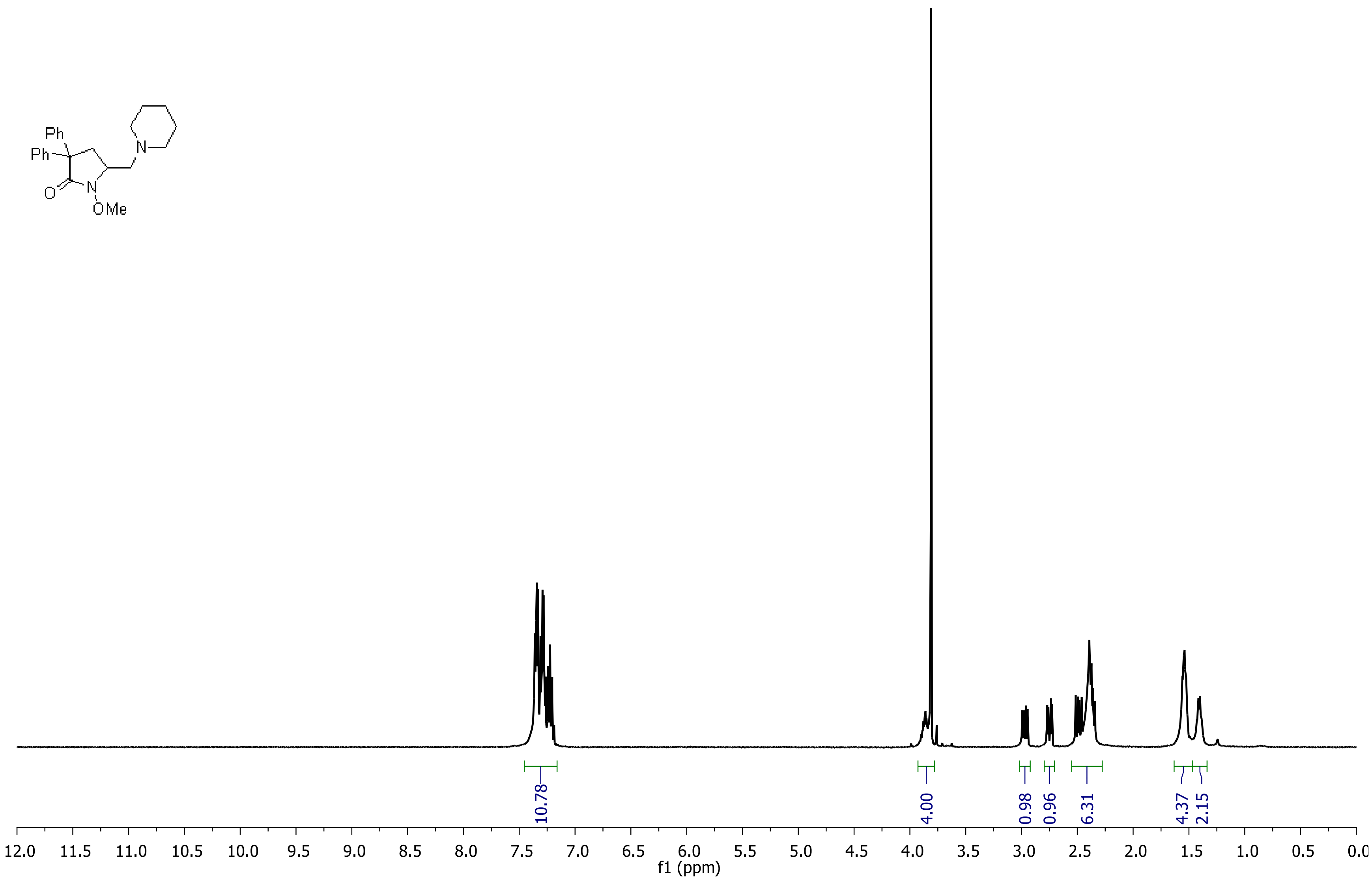
Ph
Ph
N
O
N
OMe
10.78
4.00
0.98
0.96
6.31
4.37
2.15
12.0 11.5 11.0 10.5 10.0 9.5 9.0 8.5 8.0 7.5 7.0 6.5 6.0 5.5 5.0 4.5 4.0 3.5 3.0 2.5 2.0 1.5 1.0 0.5 0.0
f1 (ppm)

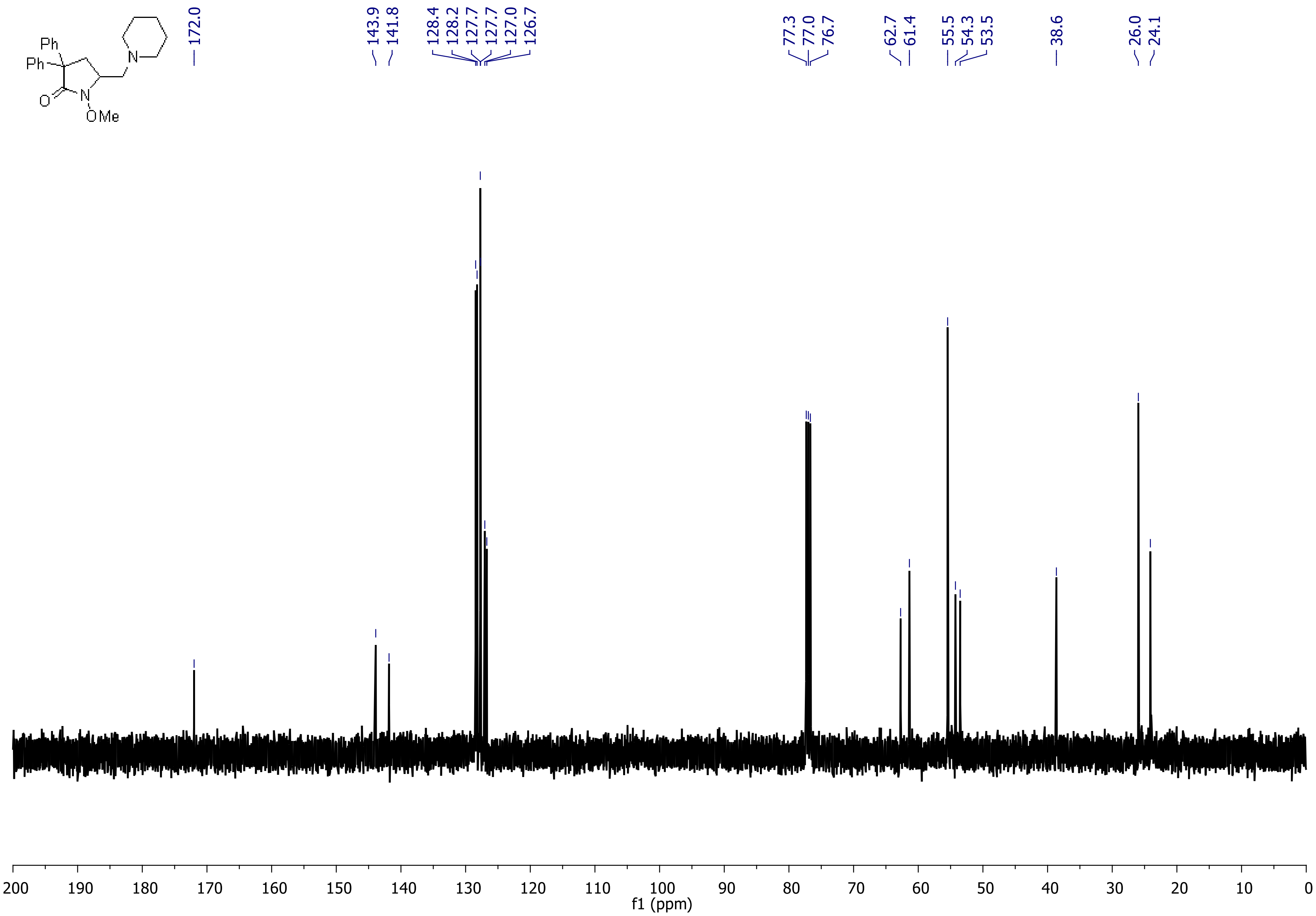

Ph
Ph
O
N
OMe
N
172.0
143.9
141.8
128.4
128.2
127.7
127.7
127.0
126.7
77.3
77.0
76.7
62.7
61.4
55.5
54.3
53.5
38.6
26.0
24.1
200 190 180 170 160 150 140 130 120 110 100 90 80 70 60 50 40 30 20 10 0
f1 (ppm)

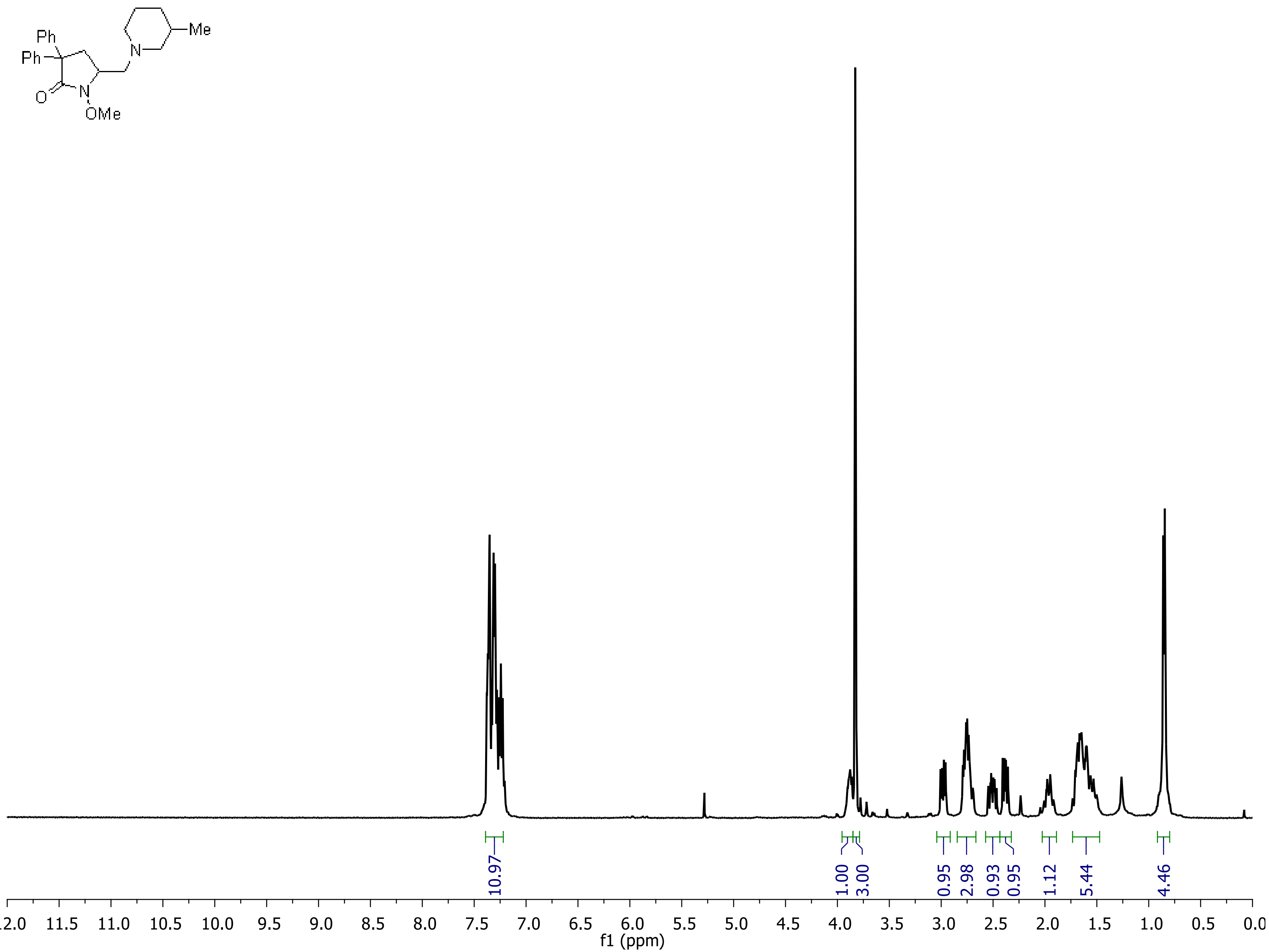
10.97
1.00
3.00
0.95
2.98
0.93
0.95
1.12
5.44
4.46
12.0 11.5 11.0 10.5 10.0 9.5 9.0 8.5 8.0 7.5 7.0 6.5 6.0 5.5 5.0 4.5 4.0 3.5 3.0 2.5 2.0 1.5 1.0 0.5 0.0
f1 (ppm)

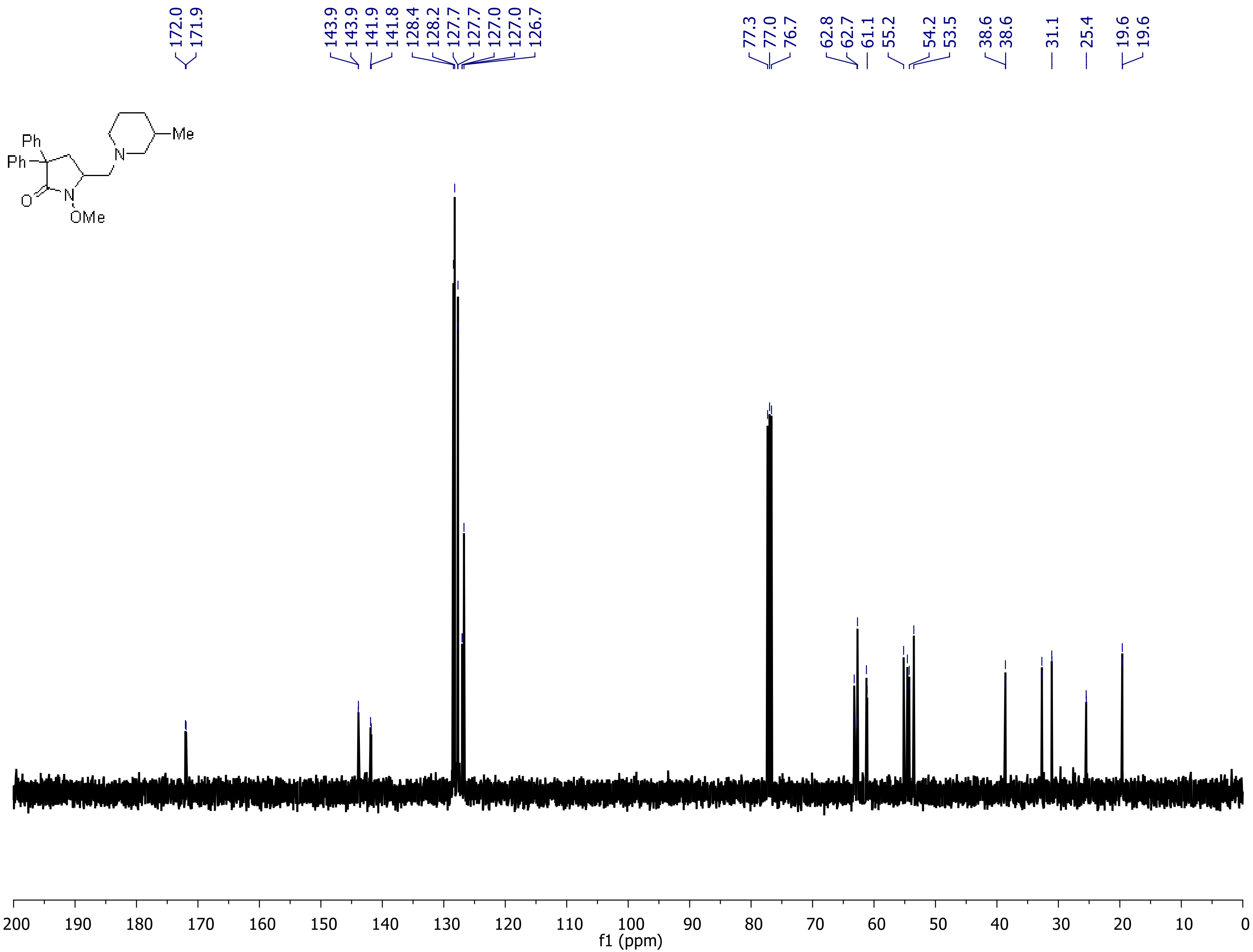
172.0
171.9
143.9
143.9
141.9
141.8
128.4
128.2
127.7
127.7
127.0
127.0
126.7
77.3
77.0
76.7
62.8
62.7
61.1
55.2
54.2
53.5
38.6
38.6
31.1
25.4
19.6
19.6
Ph
Ph
Me
N
O
N
OMe
200
190
180
170
160
150
140
130
120
110
100
90
80
70
60
50
40
30
20
10
0
f1 (ppm)

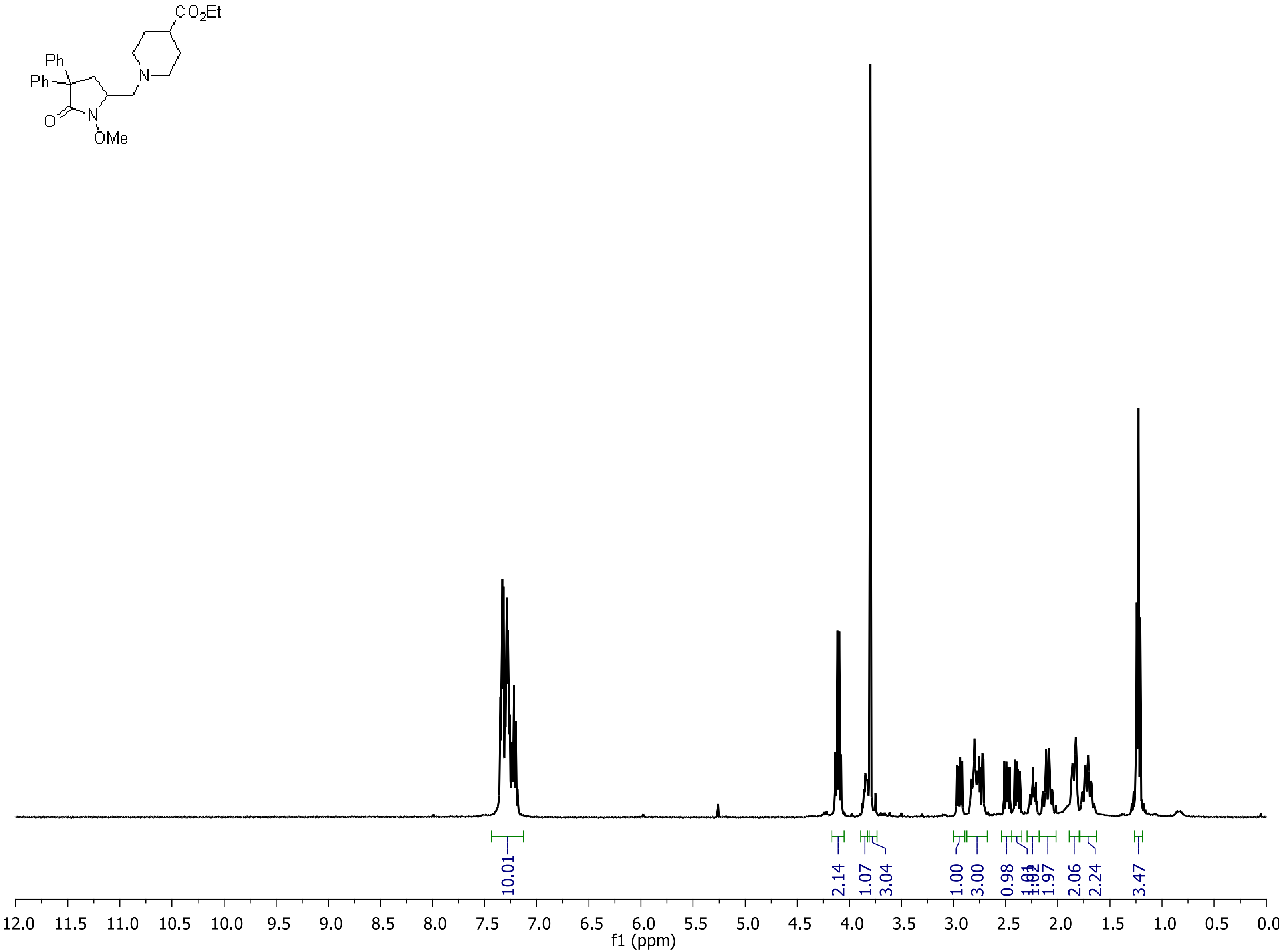
CO2Et
Ph
Ph
O
N
OMe
10.01
2.14
1.07
3.04
1.00
3.00
0.98
1.97
2.06
2.24
3.47
12.0 11.5 11.0 10.5 10.0 9.5 9.0 8.5 8.0 7.5 7.0 6.5 6.0 5.5 5.0 4.5 4.0 3.5 3.0 2.5 2.0 1.5 1.0 0.5 0.0
f1 (ppm)

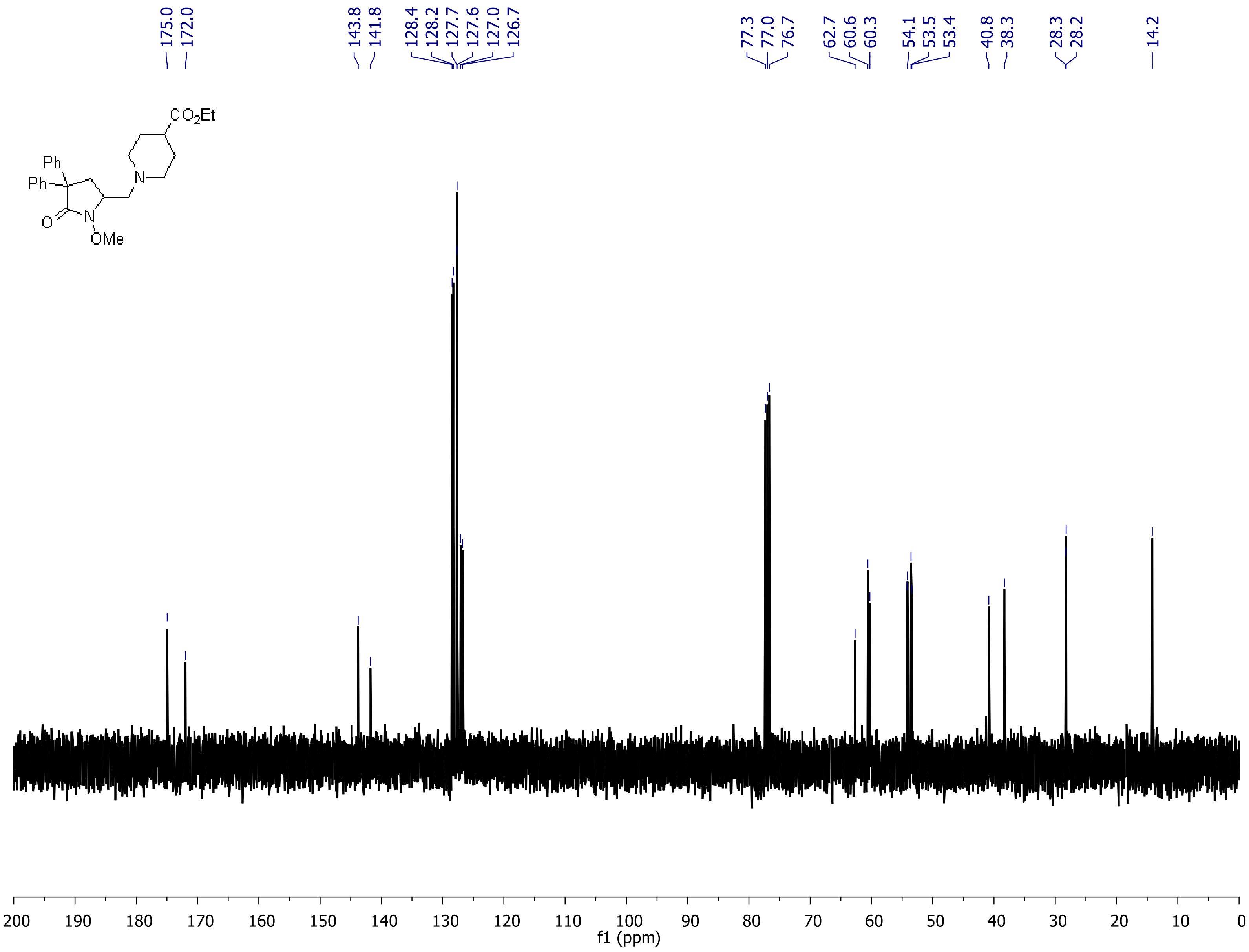
175.0
172.0
143.8
141.8
128.4
128.2
127.7
127.6
127.0
126.7
77.3
77.0
76.7
62.7
60.6
60.3
54.1
53.5
53.4
40.8
38.3
28.3
28.2
14.2
CO2Et
Ph
Ph
O
N
N
OMe
200 190 180 170 160 150 140 130 120 110 100 90 80 70 60 50 40 30 20 10 0
f1 (ppm)

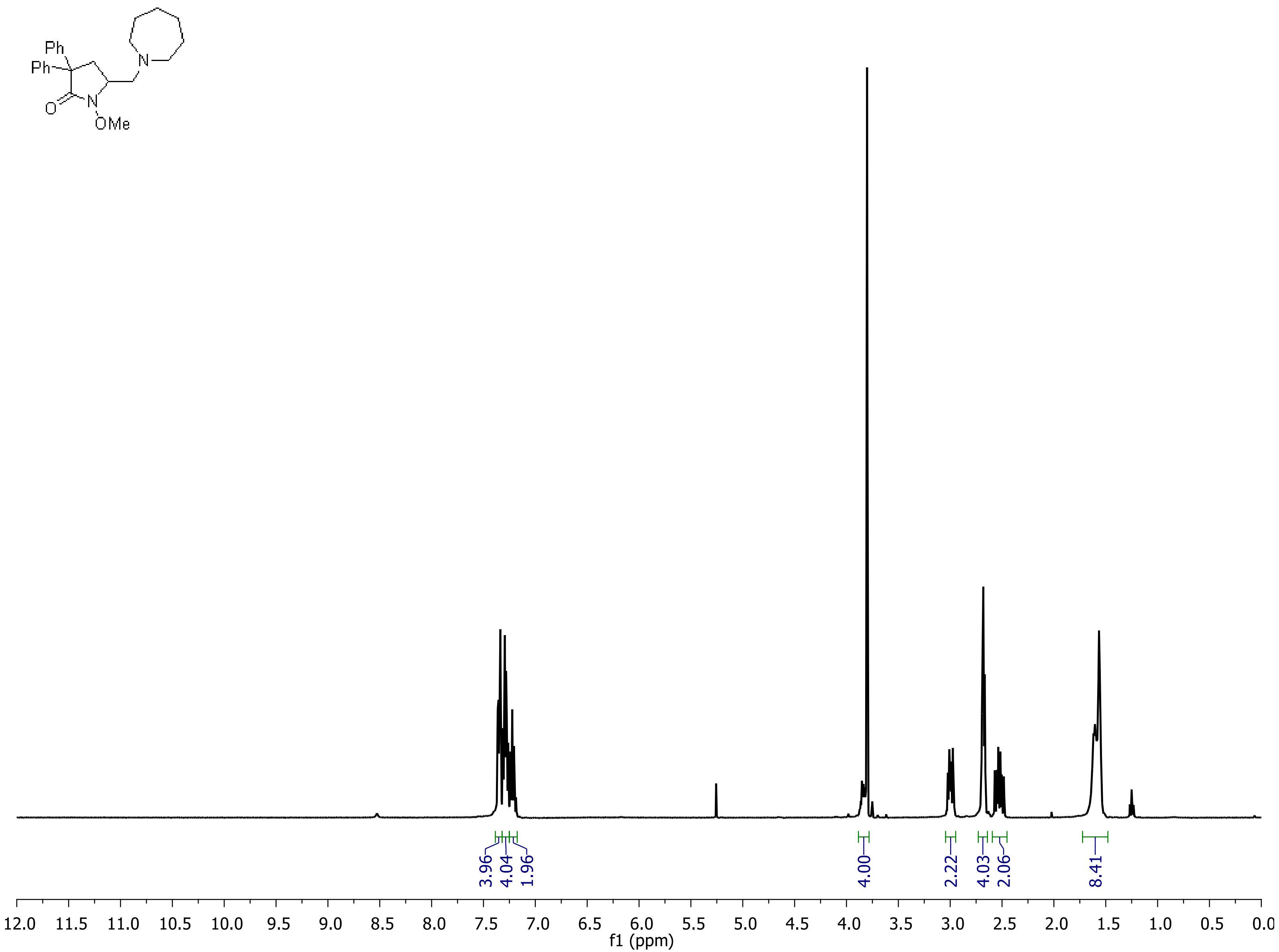

Ph
Ph
O
N
OMe
N
3.96
4.04
1.96
4.00
2.22
4.03
2.06
8.41
12.0 11.5 11.0 10.5 10.0 9.5 9.0 8.5 8.0 7.5 7.0 6.5 6.0 5.5 5.0 4.5 4.0 3.5 3.0 2.5 2.0 1.5 1.0 0.5 0.0
f1 (ppm)

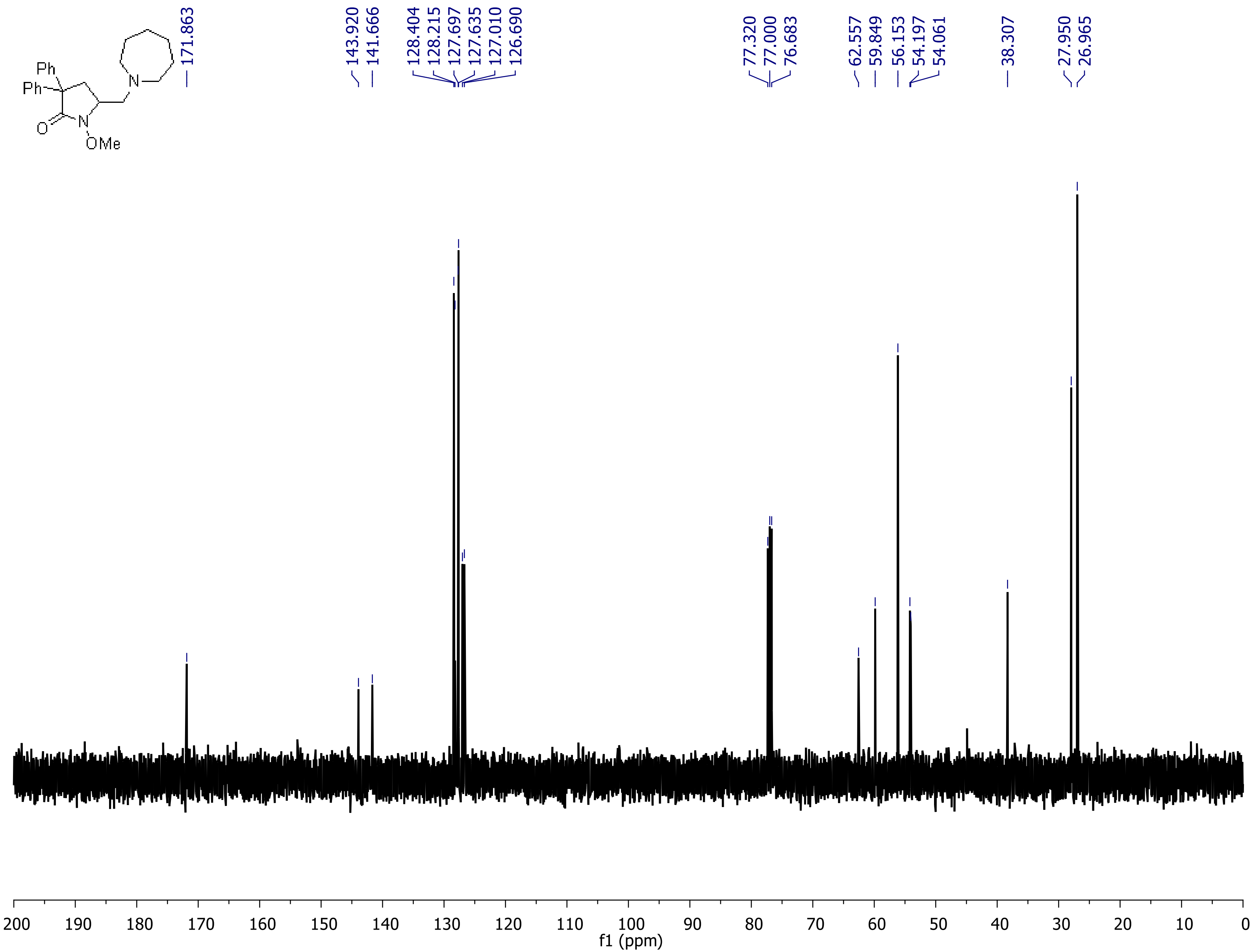
Ph
Ph
O
N
OMe
N
171.863
143.920
141.666
128.404
128.215
127.697
127.635
127.010
126.690
77.320
77.000
76.683
62.557
59.849
56.153
54.197
54.061
38.307
27.950
26.965
200 190 180 170 160 150 140 130 120 110 100 90 80 70 60 50 40 30 20 10 0
f1 (ppm)

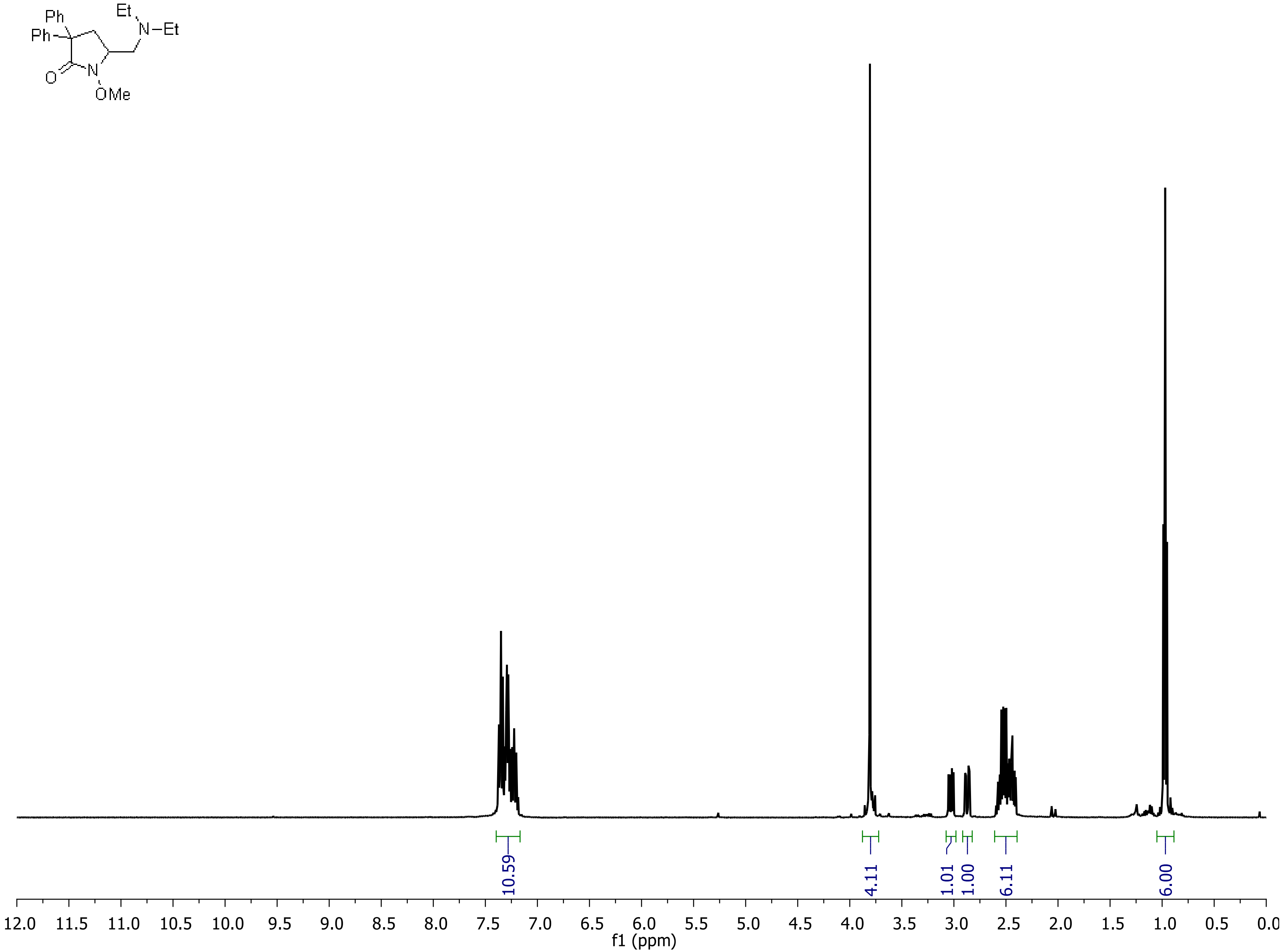

Ph
Ph
Et
N
Et
O
N
OMe
10.59
4.11
1.01
1.00
6.11
6.00
12.0
11.5
11.0
10.5
10.0
9.5
9.0
8.5
8.0
7.5
7.0
6.5
6.0
5.5
5.0
4.5
4.0
3.5
3.0
2.5
2.0
1.5
1.0
0.5
f1 (ppm)

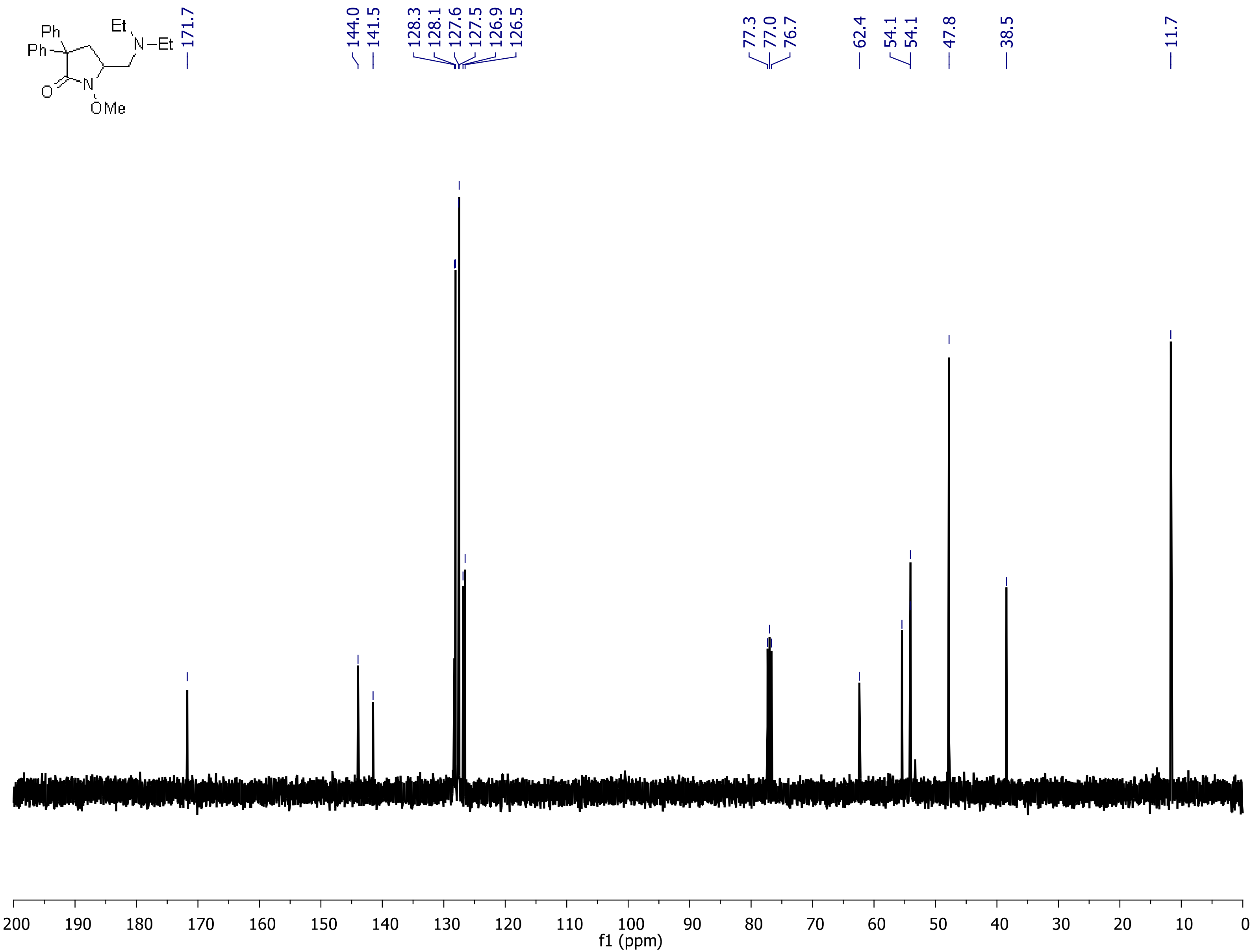
Ph
Ph
Et
N
Et
O
N
OMe
171.7
144.0
141.5
128.3
128.1
127.6
127.5
126.9
126.5
77.3
77.0
76.7
62.4
54.1
54.1
47.8
38.5
11.7
200
190
180
170
160
150
140
130
120
110
100
90
80
70
60
50
40
30
20
10
0
f1 (ppm)

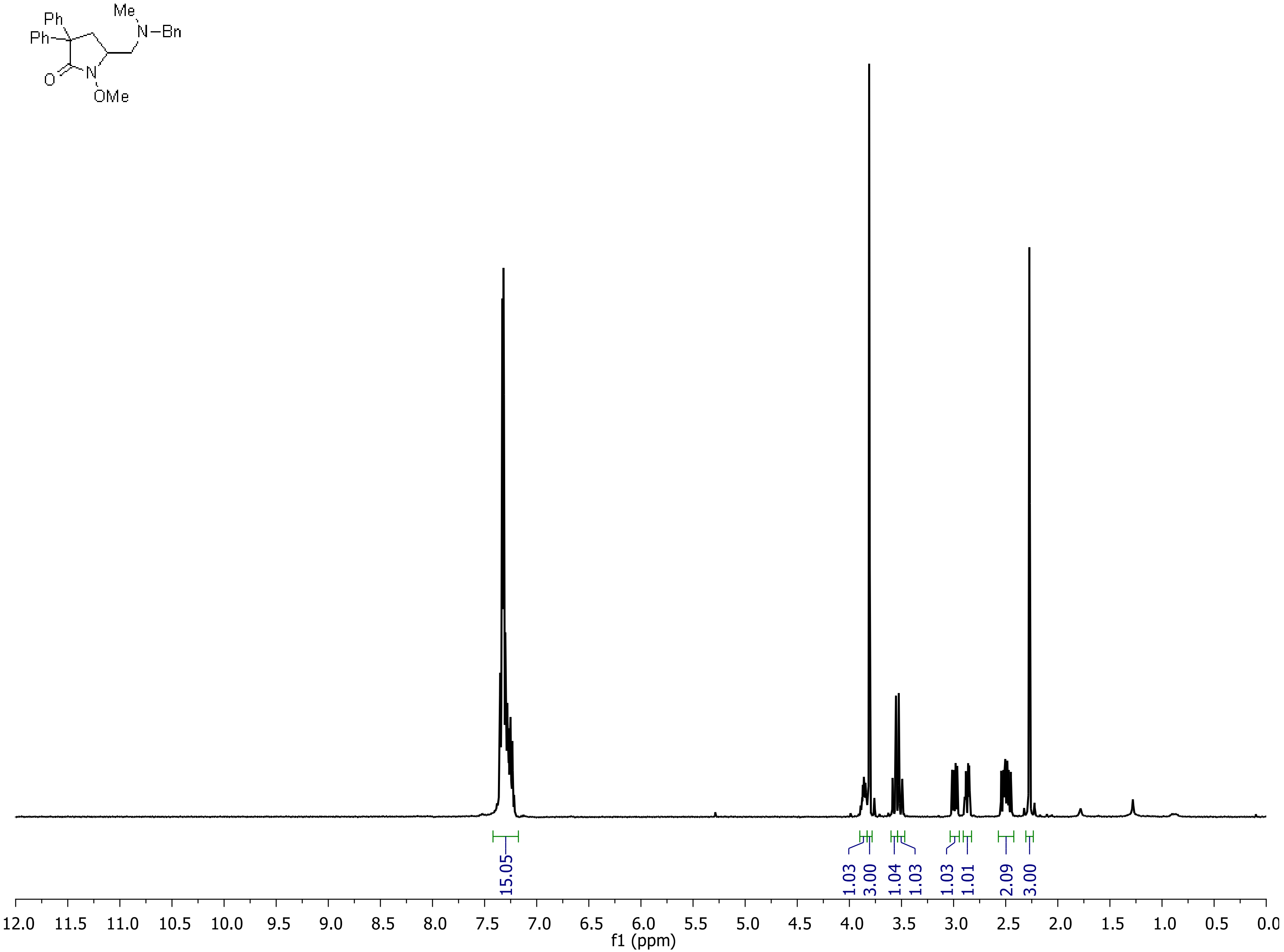
Ph
Ph
Me
N–Bn
O
N
OMe
15.05
1.03
3.00
1.04
1.03
1.03
1.01
2.09
3.00
12.0 11.5 11.0 10.5 10.0 9.5 9.0 8.5 8.0 7.5 7.0 6.5 6.0 5.5 5.0 4.5 4.0 3.5 3.0 2.5 2.0 1.5 1.0 0.5 0.0
f1 (ppm)

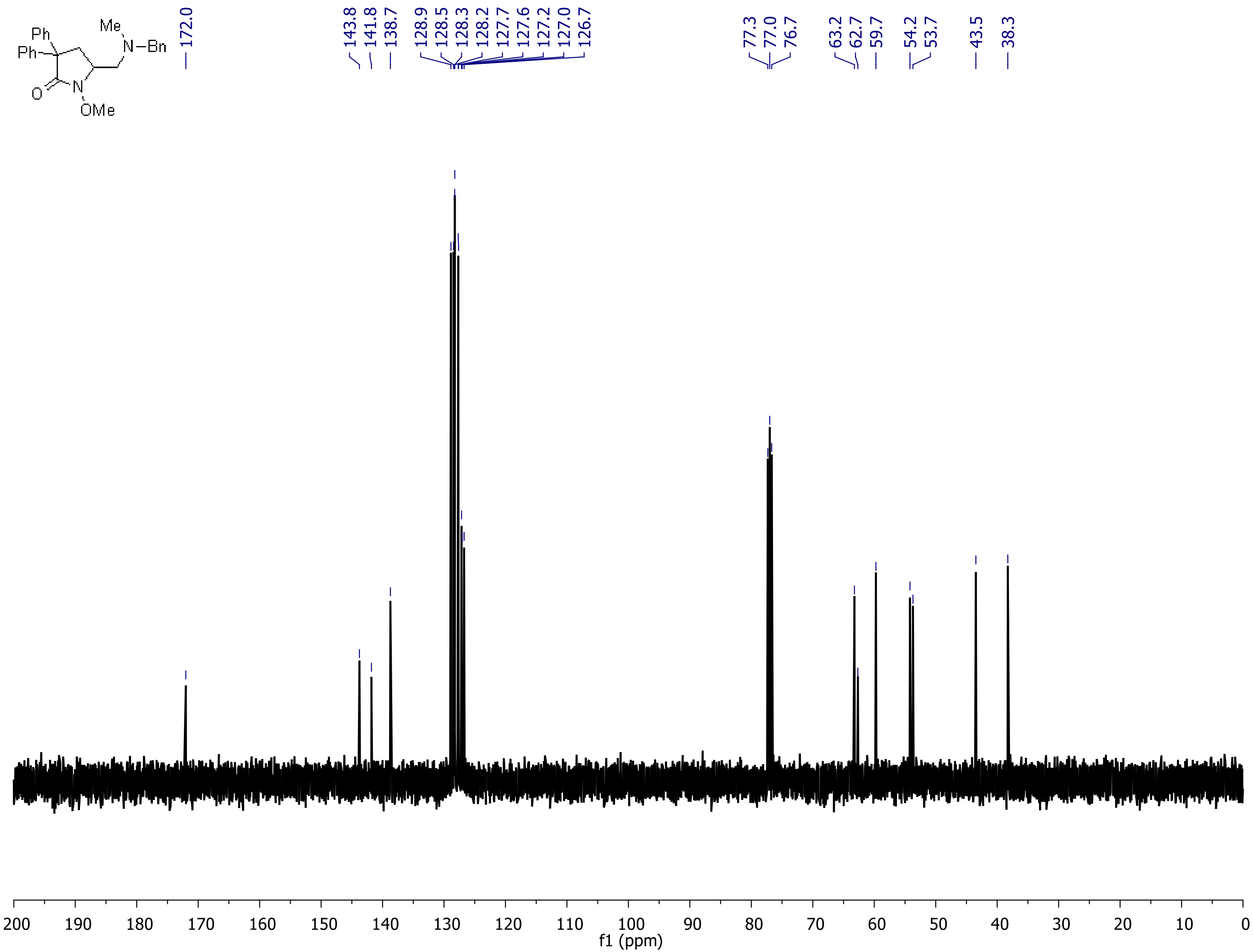

Ph
Ph
Me
N–Bn
O
N
OMe
172.0
143.8
141.8
138.7
128.9
128.5
128.3
128.2
127.7
127.6
127.2
127.0
126.7
77.3
77.0
76.7
63.2
62.7
59.7
54.2
53.7
43.5
38.3
200
190
180
170
160
150
140
130
120
110
100
90
80
70
60
50
40
30
20
10
0
f1 (ppm)

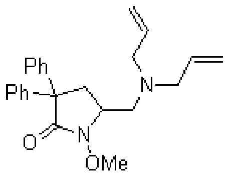
Ph
Ph
O
N
OMe
N

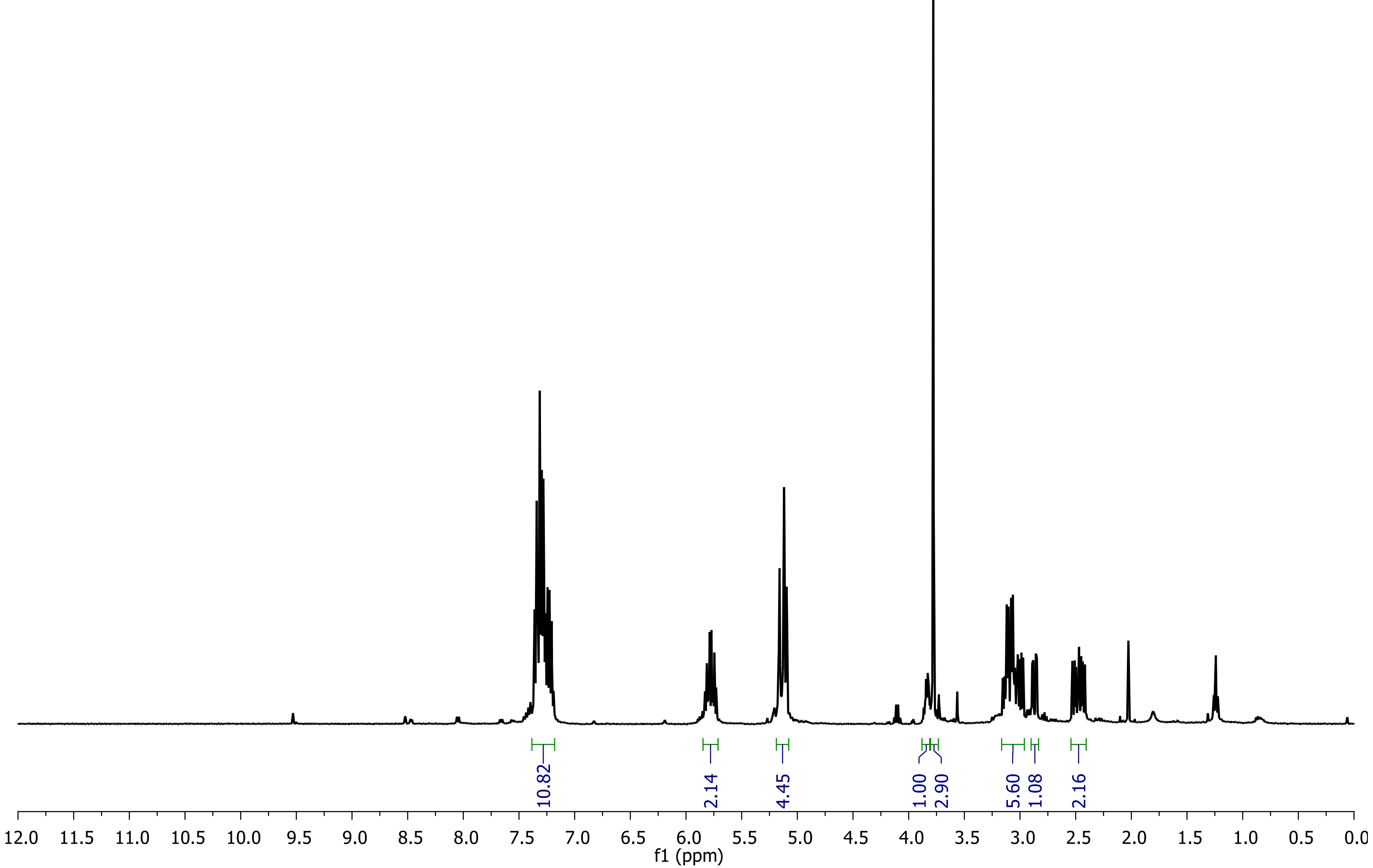
10.82
2.14
4.45
1.00
2.90
5.60
1.08
2.16
12.0 11.5 11.0 10.5 10.0 9.5 9.0 8.5 8.0 7.5 7.0 6.5 6.0 5.5 5.0 4.5 4.0 3.5 3.0 2.5 2.0 1.5 1.0 0.5 0.0
f1 (ppm)

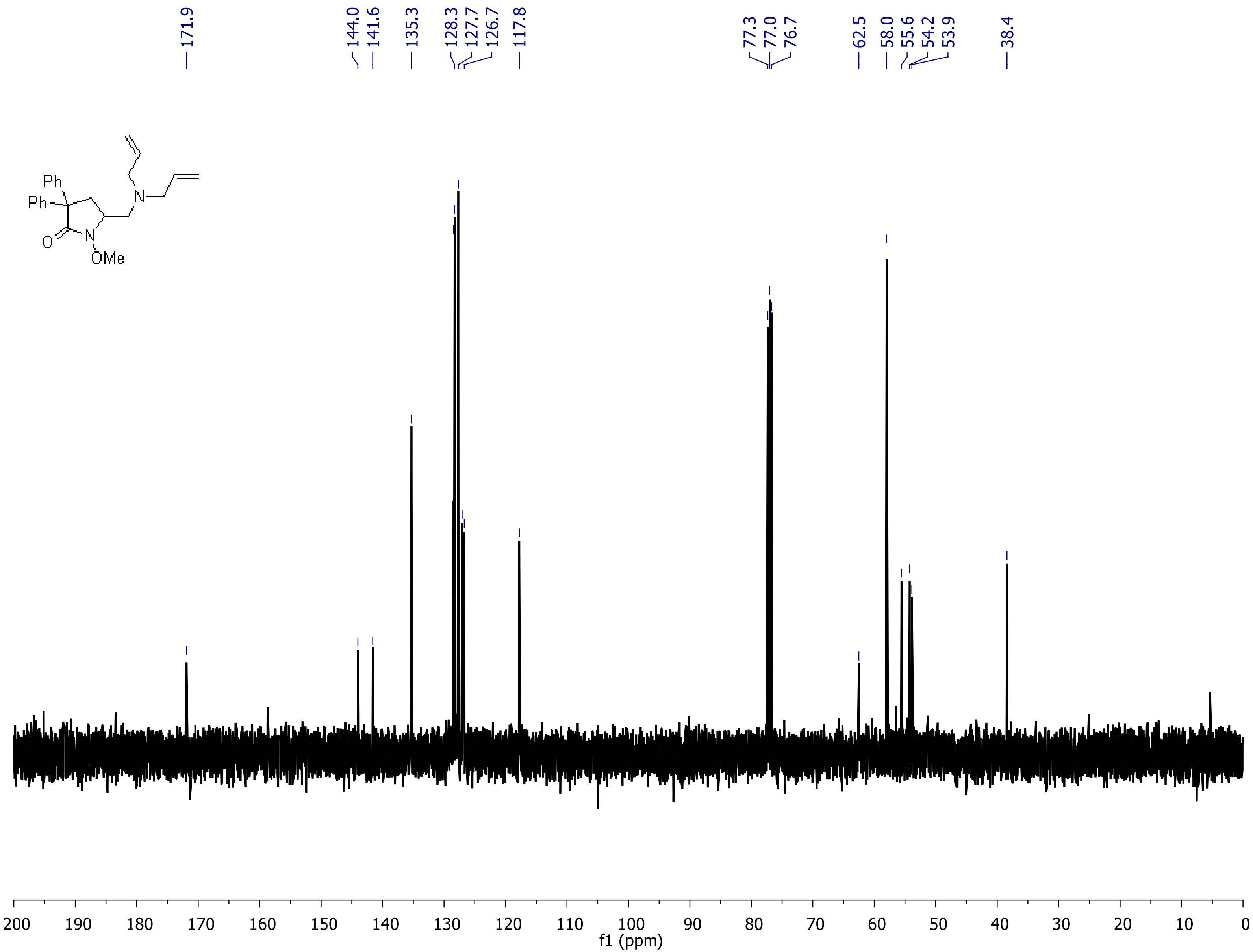
171.9
144.0
141.6
135.3
128.3
127.7
126.7
117.8
77.3
77.0
76.7
62.5
58.0
55.6
54.2
53.9
38.4
Ph
Ph
N
O
N
OMe
200 190 180 170 160 150 140 130 120 110 100 90 80 70 60 50 40 30 20 10 0
f1 (ppm)

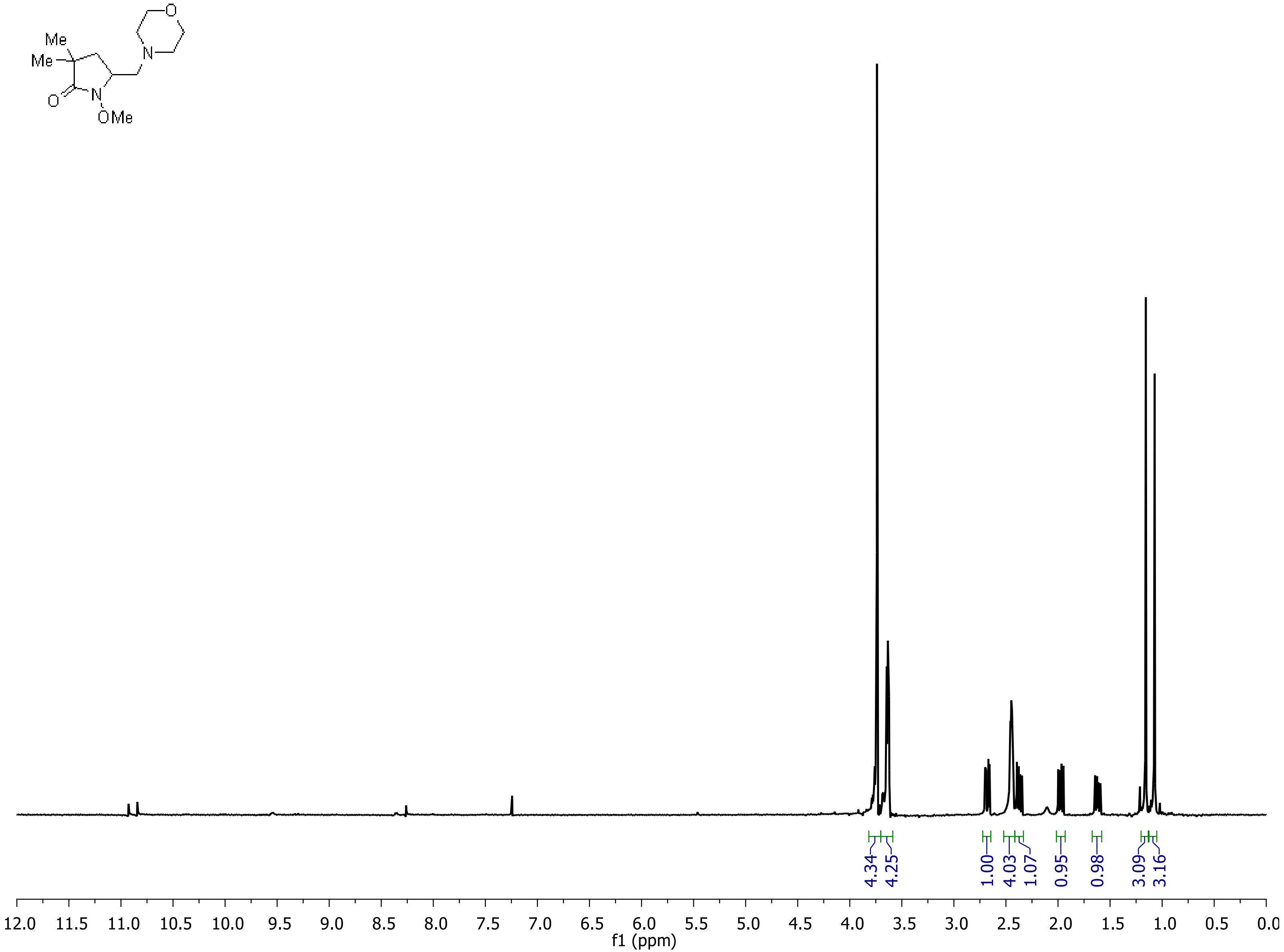

Me
Me
O
N
OMe
N
O
4.34
4.25
1.00
4.03
1.07
0.95
0.98
3.09
3.16
12.0 11.5 11.0 10.5 10.0 9.5 9.0 8.5 8.0 7.5 7.0 6.5 6.0 5.5 5.0 4.5 4.0 3.5 3.0 2.5 2.0 1.5 1.0 0.5 0.0
f1 (ppm)

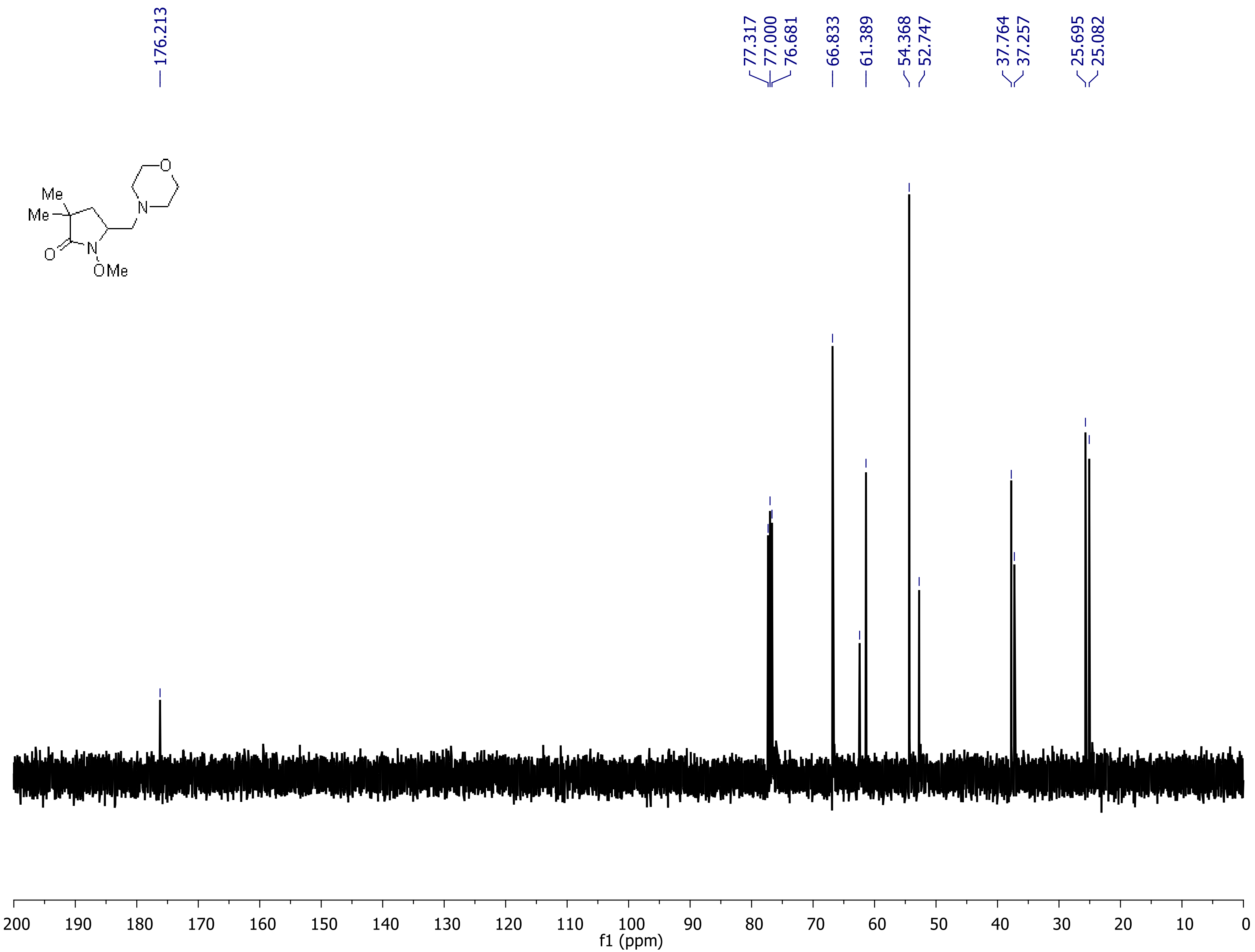

176.213
77.317
77.000
76.681
66.833
61.389
54.368
52.747
37.764
37.257
25.695
25.082
Me
Me
O
N
N
OMe
O
200 190 180 170 160 150 140 130 120 110 100 90 80 70 60 50 40 30 20 10 0
f1 (ppm)

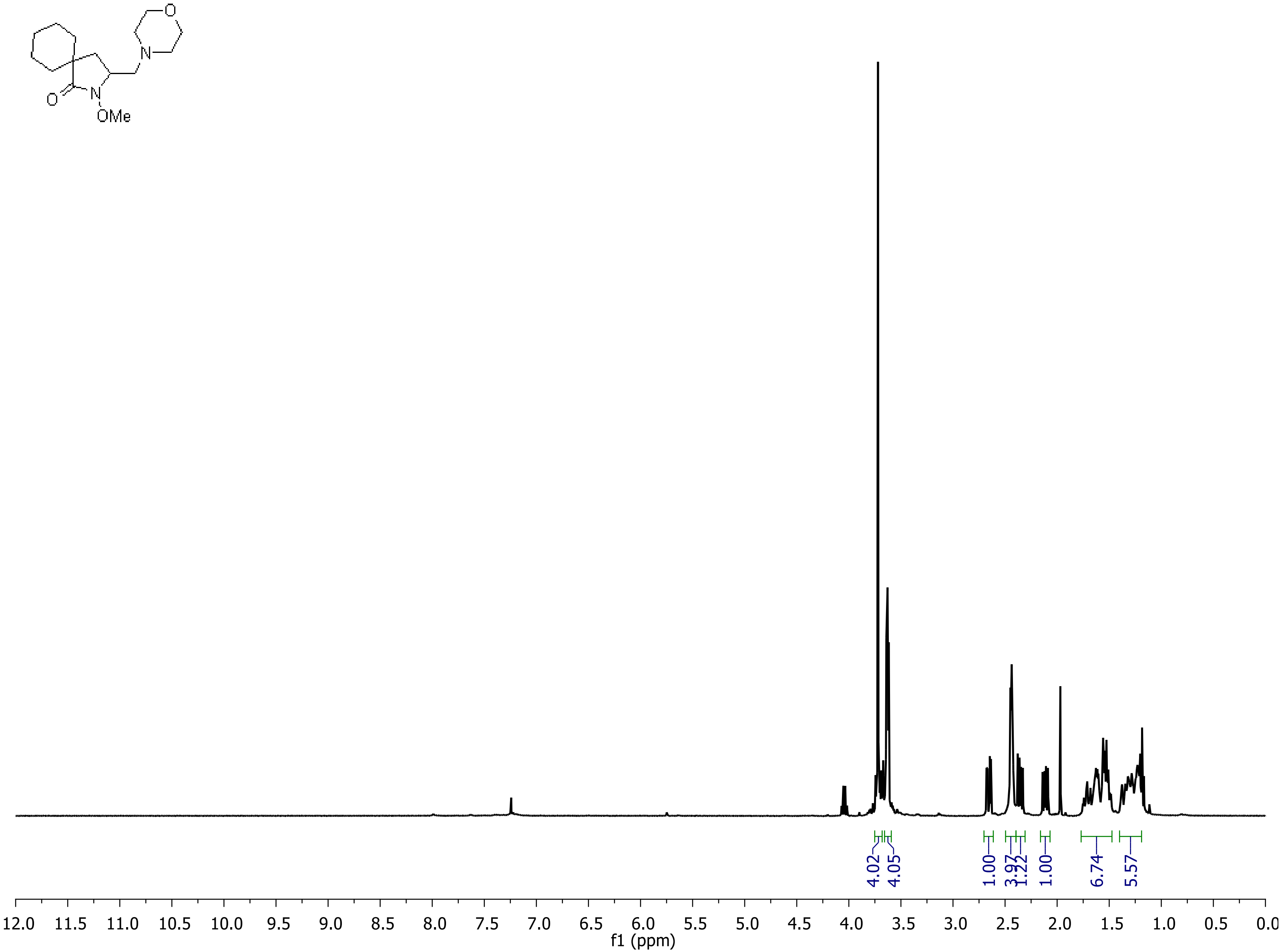
OMe
4.02
4.05
1.00
3.97
1.22
1.00
6.74
5.57
12.0 11.5 11.0 10.5 10.0 9.5 9.0 8.5 8.0 7.5 7.0 6.5 6.0 5.5 5.0 4.5 4.0 3.5 3.0 2.5 2.0 1.5 1.0 0.5 0.0
f1 (ppm)

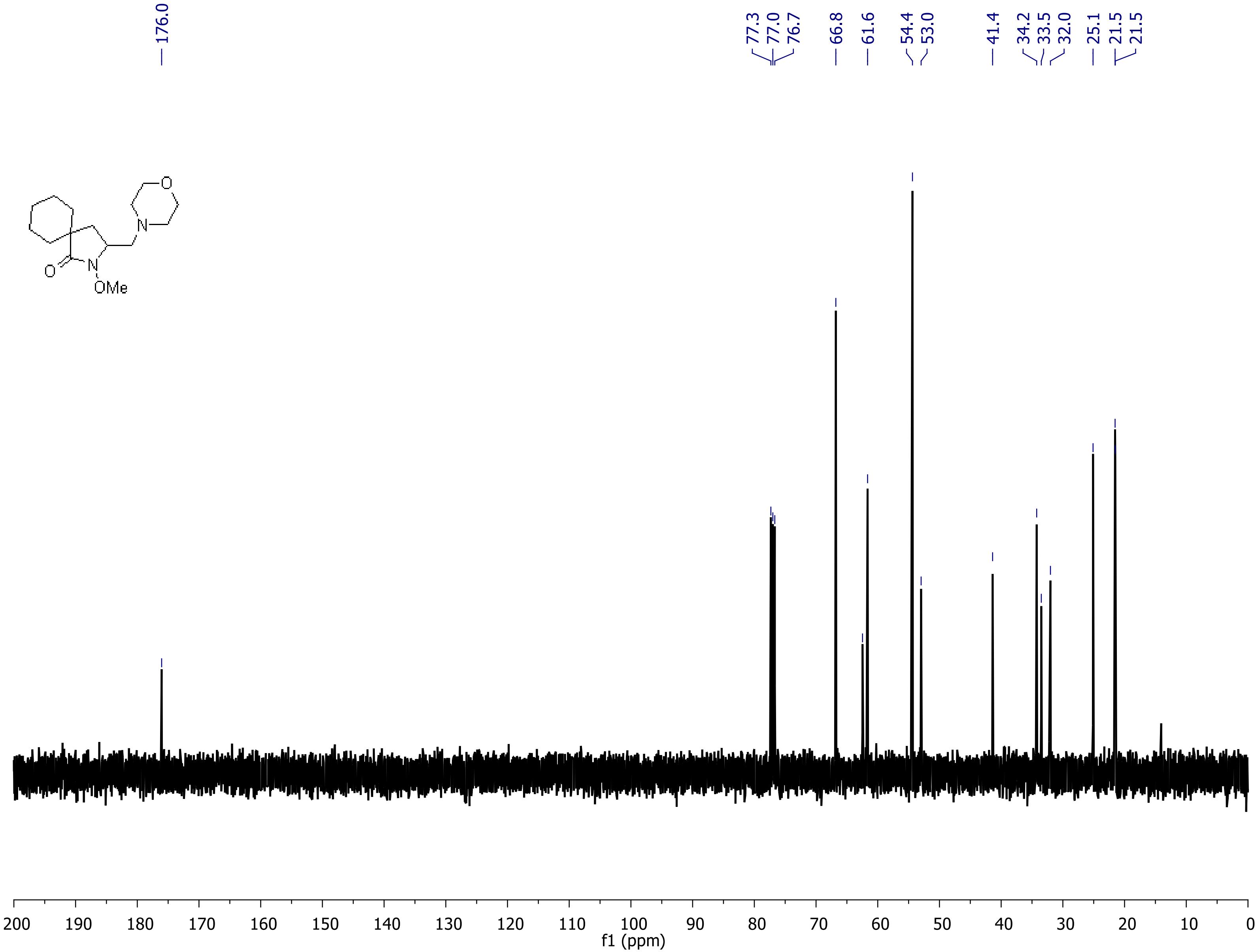
176.0
77.3
77.0
76.7
66.8
61.6
54.4
53.0
41.4
34.2
33.5
32.0
25.1
21.5
21.5
O
N
O
N
OMe
200 190 180 170 160 150 140 130 120 110 100 90 80 70 60 50 40 30 20 10 0
f1 (ppm)

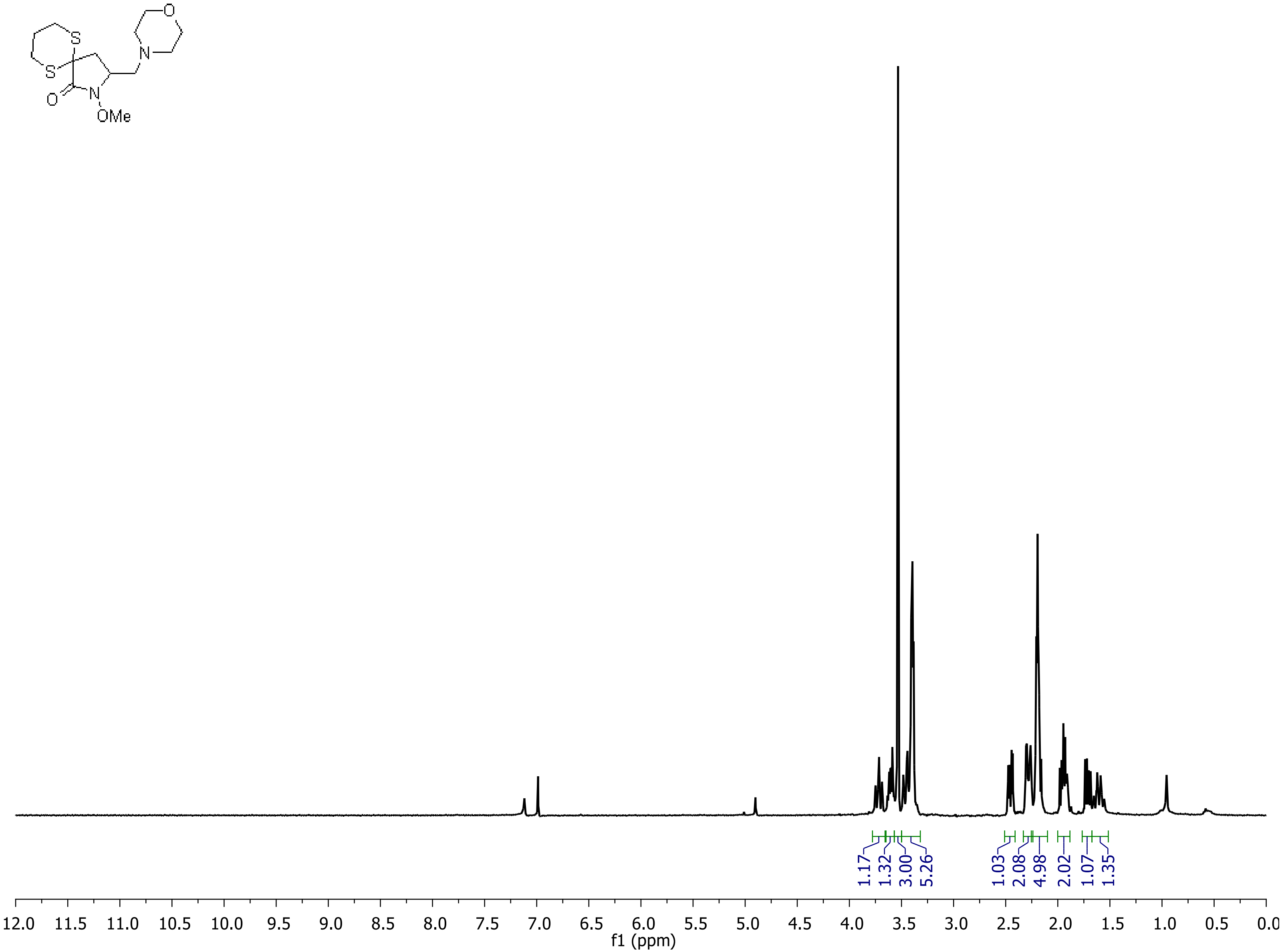
O
N
S
S
O
N
OMe
1.17
1.32
3.00
5.26
1.03
2.08
4.98
2.02
1.07
1.35
12.0 11.5 11.0 10.5 10.0 9.5 9.0 8.5 8.0 7.5 7.0 6.5 6.0 5.5 5.0 4.5 4.0 3.5 3.0 2.5 2.0 1.5 1.0 0.5 0.0
f1 (ppm)

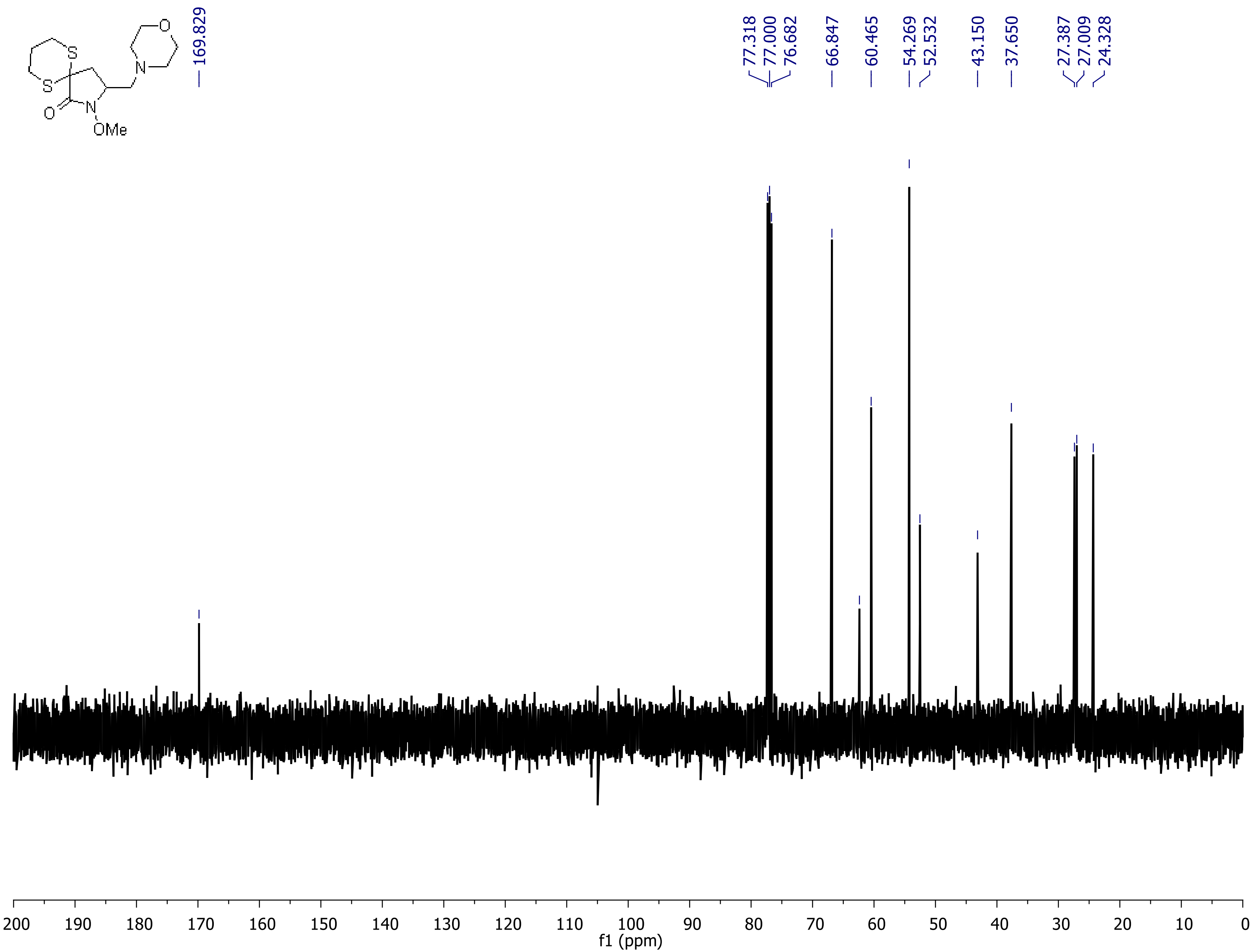
S
S
O
N
OMe
O
N
169.829
77.318
77.000
76.682
66.847
60.465
54.269
52.532
43.150
37.650
27.387
27.009
24.328
200 190 180 170 160 150 140 130 120 110 100 90 80 70 60 50 40 30 20 10 0
f1 (ppm)

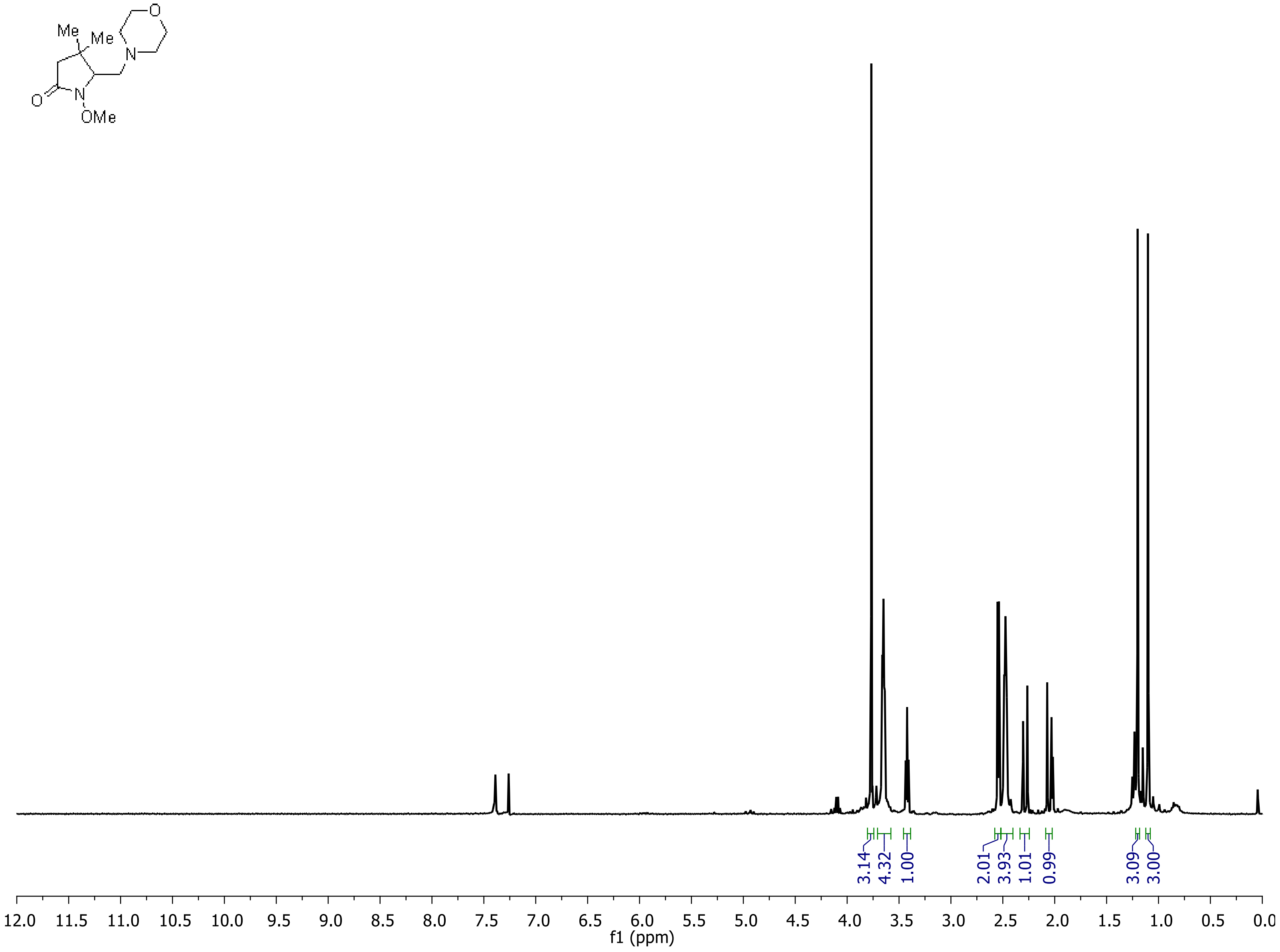

Me
Me
O
N
O
N
OMe
3.14
4.32
1.00
2.01
3.93
1.01
0.99
3.09
3.00
12.0 11.5 11.0 10.5 10.0 9.5 9.0 8.5 8.0 7.5 7.0 6.5 6.0 5.5 5.0 4.5 4.0 3.5 3.0 2.5 2.0 1.5 1.0 0.5 0.0
f1 (ppm)

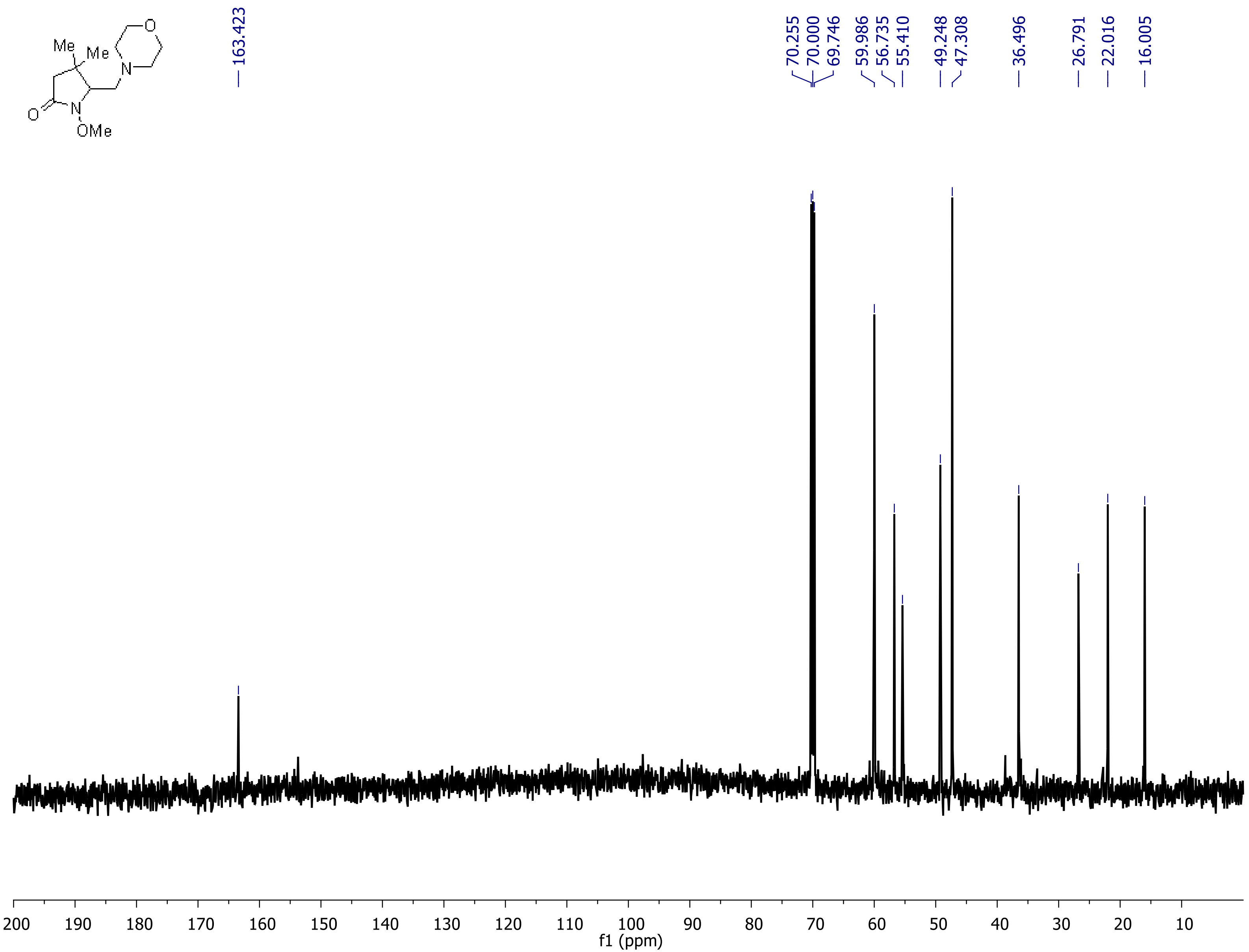

Me
Me
O
N
O
N
OMe
163.423
70.255
70.000
69.746
59.986
56.735
55.410
49.248
47.308
36.496
26.791
22.016
16.005
200 190 180 170 160 150 140 130 120 110 100 90 80 70 60 50 40 30 20 10
f1 (ppm)

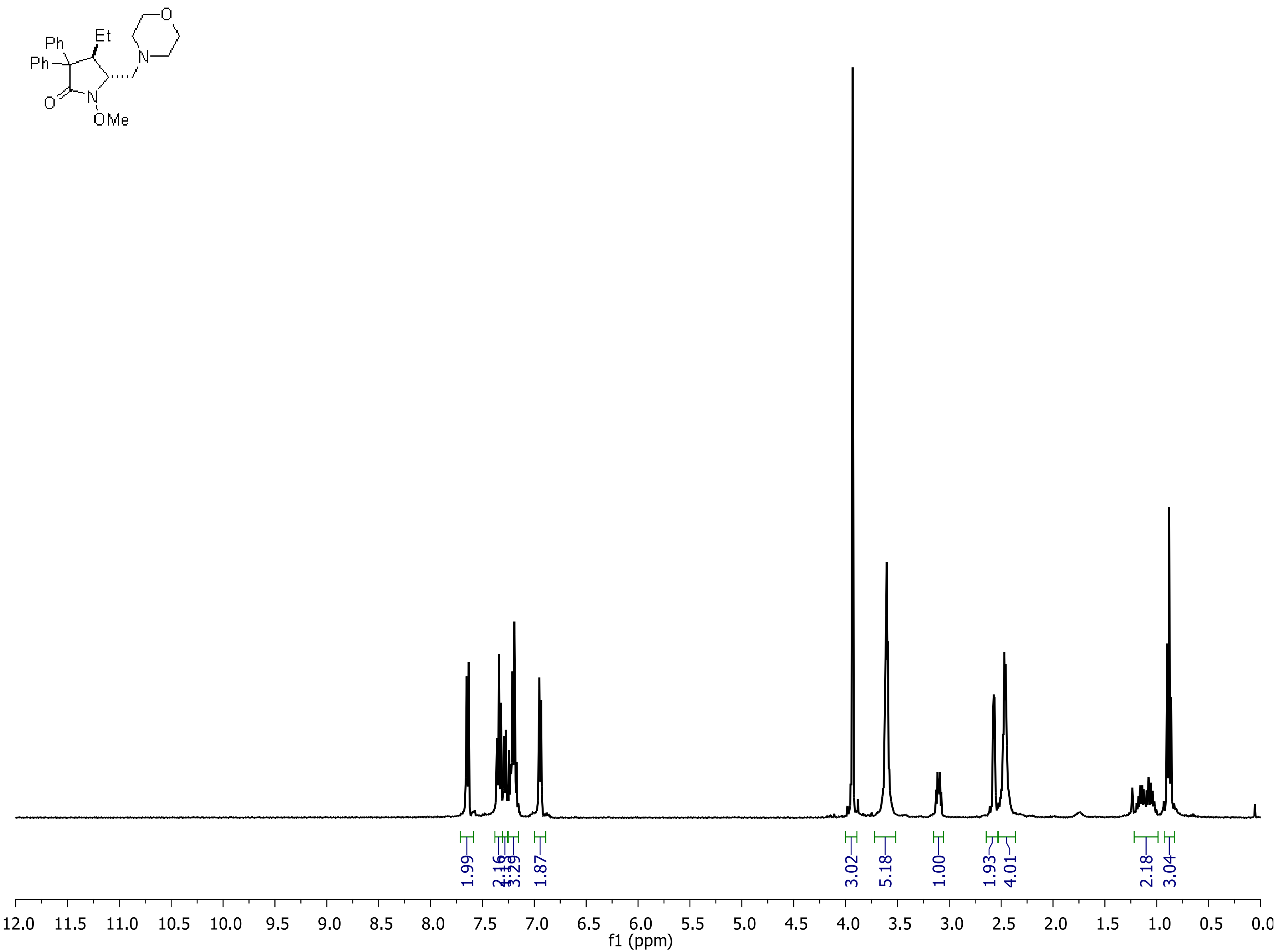
Ph
Ph
Et
O
N
O
N
OMe
1.99
2.16
4.13
3.29
1.87
3.02
5.18
1.00
1.93
4.01
2.18
3.04
12.0 11.5 11.0 10.5 10.0 9.5 9.0 8.5 8.0 7.5 7.0 6.5 6.0 5.5 5.0 4.5 4.0 3.5 3.0 2.5 2.0 1.5 1.0 0.5 0.0
f1 (ppm)

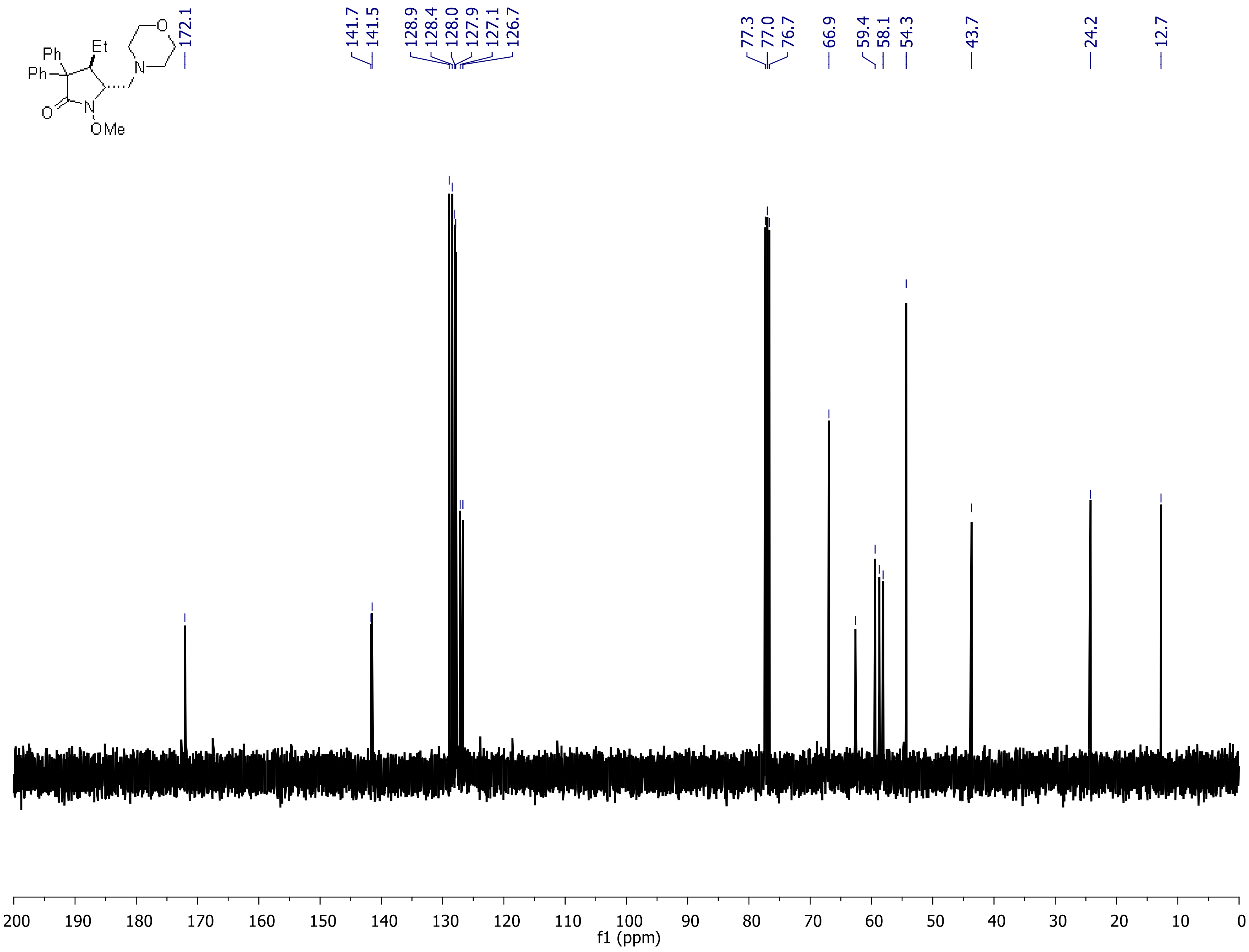

Ph
Ph
Et
O
N
N
OMe
O
172.1
141.7
141.5
128.9
128.4
128.0
127.9
127.1
126.7
77.3
77.0
76.7
66.9
59.4
58.1
54.3
43.7
24.2
12.7
200 190 180 170 160 150 140 130 120 110 100 90 80 70 60 50 40 30 20 10 0
f1 (ppm)

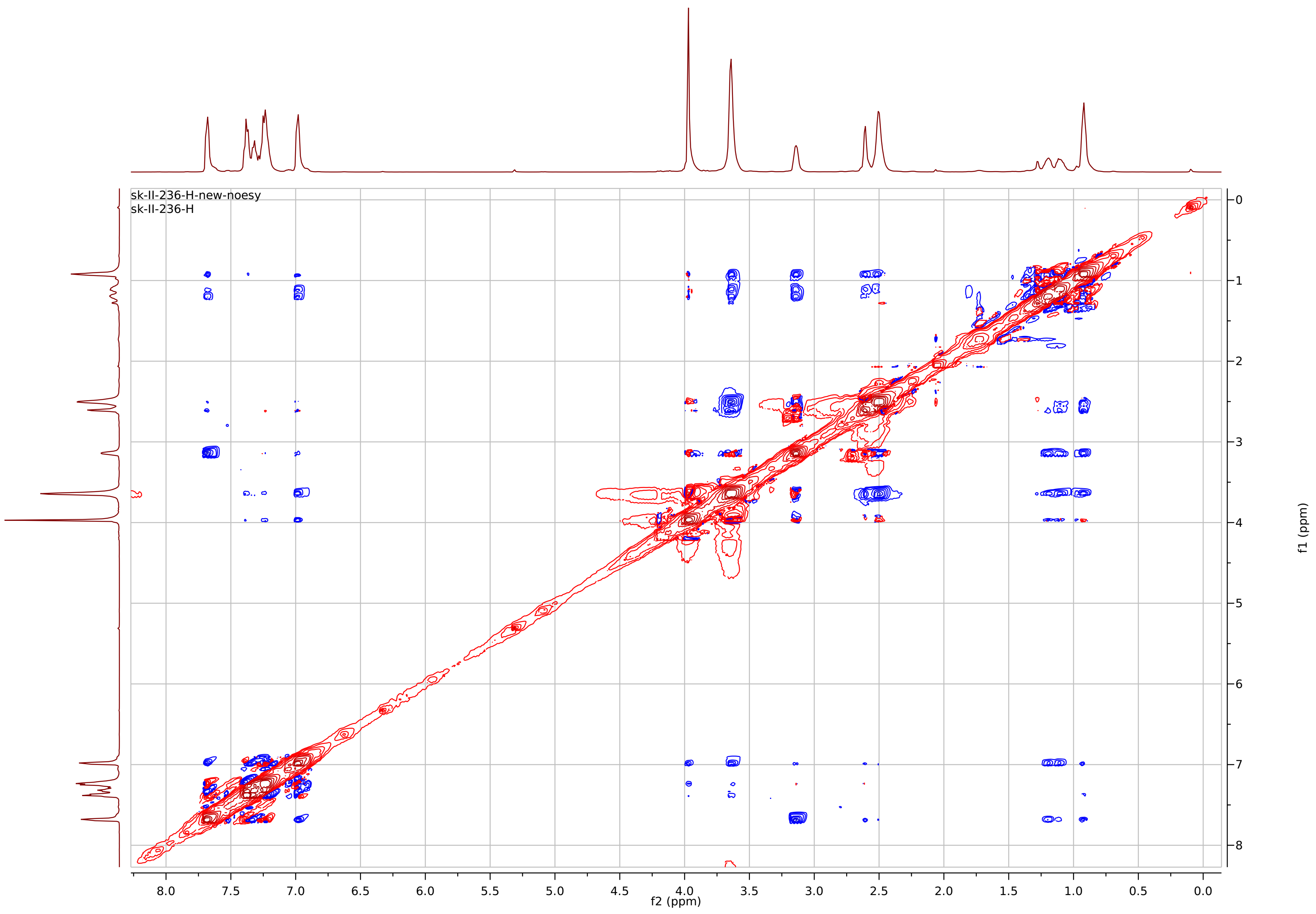

sk-II-236-H-new-noesy
sk-II-236-H
f2 (ppm)
f1 (ppm)

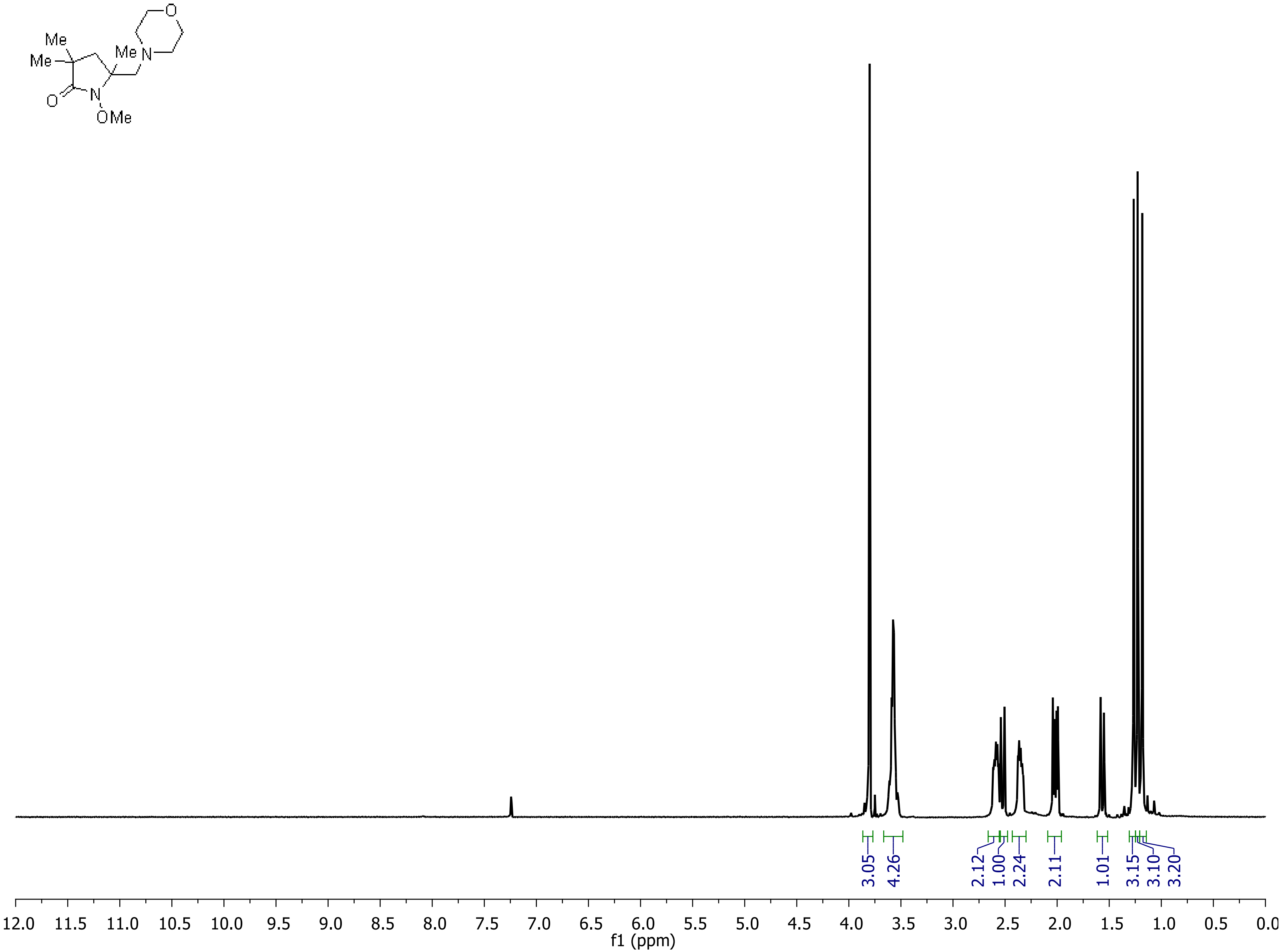

Me
Me
Me
N
O
O
N
OMe
3.05
4.26
2.12
1.00
2.24
2.11
1.01
3.15
3.10
3.20
12.0 11.5 11.0 10.5 10.0 9.5 9.0 8.5 8.0 7.5 7.0 6.5 6.0 5.5 5.0 4.5 4.0 3.5 3.0 2.5 2.0 1.5 1.0 0.5 0.0
f1 (ppm)

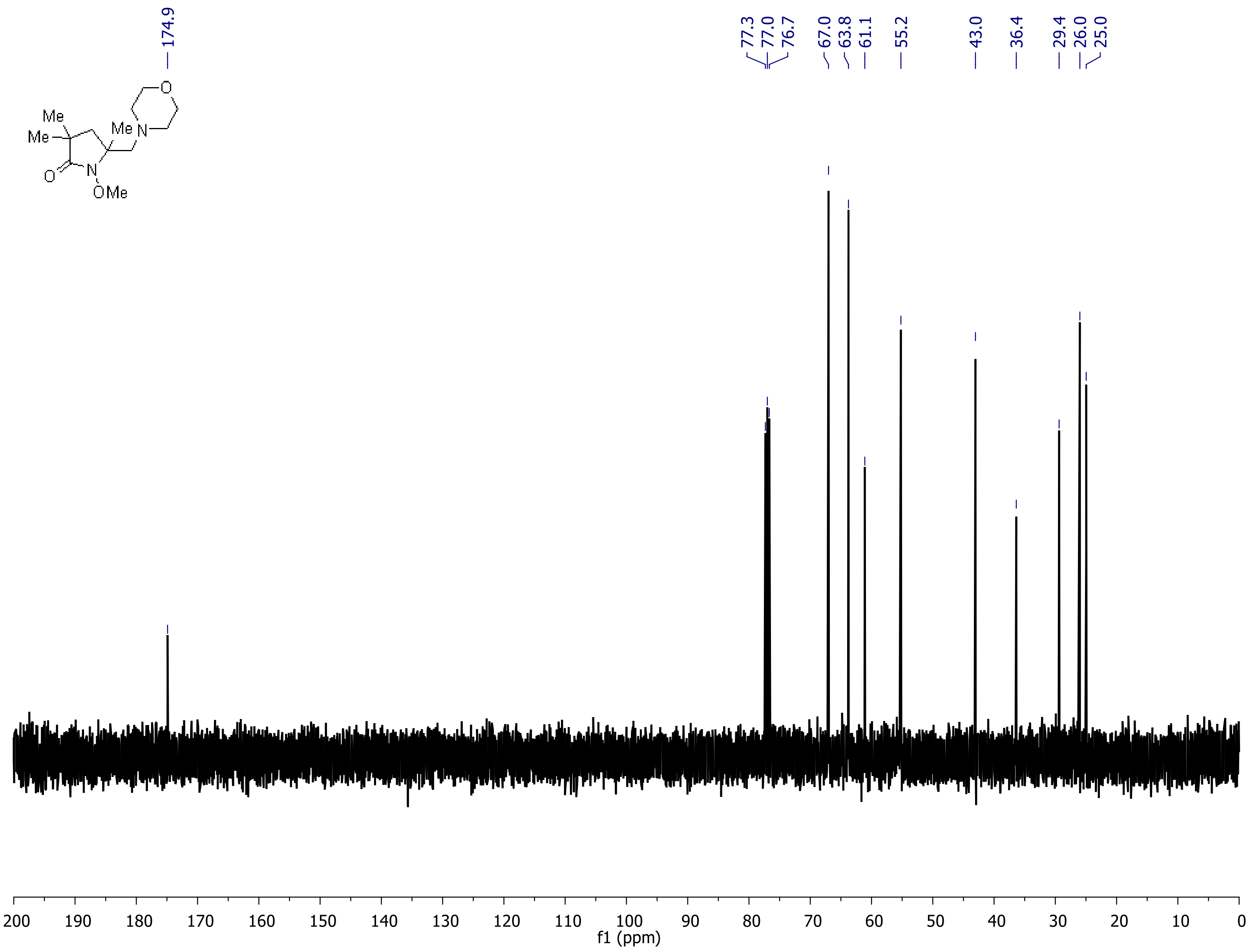

174.9
77.3
77.0
76.7
67.0
63.8
61.1
55.2
43.0
36.4
29.4
26.0
25.0
Me
Me
Me
N
O
O
N
OMe
200
190
180
170
160
150
140
130
120
110
100
90
80
70
60
50
40
30
20
10
0
f1 (ppm)

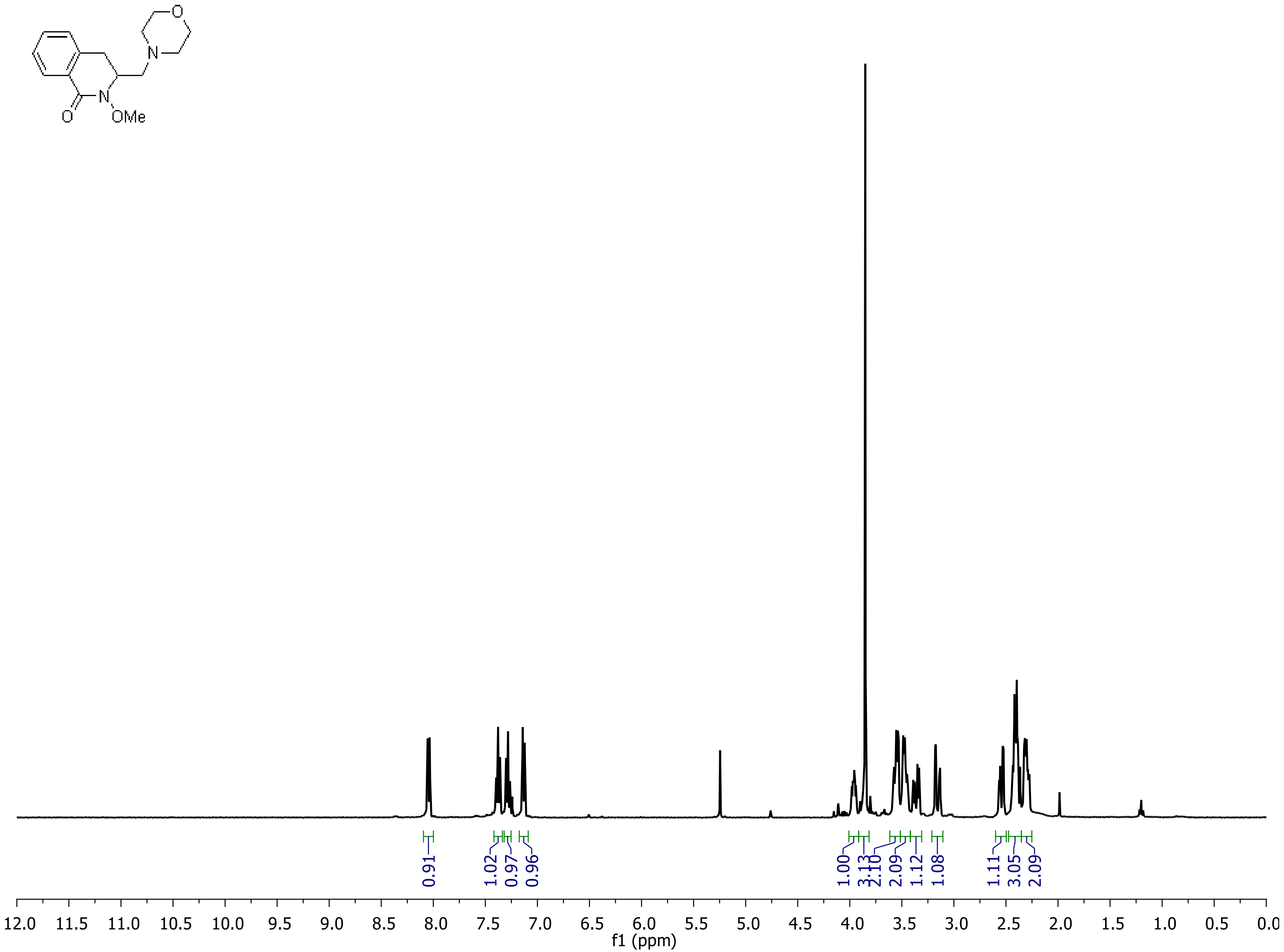
O
N
N
OMe
O
0.91
1.02
0.97
0.96
1.00
3.13
2.10
2.09
1.12
1.08
1.11
3.05
2.09
12.0
11.5
11.0
10.5
10.0
9.5
9.0
8.5
8.0
7.5
7.0
6.5
6.0
5.5
5.0
4.5
4.0
3.5
3.0
2.5
2.0
1.5
1.0
0.5
0.0
f1 (ppm)

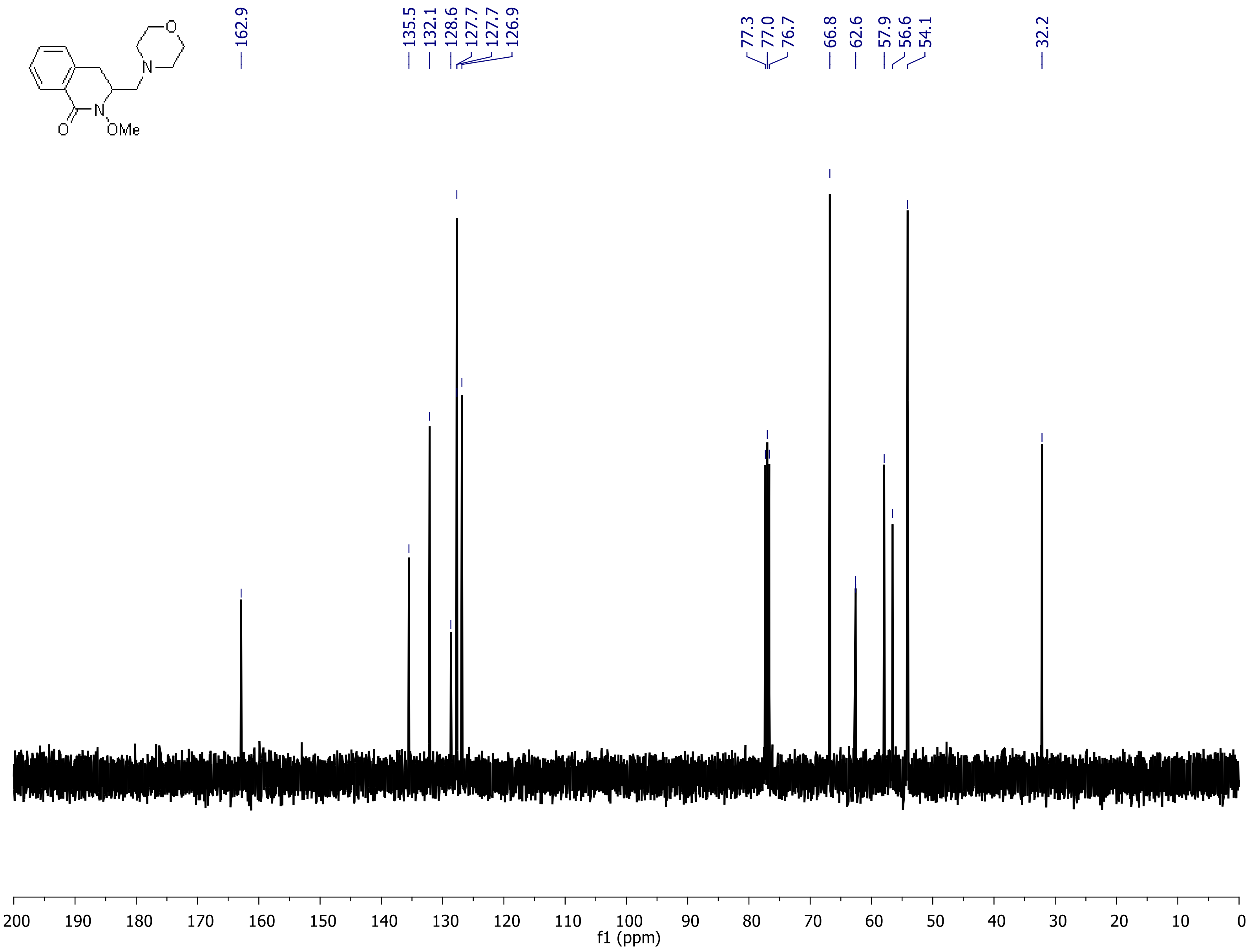

OMe
162.9
135.5
132.1
128.6
127.7
127.7
126.9
77.3
77.0
76.7
66.8
62.6
57.9
56.6
54.1
32.2
200
190
180
170
160
150
140
130
120
110
100
90
80
70
60
50
40
30
20
10
0
f1 (ppm)

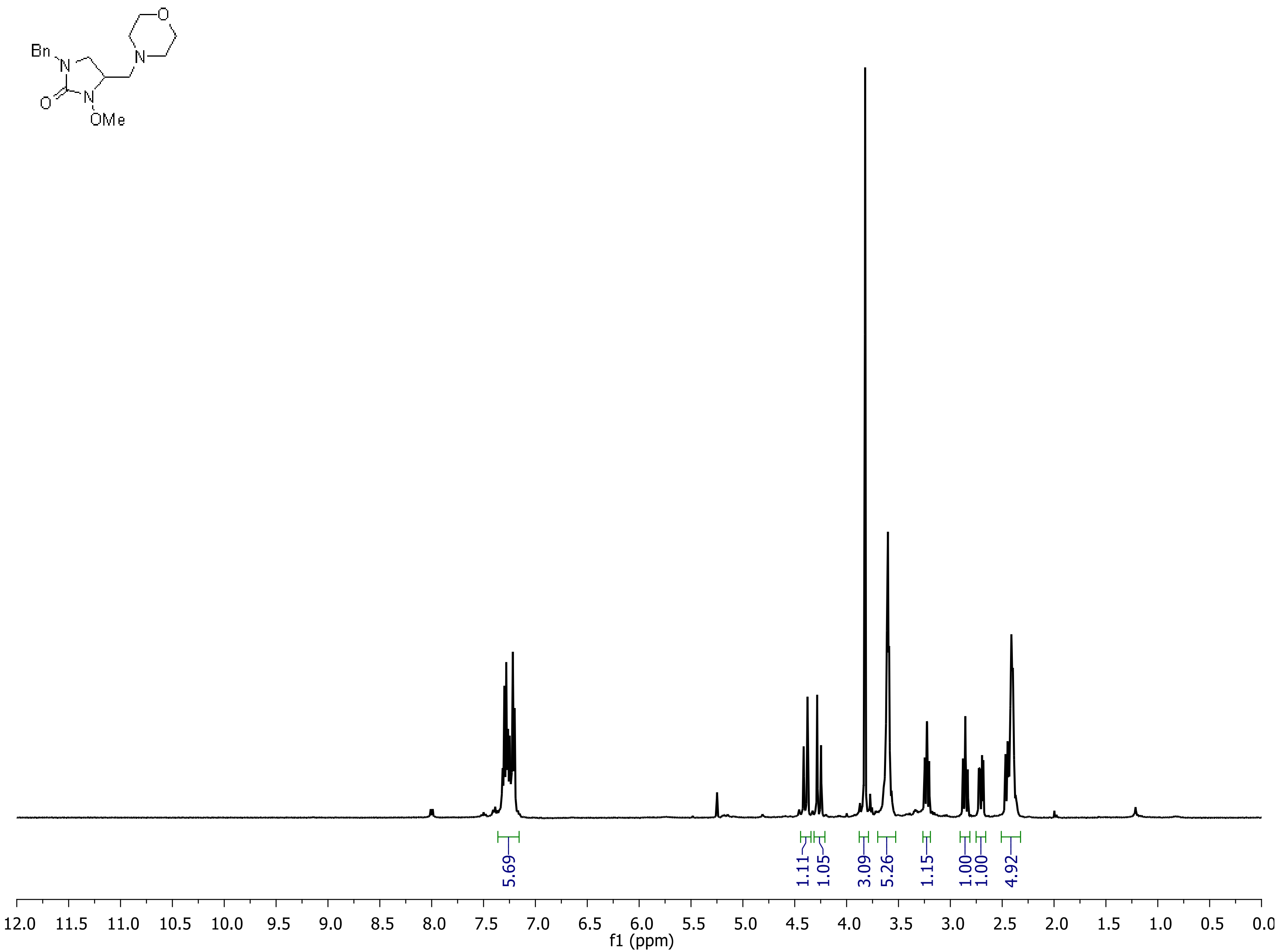
Bn
N
N
O
OMe
O
N
5.69
1.11
1.05
3.09
5.26
1.15
1.00
1.00
4.92
12.0 11.5 11.0 10.5 10.0 9.5 9.0 8.5 8.0 7.5 7.0 6.5 6.0 5.5 5.0 4.5 4.0 3.5 3.0 2.5 2.0 1.5 1.0 0.5 0.0
f1 (ppm)

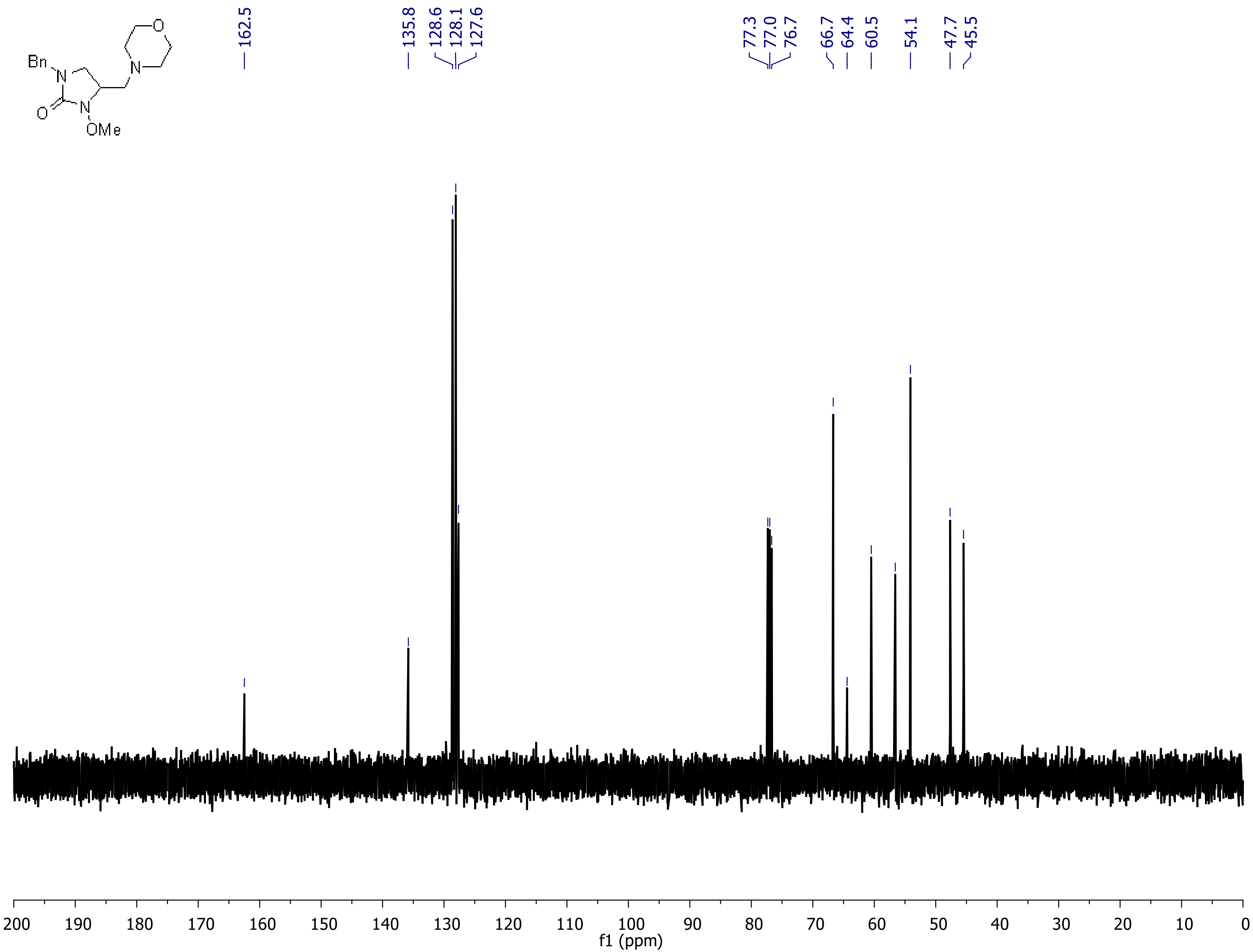
Bn
N
O
N
OMe
O
N
162.5
135.8
128.6
128.1
127.6
77.3
77.0
76.7
66.7
64.4
60.5
54.1
47.7
45.5
200 190 180 170 160 150 140 130 120 110 100 90 80 70 60 50 40 30 20 10 0
f1 (ppm)

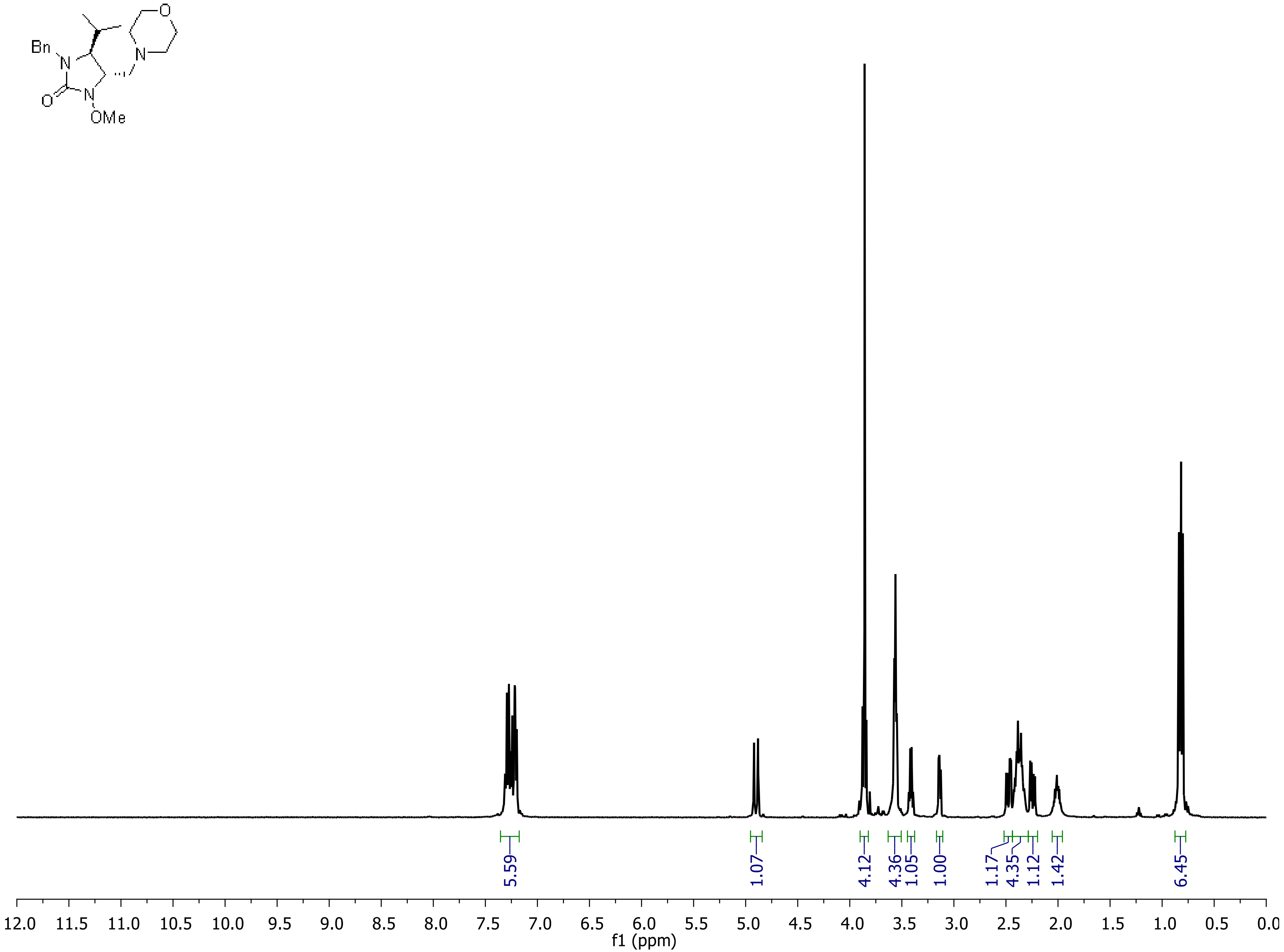
Bn
N
O
N
OMe
N
O
5.59
1.07
4.12
4.36
1.05
1.00
1.17
4.35
1.12
1.42
6.45
12.0
11.5
11.0
10.5
10.0
9.5
9.0
8.5
8.0
7.5
7.0
6.5
6.0
5.5
5.0
4.5
4.0
3.5
3.0
2.5
2.0
1.5
1.0
0.5
0.0
f1 (ppm)

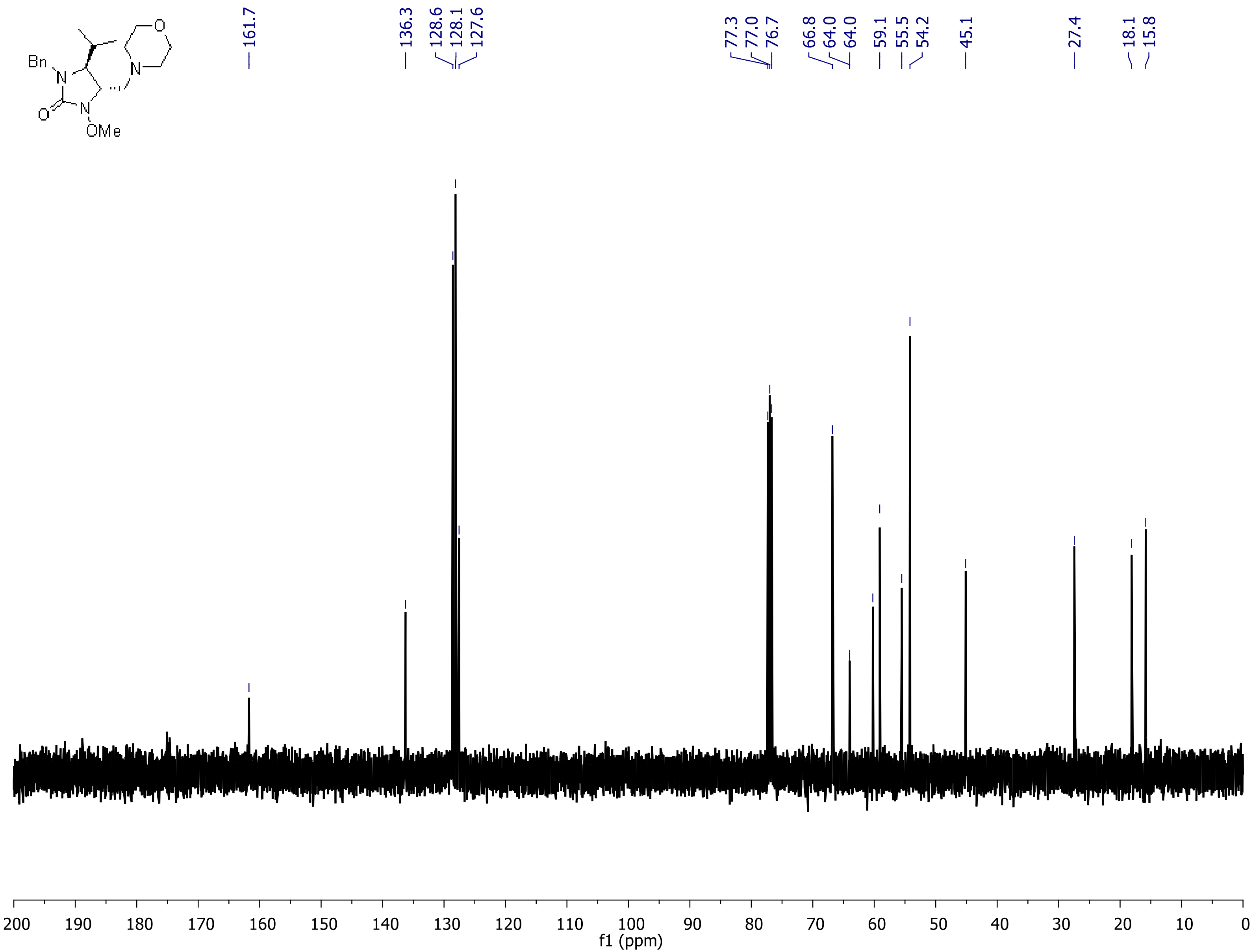

Bn
N
O
N
OMe
161.7
136.3
128.6
128.1
127.6
77.3
77.0
76.7
66.8
64.0
64.0
59.1
55.5
54.2
45.1
27.4
18.1
15.8
200
190
180
170
160
150
140
130
120
110
100
90
80
70
60
50
40
30
20
10
0
f1 (ppm)

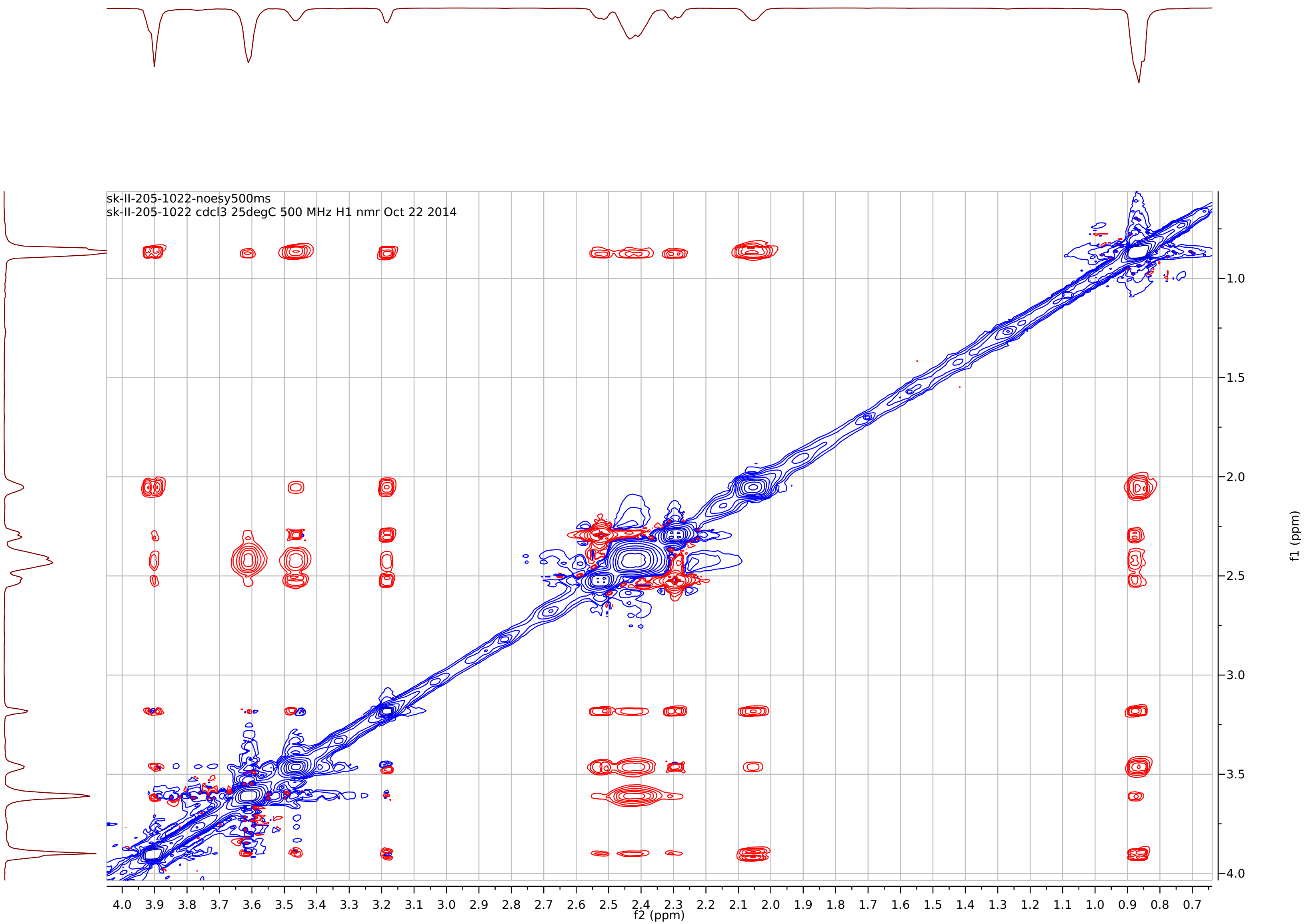
sk-II-205-1022-noesy500ms
sk-II-205-1022 cdcl3 25degC 500 MHz H1 nmr Oct 22 2014
f2 (ppm)
f1 (ppm)

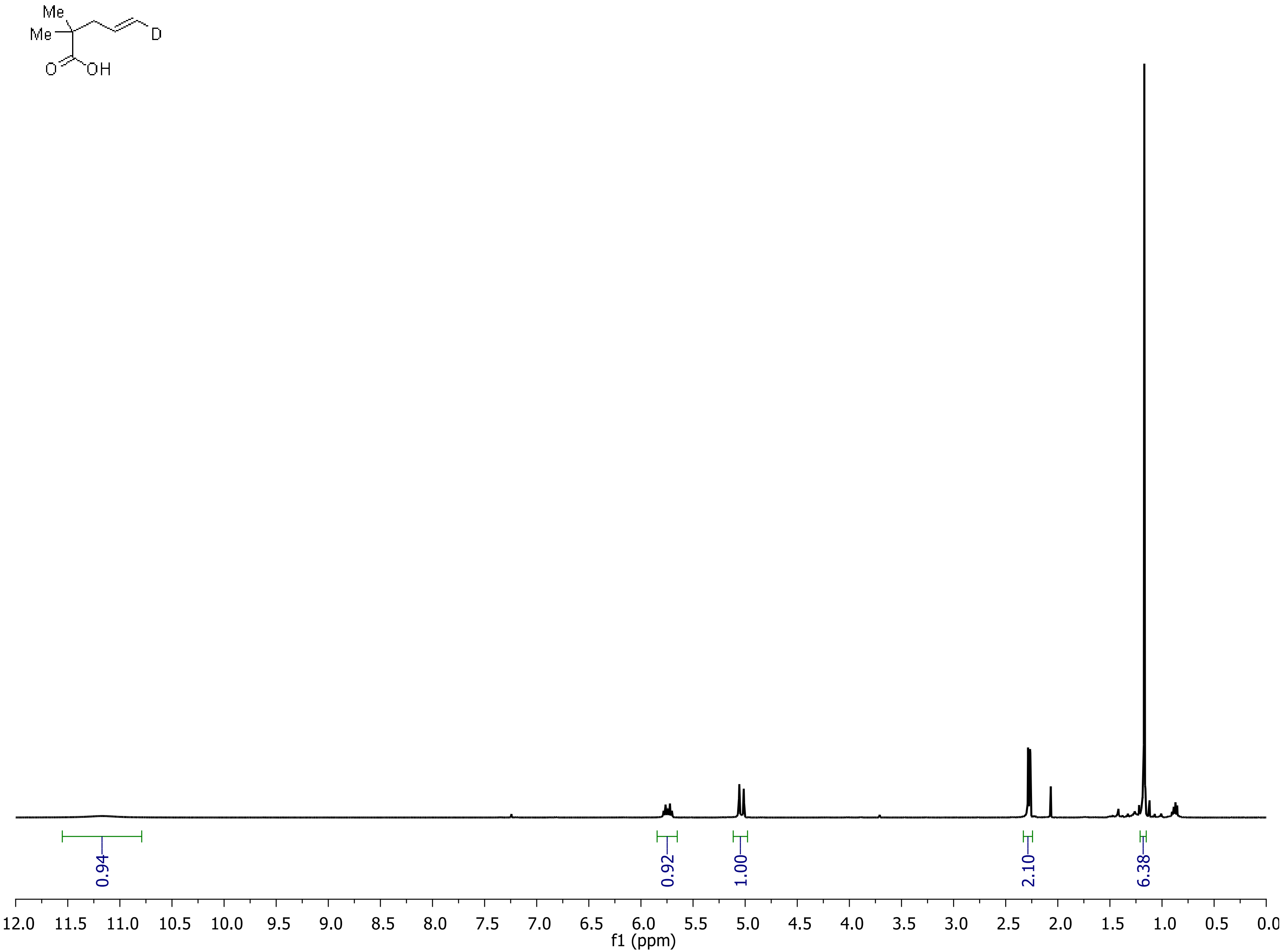
Me
Me
D
O
OH
0.94
0.92
1.00
2.10
6.38
12.0 11.5 11.0 10.5 10.0 9.5 9.0 8.5 8.0 7.5 7.0 6.5 6.0 5.5 5.0 4.5 4.0 3.5 3.0 2.5 2.0 1.5 1.0 0.5 0.0
f1 (ppm)

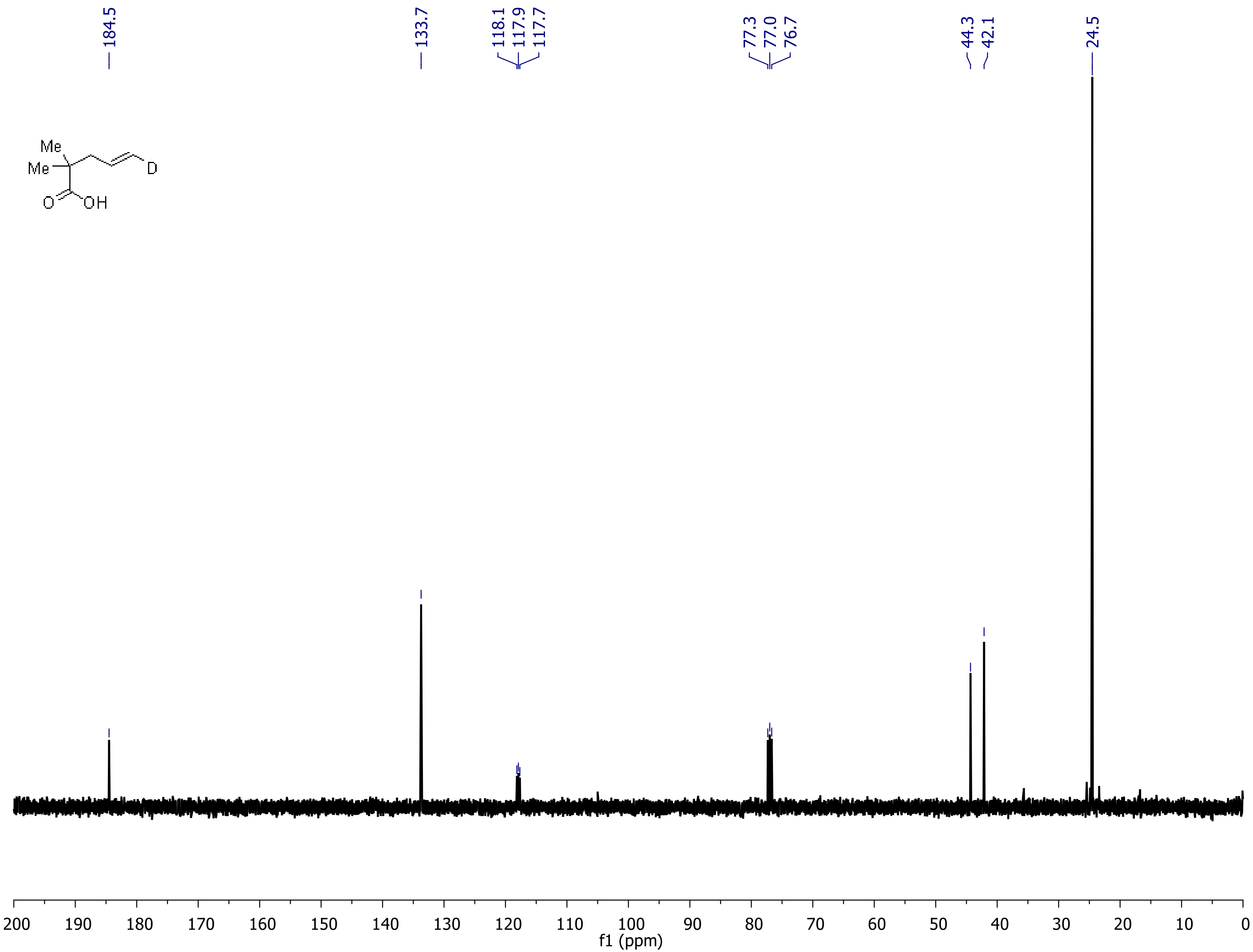
184.5
133.7
118.1
117.9
117.7
77.3
77.0
76.7
44.3
42.1
24.5
Me
Me
D
O
OH
200 190 180 170 160 150 140 130 120 110 100 90 80 70 60 50 40 30 20 10 0
f1 (ppm)

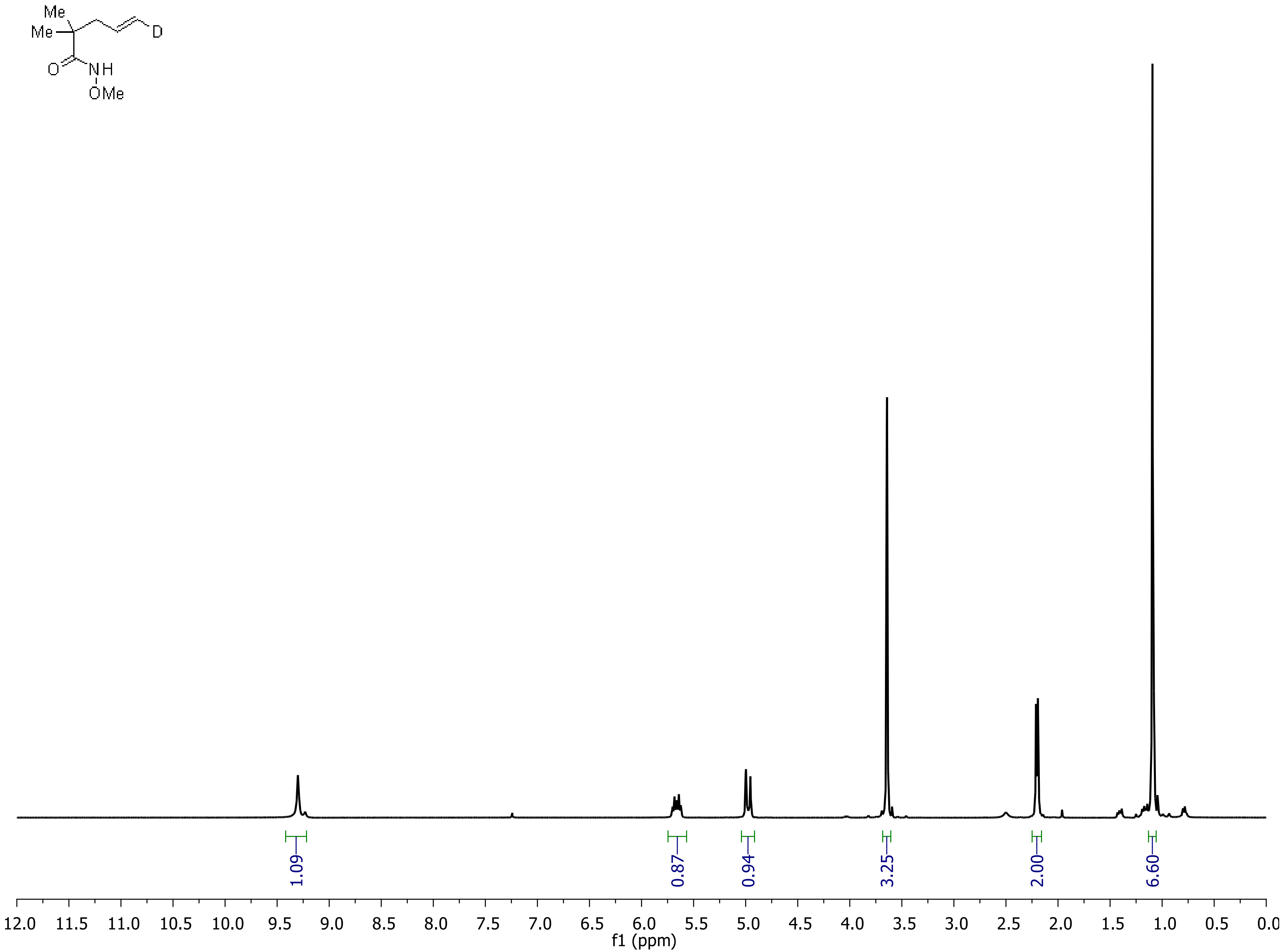
Me
Me
D
O
NH
OMe
1.09
0.87
0.94
3.25
2.00
6.60
12.0 11.5 11.0 10.5 10.0 9.5 9.0 8.5 8.0 7.5 7.0 6.5 6.0 5.5 5.0 4.5 4.0 3.5 3.0 2.5 2.0 1.5 1.0 0.5 0.0
f1 (ppm)

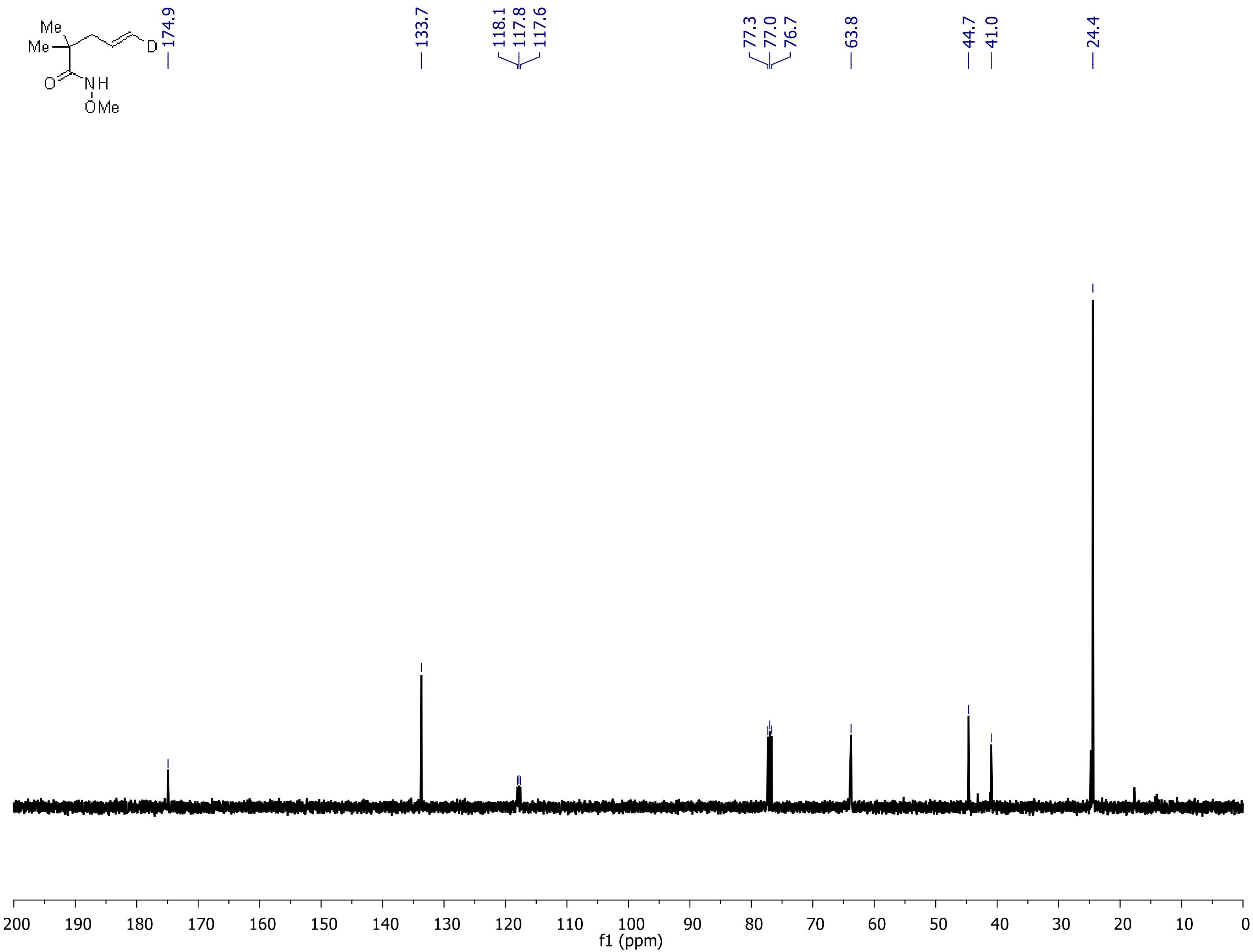

Me
Me
D
O
NH
OMe
174.9
133.7
118.1
117.8
117.6
77.3
77.0
76.7
63.8
44.7
41.0
24.4
200
190
180
170
160
150
140
130
120
110
100
90
80
70
60
50
40
30
20
10
0
f1 (ppm)

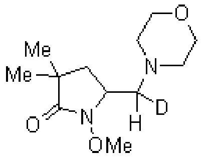

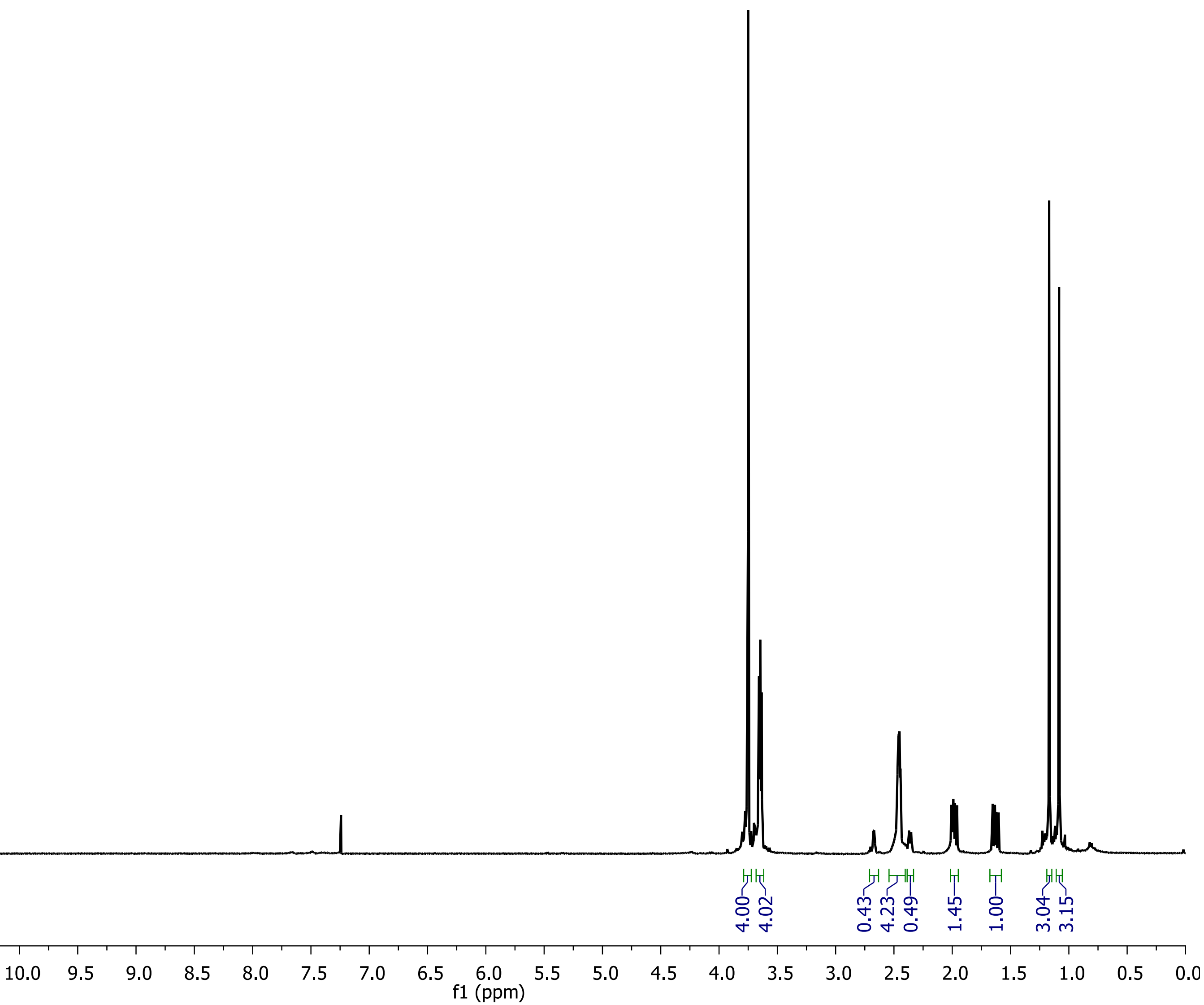

4.00
4.02
0.43
4.23
0.49
1.45
1.00
3.04
3.15
12.0 11.5 11.0 10.5 10.0 9.5 9.0 8.5 8.0 7.5 7.0 6.5 6.0 5.5 5.0 4.5 4.0 3.5 3.0 2.5 2.0 1.5 1.0 0.5 0.0
f1 (ppm)

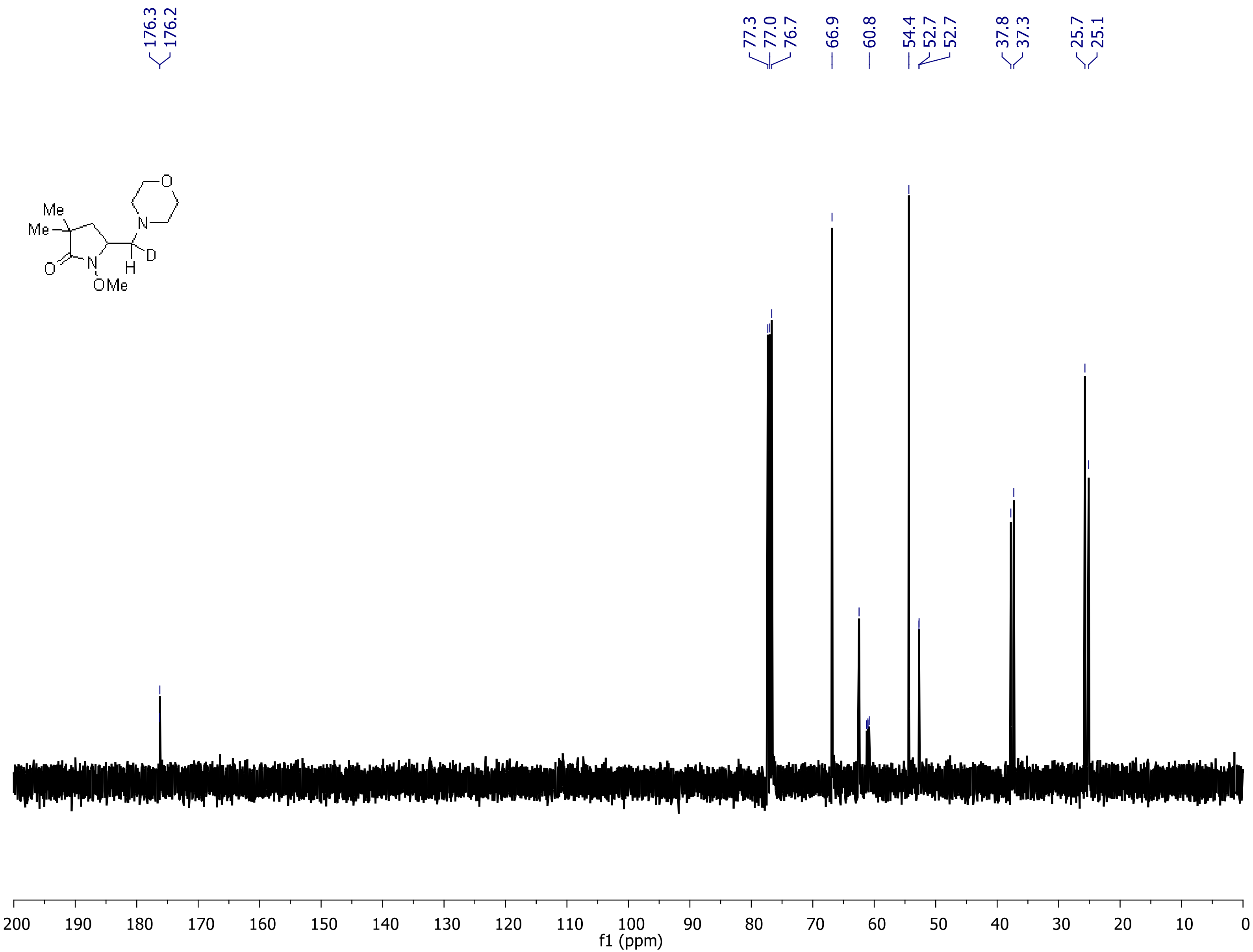
176.3
176.2
77.3
77.0
76.7
66.9
60.8
54.4
52.7
52.7
37.8
37.3
25.7
25.1
Me
Me
O
N
OMe
N
O
H
D
200 190 180 170 160 150 140 130 120 110 100 90 80 70 60 50 40 30 20 10 0
f1 (ppm)

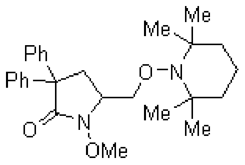

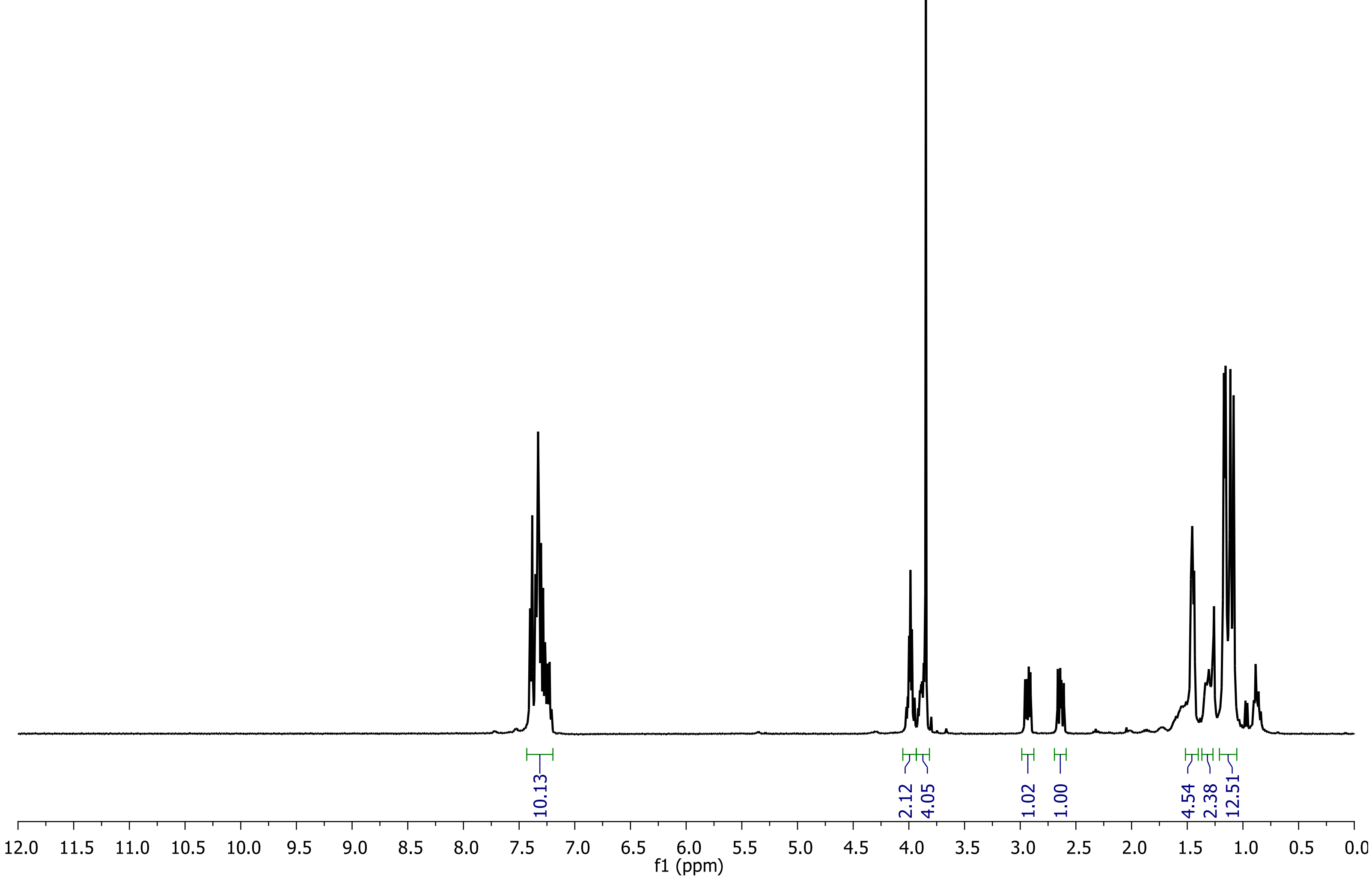
10.13
2.12
4.05
1.02
1.00
4.54
2.38
12.51
12.0 11.5 11.0 10.5 10.0 9.5 9.0 8.5 8.0 7.5 7.0 6.5 6.0 5.5 5.0 4.5 4.0 3.5 3.0 2.5 2.0 1.5 1.0 0.5 0.0
f1 (ppm)

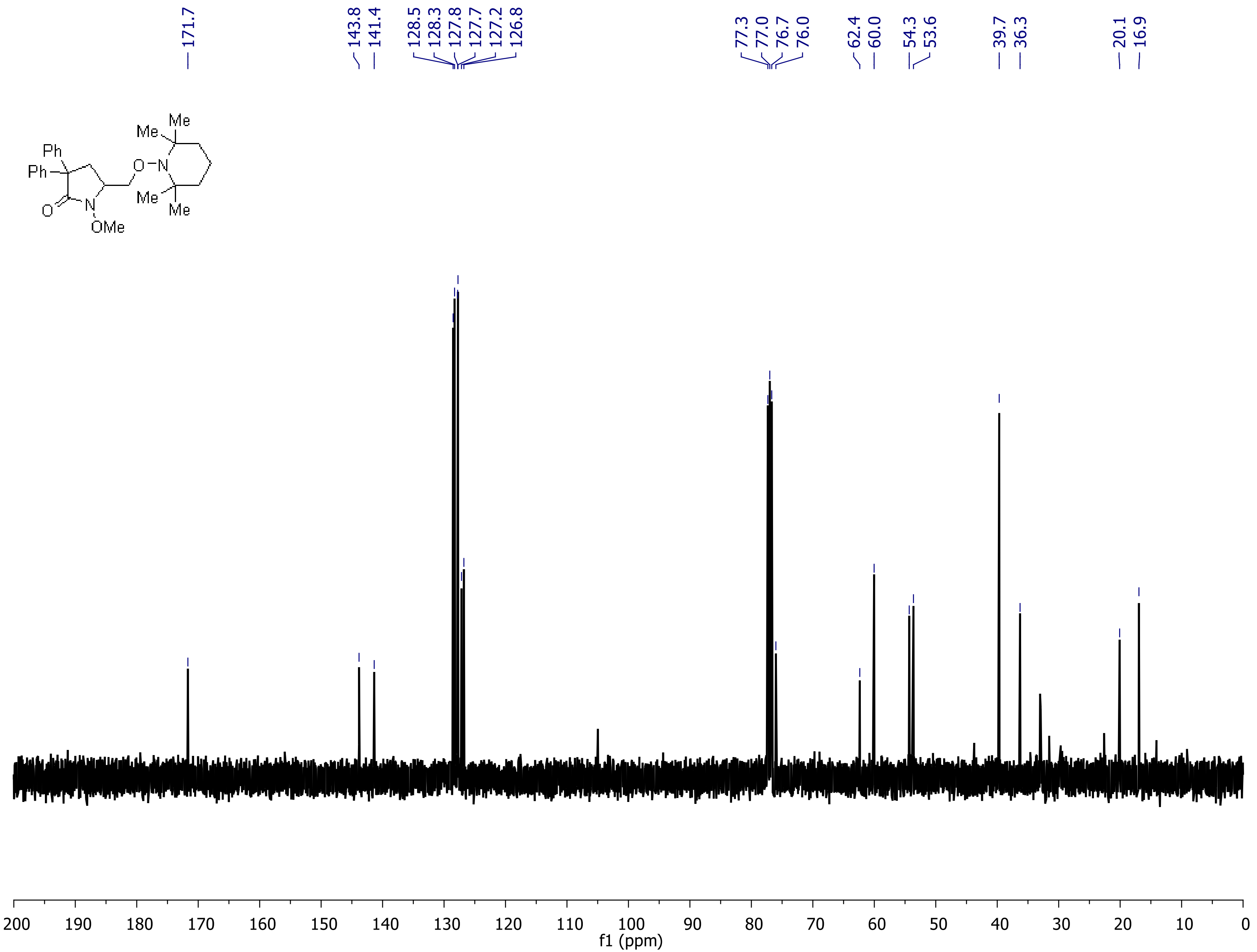
171.7
143.8
141.4
128.5
128.3
127.8
127.7
127.2
126.8
77.3
77.0
76.7
76.0
62.4
60.0
54.3
53.6
39.7
36.3
20.1
16.9
Ph
Ph
O
N
OMe
O
N
Me
Me
Me
Me
200 190 180 170 160 150 140 130 120 110 100 90 80 70 60 50 40 30 20 10 0
f1 (ppm)

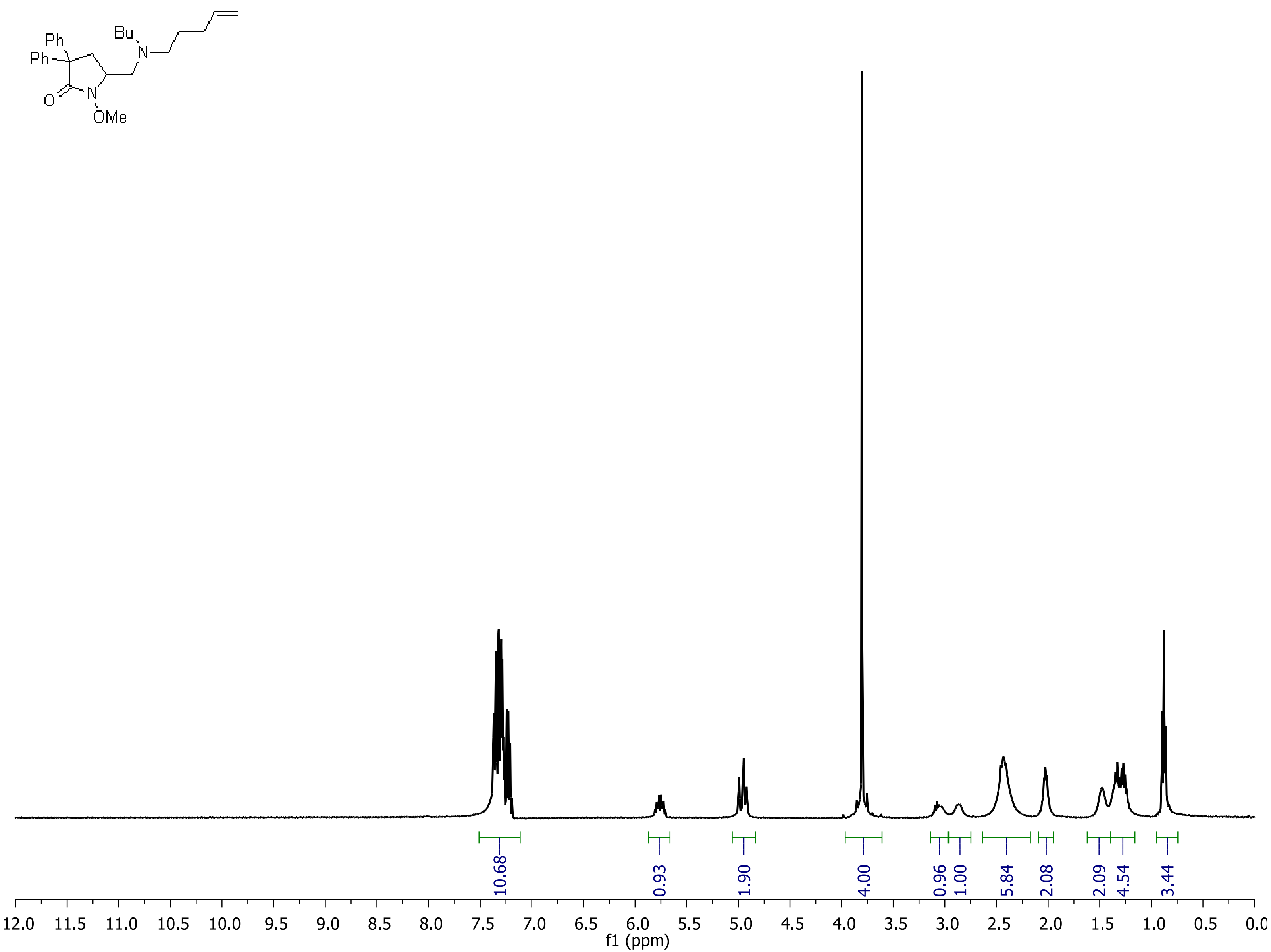
Ph
Ph
Bu
N
O
N
OMe
10.68
0.93
1.90
4.00
0.96
1.00
5.84
2.08
2.09
4.54
3.44
12.0 11.5 11.0 10.5 10.0 9.5 9.0 8.5 8.0 7.5 7.0 6.5 6.0 5.5 5.0 4.5 4.0 3.5 3.0 2.5 2.0 1.5 1.0 0.5 0.0
f1 (ppm)

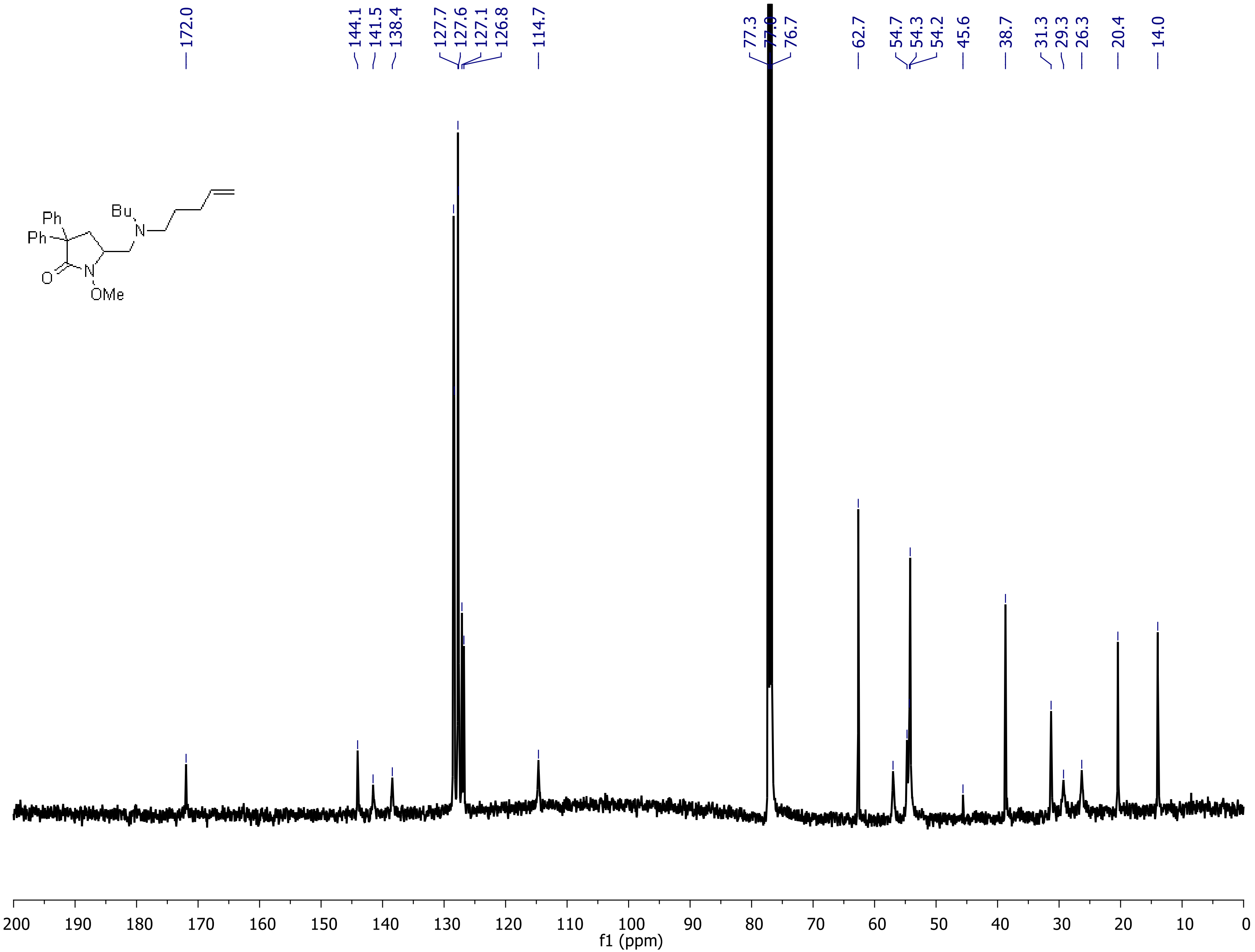
172.0
144.1
141.5
138.4
127.7
127.6
127.1
126.8
114.7
77.3
76.7
62.7
54.7
54.3
54.2
45.6
38.7
31.3
29.3
26.3
20.4
14.0
Ph
Ph
Bu
N
O
N
OMe
200 190 180 170 160 150 140 130 120 110 100 90 80 70 60 50 40 30 20 10 0
f1 (ppm)

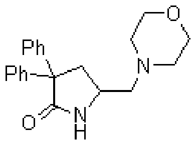
Ph
Ph
O
N
H
N
O

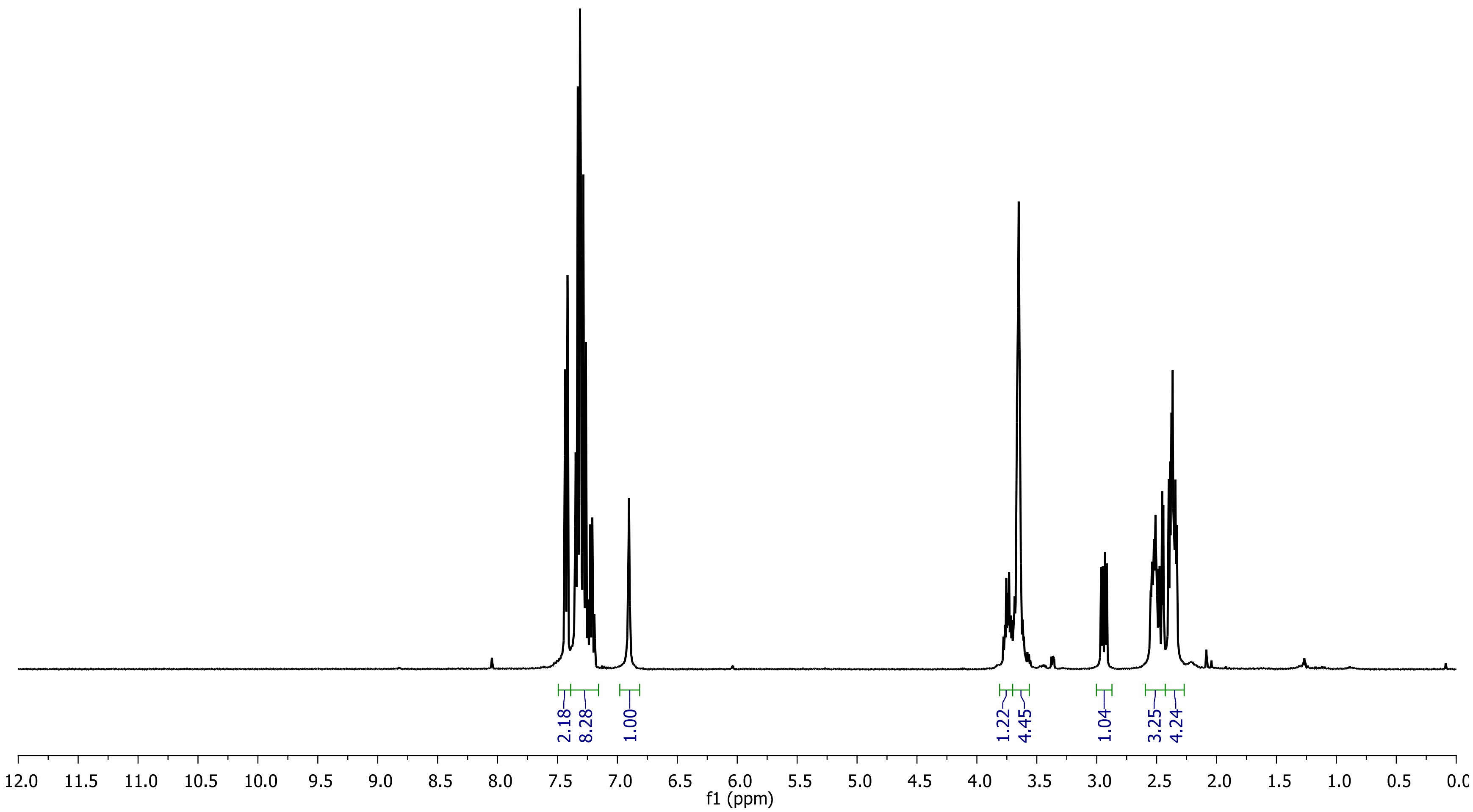
2.18
8.28
1.00
1.22
4.45
1.04
3.25
4.24
12.0
11.5
11.0
10.5
10.0
9.5
9.0
8.5
8.0
7.5
7.0
6.5
6.0
5.5
5.0
4.5
4.0
3.5
3.0
2.5
2.0
1.5
1.0
0.5
0.0
f1 (ppm)

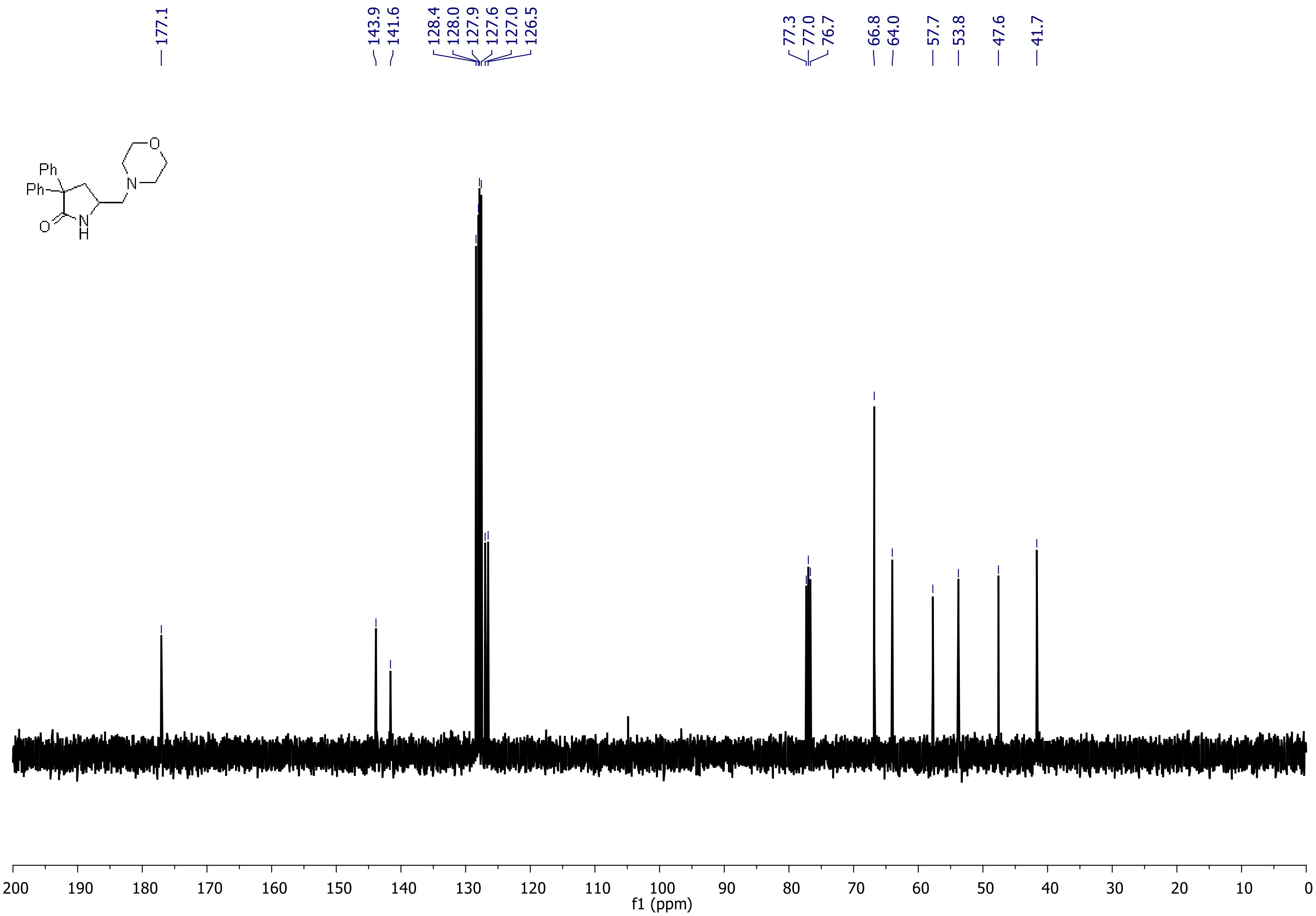
177.1
143.9
141.6
128.4
128.0
127.9
127.6
127.0
126.5
77.3
77.0
76.7
66.8
64.0
57.7
53.8
47.6
41.7
Ph
Ph
O
N
H
N
O
200 190 180 170 160 150 140 130 120 110 100 90 80 70 60 50 40 30 20 10 0
f1 (ppm)

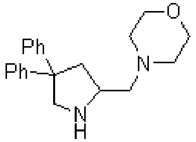
Ph
Ph
O
N
N
H

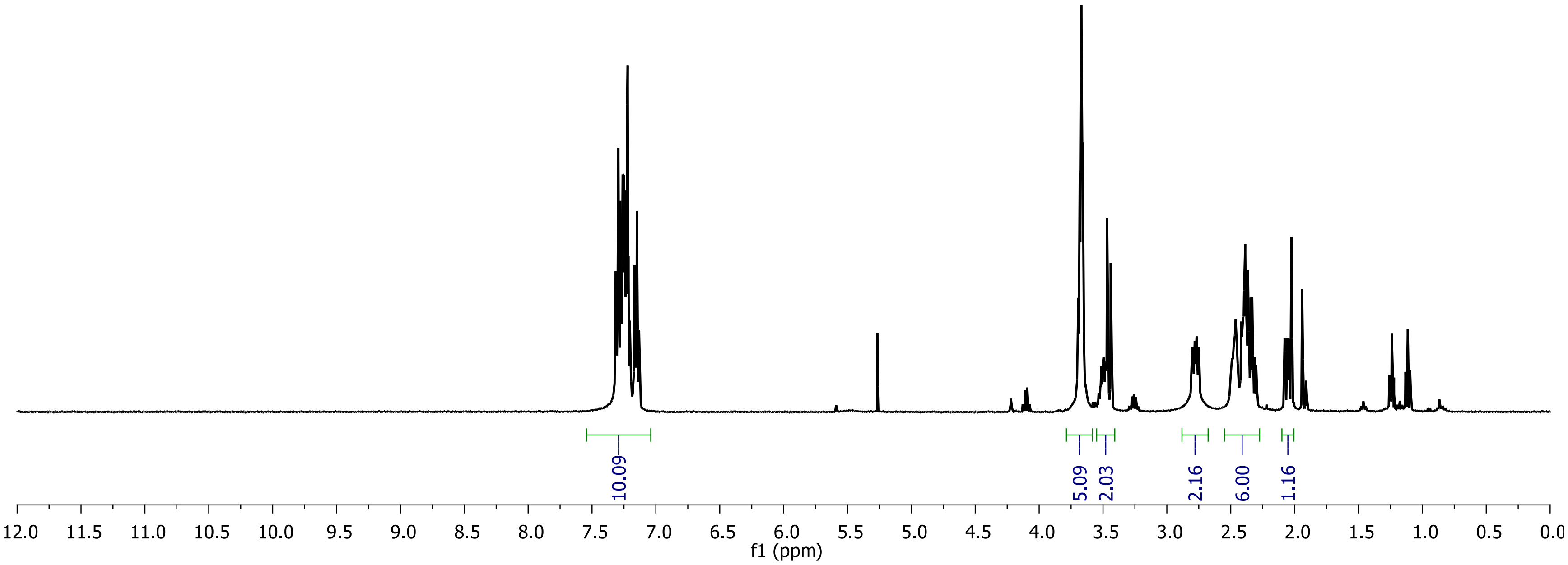
10.09
5.09
2.03
2.16
6.00
1.16
12.0 11.5 11.0 10.5 10.0 9.5 9.0 8.5 8.0 7.5 7.0 6.5 6.0 5.5 5.0 4.5 4.0 3.5 3.0 2.5 2.0 1.5 1.0 0.5 0.0
f1 (ppm)

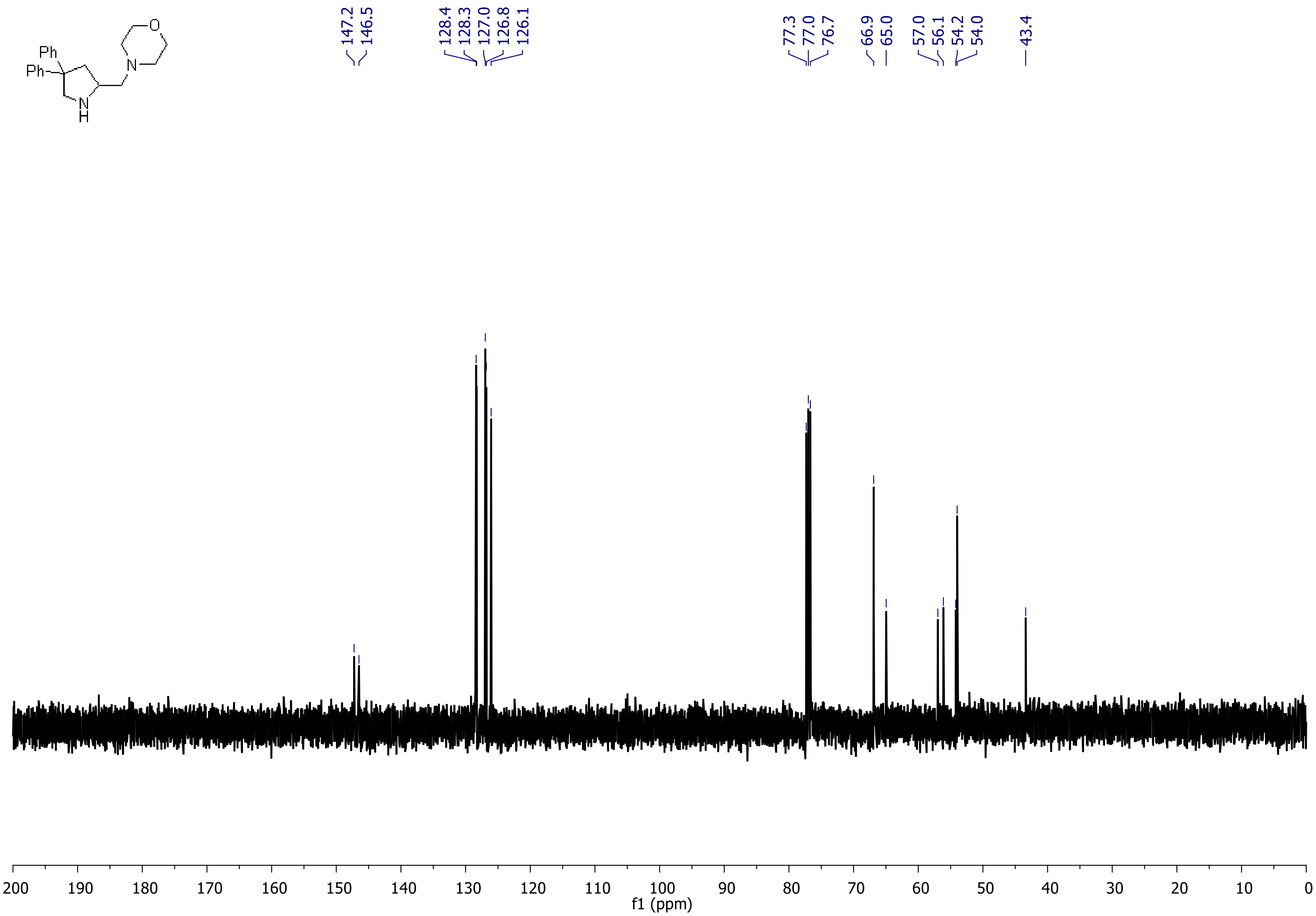

Ph
Ph
N
H
N
O
147.2
146.5
128.4
128.3
127.0
126.8
126.1
77.3
77.0
76.7
66.9
65.0
57.0
56.1
54.2
54.0
43.4
200 190 180 170 160 150 140 130 120 110 100 90 80 70 60 50 40 30 20 10 0
f1 (ppm)

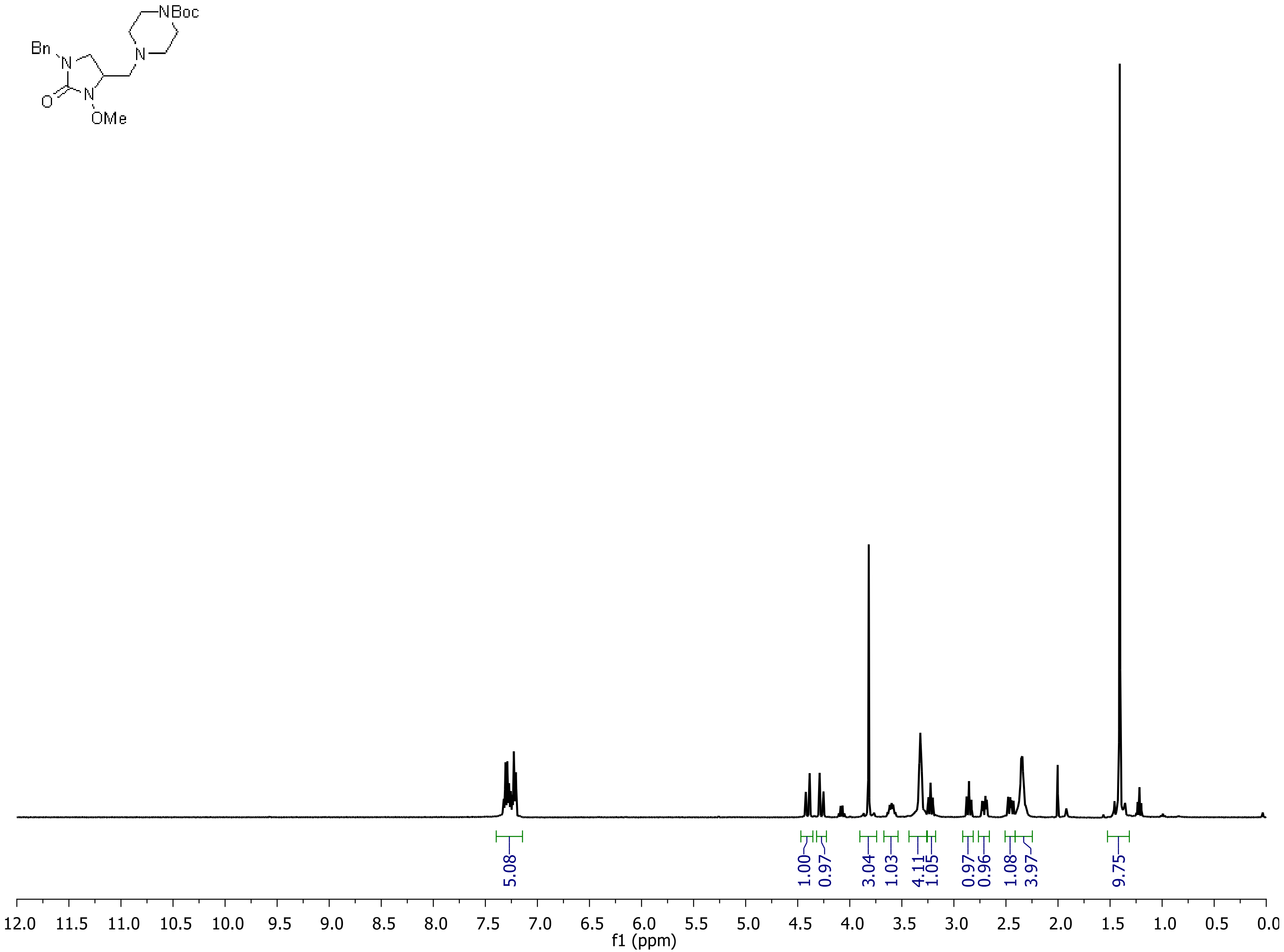
NBoc
Bn
N
N
O
N
OMe
5.08
1.00
0.97
3.04
1.03
4.11
1.05
0.97
0.96
1.08
3.97
9.75
12.0 11.5 11.0 10.5 10.0 9.5 9.0 8.5 8.0 7.5 7.0 6.5 6.0 5.5 5.0 4.5 4.0 3.5 3.0 2.5 2.0 1.5 1.0 0.5 0.0
f1 (ppm)

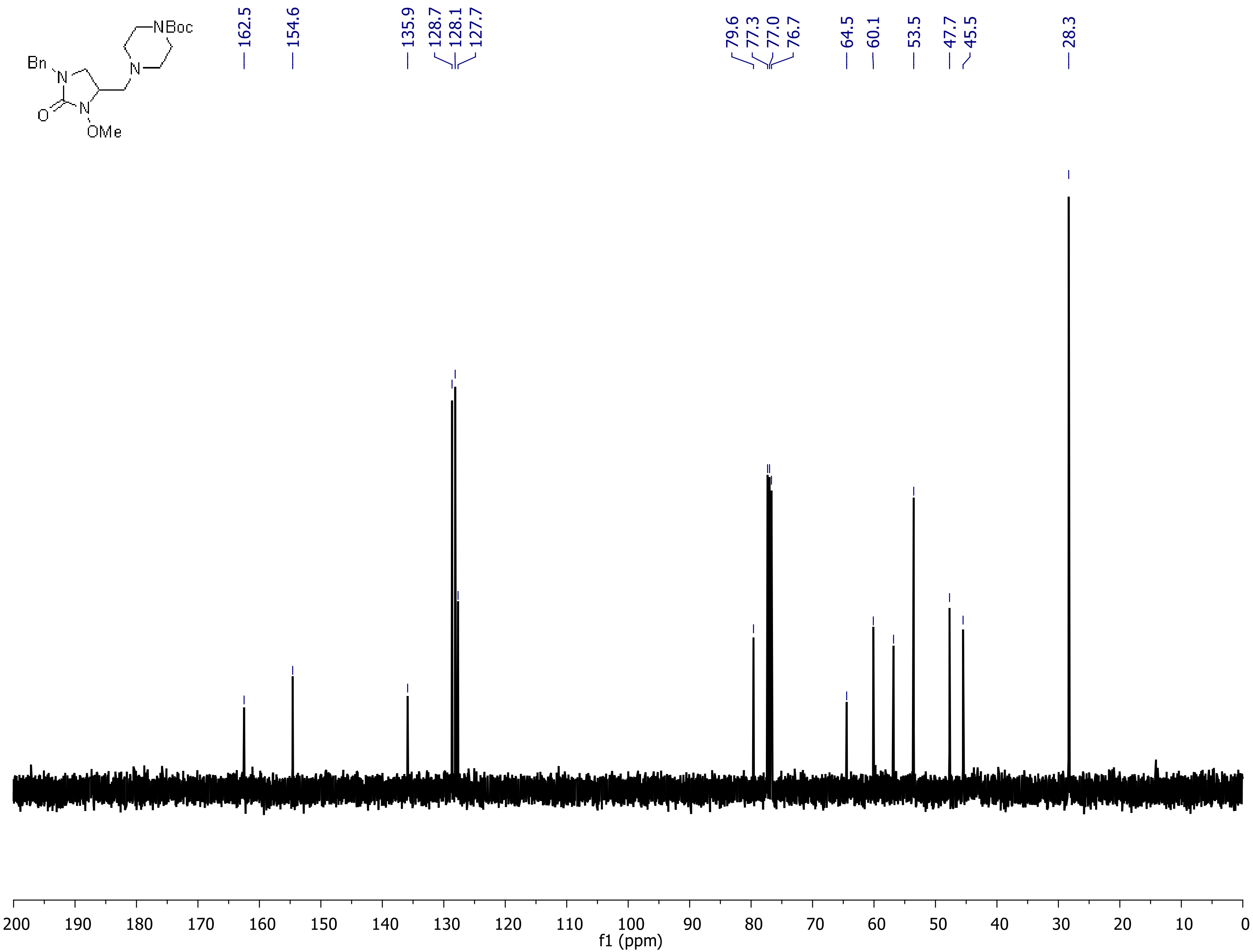

NBoc
Bn
N
N
O
N
OMe
162.5
154.6
135.9
128.7
128.1
127.7
79.6
77.3
77.0
76.7
64.5
60.1
53.5
47.7
45.5
28.3
200 190 180 170 160 150 140 130 120 110 100 90 80 70 60 50 40 30 20 10 0
f1 (ppm)

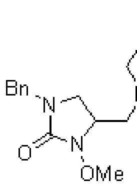
Ph
N
N
Bn
N
O
N
OMe

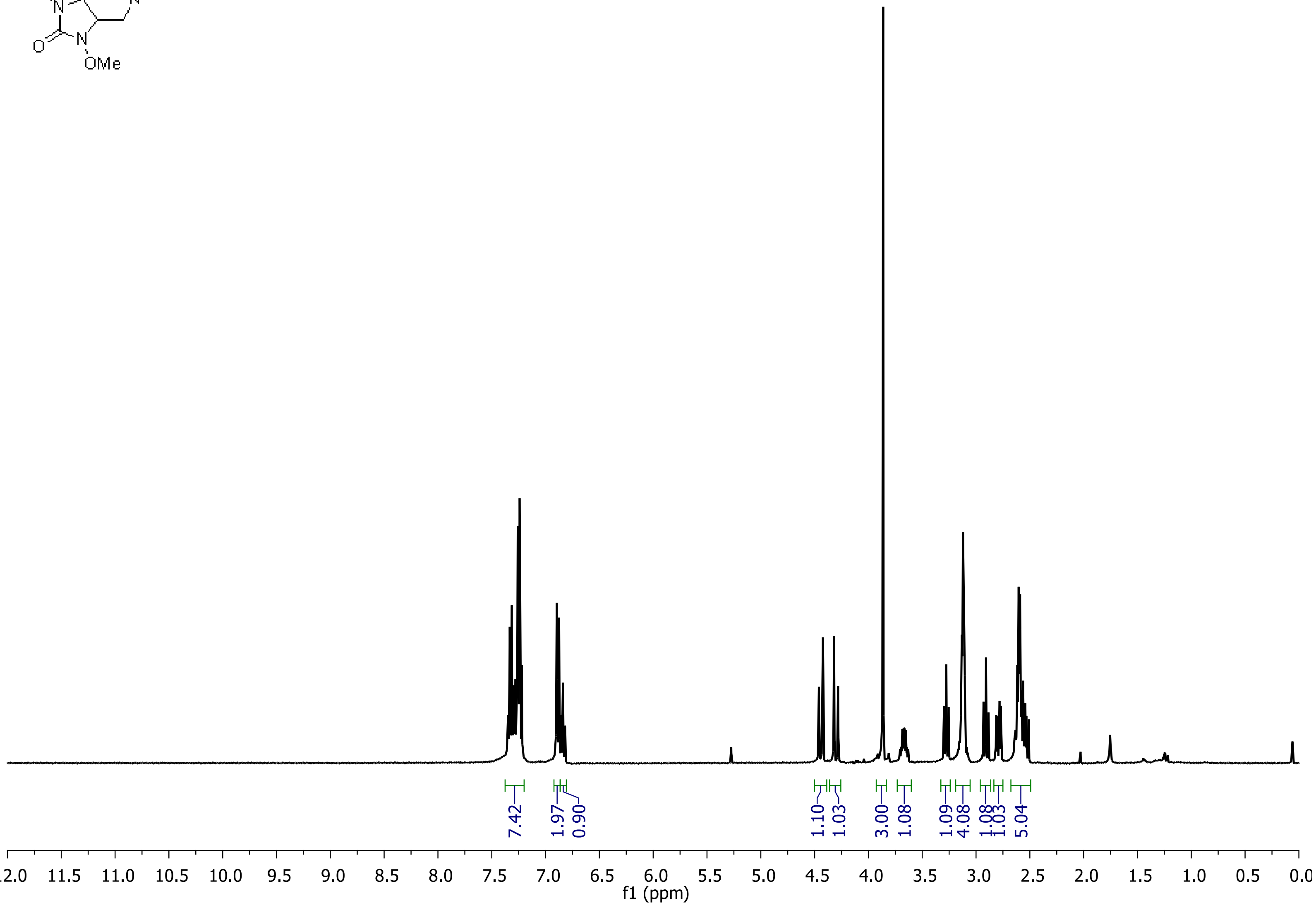
7.42
1.97
0.90
1.10
1.03
3.00
1.08
1.09
4.08
1.08
1.03
5.04
12.0 11.5 11.0 10.5 10.0 9.5 9.0 8.5 8.0 7.5 7.0 6.5 6.0 5.5 5.0 4.5 4.0 3.5 3.0 2.5 2.0 1.5 1.0 0.5 0.0
f1 (ppm)

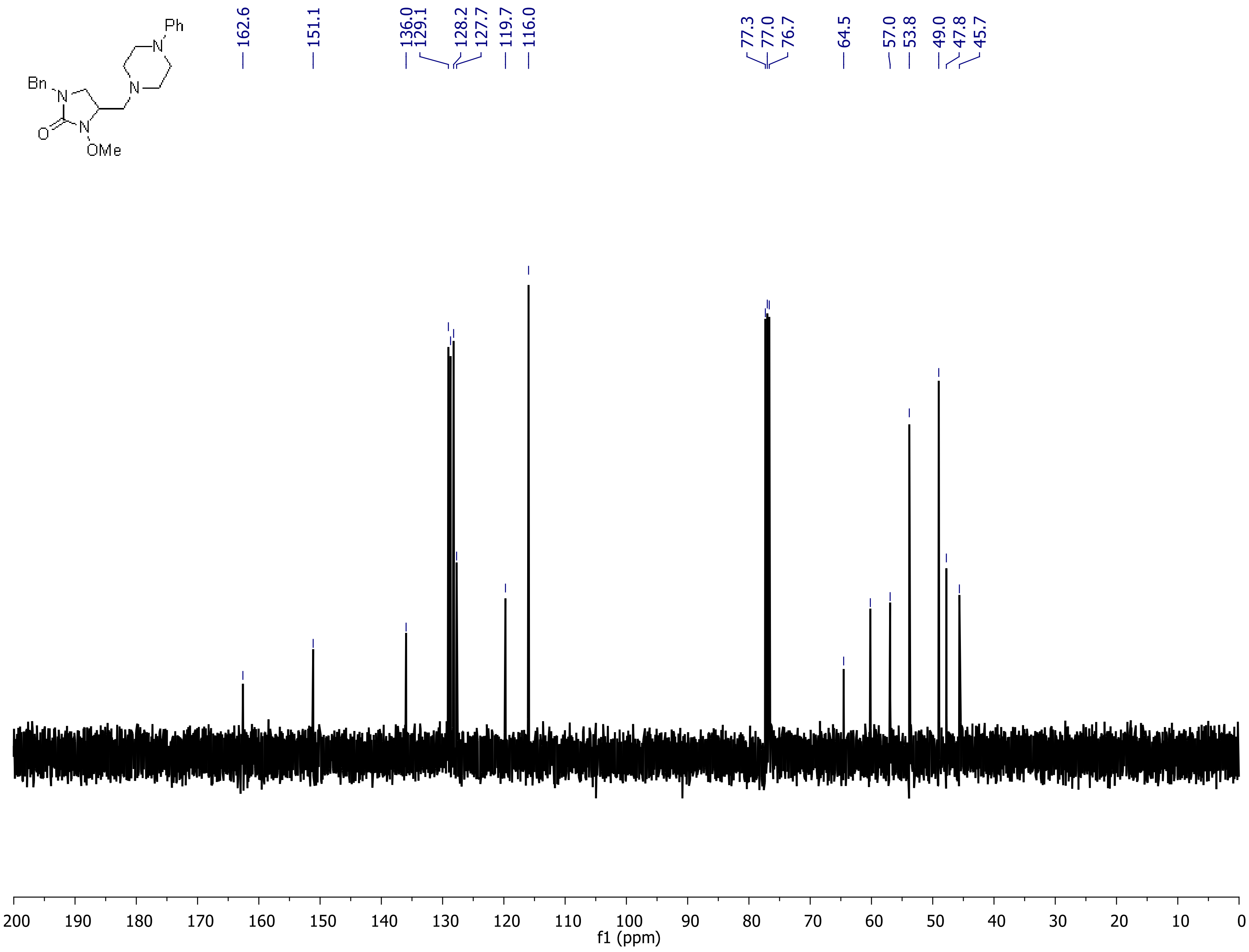
Ph
N
N
Bn
N
O
N
OMe
162.6
151.1
136.0
129.1
128.2
127.7
119.7
116.0
77.3
77.0
76.7
64.5
57.0
53.8
49.0
47.8
45.7
200 190 180 170 160 150 140 130 120 110 100 90 80 70 60 50 40 30 20 10 0
f1 (ppm)

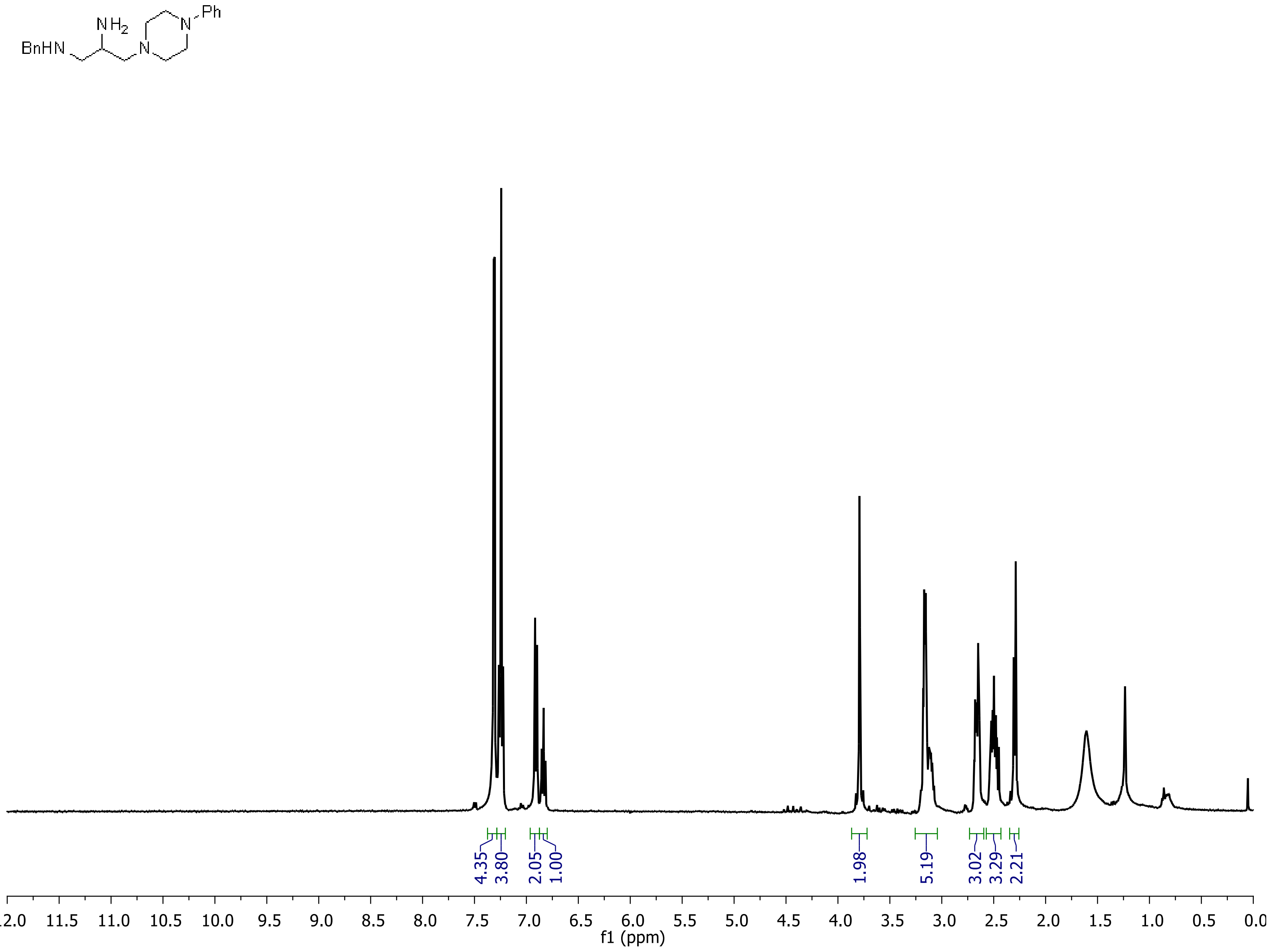

BnHN
NH2
N
N
Ph
4.35
3.80
2.05
1.00
1.98
5.19
3.02
3.29
2.21
12.0 11.5 11.0 10.5 10.0 9.5 9.0 8.5 8.0 7.5 7.0 6.5 6.0 5.5 5.0 4.5 4.0 3.5 3.0 2.5 2.0 1.5 1.0 0.5 0.0
f1 (ppm)

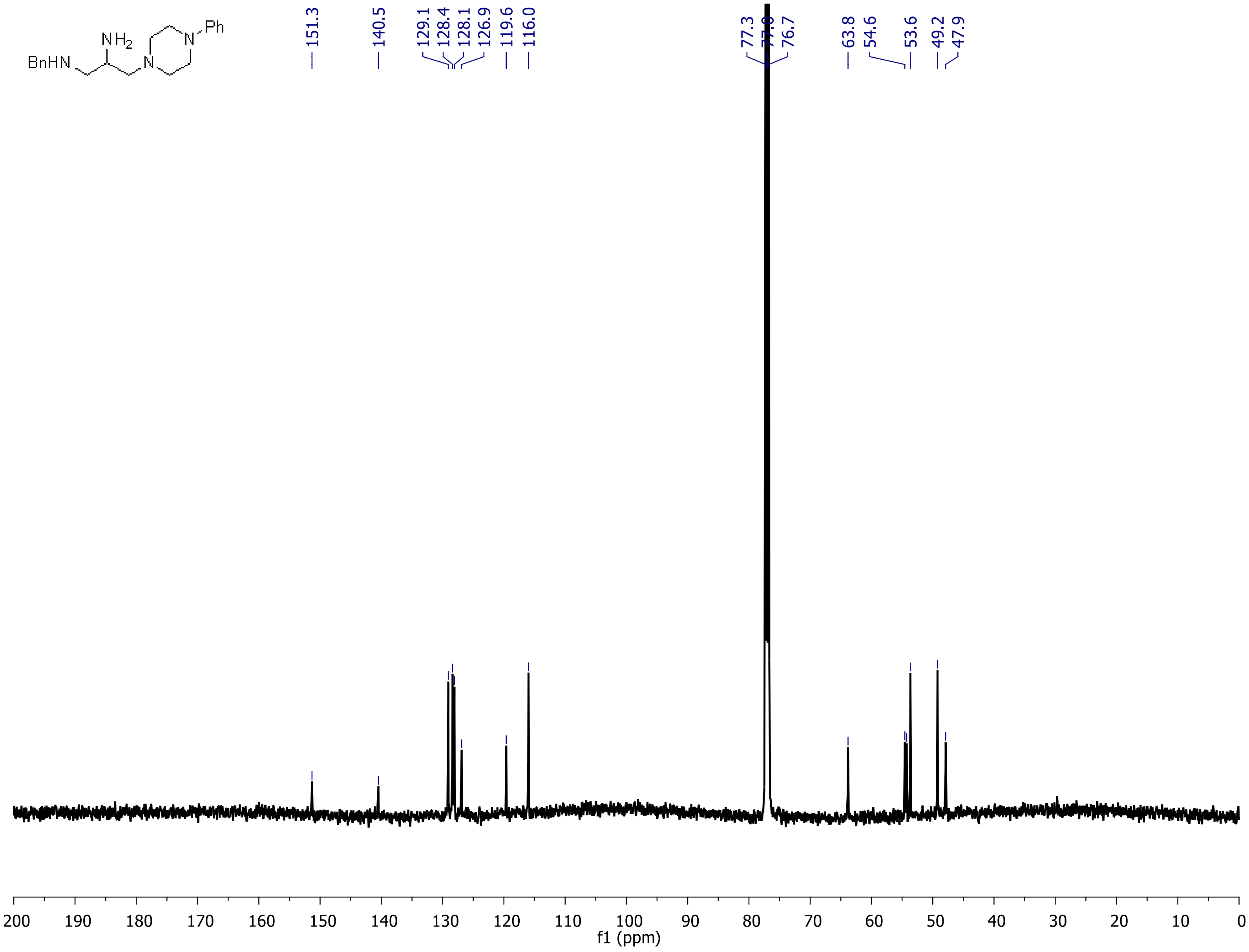

NH2
BnHN
N
N
Ph
151.3
140.5
129.1
128.4
128.1
126.9
119.6
116.0
77.3
77.0
76.7
63.8
54.6
53.6
49.2
47.9
200
190
180
170
160
150
140
130
120
110
100
90
80
70
60
50
40
30
20
10
0
f1 (ppm)

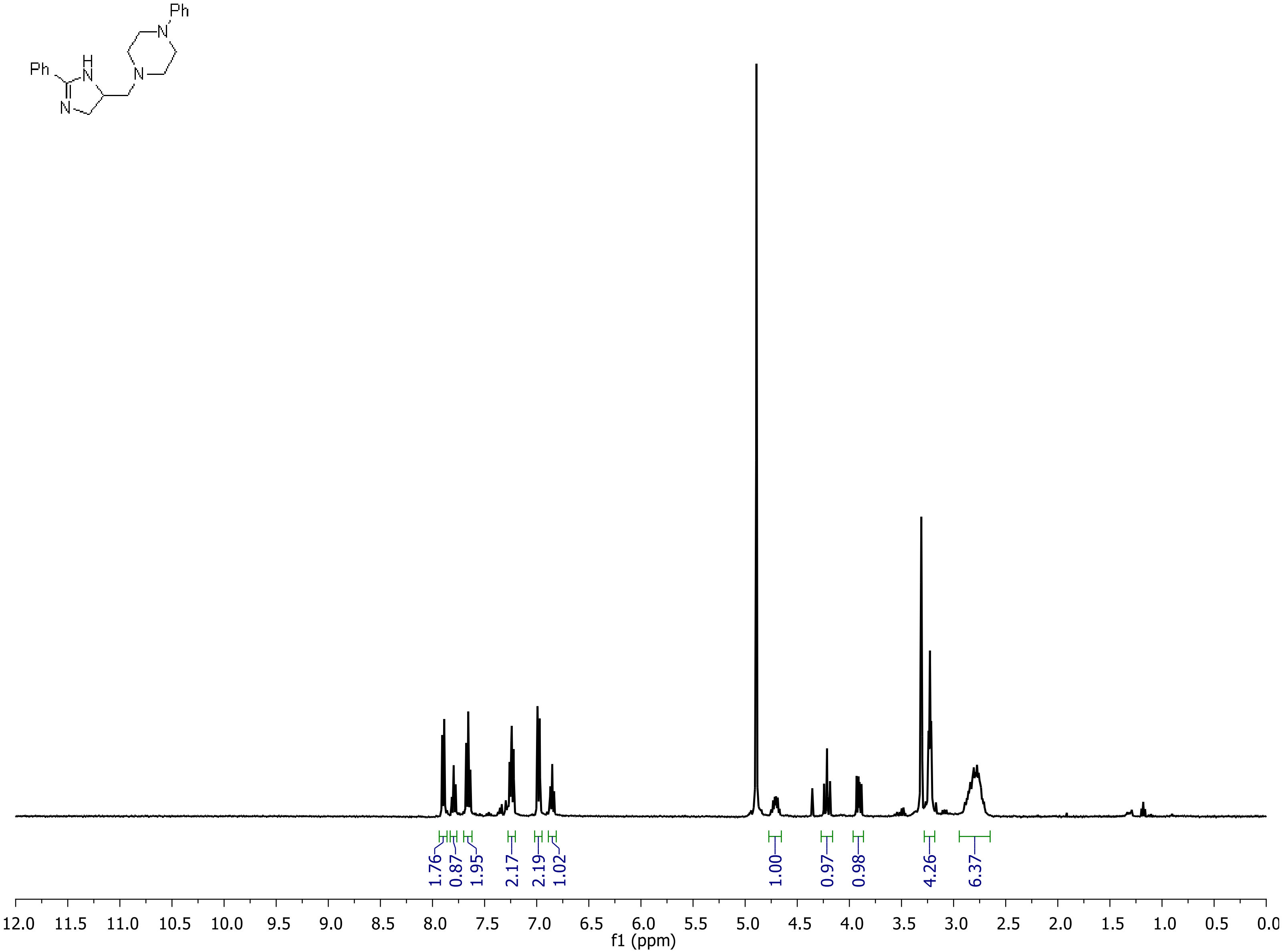

Ph
H
N
N
N
N
Ph
1.76
0.87
1.95
2.17
2.19
1.02
1.00
0.97
0.98
4.26
6.37
12.0
11.5
11.0
10.5
10.0
9.5
9.0
8.5
8.0
7.5
7.0
6.5
6.0
5.5
5.0
4.5
4.0
3.5
3.0
2.5
2.0
1.5
1.0
0.5
0.0
f1 (ppm)
